# New Zileuton-Hydroxycinnamic Acid Hybrids: Synthesis and Structure-Activity Relationship towards 5-Lipoxygenase Inhibition

**DOI:** 10.3390/molecules25204686

**Published:** 2020-10-14

**Authors:** Audrey Isabel Chiasson, Samuel Robichaud, Fanta J. Ndongou Moutombi, Mathieu P. A. Hébert, Maroua Mbarik, Marc E. Surette, Mohamed Touaibia

**Affiliations:** Department of Chemistry and Biochemistry, Université de Moncton, Moncton, NB E1A3E9, Canada; ai.chiasson@outlook.com (A.I.C.); esr4633@umoncton.ca (S.R.); fanta.ndongou.moutombi@umoncton.ca (F.J.N.M.); mathieu.hebert@umoncton.ca (M.P.A.H.); emm2954@umoncton.ca (M.M.); marc.surette@umoncton.ca (M.E.S.)

**Keywords:** 5-Lipoxygenase inhibitors, anti-leukotrienes, zileuton, sinapic acid, hydroxyurea, inflammation

## Abstract

A novel series of zileuton-hydroxycinnamic acid hybrids were synthesized and screened as 5-lipoxygenase (5-LO) inhibitors in stimulated HEK293 cells and polymorphonuclear leukocytes (PMNL). Zileuton’s (**1**) benzo[b]thiophene and hydroxyurea subunits combined with hydroxycinnamic acid esters’ ester linkage and phenolic acid moieties were investigated. Compound **28**, bearing zileuton’s (**1**) benzo[b]thiophene and sinapic acid phenethyl ester’s (**2**) α,β-unsaturated phenolic acid moiety **28,** was shown to be equipotent to zileuton (**1**), the only clinically approved 5-LO inhibitor, in stimulated HEK293 cells. Compound **28** was three times as active as zileuton (**1**) for the inhibition of 5-LO in PMNL. Compound **37**, bearing the same sinapic acid (3,5-dimethoxy-4-hydroxy substitution) moiety as **28**, combined with zileuton’s (**1**) hydroxyurea subunit was inactive. This result shows that the zileuton’s (**1**) benzo[b]thiophene moiety is essential for the inhibition of 5-LO product biosynthesis with our hydrids. Unlike zileuton (**1**), Compound **28** formed two π–π interactions with Phe177 and Phe421 as predicted when docked into 5-LO. Compound **28** was the only docked ligand that showed a π–π interaction with Phe177 which may play a part in product specificity as reported.

## 1. Introduction

In addition to playing a protective role in response to adverse stimuli, inflammation is potentially harmful in many chronic diseases when excessive [1]. By converting arachidonic acid (AA) to leukotrienes (LTs), which are potent lipid mediators [2], 5-Lipoxygenase (5-LO, EC1.13.11.34) is a key enzyme in the inflammatory cascade [3,4].

LTs are known to play an important role in the development and regulation of the inflammatory response [5]. Hence, LTs are involved in many diseases such as psoriasis [5,6], asthma [7], allergic rhinitis [8], atherosclerosis [9], Alzheimer’s disease [10], and various cancers [11,12,13].

**Figure 1:**
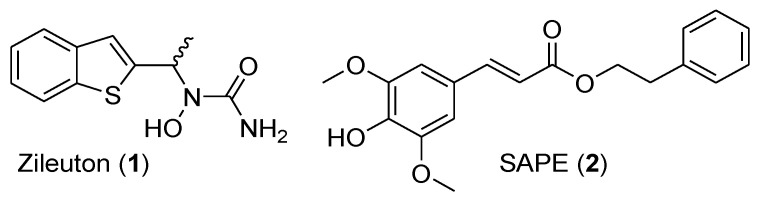
Structure of zileuton (**1**) and sinapic acid phenethyl ester (SAPE) (**2**).

Many efforts have been made to develop anti-leukotriene drugs, given the involvement of these mediators in many pathologies. Approved by the FDA in 1996, zileuton ((Zyflo^®^) (**1**); Figure 1) was prescribed as the first 5-LO inhibitor, which inhibits leukotriene (LTB_4_, LTC_4_, LTD_4_, and LTE_4_) biosynthesis. The original indication was to be used for prophylaxis and chronic treatment of asthma in adults and children over 12 years of age [14]. However, zileuton’s (**1**) dosing regimen as well as requirements for liver transaminase monitoring have limited its clinical use [15,16]. The limited use of the only approved 5-LO inhibitor combined to the rising demand for anti-LTs therapy, have resulted in the need to develop safe and efficient 5-LO inhibitors. 

Previous work from our team has shown that caffeic acid phenethyl ester (CAPE), a bioactive component of honeybee propolis, was significantly more potent than zileuton (**1**) for the inhibition of LTs biosynthesis in human polymorphonuclear leukocytes (PMNL, IC_50_ = 0.52 μM) [17]. Several phenolic acid-based analogues have been developed based on modifications of the caffeic moiety or of the ester linkage, and some have shown improved inhibition of leukotriene biosynthesis [18,19,20,21]. Moreover, we have recently demonstrated that sinapic acid phenethyl ester ((SAPE) (**2**), Figure 1) was more potent than zileuton (**1**) as 5-LO inhibitor (PMNL, IC_50_ = 0.3 μM), which highlights the importance of methoxy and hydroxyl groups on the cinnamic acid moiety [21].

Zileuton’s (**1**) benzo[b]thiophene moiety is a key pharmacophore that has been proven to act as an anti-inflammatory [22], antioxidant [23], anti-cancer [24], analgesic [22], and many more [23]. Therefore, several benzo[b]thiophene-based drugs have been developed over the years to treat numerous diseases [23]. *N*-hydroxyurea-Cetirizine hybrid, described by Lewis et al., showed histaminergic binding and 5-lipoxygenase inhibiting activities comparable to the corresponding *N*-hydroxyurea analog such as zileuton (**1**) [25]. Hydroxyurea alone is to date the only FDA approved drug for the treatment of adult patients with sickle cell disease through reduction of clinical complications [26].

**Figure 2:**
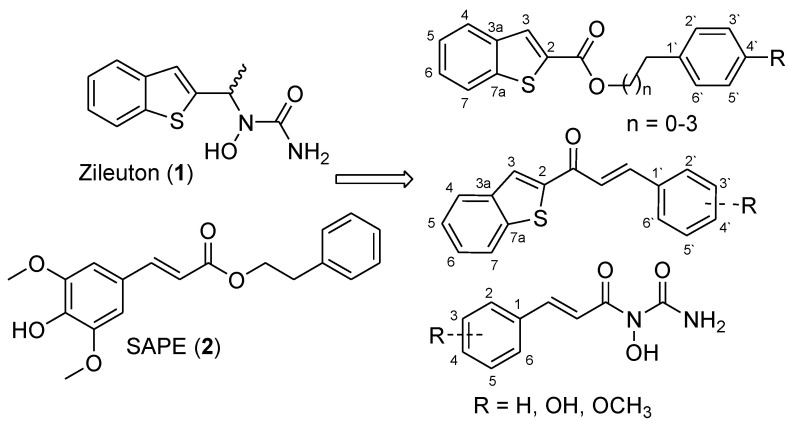
Designed compounds.

Herein, we now report the synthesis, characterization, and the anti 5-LO activity of new zileuton-hydroxycinnamic acid hybrids. Compounds that link zileuton’s (**1**) benzo[b]thiophene subunit and hydroxycinnamic acid ester’s two subunits were investigated (Figure 2). Compounds that link zileuton’s (**1**) hydroxyurea subunit and hydroxycinnamic acid ester’s acid subunit were also investigated (Figure 2). The influence of the methoxy and hydroxyl groups on the phenolic acid moiety was investigated by varying the number and position of the oxygen functions (hydroxy/methoxy groups). The influence of the α,β-unsaturation was also investigated.

## 2. Results and Discussion

### 2.1. Synthesis

**Scheme 1:**
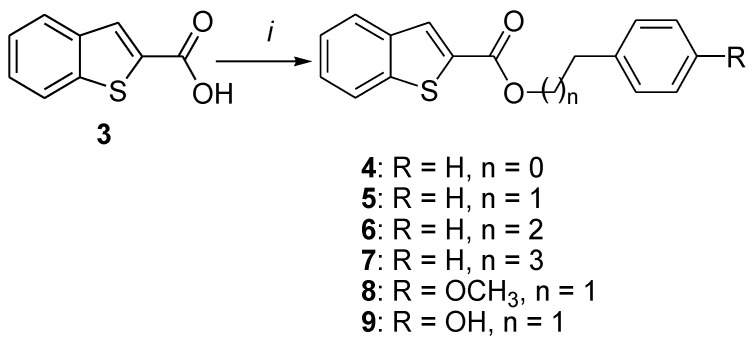
Synthesis of compounds **4–9**. Reagents and conditions: (i) Na_2_CO_3_, Ph(CH_2_)_n_Br, KI, hexamethylphosphoramide (HMPA), 0 °C (2 h) to rt (24 h).

Esters **4–9**, bearing the benzo[b]thiophene moiety, were obtained following a single step esterification of benzo[b]thiophene-2-carboxylic acid (**3**) with the appropriate alkyl bromide in the presence of sodium carbonate (Na_2_CO_3_), potassium iodide (KI), and hexamethylphosphoramide (HMPA) as a solvent. As shown in Scheme 1, the length of the linker and the presence of a methoxy or hydroxyl group on the ester moiety was investigated. The ^1^H-NMR analysis of this series shows the characteristic hydrogen of the benzo[b]thiophene (S-C=CH: 8 ppm) as well as the methylenes (4-2 ppm) of the linker ester.

**Scheme 2:**
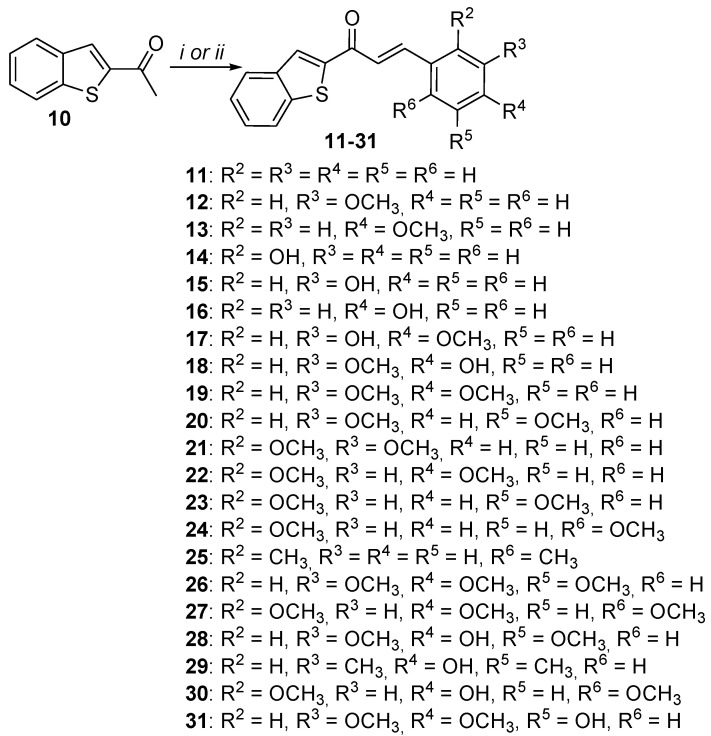
Synthesis of compounds **11–31**. Reagents and conditions: (i) C_2_H_5_ONa, C_2_H_5_OH, 30 min rt; (ii) pyrrolidine, CH_3_COOH, THF, (6–16 h), reflux.

Compounds **11–31** having benzo[b]thiophene and α,β-unsaturated carbonyl moieties were synthesized through a base-catalyzed aldol condensation by reacting the appropriate aldehyde with 2-acetylbenzothiophene (**10**) (Scheme 2). Our best yields were obtained by using pyrrolidine as a base. All our attempts to obtain compounds **11** and **12** using the same method in the presence of pyrrolidine failed which can be explained by the instability/non-reactivity of benzaldehyde and 3-methoxybenzaldehyde under such reaction conditions. Therefore, an alternative synthesis route for the aldol condensation using a stoichiometric quantity of sodium ethoxide in ethanol was used to obtain compounds **11** and **12** (Scheme 2). Unfortunately, all our attempts, whether with the first or the second method, failed to obtain dihydroxyl analogs. From the least substituted compound (**11**: R^2^-R^5^ = H) to compounds variously substituted with hydroxyl and methoxy (**12–31**), a total of 21 hybrids were obtained in this subseries (Scheme 2). Structures of compounds (**11–31**) were confirmed by NMR spectroscopy. In all compounds, ^1^H-NMR spectra showed the characteristic hydrogen of the benzo[b]thiophene (S-C=CH: 8 ppm). The characteristic α,β-unsaturation peaks were observed at 6–7 ppm. The value of the coupling constant (*J* = 16 Hz) confirms the trans stereochemistry of α,β-unsaturation. Further, ^13^C-NMR spectra also confirmed the presence of a carbonyl group by showing a peak near to 180 ppm.

**Scheme 3:**
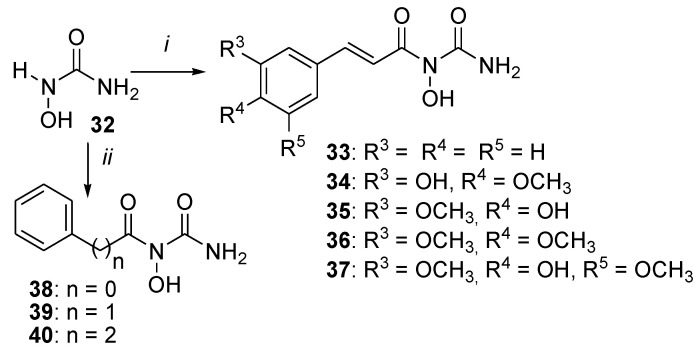
Synthesis of compounds **33–37** and **38–40**. Reagents and conditions: (i) carboxylic acid, (benzotriazol-1-yloxy)tris(dimethylamino)phosphonium hexafluorophosphate (BOP), Et_3_N, DMF, 0 °C (30 min) to rt (2 h); (ii) acyl chloride, 4-dimethylaminopyridine (DMAP), DMF, −45 °C (30 min).

An adaptation of the method described by Pariente-Cohen et al. [27] with our carboxylic acids allowed us to obtain the hydroxyurea hybrids **33–40** (Scheme 3). As described by Pariente-Cohen et al., *N*-acylation occurs through the reaction of hydroxylurea with our activated carboxylic acids. The acylation took place on the secondary nitrogen rather than on the hydroxyl group or the primary nitrogen. X-ray crystallography analysis of hydroxyurea reveals that the C–NH_2_ bond is shorter than the C–NHOH bond (1.328°A versus 1.347°A) [27,28] which suggest that the N of the –NHOH group is more nucleophilic since the C–NH_2_ contributes the dominant resonance structure and might have more sp2 hybridization.

Whether activated by a peptide coupling agent (benzotriazol-1-yloxy)tris(dimethylamino)phosphonium hexafluorophosphate (BOP) in this case, or by transformation into acyl chloride, cinnamic acid gave the corresponding hydroxy urea analog **33** (Scheme 3).

To avoid any side reactions that would be due to the presence of the hydroxyls of some of our carboxylic acids, the hydroxyurea hybrids **34–37** were obtained following activation in the presence of BOP. The benzoyl analog (**38**), obtained by Pariente-Cohen et al. starting with benzoic anhydride [27], was obtained with acyl chloride with a moderate yield. With the same procedure, phenylacetyl chloride and hydroxycinnamoyl chloride gave the corresponding hydroxyurea analogs **39** and **40** (Scheme 3).

### 2.2. Biological Evaluation

#### 2.2.1. 5-LO Product Biosynthesis Assays

**Figure 3:**
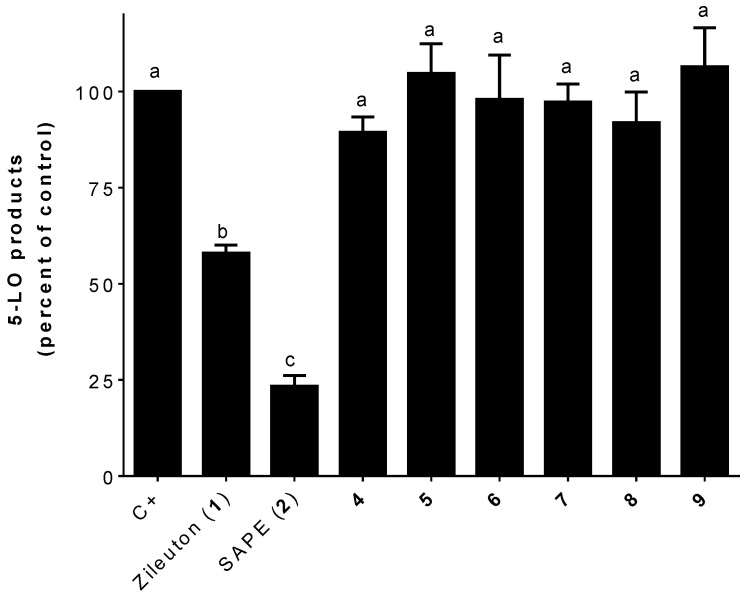
Inhibition of the biosynthesis of 5-lipoxygenase (5-LO) products in stimulated HEK293 cells by zileuton (**1**; 1 μM), SAPE (**2**; 1 μM), and **4–9** (1 μM). Data are expressed as means ± *SEM* of at least three independent experiments. Values without one common superscript (a, b, or c) are significantly different determined by one-way ANOVA with Tukey’s multiple comparison test (*p* < 0.05).

All compounds, including known inhibitors zileuton (**1**) and SAPE (**2**), were assayed at 1 µM (Figure 1) for inhibition of LTs biosynthesis in stimulated HEK293 cells. These cells are stably transfected with human 5-LO and serves as a highly reproducible model of 5-LO product biosynthesis in which compounds can be preliminarily screened for anti-LTs activity before moving on to more complex systems [17,29]. As shown in Figure 3, analogs bearing zileuton’s (**1**) benzo[b]thiophene subunit combined with a phen-alkyl moiety through an ester linkage were inactive. Esterification of benzo[b]thiophene-2-carboxylic acid to obtain benzyl to phenbutyl analogs (**4–7**) had no effect on the inhibitory activity of 5-LO. All four esters were inactive. Even the addition of a hydroxyl (**8**) or methoxy (**9**) at *para*-position of the phenethyl group has no effect on the activity.

**Figure 4:**
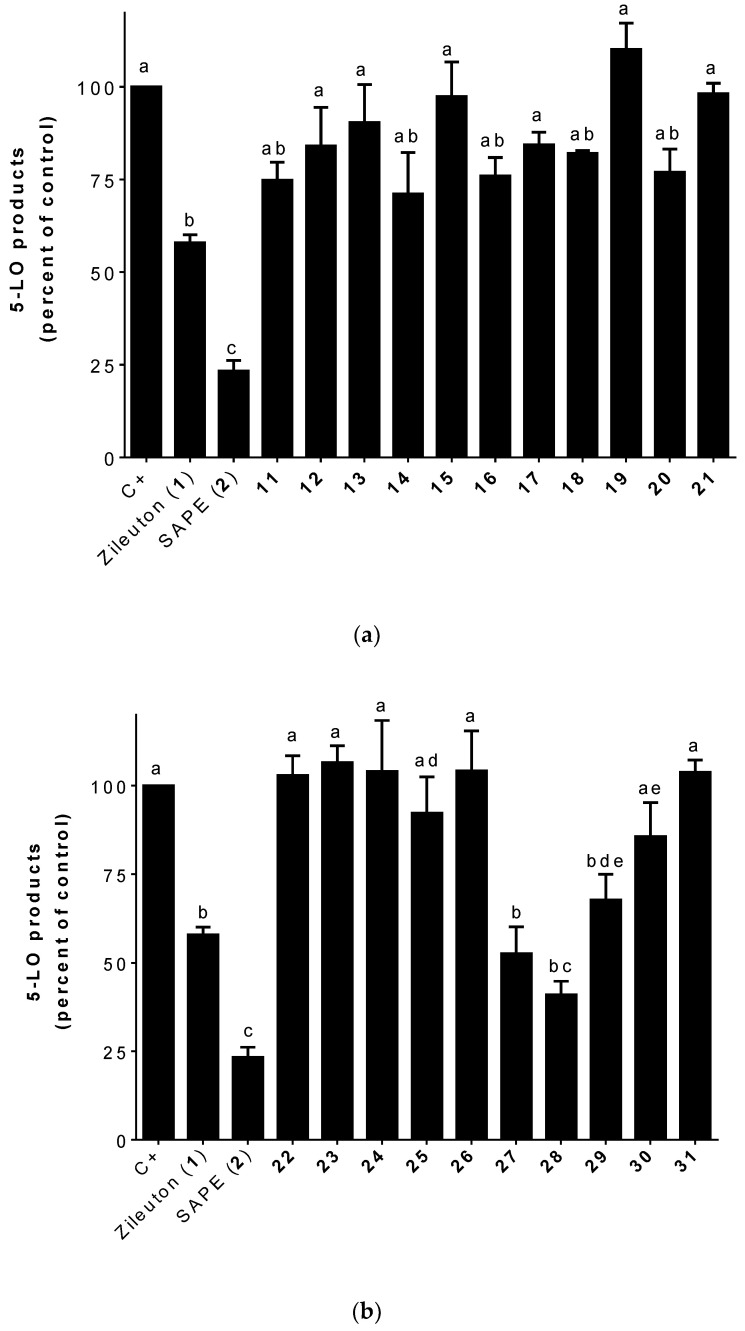
(**a**): Inhibition of the biosynthesis of 5-LO products in stimulated HEK293 cells by zileuton (**1**; 1 μM), SAPE (**2**; 1 μM), and **11–21** (1 μM). Data are expressed as means ± *SEM* of at least three independent experiments. Values without one common superscript are significantly different determined by one-way ANOVA with Tukey’s multiple comparison test (*p* < 0.05). (**b**): Inhibition of the biosynthesis of 5-LO products in stimulated HEK293 cells by zileuton (**1**; 1 μM), SAPE (**2**; 1 μM), and **22–31** (1 μM). Data are expressed as means ± *SEM* of at least three independent experiments. Values without one common superscript (a, b, c, d, or e) are significantly different determined by one-way ANOVA with Tukey’s multiple comparison test (*p* < 0.05).

Analogs bearing zileuton’s (**1**) benzo[b]thiophene subunit combined with a phenolic acid moiety through a α,β-unsaturated ketone had interesting results as shown in Figure 4a,b. The number and position of the oxygen functions (hydroxy/methoxy groups) seem to be critical for inhibition of 5-LO product biosynthesis in stimulated HEK293 cells (Figure 4a).

Unsubstituted compound (**11**) and *ortho*, *meta*, and *para*-monosubstituted (**12–16**) analogs were less active than SAPE (**2**) (Figure 4a). Compounds (**11**), **14** (*ortho*-), and **16** (*para*-) were non-significantly different than Zileuton (**1**) (Figure 4a). 

Like mono-substitution, di-substitution (**17–25**) does not seem to be beneficial for inhibition of 5-LO product biosynthesis in stimulated HEK293 cells. Hydroxyl and methoxy di-substituted compounds (**17–25**) at various positions were less active than SAPE (**2**) (Figure 4a,b). Being not-significantly different than zileuton (**1**) (Figure 4a), compounds **18** (3-OCH_3_; 4-OH) and **20** (3-OCH_3_; 5-OCH_3_) were the most active among analogs bearing zileuton’s (**1**) benzo[b]thiophene subunit combined with a di-substituted phenolic acid moiety.

Measuring the inhibition capacity of tri-substituted hybrids (**26–29**) shows that the number and position of the oxygen functions (hydroxy/methoxy groups) seem to be critical for the inhibition 5-LO product biosynthesis in HEK293 cells (Figure 4b). While compound **27**, bearing three methoxy moieties (positions 2, 4, and 6), was equipotent to Zileuton (**1**) (Figure 4b), compound **26** having three methoxy moieties (positions 3, 4, and 5) was inactive.

Interestingly, replacing the methoxy in the 4-position of compound **27** by a hydroxyl leads to the best inhibitor. Compound **28** (3,5-dimethoxy-4-hydroxy substitution) was non-significantly different than zileuton (**1**) and SAPE (**2**) (Figure 4b). It should be noted that compound **28** is a perfect hybrid of zileuton (**1**) and SAPE (**2**) (Figure 1) as it combines zileuton’s (**1**) benzo[b]thiophene and SAPE’s (**2**) α,β-unsaturated phenolic acid moieties (Scheme 2). The replacement of the two methoxy groups in positions 3 and 5 by two methyl groups does not seem to be favorable for the inhibition of 5-LO products as shown with compound **29**. Overall, the 3,5-dimethoxy-4-hydroxy tri-substitution, similar to that of SAPE (**2**), seems to be the most favorable for good inhibition (Figure 4b). Indeed, neither 2,6-dimethoxy-4-hydroxy (**30**) or 3,4-dimethoxy-5-hydroxy (**31**) substitutions appear to be as effective (Figure 4b).

**Figure 5:**
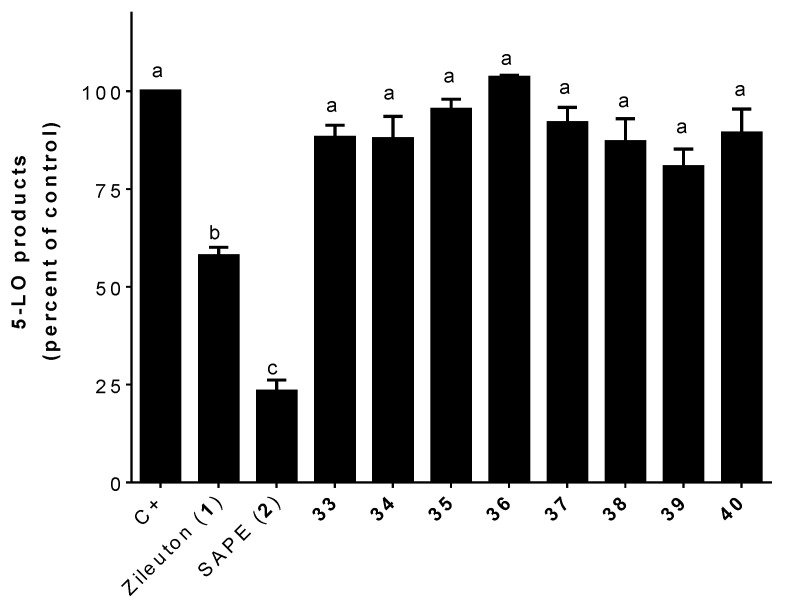
Inhibition of the biosynthesis of 5-LO products in stimulated HEK293 cells by zileuton (**1**; 1 μM), SAPE (**2**; 1 μM), and **33–40** (1 μM). Data are expressed as means ± *SEM* of at least three independent experiments. Values without one common superscript (a, b, or c) are significantly different determined by one-way ANOVA with Tukey’s multiple comparison test (*p* < 0.05).

Assays for the inhibition of 5-LO product biosynthesis by hybrids (**33–40**) combining zileuton’s (**1**) hydroxyurea subunit and hydroxycinnamic acid moieties show that the hydroxyurea subunit seems to not be critical for the inhibition of the biosynthesis of 5-LO products in stimulated HEK293 cells. As shown in Figure 5, the presence of hydroxyl and methoxy functions (OH and OCH_3_) does not seem to have an effect. Analogues obtained by condensation of hydroxyurea with cinnamic (compound **33**), ferulic (compound **34**), isoferulic (compound **35**), and dimethoxy cinnamic (compound **36**) acids were all inactive. Even compound **37** obtained with sinapic acid (3,5-dimethoxy-4-hydroxy substitution) was inactive. Of note, compound **37** had the same sinapic acid (3,5-dimethoxy-4-hydroxy substitution) moiety as the best inhibitor, compound **28**, obtained in series 2. This result shows that the zileuton’s (**1**) benzo[b]thiophene moiety is essential for the inhibition of the biosynthesis of 5-LO products with our hybrids. The linker between the hydroxylated nitrogen and the phenyl does not seem to be crucial for the inhibition of 5-LO products since analogs bearing the hydroxycinnamic acid (**40**), acetyl benzoic acid (**39**), or benzoic acid (**38**) moieties were inactive (Figure 5).

**Figure 6:**
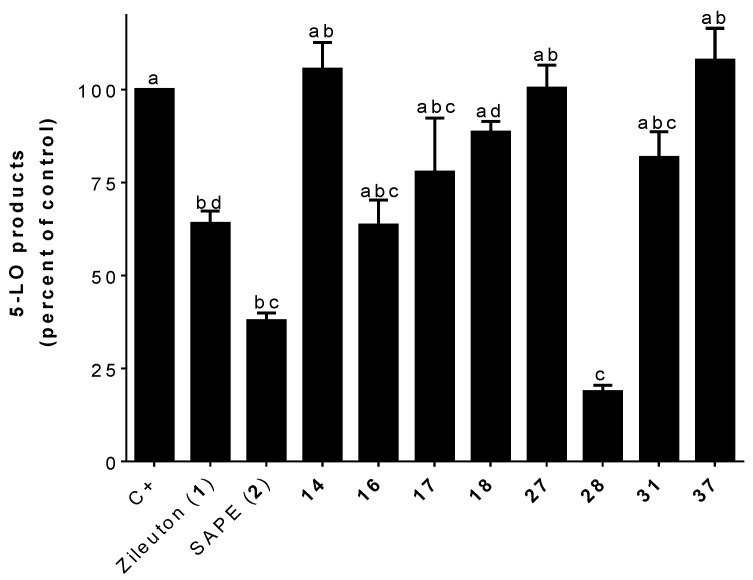
Inhibition of the biosynthesis of 5-LO products in human polymorphonuclear leukocyte (PMNL) cells by zileuton (**1**; 1 μM), SAPE (**2**; 1 μM), **14**, **16–18, 27, 28**, **31,** and **37** (1 μM). Data are expressed as means ± SEM of at least three independent experiments. Values without one common superscript (a, b, c, or d) are significantly different determined by one-way ANOVA with Tukey’s multiple comparison test (*p* < 0.05).

To further probe the inhibitory activity of compounds that approached the inhibitory activity of zileuton (**1**) and SAPE (**2**), compounds were selected for further screening in PMNL (Figure 6). 5-LO is highly expressed in PMNL and these cells are important physiological producers of LTB_4_ [17]. Compound **28** was shown to be the best inhibitor exceeding zileuton’s (**1**) inhibitory capacity in PMNL by more than three times. The calculated IC_50_ value for the inhibition of 5-LO product biosynthesis in PMNL for compound **28** was 0.37 μM (95% confidence interval: 0.25–0.53 μM). The presence of the hydroxyl group in position 4 and the methoxy groups in positions 3 and 5 seems to be an ideal combination for inhibition of 5-LO (Figure 6). These structural changes may influence the availability, cell permeation, or even the stability of inhibitors in PMNL versus HEK293 cells. A comparison of the two monosubstituted analogs, compounds **14** and **16**, reveals that hydroxyl at position 4 (**16**) seems to be more favorable for the inhibition of 5-LO, although neither compound showed significant inhibition compared to controls. As in HEK293 cells, disubstituted compounds **17** and **18** were less active than zileuton (**1**) and SAPE (**2**) (Figure 6).

A position interchange between the hydroxyl at position 4 and a methoxy at position 5 of compound **28**, as in compound **31**, leads to a total loss of activity (Figure 6). The substitution of the hydroxyl at position 4 of compound **28** by a methoxy, as in compound **27**, also leads to a complete loss of the 5-LO inhibition (Figure 6). The inactivity of hybrid **37**, as in HEK293 cells, shows that the zileuton’s (**1**) benzo[b]thiophene moiety is essential for the inhibition of 5-LO product biosynthesis with our hydrids.

#### 2.2.2. Free Radical Scavenging Activity Assay

A mechanism by which 5-LO activity can be inhibited is through reductive inhibition of the ferric non-heme iron of the enzyme. Interactions with stable free radicals provides an evaluation of the reducing ability of the test compounds. Radical scavenging activities of compounds tested in stimulated PMNL were assayed using 2,2-Diphenyl-1-picrylhydrazyl (DPPH) as a stable radical and are expressed as IC_50_ concentrations in Table 1. Mono- and di-substituted analogs (**14**, **16**, **17,** and **18**), which were non-significantly different than zileuton (**1**) in stimulated HEK293 cells (Figure 4a) had a weak anti-free radical activity compared to that of SAPE (**2**) or vitamin C but more important than that of zileuton (**1**). Among the three tri-substituted hybrids tested, compounds **27** and **31** were the most active with IC_50_ of 6.0 µM and 6.6 µM, respectively (Table 1).

Anti-radical activity of selected compounds indicates that this ability does not appear to be crucial for 5-LO inhibition by the tested compounds. Indeed, even though it was the best inhibitor of the whole series for the inhibition of 5-LO product biosynthesis in stimulated HEK293 and human PMNL cells, compound **28** had weak anti-free radical activity compared to SAPE (**2**) and vitamin C (Table 1). Compounds **31** and **37,** bearing zileuton’s (**1**) hydroxyurea subunit instead of benzo[b]thiophene subunit as compound **28**, were among the best compounds with antiradical activity (Table 1) while inactive for the inhibition of the biosynthesis of 5-LO products in both stimulated HEK293 and human PMNL cells.

### 2.3. Molecular Docking

To predict, at a molecular level, the possible interactions between 5-LO and the best ligand reported in this study, compound **28** and its related analogs **31** and **37** were docked into 5-LO (PDB ID: 3O8Y [30]). The unmodified apo structure of 5-LO, 3O8Y. “Stable-5-LO” is mutated mostly on the non-catalytic domain with a small exception of a short 3 residues sequence being mutated on the catalytic domain [30]. While these mutations may affect the structure, test results showed that catalytic fidelity was conserved [30]. This crystallized structure was used by many groups as well as our group for molecular modeling [19,20,21,31]. Zileuton (**1**) and SAPE (**2**) were docked as references. All docked compounds had better affinity for the active site than zileuton (1) and SAPE (2). As reported by De Lucia et al. [32] and demonstrated by our group recently [21], zileuton (**1**) was located in the inner part of the binding pocket and is stabilized by hydrogen bonds with Leu420, Ala424, Phe421, and Asn425 (Table 2). SAPE (**2**) was stabilized by the formation of a π–π interaction with His372 [21].

With a lower predicted binding energy than zileuton (1), compound **28** had a better affinity for 5-LO than zileuton (**1**) (Table 2). Compound **28**, as with SAPE (**2**), does not form any hydrogen bonds with the 5-LO pocket, unlike to zileuton (**1**). However, compound **28** forms two π–π interactions with Phe177 and Phe421, which can explain its superiority as an inhibitor compared to zileuton (**1**). Compound **28** was the only docked ligand that showed a π–π interaction with Phe177 which may play a part in product specificity [33].

**Figure 7:**
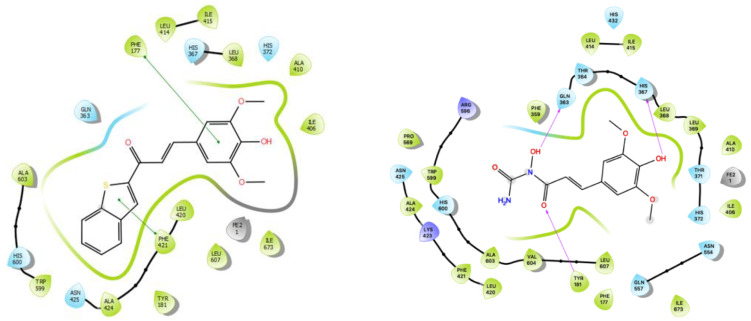
Visual of compound **28** (left: π–π interactions with Maestro) and compound **37** (right: hydrogen bonds with Maestro) docked with 5-LO.

Interestingly, zileuton’s (**1**) benzo[b]thiophene moiety of compound **28**, the most active inhibitor in human PMNL cells, is in close proximity to Leu420, Ala424, and Asn425 (Figure 7). This binding site has been reported previously for fungal 5-LO inhibitors [34]. Unlike compound **28**, compound **37** forms three hydrogen bonds with Tyr181, Gln363, His367, and Asn425. The last amino acid was also involved in a hydrogen bond with zileuton (**1**). Given its weak inhibitory activity of 5-LO in stimulated HEK293 and human PMNL cells, this implies that these interactions are not favorable for an optimal inhibition.

### 2.4. In Silico Physicochemical Properties and Druglikeness Evaluation

Absorption, distribution, metabolism, and excretion parameters (ADME) of compound **28** and its related analog **37** were predicted using the web tool SwissADME (http://www.swissadme.ch). Zileuton’s (**1**) and SAPE’s (**2**) properties were also predicted (Table 3).

As with zileuton (**1**) and SAPE (**2**), compound **28** obeys Lipinski’s Rule of Five, which predicts the drug likeness of a molecule based on its physicochemical properties [35]. Moreover, all evaluated compounds seem to present a high oral bioavailability as they all have a topological surface area (TPSA) ≤140 Å. Compound **28** showed high gastrointestinal absorption (GIA) and no blood–brain barrier permeation as with zileuton (**1**) (Table 3).

## 3. Materials and Methods

### 3.1. General Synthetic Experimental Procedures

Chemicals were purchased from Sigma-Aldrich and Alfa Aesar. The synthesized compounds were purified by flash chromatography (Isco, Inc. CombiFlash^®^ Sg100c). Thin layer chromatography (TLC) was carried out on silica gel-coated aluminum sheets (EMD Millipore^TM^ TLC Silica Gel 60) with detection by UV light (245 nm, UVP^®^ UVS-14 EL Series UV lamp). Melting points were obtained with a melting point apparatus (MELTEMP^®^ 1001D). NMR spectra were recorded on Bruker^®^ Avance III 400 MHz spectrometer with TMS as an internal standard. High-resolution mass measurements were performed on Bruker^®^ Doltonics’ micrOTOF instrument in positive or negative electrospray. Analytical high-performance liquid chromatography (HPLC) was performed on an Agilent Technologies system (Agilent1100 Series) with an ACE C18 column (150 mm × 4.6 mm, 5µ) using miliQ water (A) and HPLC grade methanol (B) as mobile phase in a gradient mode (gradient steps: 75:25 (A:B; 1 min), 15:85 (A:B; 7 min), and 75:25 (A:B; 7 min); flow rate of 1.0 mL/min, detection at 320 nm). A purity of >95% has been established for all tested compounds.

#### 3.1.1. General Procedure for the Esterification of Thianaphtene-2-carboxylic Acid

Thianaphtene-2-carboxylic acid (**3**) (1 eq) was added to a vigorously stirred solution of Na_2_CO_3_ (1.2 eq) in 7 mL of HMPA and the reaction vessel was flushed with argon. After 30 min of stirring, the appropriate bromide (1.2 eq) was added dropwise to the reaction mixture over a period of 10 min. A catalytic amount of KI was added to the reaction vessel, which is then thoroughly flushed with argon gas and sealed under balloon pressure. The reaction mixture was stirred in an ice bath for 2 h and at room temperature for 24 h under argon atmosphere. The resulting solution was quenched with ice/water and stirred for 30 min. The aqueous phase was extracted with EtOAc (3 × 50 mL). The combined organic fractions were then washed with brine (3 × 50 mL), decolorized with activated charcoal, dried over MgSO_4_, and concentrated under vacuum. The resulting crud product was purified by flash chromatography.

##### (2,4,6-Cycloheptatrien-1-yl)methyl 1-benzothiophene-2-carboxylate (**4**)

Following our general procedure of esterification with thianaphtene-2-carboxylic acid (**3**) (500 mg, 2.81 mmol, 1 eq), Na_2_CO_3_ (356 mg, 3.36 mmol, 1.2 eq), and benzyl bromide (0.40 mL, 3.36 mmol, 1.2 eq), compound **4** was obtained as a white solid after flash chromatography (10% EtOAc/Hex), yield = 80%, Rf = 0.31 (10% EtOAc/Hex), m.p. = 86–87 °C. ^1^H-NMR (400 MHz, CDCl_3_, 25 °C); δ (ppm): 8.12 (s, 1H, H3), 7.91–7.88 (m, 2H, H4, H7), 7.51–7.37 (m, 7H, H5, H6, H2′, H3′, H4′, H5′, H6′), 5.42 (s, 2H, OCH_2_C). ^13^C-NMR (101 MHz, CDCl_3_, 25 °C); δ (ppm): 162.66 (C=O), 142.32 (C7a), 138.71 (C3a), 135.65 (C2), 133.45 (C1′), 130.78 (C3′, C5′), 128.66 (C4′), 128.41 (C2′, C6′), 126.23 (C6), 127.00 (C5), 125.58 (C4), 124.94 (C7), 122.77 (C3), 67.14 (CH_2_). HRMS *m/z* calc. for C_16_H_12_O_2_S + (H^+^): 269.0631; found: 269.0628.

##### Phenethyl 1-benzothiophene-2-carboxylate (**5**)

Following our general procedure of esterification with thianaphtene-2-carboxylic acid (**3**) (500 mg, 2.81 mmol, 1 eq), Na_2_CO_3_ (356 mg, 3.36 mmol, 1.2 eq), and (2-bromoethyl)benzene (0.46 mL, 3.36 mmol, 1.2 eq), compound **5** was obtained as a yellow solid after flash chromatography (10% EtOAc/Hex), yield = 74%, m.p.= 61–62 °C, Rf = 0.37 (10% EtOAc/Hex). ^1^H-NMR (400 MHz, CDCl_3_, 25 °C); δ (ppm): 8.07 (s, 1H, H3), 7.91–7.88 (m, 2H, H4, H7), 7.51–7.27 (m, 7H, H5, H6, H2′, H3′, H4′, H5′, H6′), 4.60–4.57 (t, 2H, J = 7.0 Hz, OCH_2_CH_2_), 3.15–3.11 (t, 2H, J = 7.0 Hz, CH_2_CH_2_C). ^13^C-NMR (101 MHz, CDCl_3_, 25 °C); δ (ppm): 162.71 (C=O), 142.27 (C7a), 138.71 (C3a), 137.66 (C2), 133.59 (C1′), 130.56 (C3′, C5′), 129.05 (C2′, C6′), 128.60 (C4′), 126.95 (C6), 126.70 (C5), 125.56 (C7), 124.91 (C4), 122.77 (C3), 66.02 (OCH_2_), 35.23 (CH_2_CH_2_C). HRMS *m/z* calc. for C_17_H_14_O_2_S + (H^+^): 282.0787; found: 283.0785.

#### 3.1.2. General Procedure for the Aldol Condensation of 2-acetylbenzothiophene in Presence of Sodium Ethoxide

To a solution of 2-acetylbenzothiophene (**10**) (1 eq) and the appropriate aldehyde (1.2 eq) in ethanol (15 mL) was added sodium ethoxide (1 eq). The mixture was then stirred at room temperature for 2 h. After concentration under vacuum, EtOAc (50 mL) was added. The resulting solution was stirred with sodium bisulfite solution (100 mL, 120 g/500 mL) for 30 min to remove any traces of starting aldehyde. After separation, the combined organic phase was washed with brine, decolorized with activated charcoal, dried over MgSO_4_, filtered, and concentrated under vacuum. The resulting crud product was purified by flash chromatography.

##### (*E*)-1-(1-Benzothiophen-2-yl)-3-phenyl-2-propen-1-one (**11**)

Following our general procedure for the aldol condensation in the presence of sodium ethoxide with 2-acetylbenzothiophene (**10**) (250 mg, 1.42 mmol, 1 eq), sodium ethoxide (97 mg, 1.42 mmol, 1 eq), and benzaldehyde (252 mg, 1.70 mmol, 1.2 eq), compound **11** was obtained as a yellow solid after flash chromatography (8% EtOAc), yield = 35%, Rf = 0.23 (10% EtOAc), m.p. = 118–120 °C. ^1^H-NMR (400 MHz, CDCl_3_, 25 °C); δ (ppm): 8.14 (s, 1H, H3), 7.96–7.92 (m, 2H, H4, H7), 7.94–7.91 (d, 1H, J = 16 Hz, CH=CHC), 7.72–7.70 (m, 2H, H5, H6), 7.60–7.56 (d, 1H, J = 16 Hz, COCH=CH), 7.53–7.43 (m, 5H, H2′, H3′, H4′, H5′, H6′). ^13^C-NMR (101 MHz, CDCl_3_, 25 °C); δ (ppm): 183.44 (C=O), 145.20 (C7a), 144.42 (C2), 142.72 (COCH=CH), 139.28 (C3a), 134.67 (C1′), 130.78 (C3′, C5′), 129.03 (C4′), 128.88 (C2′, C6′), 128.60 (C6), 127.44 (C5), 125.99 (C4), 125.05 (C7), 123.03 (C3), 121.14 (COCH=CH). HRMS *m/z* calc. for C_17_H_12_OS + (H^+^): 265.0682; found: 265.0684.

#### 3.1.3. General Procedure for the Base-Catalyzed Aldol Condensation of 2-acetylbenzothiophene

To a stirred solution of 2-acetylbenzothiophene (**10**) (1.2 eq) and the appropriate aldehyde (1 eq) in 10 mL of THF was added 100 μL of pyrrolidine and a catalytic amount of acetic acid (4 drops). The solution was refluxed under argon atmosphere and monitored by TLC. Solvent was removed under vacuum, Water (50 mL) was added followed by extraction with EtOAc (3 × 25 mL). The combined organic fractions were then combined and stirred with sodium bisulfite solution (100 mL, 120 g/500 mL) for 30 min to remove any traces of starting aldehyde. After separation, the combined organic fractions were washed with brine, dried over MgSO_4_, decolorized with activated charcoal, filtered, and concentrated under vacuum. The resulting crud product was purified by flash chromatography.

##### (*E*)-1-(1-Benzothiophen-2-yl)-3-(p-methoxyphenyl)-2-propen-1-one (**13**)

Following our base-catalyzed aldol condensation general procedure with 2-acetylbenzothiophene (**10**) (250 mg, 1.42 mmol, 1.2 eq) and 4-methoxybenzaldehyde (161 mg, 1.18 mmol, 1 eq) under reflux for 10h, compound **13** was obtained as a yellow solid after flash chromatography (10% EtOAc/Hex), yield = 19%, Rf = 0.39 (20% EtOAc/Hex), m.p. = 112–115 °C. ^1^H-NMR (400 MHz, CDCl_3_, 25 °C); δ (ppm): 8.12 (s, 1H, H3), 7.96–7.93 (d, 2H, J = 8 Hz, H4, H7), 7.92–7.88 (d, 1H, J = 15 Hz, CH=CHC), 7.68–7.66 (d, 2H, J = 7 Hz, H5, H6,), 7.51–7.42 (m, 2H, H6′, H2′), 7.46–7.43 (d, 1H, J = 15 Hz, COCH=CH), 6.99–6.97 (d, 2H, J = 9 Hz, H3′, H5′), 3.90 (s, 3H, OCH_3_). ^13^C–NMR (101 MHz, CDCl_3_, 25 °C); δ (ppm): 183.43 (C=O), 161.89 (C4′), 145.49 (C7a), 144.27 (C2), 142.60 (COCH=CH), 139.34 (C3a), 130.43 (C1′), 128.44 (C2′, C6′), 127.41 (C6), 127.26 (C5), 125.89 (C4), 124.98 (C7), 123.00 (COCH=CH), 118.80 (C3), 114.49 (C3′, C5′), 55.46 (OCH_3_). HRMS *m/z* calc. for C_18_H_14_O_2_S + (H^+^): 295.0787; found: 295.0776.

##### (*E*)-1-(1-Benzothiophen-2-yl)-3-(*o*-hydroxyphenyl)-2-propen-1-one (**14**)

Following our base-catalyzed aldol condensation general procedure with 2-acetylbenzothiophene (**10**) (250 mg, 1.42 mmol, 1.2 eq) and 2-hydroxybenzaldehyde (145 mg, 1.18 mmol, 1 eq) under reflux for 14.5 h, compound **14** was obtained as a yellow solid after flash chromatography (18% EtOAc/Hex), yield = 39%, Rf = 0.57 (30% EtOAc/Hex), m.p. = 178–180 °C. ^1^H-NMR (400 MHz, DMSO-d6, 25 °C); δ (ppm): 10.34 (s, 1H, OH—D_2_O exchange), 8.67 (s, 1H, H3), 8.13–8.09 (d, 1H, J = 16 Hz, CH=CHC), 8.07–8.05 (d, 2H, J = 8 Hz, H4, H7), 7.98–7.94 (d, 1H, J = 16 Hz, COCH=CH), 7.95–7.93 (m, 1H, H5′), 7.58–7.48 (m, 2H, H5, H6), 7.33–7.29 (m, 1H, H3′), 6.98–6.90 (m, 2H, H4′, H6′). ^13^C-NMR (101 MHz, DMSO-d6, 25 °C); δ (ppm): 183.73 (C=O), 157.88 (C2′), 145.75 (C7a), 142.21(C2), 139.87 (COCH=CH), 139.32 (C3a), 132.84 (C4′), 130.87 (C6′), 129.02 (C6), 128.14 (C5), 126.81 (C4), 125.74 (C7), 123.62 (COCH=CH), 121.64 (C5′), 120.58 (C3), 119.90 (C1′), 116.78 (C3′). HRMS *m/z* calc. for C_17_H_12_O_2_S + (H^+^): 281.0631; found: 281.062.

#### 3.1.4. General Procedure of Hydroxyurea Analogs by Peptide Coupling

Et_3_N (TEA) (1 eq) was added dropwise to a stirred solution of hydroxyl urea (**32**) (1 eq) and the appropriate carboxylic acid (1 eq) in 5 mL of DMF at 0 °C. BOP (1 eq) dissolved in 10 mL of CH_2_Cl_2_ was then added dropwise to the mixture under argon atmosphere at 0 °C. The mixture was stirred for 30 min at 0 °C and at room temperature for 2 h. Water (50 mL) was added followed by extraction with EtOAc (3 × 25 mL). The combined organic fractions were washed with brine, dried over MgSO_4_, decolorized with activated charcoal, filtered, and concentrated under vacuum. The resulting oil was purified by flash chromatography or filtered through celite if the product crystallizes during concentration.

##### 1-Hydroxy-1-((*E*)-3-phenylacryloyl)urea (**33**)

Following our general procedure for the peptide coupling of hydroxyl urea (**32**) (183 mg, 2.4 mmol, 2 eq) with 3,4-dimethoxycinnamic acid (250 mg, 1.2 mmol, 1 eq), TEA (334 μL, 2.4 mmol, 2 eq), and BOP (530 mg, 1.2 mmol, 1 eq), compound **33** was obtained as a white solid after filtering through celite, yield = 40%, Rf = 0.29 (50% EtOAc/Hex), m.p. = 130–132 °C, ^1^H-NMR (400 MHz, DMSO-d6, 25 °C); δ (ppm): 9.66 (s, 1H, OH-D_2_O exchange), 7.80–7.76 (d, 1H, J = 16 Hz, CCH=CH), 7.78–7.75 (m, 2H, H2, H6), 7.47–7.45 (m, 3H, H3, H4, H5), 6.76–6.72 (d, 1H, J = 16 Hz, CH=CHCO), 6.54 (s, 2H, NH_2_–D_2_O exchange). ^13^C-NMR (101 MHz, DMSO-d6, 25 °C); δ (ppm): 166.19 (C=O), 159.91 (NCON), 146.26 (CCH=CH), 134.36 (C1), 131.30 (C3, C5), 129.47 (C2, C6), 128.97 (C4), 115.87 (CH=CHCO). HR-MS *m/z* calc. for C_10_H_10_N_2_O_3_ + (Na^+^): 229.0584; found: 229.0573.

##### 1-Hydroxy-1-((*E*)-3-(3-hydroxy-4-methoxyphenyl)acryloyl)urea (**34**)

Following our general procedure for the peptide coupling of hydroxyl urea (**32**) (195 mg, 2.57 mmol, 1 eq) with 3-hydroxy-4-methoxycinnamic acid (500 mg, 2.57 mmol, 1 eq), TEA (537 μL, 3.85 mmol, 1.5 eq), and BOP (1136 mg, 2.57 mmol, 1 eq), compound **34** was obtained as a light yellow solid after filtering through celite, yield = 29%, Rf = 0.45 (80% EtOAc/Hex), m.p. = 170–172 °C. ^1^H-NMR (400 MHz, DMSO-d6, 25 °C); δ (ppm): 9.60 (s, 1H, OH-D_2_O exchange), 9.25 (s, 1H, OH-D_2_O exchange), 7.65–7.61 (d, 1H, J = 16 Hz, CH=CH), 7.19–7.15 (m, 2H, H_ar_), 7.00–6.98 (d, 1H, J = 8 Hz, H_ar_), 6.52 (s, 2H, NH_2_), 6.46–6.42 (d, 1H, J = 16 Hz, CH=CH), 3.83 (s, 3H, OCH_3_). ^13^C-NMR (101 MHz, DMSO-d6, 25 °C); δ (ppm): 166.44 (C=O), 159.99 (NCON), 150.87 (C4), 147.18 (C3), 146.60 (CCH=CH), 127.21 (C1), 122.13 (C6), 114.69 (CCH=CH), 112.64 (C5), 112.42 (C2), 56.11 (OCH_3_). HRMS *m/z* calc. for C_11_H_12_N_2_O_5_ + (Na^+^): 275.0644; found: 275.0640.

#### 3.1.5. General Procedure of Hydroxyurea Analogs Synthesis with Acyl Chlorides 

To a solution of hydroxyurea (**32**) in dry DMF under argon was added 4-dimethylaminopyridine (DMAP) (1 eq) followed by the appropriate acyl chloride (1 eq) which can be obtained after reflux of the corresponding carboxylic acid with thionyl chloride catalyzed by DMF. The mixture was stirred at −40 °C (acetonitrile/dry ice bath) for 30 min and was then evaporated. The mixture was then diluted with water (50 mL), extracted with ethyl acetate (3 × 25 mL), washed with brine solution (2 × 25 mL), dried over MgSO_4_, and concentrated on a rotary evaporator. The residue was purified by column chromatography eluting with hexane to gradually increase the polarity of ethyl acetate.

##### 1-Hydroxy-1-(benzoyl)urea (**38**)

Following our general procedure for hydroxyurea analogs synthesis with acyl chlorides, hydroxyurea (**32**) (250 mg, 3.27 mmol), DMAP (400 mg, 3.27 mmol), and benzoic acid (400 mg, 3.27 mmol), compound **38** was obtained as a yellow solid after flash chromatography, yield = 41%, Rf = 0.25 (50% EtOAc/Hex), m.p.=127–129 °C.^1^H-NMR (400 MHz, acetone-d6, 25 °C); δ (ppm): 9.28 (s, 1H, OH-D_2_O exchange), 8.12–8.10 (m, 2H, H2, H6), 7.74–7.70 (t, 1H, J = 8 Hz, H4), 7.60–7.56 (m, 2H, J = 8 Hz, H3, H5), 6.39 (s, 2H, NH_2_–D_2_O exchange). ^13^C-NMR (101 MHz, acetone-d6, 25 °C); δ (ppm): 165.34 (CO), 159.59 (NCON), 133.88 (C1), 129.62 (C3, C5), 128.74 (C4), 127.84 (C2, C6). LC-MS *m/z* calc. for C_8_H_8_N_2_O_3_ + (H^+^): 181.0608; found: 181.0596.

##### 1-Hydroxy-1-(2-phenylacetyl)urea (**39**)

Following our general procedure for hydroxyurea analogs synthesis with acyl chlorides, hydroxyurea (**32**) (245 mg, 3.23 mmol), DMAP (394 mg, 3.23 mmol), and phenylacetyl chloride (500 mg, 3.23 mmol), compound **39** was obtained as a white solid after flash chromatography, yield = 65%, Rf = 0.29 (50% EtOAc/Hex), m.p. = 133–135 °C. ^1^H-NMR (400 MHz, DMSO-d6, 25 °C); δ (ppm): 9.60 (s, 1H, OH-D_2_O exchange), 7.37–7.26 (m, 5H, H2, H3, H4, H5, H6), 6.56 (s, 2H, NH_2_–D_2_O exchange), 3.82 (s, 2H, COCH_2_). ^13^C-NMR (101 MHz, DMSO-d6, 25 °C); δ (ppm): 171.03 (CO), 159.93 (NCON), 134.11 (C1), 129.95 (C2, C6), 128.86 (C3, C5), 127.46 (C4), 38.46 (CH_2_).

Details of the synthesis and characterization of compounds **6–9**, **12**, **15–31**, **35–37**, and **40** are in the Supplementary Materials. NMR (^1^H and ^13^C) and HRMS spectra of all compounds are in the Supplementary Materials.

### 3.2. 5-LO Product Biosynthesis Assays in HEK293 Cells

HEK293 cells were stably co-transfected with a pcDNA3.1 vector expressing 5-LO and a pBUDCE4.1 vector expressing 5-LO activating protein (FLAP) as previously reported [29]. This represents a controlled model for the evaluation of 5-LO inhibition in a cellular system. For cell stimulation of 5-LO products, transfected HEK293 cells were collected following trypsinization, washed and the cell pellet was resuspended in Hank’s balanced salt solution (HBSS) (Lonza, Walkerville, MD) containing 1.6 mM CaCl_2_ at a concentration of 5 × 10^5^ cells/mL. Cells were pre-incubated with each compound dissolved in DMSO at the indicated concentration for 5 min at 37 °C. Cells were then stimulated for 15 min at 37 °C with the addition of 10 µM calcium ionophore A23187 (Sigma-Aldrich, Oakville, ON, Canada) and 10 µM arachidonic acid (Cayman Chemical, Ann Arbor, MI). Stimulation was stopped and processed for RP-HPLC analysis as described previously [29,36].

### 3.3. 5-LO Products Biosynthesis Assays in Human PMNL Cells

Human PMNL were prepared from peripheral blood of healthy consenting volunteers as described [17]. Human PMNL are an important source of 5-LO metabolites and are relevant primary inflammatory cells for the evaluation of the best compounds identified during the screening process. PMNL were suspended in HBSS containing 1.6 mM CaCl_2_ (10^7^ cells/mL) and pre-incubated with compounds dissolved in DMSO for 5 min at 37 °C in the presence of 0.4 U/mL of adenosine deaminase (Sigma-Aldrich, Oakville, On, Canada). Cells were then stimulated for 15 min at 37 °C with 1 µM thapsigargin (Sigma-Aldrich) [21]. Reactions were stopped by the addition of 0.5 mL of cold MeOH:CH_3_CN (1:1) and 50 ng of PGB_2_ as internal standard and samples were stored at −20 °C until processing for RP-HPLC analysis as indicated above.

### 3.4. Free Radical Scavenging Activity Assay

The free radical scavenging activity of test compounds was measured as previously described using 2,2-diphenyl-1-picrylhydrazyl (DPPH) as a stable radical [21] with slight modifications. DPPH (200 µL) in ethanol (250 µM) was mixed with 200 µL of the test compounds (1, 5, 10, 25, 50, 75, and 100 µg/mL). Each mixture was then shaken vigorously and held in the dark for 30 min at room temperature. The absorbance of DPPH at 517 nm was then measured. The radical scavenging activity was expressed in terms of % inhibition of DPPH absorbance:

% Inhibition = (A control − A test)/A control) × 100

where A control is the absorbance of the control (DPPH solution without test compounds) and A test is the absorbance of the test sample (DPPH solution plus compounds). Ascorbic acid was used as a positive control. Data were expressed as mean *±* SEM of three independent experiments, each performed in triplicate. IC_50_ values were calculated from a sigmoidal concentration-response curve-fitting model with a variable slope on GraphPad Prism software.

### 3.5. Molecular Docking

Docking simulations were performed with AutoDock Vina 1.1.2 [37] on 5-LO (PDB ID: 3O8Y [30]) according to the reported procedure [32]. The AutoDock Vina parameters were set as follows; box size: 30 × 20 × 30 Å, the center of box: x = 4.049, y = 21.347, z = −0.284, the exhaustiveness: 8, and the other parameters were left unchanged. The structures of all the compounds were optimized using the MMFF94 force field. The calculated geometries were ranked in terms of free energy of binding and the best pose was selected for further analysis. Molecular visualization was performed by with Maestro 11.7 [38] and LigPlot^+^ 2.1 [39].

### 3.6. Statistical Analysis

Statistical analyses and graph design were performed using GraphPad Prism 5 software (GraphPad Software, San Diego, CA, USA).

## 4. Conclusions

Thirty-five zileuton-hydroxycinnamic acid hybrids were synthesized and evaluated for their 5-LO inhibition activities in stimulated HEK293 cells. To further probe the inhibitory activity of compounds that approached the inhibitory activity of zileuton (**1**) and SAPE (**2**), eight compounds were selected for further screening in PMNL. Compounds bearing zileuton’s (**1**) benzo[b]thiophene subunit combined with a phenolic acid moiety through a α,β-unsaturated ketone had interesting results. The number and position of the oxygen functions (hydroxy/methoxy groups) seem to be critical for inhibition of 5-LO product biosynthesis. Compound **28**, having zileuton’s (**1**) benzo[b]thiophene and SAPE’s (**2**) α,β-unsaturated 3,5-dimethoxy-4-hydroxyphenolic acid moieties, was three times as active as Zileuton (**1**) for the inhibition of 5-LO in PMNL. Even with a low antiradical potency, compound **28** was found to be the best 5-LO inhibitor.

The 3,5-dimethoxy-4-hydroxy tri-substitution seems to be the most favorable for good inhibition. Indeed, neither the 2,6-dimethoxy-4-hydroxy or the 3,4-dimethoxy-5-hydroxy substitution appears to be as effective. Compounds **30** and **31** having these substitution patterns were less active than compound **28**. Unlike zileuton (**1**), compound **28** forms π–π interactions with both Phe177 and Phe421 as predicted when docked into 5-LO. Such interactions, in particular with Phe177, may explain the activity of this molecule compared to zileuton (**1**) or SAPE (**2**). Further investigations will be necessary to elucidate the mode of action and confirm the implication of this interaction.

## Figures and Tables

**Table 1:** Free radical scavenging activity.

Compounds	IC_50_ (µM) [SEM]
**14**	31.8 [0.016]
**16**	68.9 [0.015]
**17**	64.7 [0.013]
**18**	68.8 [0.022]
**27**	6.9 [0.023]
**28**	48.9 [0.025]
**31**	6.6 [0.030]
**37**	13.3 [0.021]
Zileuton (**1**)	>100
SAPE (**2**)	25.4 [0.7]
Vitamin C	21.8 [0.023]

**Table 2:** Molecular modeling results predicting affinity for the active site, π–π interactions, and hydrogen bond lengths.

Compound	Affinity (kcal/mol)	π–π Interactions	H-Bond	Distance (Å)
(*R*)-Zileuton (**1**)	−6.6	Phe421	Leu420 × 2, Asn425	2.50, 3.15, 3.29
(*S*)-Zileuton (**1**)	−6.5	-	Leu420 × 2, Ala424, Phe421	2.81, 3.09, 3.34, 2.97
SAPE (**2**)	−8.7	His372	-	-
**28**	−8.4	Phe177, Phe421	-	-
**31**	−8.8	His372, His367	-	-
**37**	−7.6	-	Tyr181, Gln363, His367	3.09, 3.09 3.30, 3.34

**Table 3:** Absorption, distribution, metabolism, and excretion (ADME) profile of molecules of interest.

	Physicochemical Properties				Lipophilicity		Pharmacokinetics	
	MW (g/mol)	ROTB (n)	HBA (n)	HBD (n)	TPSA (Å)	CLogP_o/w_	GIA	BBBP
Rule	<500	≤10	<10	<5	≤140	<5	-	-
Zileuton (**1**)	236.29	3	2	2	94.80	1.81	High	No
SAPE (**2**)	328.36	8	5	1	64.99	3.35	High	Yes
**28**	340.39	5	4	1	84.00	3.99	High	No
**37**	282.25	6	6	3	122.32	0.47	High	No

Abbreviations: BBBP, blood–brain barrier permeation; CLog P_o/w_, logarithm of compound partition coefficient between *n*-octanol and water; GIA, gastrointestinal absorption; HBA, hydrogen bonds acceptors; HBD, hydrogen bond donors; MW, molecular weight; n, number; ROTB, rotatable bonds; TPSA, topological polar surface area.

## Supplementary Materials

The following are available online: Details of the synthesis and characterization of compounds **6–9**, **12**, **15–31**, **35–37**, and **40**. NMR (^1^H and ^13^C) and HRMS spectra of all compounds.
