# Supplementary material for: New Zileuton-Hydroxycinnamic Acid Hybrids: Synthesis and Structure-Activity Relationship towards 5-Lipoxygenase Inhibition
Source: Molecules. 2020 Oct 14;25(20):4686. doi: 10.3390/molecules25204686 (PMC7587396; doi:10.3390/molecules25204686)
Supplement: Supplementary file 1 [file molecules-25-04686-s001.pdf]

Supporting Information

To accompany

New Zileuton-Hydroxycinnamic Acid Hybrids: Synthesis and Structure-Activity Relationship towards 5-Lipoxygenase Inhibition

Audrey Isabel Chiasson, Samuel Robichaud, Fanta J. Ndongou Moutombi, Mathieu P.A. Hébert, Maroua Mbarik, Marc E. Surette and Mohamed Touaibia *

Department of Chemistry and Biochemistry, Université de Moncton, Moncton, NB E1A 3E9, Canada

*Corresponding author. E-mail address: mohamed.touaibia@umoncton.ca

3-Phenylpropyl 1-benzothiophene-2-carboxylate (6)

Following our general procedure esterification with thianaphtene-2-carboxylic acid (**3**) (500 mg, 2.81 mmol, 1 eq), Na_2CO_3 (356 mg, 3.36 mmol, 1.2 eq), and 1-bromo-3-phenylpropane (0.51 mL, 3.36 mmol, 1.2 eq) compound **6** was obtained as a yellow oil after flash chromatography (8% EtOAc/Hex), yield = 20%, Rf = 0.46 (10% EtOAc/Hex). ^{1}H-NMR (400 MHz, $CDCl_3$, 25°C); δ (ppm): 8.07 (s, 1H, H3), 7.92-7.88 (t, 2H, J = 7.0 Hz, H4, H7), 7.51-7.22 (m, 7H, H5, H6, H2′, H3′, H4′, H5′, H6′), 4.41-4.38 (t, 2H, J = 7.0 Hz, $OC\underline{H_2}CH_2CH_2C$), 2.85-2.82 (t, 2H, J =7.0 Hz, $CH_2CH_2C\underline{H_2}C$), 2.19-2.12 (quint., 2H, J = 7.0 Hz, $CH_2C\underline{H_2}CH_2C$). ^{13}C-NMR (101 MHz, $CDCl_3$, 25°C); δ (ppm): 162.81 (C=O), 142.24 (C7a), 141.06 (C3a), 138.74 (C2), 133.70 (C1′), 130.48 (C3′, C5′), 128.51 (C2′, C6′), 128.48 (C4′), 126.93 (C6), 126.09 (C5), 125.55 (C7), 124.91 (C4), 122.77 (C2), 64.78 ($O\underline{C}H_2CH_2CH_2C$), 32.19 ($OCH_2CH_2\underline{C}H_2C$), 30.24 ($OCH_2\underline{C}H_2CH_2C$). HRMS *m*/*z* calc. for $C_{18}H_{16}O_2S$ + (H^+): 297.0944; found: 297.0945.

4-Phenylbutyl 1-benzothiophene-2-carboxylate (7)

Following our general procedure esterification with thianaphtene-2-carboxylic acid (**3**) (250 mg, 1.40 mmol, 1 eq), Na_2CO_3 (178 mg, 1.68 mmol, 1.2 eq), and 1-bromo-4-phenylbutane (358 mg, 1.68 mmol, 1.2 eq) compound **7** was obtained as a yellow solid after flash chromatography (8% EtOAc/Hex), yield = 62%, Rf = 0,61 (10% EtOAc/Hex), m.p.= 56-57°C. ^{1}H-NMR (400 MHz, $CDCl_3$, 25°C); δ (ppm): 8.08 (s, 1H, H3), 7.91-7.88 (t, 2H, J = 7.0 Hz, H4, H7), 7.50-7.20 (m, 7H, H5, H6, H2′, H3′, H4′, H5′, H6′), 4.41-4.38 (t, 2H, J = 6.0 Hz, $OC\underline{H_2}CH_2CH_2CH_2$), 2.75-2.71 (t, 2H, J =7.0 Hz, $CH_2CH_2CH_2C\underline{H_2}C$), 1.87-1.82 (m, 4H, $CH_2C\underline{H_2}C\underline{H_2}CH_2C$). ^{13}C-NMR (101 MHz, $CDCl_3$, 25°C); δ (ppm) : 162.87 (C=O), 142.22 (C7a), 141.98 (C3a), 138.74 (C2), 133.78 (C1′), 130.42 (C3′, C5′), 128.43 (C2′, C6′), 128.39 (C4′), 126.90 (C6), 125.89 (C5), 125.53 (C4), 124.89 (C7), 122.76 (C3), 65.43 ($\underline{C}H_2CH_2CH_2CH_2C$), 35.46 ($CH_2CH_2CH_2\underline{C}H_2C$), 28.27 ($CH_2\underline{C}H_2CH_2CH_2C$), 27.74 ($CH_2CH_2\underline{C}H_2CH_2C$). HRMS *m*/*z* calc. for $C_{19}H_{18}O_2S$ + (H^+): 311.1100; found: 311.1098.

2-(*p*-Methoxyphenyl)ethyl 1-benzothiophene-2-carboxylate (8)

Following our general procedure esterification with thianaphtene-2-carboxylic acid (**3**) (250 mg, 1.40 mmol, 1 eq), Na_2CO_3 (178 mg, 1.68 mmol, 1.2 eq), and 4-methoxyphenethylbromide (361 mg, 1.68 mmol, 1.2 eq) compound **8** was obtained as a pink solid after flash chromatography (10% EtOAc/Hex), yield = 76%, Rf = 0,48 (10% EtOAc/Hex), m.p.= 98-99°C. ^{1}H-NMR (400 MHz, $CDCl_3$, 25°C); δ (ppm): 8.07 (s, 1H, H3), 7.91-7.89 (m, 2H, H4, H7), 7.50-7.41 (m, 2H, H5, H6), 7.28-7.24 (m, 2H, H2′, H6′), 6.91-6.89 (d, 2H, J = 8.0 Hz, H3′, H5′) 4.56-4.52 (t, 2H, J = 7.0 Hz, $OC\underline{H_2}CH_2C$), 3.82 (s, 3H, $OC\underline{H_3}$), 3.08-3.05 (t, 2H, J = 7.0 Hz, $OCH_2C\underline{H_2}C$).^{13}C-NMR (101 MHz, $CDCl_3$, 25°C); δ (ppm): 162.72 (C=O), 158.42 (C4′), 142.25 (C7a), 138.72 (C3a), 133.64 (C2), 130.53 (C1′), 130.01 (C2′, C6′), 129.67 (C6), 126.94 (C5), 125.56 (C4), 124.90 (C7), 122.77 (C3), 114.01 (C3′, C5′), 66.26 ($O\underline{C}H_2CH_2C$), 55.27 (OCH_3), 34.35 ($OCH_2\underline{C}H_2C$). HRMS *m*/*z* calc. for $C_{18}H_{16}O_3S$ + (Na^+): 335.0712; found: 335.0705.

2-(*p*-Hydroxyphenyl)ethyl 1-benzothiophene-2-carboxylate (9)

Following our general procedure esterification with thianaphtene-2-carboxylic acid (**3**) (184 mg, 1.00 mmol, 1 eq),

Na_2CO_3 (127 mg, 1.20 mmol, 1.2 eq), and 4-hydroxyphenethylbromide (250 mg, 1.2 mmol, 1.2 eq) compound **9** was obtained as an orange solid after flash chromatography (15% EtOAc/Hex), yield = 57%, Rf = 0,40 (10% EtOAc/Hex), m.p.= 142-143°C. ^{1}H-NMR (400 MHz, $CDCl_3$, 25°C); δ (ppm) : 8.07 (s, 1H, H3), 7.91-7.89 (m, 2H, H4, H7), 7.50-7.41 (m, 2H, H5, H6), 7.20-7.18 (d, 2H, J=8.0 Hz, H2′, H6′), 6.84-6.80 (m, 2H, H3′, H5′), 4.55-4.51 (t, 2H, J = 7.0 Hz, $OC\underline{H_2}CH_2$), 3.06-3.03 (t, 2H, $CH_2C\underline{H_2}C$). ^{13}C-NMR (101 MHz, $CDCl_3$, 25°C); δ (ppm): 162.81 (C=O), 154.45 (C4′), 142.26 (C7a), 138.70 (C3a), 133.55 (C2), 133.55 (C1′), 130.59 (C2′, C6′), 130.20 (C6), 129.69 (C5), 126.96 (C4), 125.57 (C7), 124.91 (C3), 115.46 (C3′, C5′), 66.28 ($\underline{C}H_2CH_2C$), 34.34 ($CH_2\underline{C}H_2C$). HRMS *m*/*z* calc. for $C_{17}H_{14}O_3S$ + (NH_4^+): 316.4; found: 316.1006.

(*E*)-1-(1-Benzothiophen-2-yl)-3-(*m*-methoxyphenyl)-2-propen-1-one (12)

Following our general procedure for the aldol condensation in the presence of sodium ethoxide with 2-acetylbenzothiophene (**10**) (250 mg, 1.42 mmol, 1 eq), sodium ethoxide (97 mg, 1.42 mg, 1 eq) and 3-methoxybenzaldehyde (232 mg, 1.70 mmol, 1.2 eq) compound **12** was obtained as a yellow solid after flash chromatography (8% EtOAc), yield = 17%, Rf = 0.27 (10% EtOAc), m.p. = 124-125˚C. ^{1}H-NMR (400 MHz, $CDCl_3$, 25°C); δ (ppm): 8.13 (s, 1H, H3), 7.96-7.92 (m, 2H, H4, H7), 7.91-7.87 (d, 1H, J=16 Hz, CH=C$\underline{H}$C), 7.57-7.53 (d, 1H, J=16 Hz, COC$\underline{H}$=CH), 7.51-7.28 (m, 4H, H5, H6, H2′, H6′), 7.21 (s, 1H, H5′), 7.03-7.00 (m, 1H, H4′), 3.90 (s, 3H, OCH_3). ^{13}C-NMR (101 MHz, $CDCl_3$, 25°C); δ (ppm): 183.42 (C=O), 160.00 (C3′), 145.17 (C7a), 144.34 (C2), 142.73 (COCH=$\underline{C}$H), 139.28 (C3a), 136.04 (C1′), 130.02 (C5′), 128.92 (C6), 127.46 (C5), 126.00 (C4), 125.06 (C7), 123.03 (CO$\underline{C}$H=CH), 121.43 (C3), 121.22 (C6′), 116.49 (C4′), 113.65 (C2′), 55.41 (OCH_3). HRMS *m*/*z* calc. for $C_{18}H_{14}O_2S$ + (H^+): 295.0787; found: 295.078.

(*E*)-1-(1-Benzothiophen-2-yl)-3-(*m*-hydroxyphenyl)-2-propen-1-one (15)

Following our base-catalyzed aldol condensation general procedure with 2-acetylbenzothiophene (**10**) (250 mg, 1.42 mmol, 1.2 eq) and 3-hydroxybenzaldehyde (145 mg, 1.18 mmol, 1 eq) under reflux for 20 h, compound **15** was obtained as a yellow solid after flash chromatography (18% EtOAc/Hex), yield = 18 %, Rf = 0.60 (30% EtOAc/Hex), m.p. =197-199˚C. ^{1}H-NMR (400 MHz, DMSO-d6, 25°C); δ (ppm): 9.67 (s, 1H, OH - D_2O exchange), 8.78 (s, 1H, H3), 8.10-8.04 (m, 2H, H4, H7), 7.98-7.94 (d, 1H, J=16 Hz, CH=C$\underline{H}$C), 7.72-7.68 (d, 1H, J=16 Hz, COC$\underline{H}$=CH), 7.58-7.49 (m, 2H, H2′, H5′), 7.37-7.27 (m, 3H, H5, H6, H6′), 6.93-6.90 (m, 1H, H4′). ^{13}C-NMR (101 MHz, DMSO-d6, 25°C); δ (ppm): 183.62 (C=O), 158.25 (C3′), 145.48 (C7a), 144.25 (C2), 142.32 (COCH=$\underline{C}$H), 139.86 (C3a), 136.24 (C1′), 131.58 (C5′), 130.38 (C6), 128.28 (C5), 126.86 (C4), 125.79 (C7), 123.65 (CO$\underline{C}$H=CH), 121.79 (C3), 120.51 (C6′), 118.51 (C4′), 115.89 (C2′). HRMS *m*/*z* calc. for $C_{17}H_{12}O_2S$ + (H^+): 281.0631; found: 281.062.

(*E*)-1-(1-Benzothiophen-2-yl)-3-(*p*-hydroxyphenyl)-2-propen-1-one (16)

Following our base-catalyzed aldol condensation general procedure with 2-Acetylbenzothiophene (**10**) (250 mg, 1.42 mmol, 1.2 eq) and 4-hydroxybenzaldehyde (144 mg, 1.18 mmol, 1 eq) under reflux for 6h, compound **16** was obtained as a yellow solid after flash chromatography (40% EtOAc/Hex), yield = 28%, Rf = 0,18 (50% EtOAc/Hex),

m.p.= 179-180°C. ^{1}H-NMR (400 MHz, $CDCl_3$, 25°C); δ (ppm) : 8.13 (s, 1H, H3), 7.96-7.92 (m, 2H, H4, H7), 7.91-7.87 (d, 1H, J=16 Hz, CH=CHC), 7.64-7.62 (d, 2H, J=8.6 Hz, H5, H6), 7.52-7.45 (m, 2H, H2′, H6′), 7.47-7.43 (d, 1H, J=16 Hz, COCH=CH), 6.94-6.92 (d, 2H, J=8.6 Hz, H3′, H5′), 5.39 (s, 1H, OH). ^{13}C-NMR (101 MHz, $CDCl_3$, 25°C); δ (ppm): 183.58 (C=O), 158.07 (C4′), 145.39 (C7a), 144.21 (C2), 142.63 (COCH=CH), 139.32 (C3a), 130.65 (C1′), 128.65 (C2′, C6′), 127.64 (C6), 127.31 (C5), 125.90 (C4, C7)), 123.01 (COCH=CH), 118.90 (C3), 116.05 (C3′, C5′). HRMS *m/z* calc. for $C_{17}H_{12}O_2S$ + (H^+): 281.0631; found: 281.0628.

(*E*)-1-(1-Benzothiophen-2-yl)-3-(3-hydroxy-4-methoxyphenyl)-2-propen-1-one (17)

Following our base-catalyzed aldol condensation general procedure with 2-acetylbenzothiophene (**10**) (250 mg, 1.42 mmol, 1.2 eq) and 3-hydroxy-4-methoxybenzaldehyde (180 mg, 1.18 mmol, 1 eq) under reflux for 10h, compound **17** was obtained as a yellow solid after flash chromatography (28% EtOAc/Hex), yield = 47%, Rf = 0.50 (40% EtOAc/Hex), m.p. = 158-159°C. ^{1}H-NMR (400 MHz, $CDCl_3$, 25°C); δ (ppm): 8.11 (s, 1H, H3), 7.96-7.91 (m, 2H, H4, H7), 7.86-7.82 (d, 1H, J = 15 Hz, CH=CHC), 7.51-7.42 (m, 2H, H_{ar}), 7.45-7.41 (d, 1H, J=15 Hz, COCH=CH), 7.36-7.35 (d, 1H, J = 2.0 Hz H6′), 7.29 (s, 1H, H5), 7.21-7.18 (m, 1H, H6), 6.93-6.90 (d, 1H, J = 8.0 Hz, H2′), 5.73 (s, 1H, OH), 3.98 (s, 3H, OCH_3). ^{13}C-NMR (101 MHz, $CDCl_3$, 25°C); δ (ppm): 183.39 (C=O), 149.03 (C7a), 145.98 (C2), 145.45 (C3′), 144.33 (C4′), 142.62 (COCH=CH), 139.34 (C3a), 128.56 (C1′), 128.34 (C6), 127.29 (C5), 125.93 (C4), 124.98 (C7), 123.11 (COCH=CH), 122.99 (C3), 119.34 (C2′), 112.97 (C3′), 110.61 (C6′), 56.07 (OCH_3). HRMS *m/z* calc. for $C_{18}H_{14}O_3S$ + (H^+): 311.0736; found: 311.0729.

(*E*)-1-(1-Benzothiophen-2-yl)-3-(4-hydroxy-3-methoxyphenyl)-2-propen-1-one (18)

Following our base-catalyzed aldol condensation general procedure with 2-acetylbenzothiophene (**10**) (250 mg, 1.42 mmol, 1.2 eq) and 4-hydroxy-3-methoxybenzaldehyde (180 mg, 1.18 mmol, 1 eq) under reflux for 9h, compound **18** was obtained as a yellow solid after flash chromatography (30% EtOAc/Hex), yield = 29%, Rf = 0,29 (40% EtOAc/Hex), m.p. = 113-114°C. ^{1}H-NMR (400 MHz, $CDCl_3$, 25°C); δ (ppm): 8.13 (s, 1H, H3), 7.96-7.91 (t, 2H, J=8.0 Hz H4, H7), 7.88-7.85 (d, 1H, J=15 Hz, CH=CHC), 7.52-7.42 (m, 2H, H5, H6), 7.43-7.40 (d, 1H, J=15 Hz, COCH=CH), 7.31-7.30 (d, 1H, J=2.0 Hz H6′), 7.18 (d, 1H, J=2.0 Hz, H2′), 7.01-6.99 (d, 1H, J=8.0 Hz, H5′), 6.0 (s, 1H, OH), 4.02 (s, 3H, OCH_3). ^{13}C-NMR (101 MHz, $CDCl_3$, 25°C); δ (ppm): 183.39 (C=O), 148.52 (C7a), 146.84 (C3′), 145.44 (C2), 144.76 (C4′), 142.60 (COCH=CH), 139.32 (C3a), 128.45 (C1′), 127.28 (C6), 125.87 (C5), 125.00 (C4), 123.50 (C7), 123.01 (COCH=CH), 118.76 (C3, C6′), 114.95 (C5′), 110.29 (C6′). HRMS *m/z* calc. for $C_{18}H_{14}O_3S$ + (H^+): 311.0736; found: 311.0736.

(*E*)-1-(1-Benzothiophen-2-yl)-3-(3,4-dimethoxyphenyl)-2-propen-1-one (19)

Following our base-catalyzed aldol condensation general procedure with 2-acetylbenzothiophene (**10**) (500 mg, 2.84 mmol, 1.2 eq) and 3,4-dimethoxybenzaldehyde (394 mg, 2.37 mmol, 1 eq) under reflux for 22.5 h, compound **19** was obtained as a yellow solid after flash chromatography (18% EtOAc/Hex), yield = 23 %, Rf = 0.24 (20% EtOAc/Hex), m.p. =127-128˚C. ^{1}H-NMR (400 MHz, $CDCl_3$, 25°C); δ (ppm): 8.13 (s, 1H, H3), 7.96-7.91 (m, 2H, H4,

H7), 7.89-7.86 (d, 1H, J=15 Hz, CH=CHC), 7.52-7.42 (m, 2H, H5, H6), 7.45-7.41 (d, 1H, J=15 Hz, COCH=CH), 7.32-7.28 (m, 1H, H6′), 7.22-7.21 (d, 1H, J=2 Hz, H2′), 6.95-6.93 (d, 1H, J=8 Hz, H5′), 4.00 (s, 3H, OCH_3), 3.97 (s, 3H, OCH_3). ^{13}C-NMR (101 MHz, $CDCl_3$, 25°C); δ (ppm): 183.38 (C=O), 151.68 (C7a), 149.32 (C3′), 145.43 (C4′), 144.60 (C2), 142.61 (COCH=CH), 139.32 (C3a), 138.48 (C1′), 127.65 (C6), 127.30 (C5), 125.88 (C4), 125.00 (C7), 123.41 (COCH=CH), 123.01 (C3), 119.00 (C2′), 111.18 (C3′) , 110.28 (C6′), 56.04 (OCH_3). HRMS *m/z* calc. for $C_{19}H_{16}O_3S$ + (H^+): 325.0893; found: 325.0883.

(*E*)-1-(1-Benzothiophen-2-yl)-3-(3,5-dimethoxyphenyl)-2-propen-1-one (20)

Following our base-catalyzed aldol condensation general procedure with 2-acetylbenzothiophene (**10**) (250 mg, 1.42 mmol, 1.2 eq) and 3,5-dimethoxybenzaldehyde (197 mg, 1.18 mmol, 1 eq) under reflux for 8 h, compound **20** was obtained as a yellow solid after flash chromatography (12% EtOAc/Hex), yield = 15 %, Rf = 0,62 (20% EtOAc/Hex), m.p. =113-114˚C. ^{1}H-NMR (400 MHz, $CDCl_3$, 25°C); δ (ppm): 8.13 (s, 1H, H3), 7.96-7.93 (t, 2H, J=8 Hz, H4, H7), 7.91-7.85 (d, 1H, J=16 Hz, CH=CHC), 7.53-7.49 (d, 1H, J=16 Hz, COCH=CH), 7.53-7.91-7.43 (m, 2H, H5, H6), 6.84-6.83 (d, 2H, J = 2 Hz, H2′, H6′), 6.58-6.57 (t, 1H, J=2 Hz, H4′), 3.88 (s, 6H, OCH_3). ^{13}C-NMR (101 MHz, $CDCl_3$, 25°C); δ (ppm): 183.40 (C=O), 161.12 (C3′, C5′), 145.13 (C7a), 144.43 (C2), 142.73 (COCH=CH), 139.27 (C3a), 136.54 (C1′), 128.94 (C6), 127.47 (C5), 126.00 (C4), 125.06 (C7), 123.02 (COCH=CH), 121.62 (C3), 106.51 (C2′, C6′), 102.94 (C4′), 55.53 (OCH_3). HRMS *m/z* calc. for $C_{19}H_{16}O_3S$ + (H^+): 325.0893; found: 325.0885.

(*E*)-1-(1-Benzothiophen-2-yl)-3-(2,3-dimethoxyphenyl)-2-propen-1-one (21)

Following our base-catalyzed aldol condensation general procedure with 2-acetylbenzothiophene (**10**) (250 mg, 1.42 mmol, 1.2 eq) and 2,3-dimethoxybenzaldehyde (197 mg, 1.18 mmol, 1 eq) under reflux for 14.5 h, compound **21** was obtained as a yellow solid after flash chromatography (10 % EtOAc/Hex), yield = 14 %, Rf = 0.19 (10 % EtOAc/Hex), m.p. =86-87˚C. ^{1}H-NMR (400 MHz, $CDCl_3$, 25°C); δ (ppm): 8.22-8.18 (d, 1H, J = 16 Hz, CH=CHC), 8.11 (s, 1H, H3), 7.95-7.90 (t, 2H, J = 9 Hz, H4, H7), 7.67-7.63 (d, 1H, J = 16 Hz, COCH=CH), 7.51-7.42 (m, 2H, H5, H6), 7.34-7.32 (d, 1H, J = 8 Hz, H6′), 7.16-7.12 (t, 1H, J = 8 Hz, H5′), 7.02-7.00 (d, 1H, J = 8 Hz, H4′), 3.94 (s, 3H, OCH_3), 3.92 (s, 3H, OCH_3). ^{13}C-NMR (101 MHz, $CDCl_3$, 25°C); δ (ppm): 183.79 (C=O), 153.28 (C7a), 149.16 (C3′), 145.34 (C2′), 142.68 (C2), 139.35 (COCH=CH), 139.31 (C3a), 128.87 (C6), 128.84 (C5), 127.37 (C4), 125.98 (C7), 125.01 (COCH=CH), 124.25 (C3), 122.72 (C5′), 119. 82 (C6′), 114.44 (C4′), 61.39 (OCH_3), 55.94 (OCH_3). HRMS *m/z* calc. for $C_{19}H_{16}O_3S$ + (H^+): 325.0893; found: 325.0886.

(*E*)-1-(1-Benzothiophen-2-yl)-3-(2,4-dimethoxyphenyl)-2-propen-1-one (22)

Following our base-catalyzed aldol condensation general procedure with 2-acetylbenzothiophene (**10**) (250 mg, 1.42 mmol, 1.2 eq) and 2,4-dimethoxybenzaldehyde (197 mg, 1.18 mmol, 1 eq) under reflux for 14.5 h, compound **22** was obtained as a yellow solid after flash chromatography (15 % EtOAc/^{1}H-NMR (400 MHz, $CDCl_3$, 25°C); δ (ppm Hex), yield = 45 %, Rf = 0.45 (20% EtOAc/Hex), m.p. = 98-100˚C.): 8.17-8.13 (d, 1H, J = 16 Hz, CH=CHC), 8.08 (s, 1H, H3), 7.94-7.90 (t, 2H, J = 8 Hz, H4, H7), 7.63-7.60 (m, 1H, H5), 7.61-7.57 (d, 1H, J = 16 Hz, CH=CHC), 7.49-

7.41 (m, 1H, H6′), 6.59-6.56 (m, 1H, H5′$_r$), 6.51-6.50 (d, 1H, J = 2 Hz, H3′), 3.95 (s, 3H, $OC\underline{H}_3$), 3.86 (s, 3H, $OC\underline{H}_3$). ^{13}C-NMR (101 MHz, $CDCl_3$, 25°C); δ (ppm): 184.02 (C=O), 163.27 (C4′), 160.65 (C7a), 145.87 (C2′), 142.50 (C2), 140.20 (COCH=$\underline{C}$H), 139.41 (C3a), 131.32 (C6′), 128.14 (C6), 127.05 (C5), 125.81 (C4), 124.86 (C7), 122.96 (COCH=$\underline{C}$H), 119.47 (C3), 116.94 (C1′), 105.53 (C5′), 98.48 (C3′), 55.61 (OCH_3), 55.53 (OCH_3). LC-MS *m/z* calc. for $C_{19}H_{16}O_3S$ + (H^+): 325.0893; found: 325.089.

(*E*)-1-(1-Benzothiophen-2-yl)-3-(2,5-dimethoxyphenyl)-2-propen-1-one (23)

Following our base-catalyzed aldol condensation general procedure with 2-acetylbenzothiophene (250 mg, 1.42 mmol, 1.2 eq) and 2,5-dimethoxybenzaldehyde (197 mg, 1.18 mmol, 1 eq) under reflux for 13 h, compound **23** was obtained as a brown solid after flash chromatography (7-10 % EtOAc/Hex), quantitative yield, Rf = 0.22 (10% EtOAc/Hex), m.p.= 95-97 ˚C. ^{1}H-NMR (400 MHz, $CDCl_3$, 25°C); δ (ppm): 8.17-8.13 (d, 1H, J=16 Hz, CH=C$\underline{H}$C), 8.08 (s, 1H, H3), 7.94-7.90 (t, 2H, J = 8 Hz, H4, H7), 7.63-7.60 (m, 1H, H6′), 7.61-7.57 (d, 1H, J = 16 Hz, COC$\underline{H}$=CH), 7.49-7.41 (m, 2H, H5, H6), 6.59-6.56 (m, 1H, H3′), 6.51-6.50 (d, 1H, J = 2 Hz, H4′), 3.95 (s, 3H, $OC\underline{H}_3$), 3.86 (s, 3H, $OC\underline{H}_3$). ^{13}C-NMR (101 MHz, $CDCl_3$, 25°C); δ (ppm): 183.94 (C=O), 153.56 (C5′), 153.55 (C2′), 145.46 (C7a), 142.64 (C2), 139.85 (COCH=$\underline{C}$H), 139.33 (C3a), 128.67 (C6), 127.27 (C5), 125.93 (C4), 124.96 (C7), 124.29 (CO$\underline{C}$H=CH), 122.99 (C3), 122.22 (C3′), 117.45 (C1′), 114.09 (C4′), 112.52 (C6′), 56.16 (OCH_3), 55.90 (OCH_3). LC-MS *m/z* calc. for $C_{19}H_{16}O_3S$ + (H^+): 325.0893; found: 325.0883.

(*E*)-1-(1-Benzothiophen-2-yl)-3-(2,6-dimethoxyphenyl)-2-propen-1-one (24)

Following our base-catalyzed aldol condensation general procedure with 2-acetylbenzothiophene (**10**) (250 mg, 1.42 mmol, 1.2 eq) and 2,6-dimethoxybenzaldehyde (196 mg, 1.18 mmol, 1 eq), under reflux for 7.5h, compound **24** was obtained as a yellow solid after flash chromatography (18% EtOAc/Hex), yield = 36%, Rf = 0,25 (20% EtOAc/Hex), m.p. = 175-176 ˚C. ^{1}H-NMR (400 MHz, $CDCl_3$, 25°C); δ (ppm): 8.40-8.36 (d, 1H, J=16 Hz, CH=C$\underline{H}$C), 8.07-8.01 (d, 1H, J = 16 Hz, COC$\underline{H}$=CH), 8.05 (s, 1H, H3), 7.95-7.91 (t, 2H, J = 9 Hz, H4, H7), 7.50-7.41 (m, 2H, H5, H6), 7.36-7.32 (t, 1H, J=8 Hz, H4′), 6.38-6.62 (d, 2H, J = 8 Hz, H3′, H5′), 3.98 (s, 6H, $OC\underline{H}_3$). ^{13}C-NMR (101 MHz, $CDCl_3$, 25°C); δ (ppm): 185.11 (C=O), 160.57 (C7a), 146.05 (C2′, C6′), 142.53 (C2), 139.43 (COCH=$\underline{C}$H), 135.48 (C3a), 131.79 (C4′), 128.20 (C6), 126.98 (C5), 125.80 (C4), 124.80 (C7), 124.01 (CO$\underline{C}$H=CH), 122.97 (C3), 112.78 (C1′), 103.79 (C3′, C5′), 55.94 (OCH_3). HRMS *m/z* calc. for $C_{19}H_{16}O_3S$ + (H^+): 325.0893; found: 325.0885.

(*E*)-1-(1-Benzothiophen-2-yl)-3-(3,4,5-trimethoxyphenyl)-2-propen-1-one (26)

Following our base-catalyzed aldol condensation general procedure with 2-acetylbenzothiophene (**10**) (250 mg, 1.42 mmol, 1.2 eq) and 3,4,5-trimethoxybenzaldehyde (232 mg, 1.18 mmol, 1 eq) under reflux for 14 h, compound **26** was obtained as a yellow solid after flash chromatography (18% EtOAc/Hex), yield = 12 %, Rf = 0,20 (20% EtOAc/Hex), m.p. =125-127˚C. ^{1}H-NMR (400 MHz, $CDCl_3$, 25°C); δ (ppm): 8.14 (s, 1H, H3), 7.97-7.92 (t, 2H, J = 9 Hz, H4, H7), 7.86-7.82 (d, 1H, J = 16 Hz, CH=C$\underline{H}$C), 7.53-7.43 (m, 2H, H5, H6) 7.46-7.42 (d, 1H, J = 16 Hz, COC$\underline{H}$=CH), 6.93 (s, 2H, H2′, H6′), 3.97 (s, 6H, O-$C\underline{H}_3$), 3.94 (s, 3H, O-$C\underline{H}_3$). ^{13}C-NMR (101 MHz, $CDCl_3$, 25°C); δ

(ppm): 183.30 (C=O), 153.54 (C7a), 145.22 (C3′), 144.61 (C5′), 142.68 (COCH=CH), 140.72 (C3a), 139.28 (C4′), 130.12 (C1′), 128.73 (C6), 127.42 (C5), 125.93 (C4), 125.06 (C7), 123.03 (COCH=CH), 120.37 (C3), 105.88 (C2′, C6′), 61.04 (OCH_3), 56.30 (OCH_3). HRMS *m/z* calc. for $C_{20}H_{18}O_4S$ + (H^+): 355.0999; found: 355.0989.

(*E*)-1-(1-Benzothiophen-2-yl)-3-(2,4,6-trimethoxyphenyl)-2-propen-1-one (27)

Following our base-catalyzed aldol condensation general procedure with 2-acetylbenzothiophene (**10**) (250 mg, 1.42 mmol, 1.2 eq) and 2,4,6-trimethoxybenzaldehyde (232 mg, 1.18 mmol, 1 eq) under reflux for 7 h, compound **27** was obtained as a yellow solid after flash chromatography (25% EtOAc/Hex), yield = 36 %, Rf = 0.41 (30% EtOAc/Hex), m.p. =145-147˚C. ^{1}H-NMR (400 MHz, $CDCl_3$, 25°C); δ (ppm): 8.38-8.34 (d, 1H, J=16 Hz, CH=CHC), 8.04 (s, 1H, H3), 7.94-7.87 (d, 1H, J=16 Hz, COCH=CH), 7.92 (s, 2H, H4, H7), 7.48-7.40 (m, 2H, H5, H6), 6.17 (s, 2H, H3′, H5′), 3.96 (s, 6H, OCH_3), 3.89 (s, 3H, OCH_3). ^{13}C-NMR (101 MHz, $CDCl_3$, 25°C); δ (ppm): 185.04 (C=O), 163.40 (C4′), 161.94 (C2′, C6′), 146.40 (C7a), 142.40 (C2), 139.49 (COCH=CH), 135.72 (C3a), 127.71 (C6), 126.79 (C5), 125.69 (C4), 124.72 (C7), 122.93 (COCH=CH), 121.04 (C3), 106.47 (C1′), 90.57 (C3′, C5′), 55.88 (OCH_3), 55.44 (OCH_3). HRMS *m/z* calc. for $C_{20}H_{18}O_4S$ + (H^+): 355.0999; found: 355.099.

(*E*)-1-(1-Benzothiophen-2-yl)-3-(4-hydroxy-3,5-dimethoxyphenyl)-2-propen-1-one (28)

Following our base-catalyzed aldol condensation general procedure with 2-acetylbenzothiophene (**10**) (250 mg, 1.42 mmol, 1.2 eq) and 4-hydroxy-3,5-methoxybenzaldehyde (215 mg, 1.18 mmol, 1 eq) under reflux for 10h, compound **28** was obtained as a yellow solid after flash chromatography (30% EtOAc/Hex), yield = 11%, Rf = 0.26 (50% EtOAc/Hex), m.p. = 69-70°C. ^{1}H-NMR (400 MHz, $CDCl_3$, 25°C); δ (ppm): 8.13 (s, 1H, H3), 7.96-7.91 (m, 2H, H4, H7), 7.85-7.81 (d, 1H, J = 15 Hz, CH=CHC), 7.51-7.28 (m, 2H, H5, H6), 7.42-7.38 (d, 1H, J=15 Hz, COCH=CH), 6.94 (s, 2H, H2′, H6′), 5.9 (s, 1H, OH), 4.00 (s, 6H, OCH_3). ^{13}C-NMR (101 MHz, $CDCl_3$, 25°C); δ (ppm): 183.26, (CO) 147.32 (C3′, C5′), 145.39 (C7a), 145.00 (C2), 142.61 (COCH=CH), 139.31 (C3a), 137.79 (C4′), 128.51 (C1′), 127.31 (C6), 126.18 (C5), 125.87 (C4), 125.01 (C7), 123.00 (COCH=CH), 118.99 (C3), 105.74 (C2′, C6′), 56.46 (OCH_3). HRMS *m/z* calc. for $C_{19}H_{16}O_4S$ + (H^+): 341.0842; found: 341.0842.

(*E*)-1-(1-Benzothiophen-2-yl)-3-(4-hydroxy-3,5-dimethylphenyl)-2-propen-1-one (29)

Following our base-catalyzed aldol condensation general procedure with 2-acetylbenzothiophene (**10**) (250 mg, 1.42 mmol, 1.2 eq) and 4-hydroxy-3,5-dimethylbenzaldehyde (178 mg, 1.18 mmol, 1 eq) under reflux for 7.5h, compound **29** was obtained as a yellow solid after flash chromatography (18% EtOAc/Hex), yield = 16%, Rf = 0,51 (30% EtOAc/Hex), m.p. = 178-179˚C. ^{1}H-NMR (400 MHz, $CDCl_3$, 25°C); δ (ppm): 8.12 (s, 1H, H3), 7.95-7.82 (t, 2H, J = 9 Hz, H4, H7), 7.85-7.82 (d, 1H, J = 15 Hz, CH=CHC), 7.51-7.28 (m, 4H, H5, H6, H2′, H6′), 7.45-7.41 (d, 1H, J = 15 Hz, COCH=CH), 5.15 (s, 1H, OH), 2.33 (s, 6H, OCH_3). ^{13}C-NMR (101 MHz, $CDCl_3$, 25°C); δ (ppm): 183.52 (C=O), 155.07 (C4′), 145.60 (C7a), 144.83 (C2), 142.59 (COCH=CH), 139.37 (C3a), 129.57 (C2′, C6′), 128.40 (C1′), 127.22 (C6), 126.87 (C5), 125.86 (C4), 124.96 (C7), 123.73 (COCH=CH), 122.99 (C3), 118.35 (C3′, C5′), 15.91 (CH_3). HRMS *m/z* calc. for $C_{19}H_{16}O_2S$ + (H^+): 309.0944; found: 309.0933.

(*E*)-1-(1-Benzothiophen-2-yl)-3-(4-hydroxy-2,6-dimethoxyphenyl)-2-propen-1-one (30)

Following our base-catalyzed aldol condensation general procedure with 2-acetylbenzothiophene (**10**) (250 mg, 1.42 mmol, 1.2 eq) and 4-hydroxy-2,6-dimethoxybenzaldehyde (215 mg, 1.18 mmol, 1 eq), under reflux for 15 hr, compound **30** was obtained as a yellow solid after flash chromatography (40% EtOAc/Hex), yield = 66%, Rf = 0,38 (60% EtOAc/Hex), m.p. = 195-197 ˚C. ^{1}H-NMR (400 MHz, DMSO-d6 25°C); δ (ppm): 10.40 (s, 1H, OH), 8.35 (s, 1H, H3), 8.17-8.13 (d, 1H, J = 15 Hz, CH=C$\underline{H}$C), 8.09-8.05 (t, 2H, J = 8 Hz, H4, H7), 7.89-7.85 (d, 1H, J = 15 Hz, COC$\underline{H}$=CH), 7.53-7.48 (m, 2H, H5, H6), 6.19 (s, 2H, H3′, H5′), 3.32 (s, 6H, OC$\underline{H}_3$). ^{13}C-NMR (101 MHz, DMSO-d6, 25°C); δ (ppm): 184.14 (C=O), 163.07 (C2′, C6′, C4′), 162.38 (C7a), 146.34 (C2), 141.89 (COCH=$\underline{C}$H), 139.97 (C3a), 135.75 (C6), 129.07 (C5), 127.77 (C4), 126.71 (C7), 125.56 (CO$\underline{C}$H=CH), 118.84 (C3), 104.19 (C1′), 92.71 (C3′, C5′), 56.38 (OCH_3). HRMS *m*/*z* calc. for $C_{19}H_{16}O_4S$ + (H^+): 341.0842; found: 341.0836.

(*E*)-1-(1-Benzothiophen-2-yl)-3-(3-hydroxy-4,5-dimethoxyphenyl)-2-propen-1-one (31)

Following our base-catalyzed aldol condensation general procedure with 2-acetylbenzothiophene (**10**) (250 mg, 1.42 mmol, 1.2 eq) and 5-hydroxy-3,4-dimethoxybenzaldehyde (215 mg, 1.18 mmol, 1 eq) under reflux for 15 h, compound **31** was obtained as a yellow solid after flash chromatography (20 % EtOAc/Hex), yield = 41 %, Rf = 0.29 (30 % EtOAc/Hex), m.p. = 158-160˚C. ^{1}H-NMR (400 MHz, DMSO, 25°C); δ (ppm): 9.37 (s, 1H, OH), 8.73 (s, 1H, H3), 8.10-8.8.05 (m, 2H, H4, H7), 7.90-7.86 (d, 1H, J = 16 Hz, CH=C$\underline{H}$C), 7.67-7.63 (d, 1H, J = 16 Hz, COC$\underline{H}$=CH), 7.58-7.49 (m, 2H, H5, H6), 7.14-7.13 (d, 1H, J = 2 Hz, H2′), 7.01-7.00 (d, 1H, J = 2 Hz, H6′), 3.89 (s, 3H, OC$\underline{H}_3$), 3.75 (s, 3H, OC$\underline{H}_3$). ^{13}C-NMR (101 MHz, DMSO, 25°C); δ (ppm): 183.50 (C=O), 153.88 (C7a), 151.15 (C5′), 145.70 (C3′), 144.51 (C2), 142.29 (COCH=$\underline{C}$H), 139.84 (C3′), 139.46 (C3a), 131.20 (C1′), 130.36 (C6), 128.21 (C5), 126.77 (C4), 125.79 (C7), 123.65 (CO$\underline{C}$H=CH), 120.78 (C3), 111.52 (C2′), 104.60 (C6′), 60.58 (OCH3), 56.52 (OCH3). HRMS *m*/*z* calc. for $C_{19}H_{16}O_4S$ + (H^+): 341.0842; found: 341.0835.

1-Hydroxy-1-((E)-3-(4-hydroxy-3-methoxyphenyl)acryloyl)urea (35)

Following our general procedure for the peptide coupling of hydroxyl urea (**32**) (195 mg, 2.57 mmol, 1 eq) with 4-hydroxy-3-methoxycinnamic acid (500 mg, 2.57 mmol, 1 eq), TEA (537 µL, 3.85 mmol, 1.5 eq), and BOP (1136 mg, 2.57 mmol, 1 eq), compound **35** was obtained as a light yellow solid after flash chromatography (70% EtOAc/Hex), yield = 38 %, Rf = 0.24 (80 % EtOAc/Hex), m.p. = 150-152˚C. ^{1}H-NMR (400 MHz, DMSO-d6, 25°C); δ (ppm): 9.69 (s, 1H, OH - D_2O exchange), 9.58 (s, 1H, OH - D_2O exchange), 7.69-7.65 (d, 1H, J = 16 Hz, C$\underline{H}$=CHCO), 7.36-7.35 (d, 1H, J = 2 Hz, H2), 7.19-7.16 (m, 1H, H6), 6.83-6.81 (d, 1H, J = 8 Hz, $H5_r$), 6.58-6.54 (d, 1H, J = 16 Hz, CH=C$\underline{H}$CO), 6.49 (s, 2H, NH_2 - D_2O exchange), 3.83 (s, 3H, OCH_3). ^{13}C-NMR (101 MHz, DMSO-d6, 25°C); δ (ppm): 166.64 (C=O), 159.97 (NCON), 150.19 (C3), 148.42 (C4), 146.89 (C$\underline{C}$H=CH), 125.94 (C1), 123.92 (C6), 116.03 (CCH=$\underline{C}$H)), 111.99 (C5), 111.85 (C2), 56.18 (OCH_3). HRMS *m*/*z* calc. for $C_{11}H_{12}N_2O_5$ + (Na^+): 275.0644; found: 275.0640.

1-Hydroxy-1-((E)-3-(3,4-dimethoxyphenyl)acryloyl)urea (36)

Following our general procedure for the peptide coupling of hydroxyl urea (**32**) (183 mg, 2.4 mmol, 2 eq) with 3,4-

dimethoxycinnamic acid (250 mg, 1.2 mmol, 1 eq), TEA (334 µL, 2.4 mmol, 2 eq), and BOP (530 mg, 1.2 mmol, 1 eq), compound **36** was obtained as a white solid after filtering through celite, quantitative yield, Rf = 0.49 (50% EtOAc/Hex), m.p. = 195-197˚C. ^{1}H-NMR (400 MHz, DMSO-d6, 25°C); δ (ppm) : 9.67 (s, 1H, OH - D_2O exchange), 7.72-7.68 (d, 1H, J = 16 Hz, CCH=CH), 7.39-7.38 (d, 1H, J = 2 Hz, H2), 7.31-7.29 (d, 1H, J = 8 Hz, $H6_r$), 7.02-7.00 (d, 1H, J = 8 Hz, H5), 6.67-6.63 (d, 1H, J = 16 Hz, CCH=CH), 6.52 (s, 2H, NH_2 - D_2O exchange), 3.82 (s, 3H, OCH_3), 3.81 (s, 3H, OCH_3). ^{13}C-NMR (101 MHz, DMSO-d6, 25°C); δ (ppm): 166.51 (C=O), 159.95 (NCON), 151.75 (C3), 149.45 (C4), 146.50 (CCH=CH), 127.20 (C1), 123.75 (C6), 113.15 CCH=CH), 112.02 (C5), 110.94 (C2), 56.11 (CH_3), 56.07 (OCH_3). HRMS *m/z* calc. for $C_{12}H_{14}N_2O_5$ + (Na^+): 289.0795; found: 289.0778.

1-Hydroxy-1-((E)-3-(4-hydroxy-3,5-dimethoxyphenyl)acryloyl)urea (37)

Following our general procedure for the peptide coupling of hydroxyl urea (**32**) (85 mg, 1.12 mmol, 1 eq) with 4-hydroxy-3,5-dimethoxycinnamic acid (250 mg, 1.12 mmol, 1 eq), TEA (234 µL, 1.68 mmol, 1.5 eq), and BOP (495 mg, 1.12 mmol, 1 eq), compound **37** was obtained as a light yellow solid after flash chromatography (75% EtOAc/Hex), yield = 23 %, Rf = 0.15 (80 % EtOAc/Hex), m.p. = 164-166˚C. ^{1}H-NMR (400 MHz, DMSO-d6, 25°C) ; δ (ppm) : 9.59 (s, 1H, OH - D_2O exchange), 9.05 (s, 1H, OH - D_2O exchange), 7.69-7.65 (d, 1H, J = 16 Hz, CCH=CH), 7.08 (s, 2H, NH_2 - D_2O exchange), 6.64-6.60 (d, 1H, J = 16 Hz, CCH=CH), 6.49 (s, 2H, H2, H6), 3.81 (s, 6H, OCH_3). ^{13}C-NMR (101 MHz, DMSO-d6, 25°C); δ (ppm): 166.61 (C=O), 159.96 (NCON), 148.51 (C3, C5), 147.20 (CCH=CH), 139.17 (C4), 124.75 (C1), 112.44 (CCH=CH), 106.93 (C2, C6), 56.58 (OCH_3). HRMS *m/z* calc. for $C_{12}H_{14}N_2O_6$ + (Na^+):305.0732; found:305.0744.

1-(3-Phenylpropanoyl)-1-hydroxyurea (40)

Following our general procedure for hydroxyurea analogs synthesis with acyl chlorides, hydroxyurea (**32**) (245 mg, 3.23 mmol), DMAP (394 mg, 3.23 mmol), and hydrocinnamoyl chloride (500 mg, 3.23 mmol) compound **40** was obtained as a white solid after flash chromatography, yield = 65 %, Rf = 0.25 (50 % EtOAc/Hex), m.p. = 79-81˚C.^{1}H-NMR (400 MHz, DMSO-d6, 25°C); δ (ppm): 9.55 (s, 1H, OH - D_2O exchange), 7.31-7.25 (m, 4H, H2, H3, H5, H6), 7.22-7.19 (m, 1H, H4), 6.49 (s, 2H, NH_2 – D_2O exchange), 2.90-2.87 (t, 2H, J = 7 Hz, CCH_2CH_2), 2.78-2.74 (t, 2H, J = 7 Hz, CCH_2CH_2). ^{13}C-NMR (101 MHz, DMSO-d6, 25°C); δ (ppm): 172.09 (C=O), 159.95 (NCON), 140.70 (C1), 128.83 (C3, C5), 128.73 (C2, C6), 126.63 (C4), 33.25 (CCH_2CH_2), 30.28 (CCH_2CH_2),. LC-MS *m/z* calc. for $C_{10}H_{12}N_2O_3$ + (Na^+): 231.0740; found: 231.0745.

^{1}H NMR

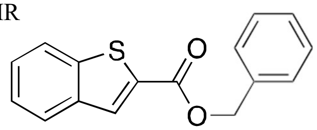

4

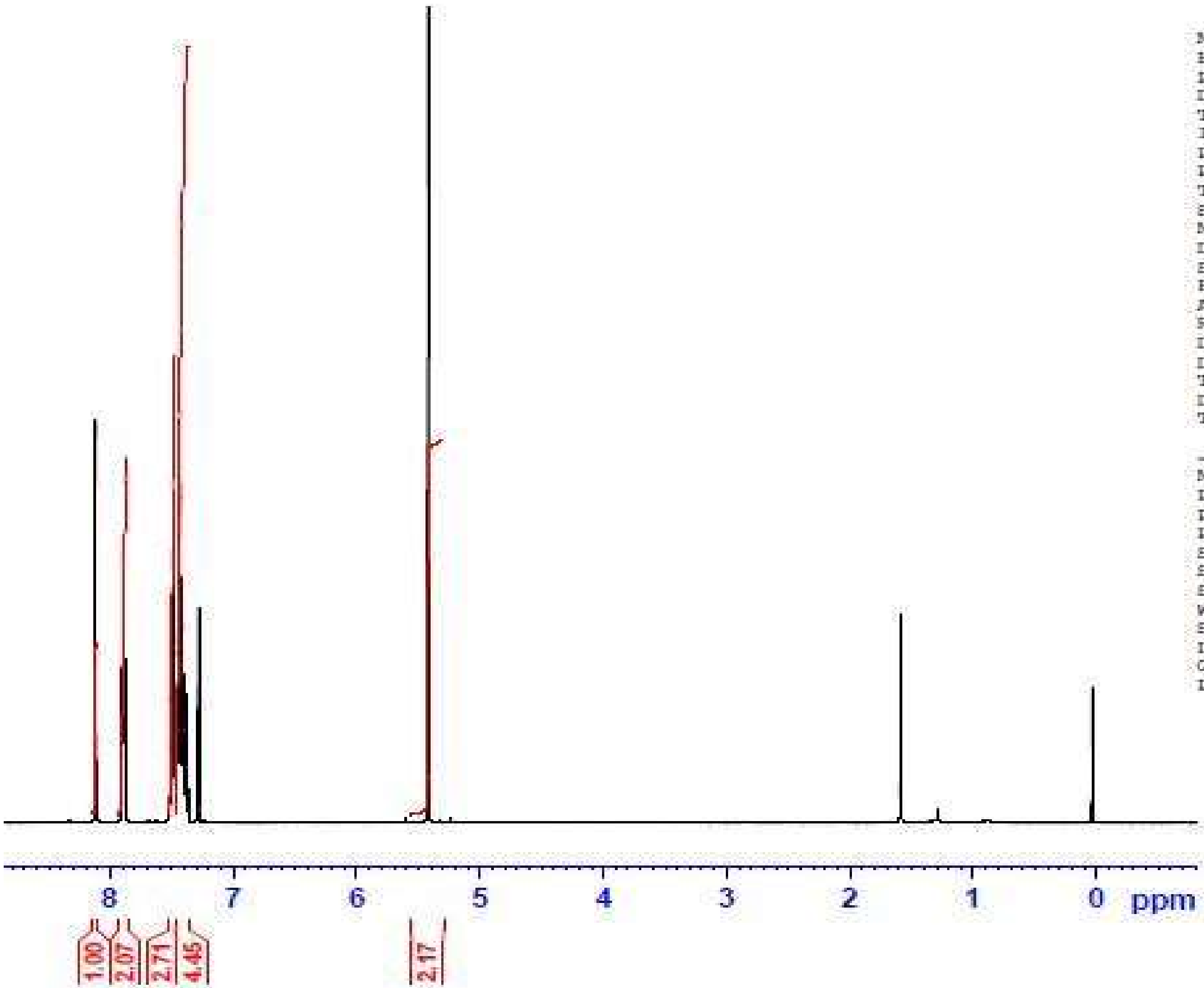

BRUKER

NAME	Audrey Isabelle	
EXPNO	8	
PROCNO	1	
Date_	20190131	
Time	11.15	
INSTRUM	spect	
PROBHD	5 mm PABBO BB-	
PULPROG	zg30	
TD	65536	
SOLVENT	CDCl3	
NS	16	
DS	2	
SWH	8223.685	Hz
FIDRES	0.125483	Hz
AQ	3.9846387	sec
RG	101	
DW	60.800	usec
DE	6.50	usec
TE	298.0	K
D1	1.00000000	sec
TD0	1	

======== CHANNEL f1 ========

NUC1	1H	
P1	14.07	usec
PL1	0.30	dB
PL1W	11.25229836	W
SFO1	400.1324710	MHz
SI	32768	
SF	400.1300000	MHz
WDW	EM	
SSB	0	
LB	0.30	Hz
GB	0	
PC	1.00	

^{13}C NMR

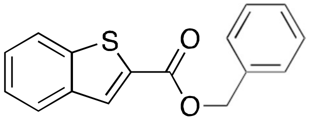
S
O
O

4

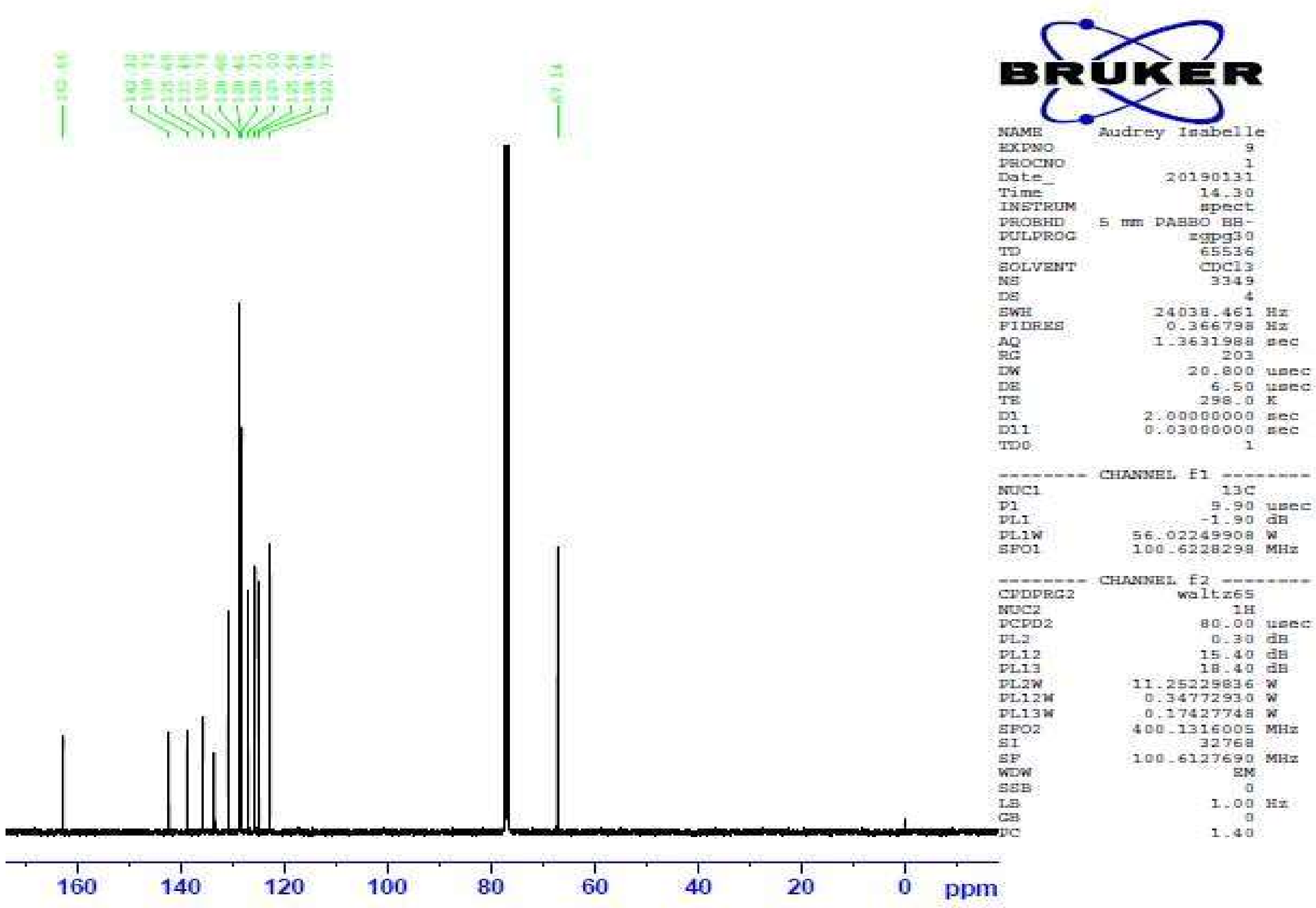
BRUKER
NAME Audrey Isabelle
EXPNO 9
PROCNO 1
Date_ 20190131
Time 14.30
INSTRUM spect
PROBHD 5 mm PABBO BB-
PULPROG zgpg30
TD 65536
SOLVENT CDCl3
NS 3349
DS 4
SWH 24038.461 Hz
FIDRES 0.366798 Hz
AQ 1.3631988 sec
RG 203
DW 20.800 usec
DE 6.50 usec
TE 298.0 K
D1 2.00000000 sec
D11 0.03000000 sec
TD0 1
======== CHANNEL f1 ========
NUC1 13C
P1 9.90 usec
PL1 -1.90 dB
PL1W 56.02249908 W
SFO1 100.6228298 MHz
======== CHANNEL f2 ========
CPDPRG2 waltz65
NUC2 1H
PCPD2 80.00 usec
PL2 0.30 dB
PL12 15.40 dB
PL13 18.40 dB
PL2W 11.25229836 W
PL12W 0.34772930 W
PL13W 0.17427748 W
SFO2 400.1316005 MHz
SI 32768
SF 100.6127690 MHz
WDW EM
SSB 0
LB 1.00 Hz
GB 0
PC 1.40
160
140
120
100
80
60
40
20
0
ppm

HRMS

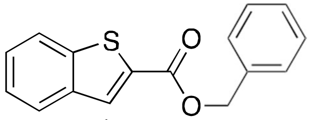

4

Compound Label	RT	Algorithm	Mass
Cpd 1: C16 H12 O2 S	0.13	Find By Formula	268.0556

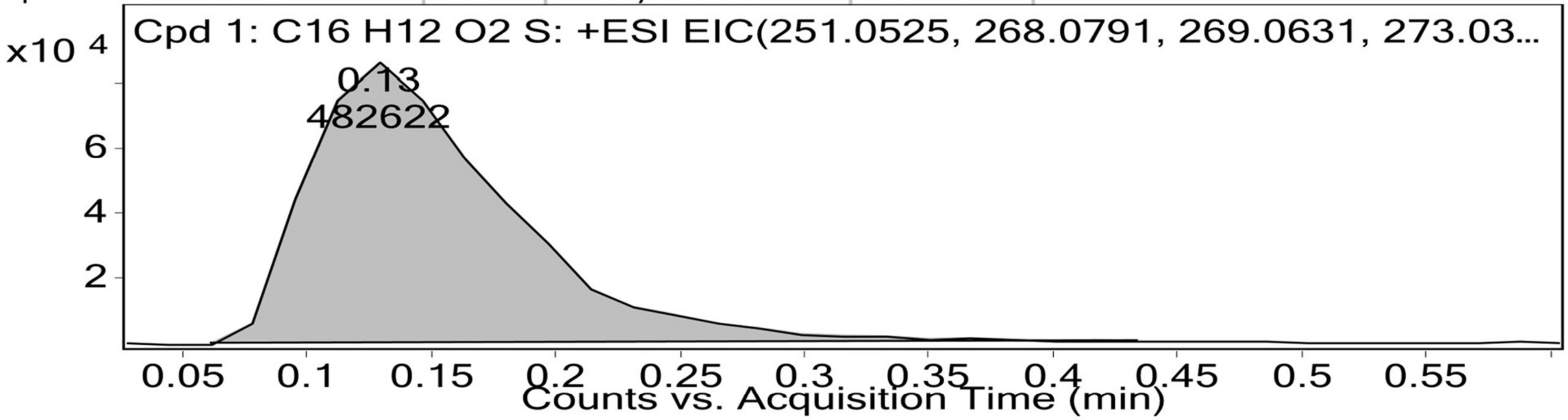

MS Spectrum

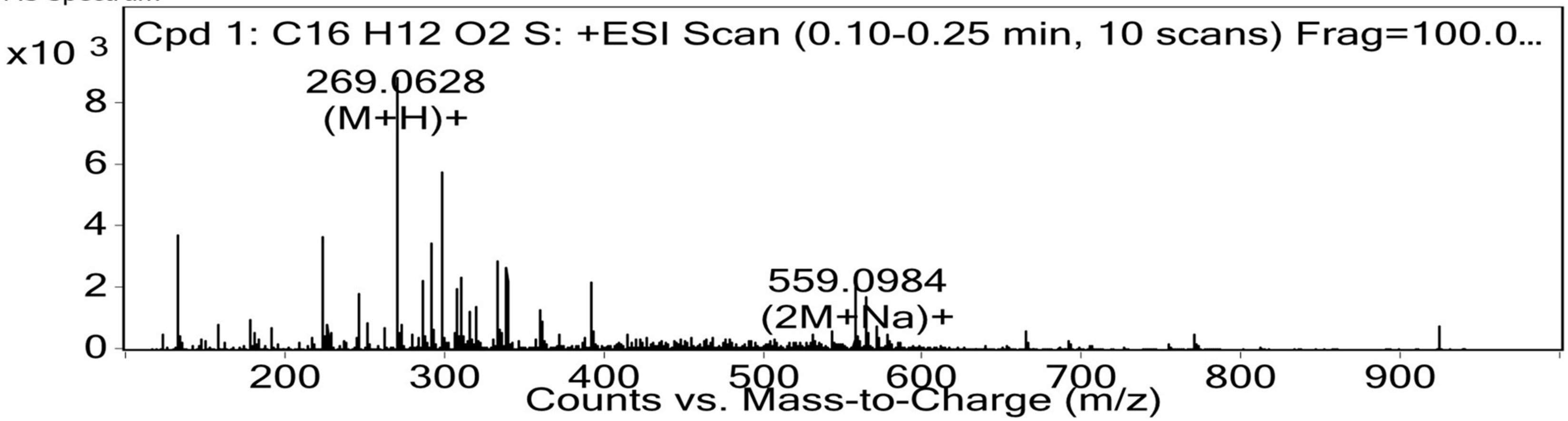

^{1}H NMR

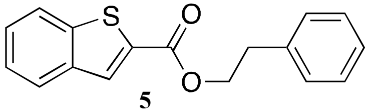

5

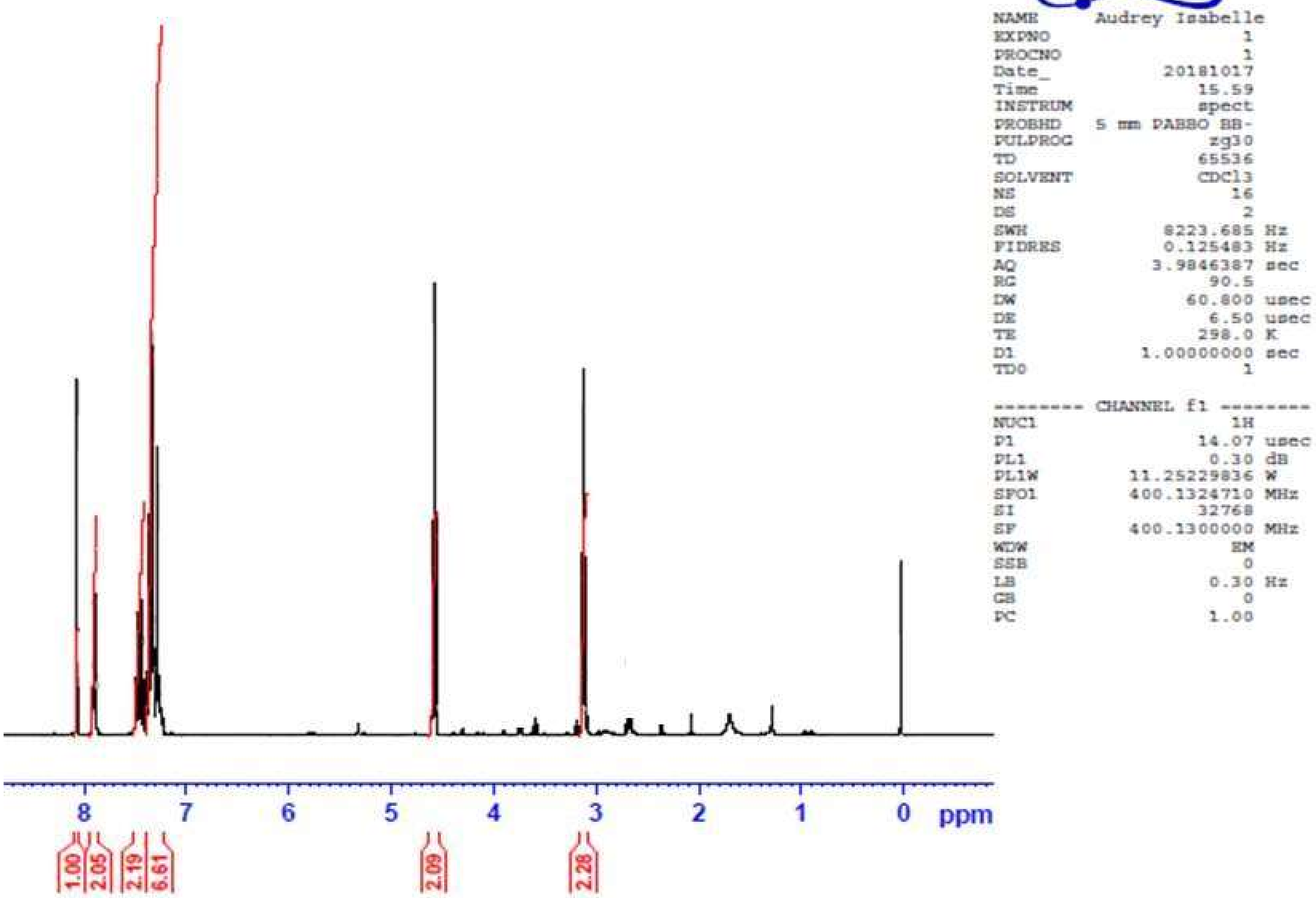

BRUKER
NAME Audrey Isabelle
EXPNO 1
PROCNO 1
Date_ 20181017
Time 15.59
INSTRUM spect
PROBHD 5 mm PABBO BB-
PULPROG zg30
TD 65536
SOLVENT CDCl3
NS 16
DS 2
SWH 8223.685 Hz
FIDRES 0.125483 Hz
AQ 3.9846387 sec
RG 90.5
DW 60.800 usec
DE 6.50 usec
TE 298.0 K
D1 1.00000000 sec
TD0 1
======== CHANNEL f1 ========
NUC1 1H
P1 14.07 usec
PL1 0.30 dB
PL1W 11.25229836 W
SFO1 400.1324710 MHz
SI 32768
SF 400.1300000 MHz
WDW EM
SSB 0
LB 0.30 Hz
GB 0
PC 1.00
8 7 6 5 4 3 2 1 0 ppm
1.00 2.05 2.19 6.61 2.09 2.28

^{13}C NMR

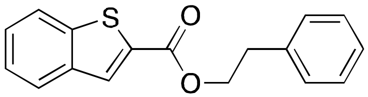

5

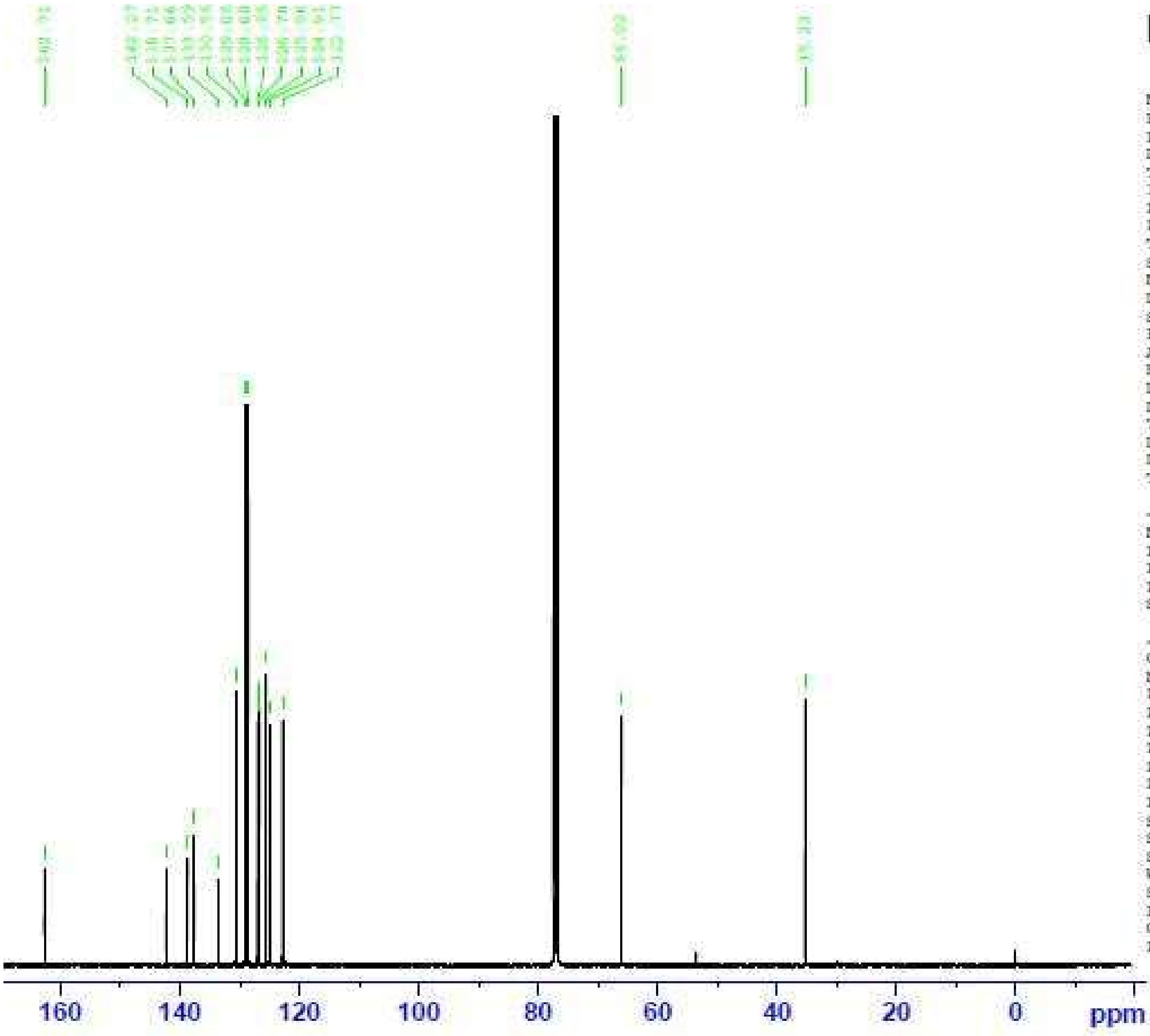


```
NAME          Audrey Isabelle
EXPNO                       4
PROCNO                      1
Date_                20181107
Time                    20.47
INSTRUM                 spect
PROBHD       5 mm PABBO BB-
PULPROG               zgpg30
TD                     65536
SOLVENT                CDCl3
NS                      5000
DS                         4
SWH                24038.461 Hz
FIDRES              0.366798 Hz
AQ                 1.3631988 sec
RG                       203
DW                    20.800 usec
DE                      6.50 usec
TE                     298.0 K
D1                2.00000000 sec
D11               0.03000000 sec
TD0                        1

======== CHANNEL f1 ========
NUC1                     13C
P1                      9.90 usec
PL1                    -1.90 dB
PL1W             56.02249908 W
SFO1             100.6228298 MHz

======== CHANNEL f2 ========
CPDPRG2              waltz65
NUC2                      1H
PCPD2                  80.00 usec
PL2                     0.30 dB
PL12                   15.40 dB
PL13                   18.40 dB
PL2W             11.25229836 W
PL12W             0.34772930 W
PL13W             0.17427748 W
SFO2             400.1316005 MHz
SI                     32768
SF               100.6127690 MHz
WDW                       EM
SSB                        0
LB                      1.00 Hz
GB                         0
PC                      1.40
```

HRMS

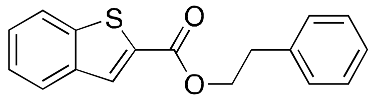

5

Compound Label	RT	Algorithm	Mass
Cpd 1: C17 H14 O2 S	0.13	Find By Formula	282.0712

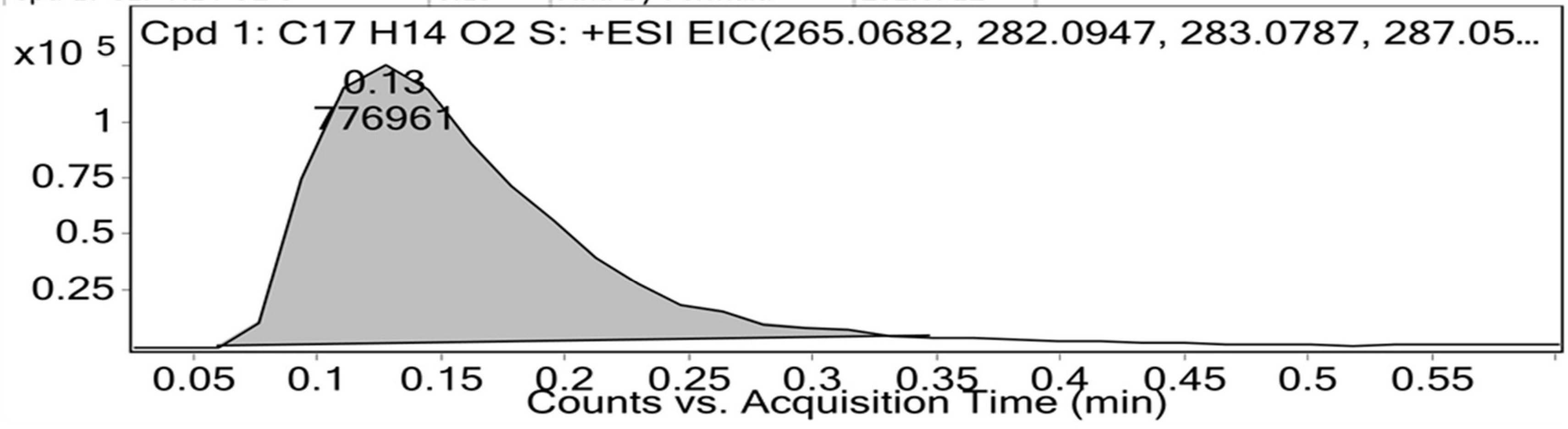

MS Spectrum

MS Zoomed Spectrum

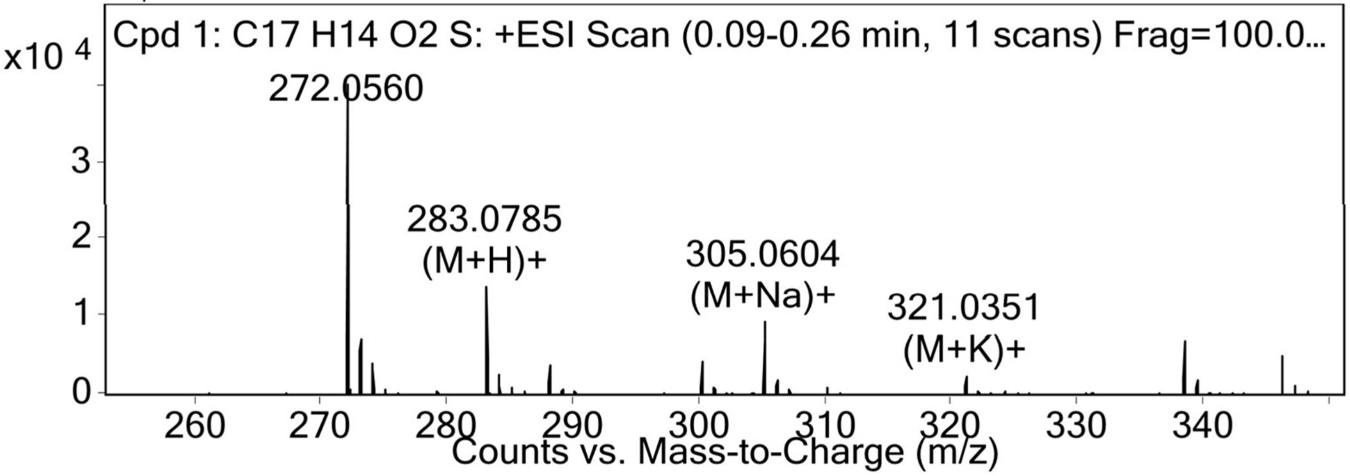

^{1}H NMR

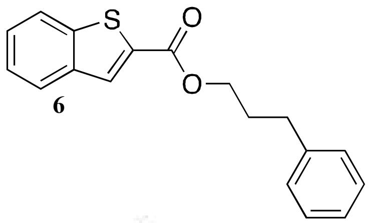

6

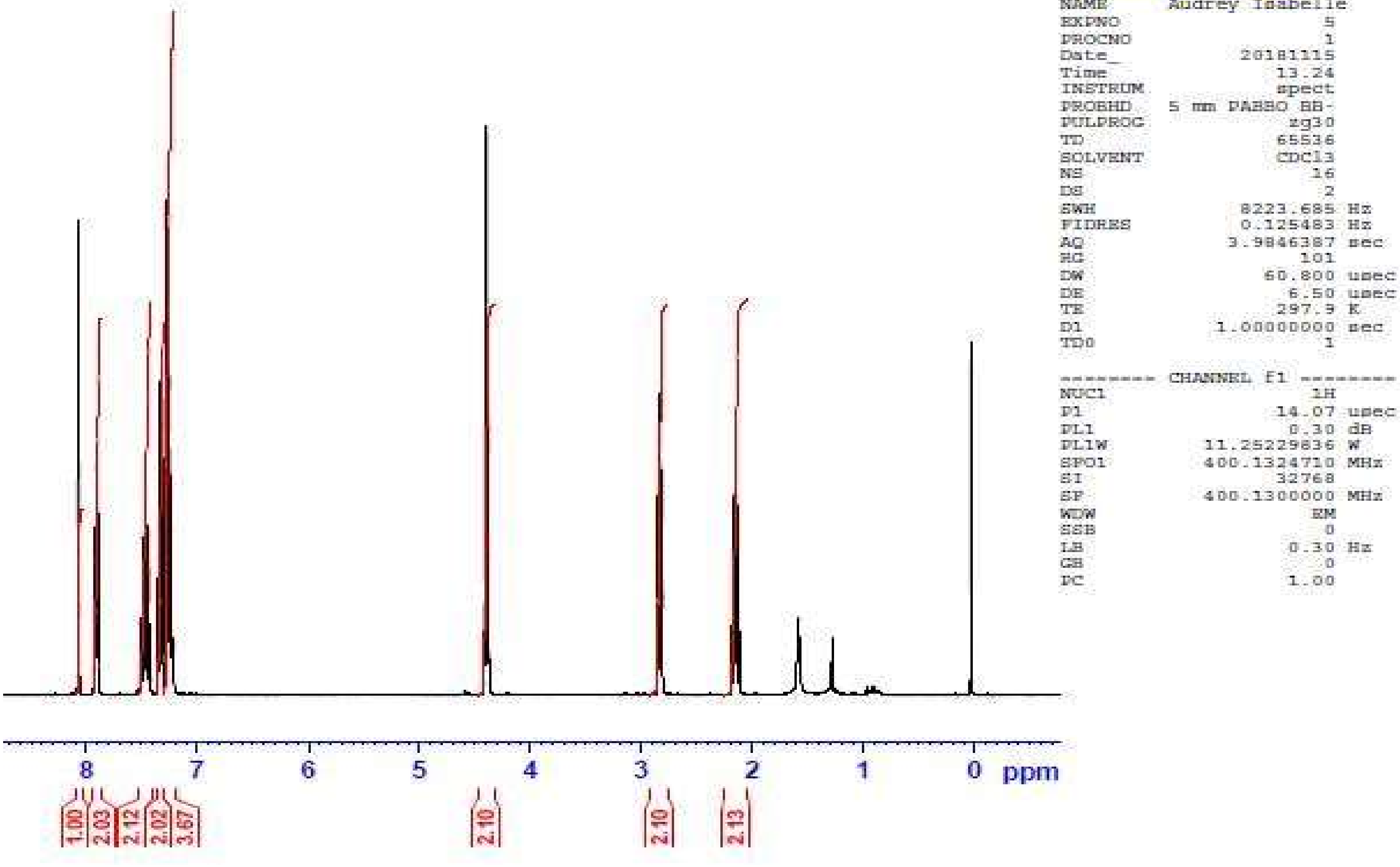

8
7
6
5
4
3
2
1
0
ppm
1.00
2.03
2.12
2.02
3.67
2.10
2.10
2.13
NAME Audrey Isabelle
EXPNO 5
PROCNO 1
Date_ 20181115
Time 13.24
INSTRUM spect
PROBHD 5 mm PABBO BB-
PULPROG zg30
TD 65536
SOLVENT CDCl3
NS 16
DS 2
SWH 8223.685 Hz
FIDRES 0.125483 Hz
AQ 3.9846387 sec
RG 101
DW 60.800 usec
DE 6.50 usec
TE 297.9 K
D1 1.00000000 sec
TD0 1
======== CHANNEL f1 ========
NUC1 1H
P1 14.07 usec
PL1 0.30 dB
PL1W 11.25229836 W
SFO1 400.1324710 MHz
SI 32768
SF 400.1300000 MHz
WDW EM
SSB 0
LB 0.30 Hz
GB 0
PC 1.00

^{13}C NMR

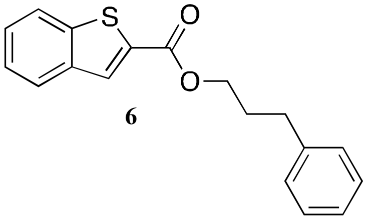

6

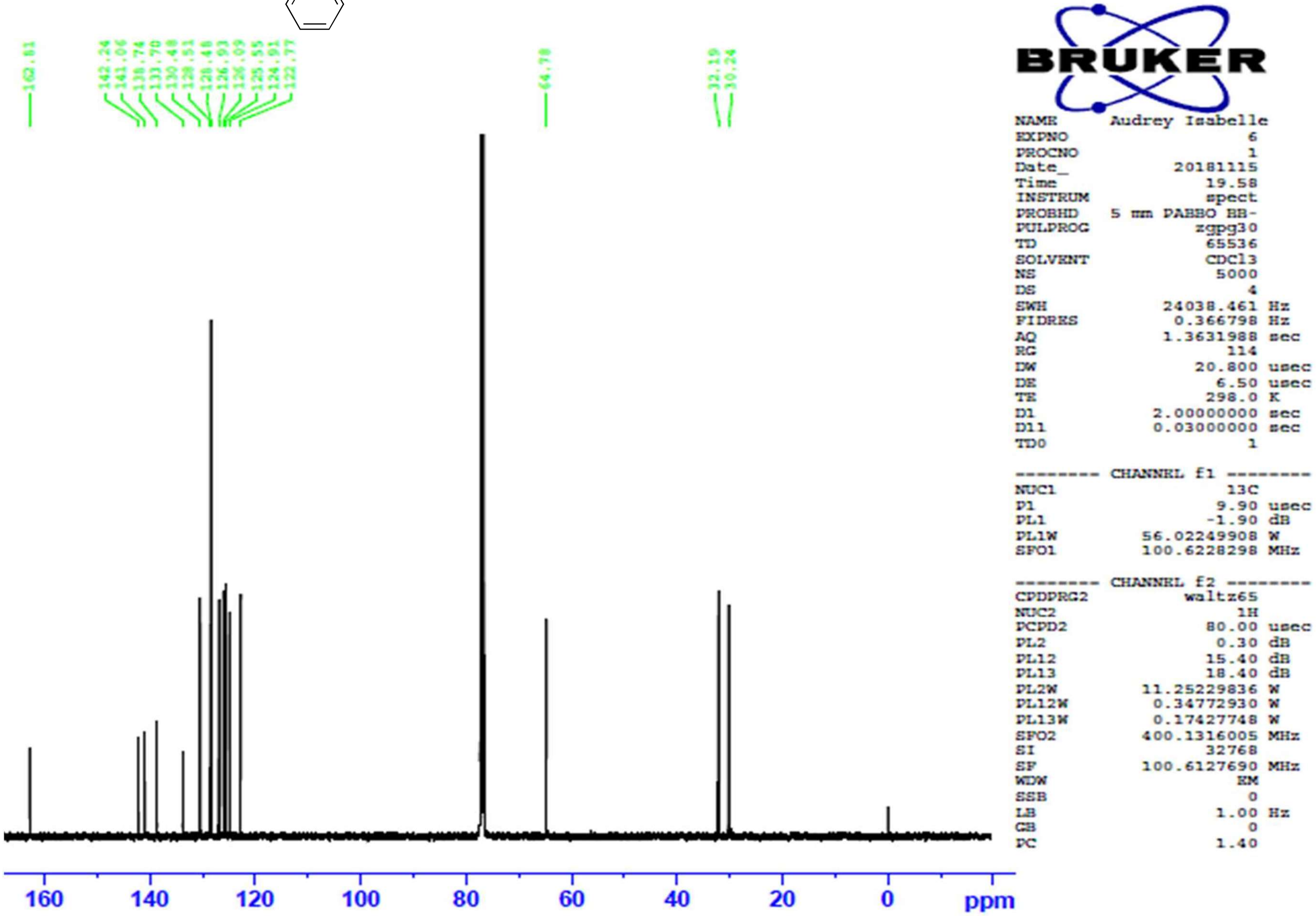
162.61
142.24
141.06
138.74
133.70
130.48
128.51
128.46
126.93
126.09
125.55
124.91
122.77
64.78
32.19
30.24
BRUKER
NAME Audrey Isabelle
EXPNO 6
PROCNO 1
Date_ 20181115
Time 19.58
INSTRUM spect
PROBHD 5 mm PABBO BB-
PULPROG zgpg30
TD 65536
SOLVENT CDCl3
NS 5000
DS 4
SWH 24038.461 Hz
FIDRES 0.366798 Hz
AQ 1.3631988 sec
RG 114
DW 20.800 usec
DE 6.50 usec
TE 298.0 K
D1 2.00000000 sec
D11 0.03000000 sec
TD0 1
======== CHANNEL f1 ========
NUC1 13C
P1 9.90 usec
PL1 -1.90 dB
PL1W 56.02249908 W
SFO1 100.6228298 MHz
======== CHANNEL f2 ========
CPDPRG2 waltz65
NUC2 1H
PCPD2 80.00 usec
PL2 0.30 dB
PL12 15.40 dB
PL13 18.40 dB
PL2W 11.25229836 W
PL12W 0.34772930 W
PL13W 0.17427748 W
SFO2 400.1316005 MHz
SI 32768
SF 100.6127690 MHz
WDW EM
SSB 0
LB 1.00 Hz
GB 0
PC 1.40
160
140
120
100
80
60
40
20
0
ppm

HRMS

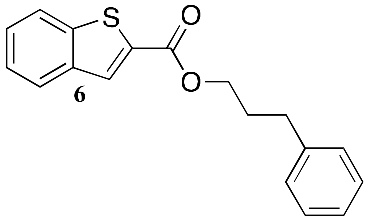

6

Compound Label	RT	Algorithm	Mass
Cpd 1: C18 H16 O2 S	0.12	Find By Formula	296.0872

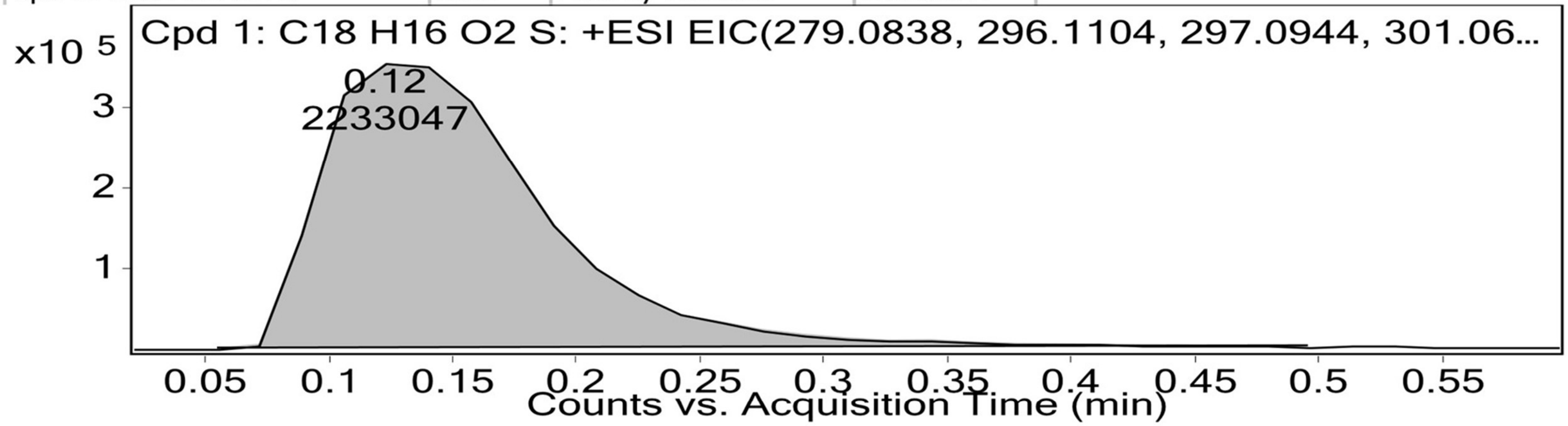

MS Spectrum

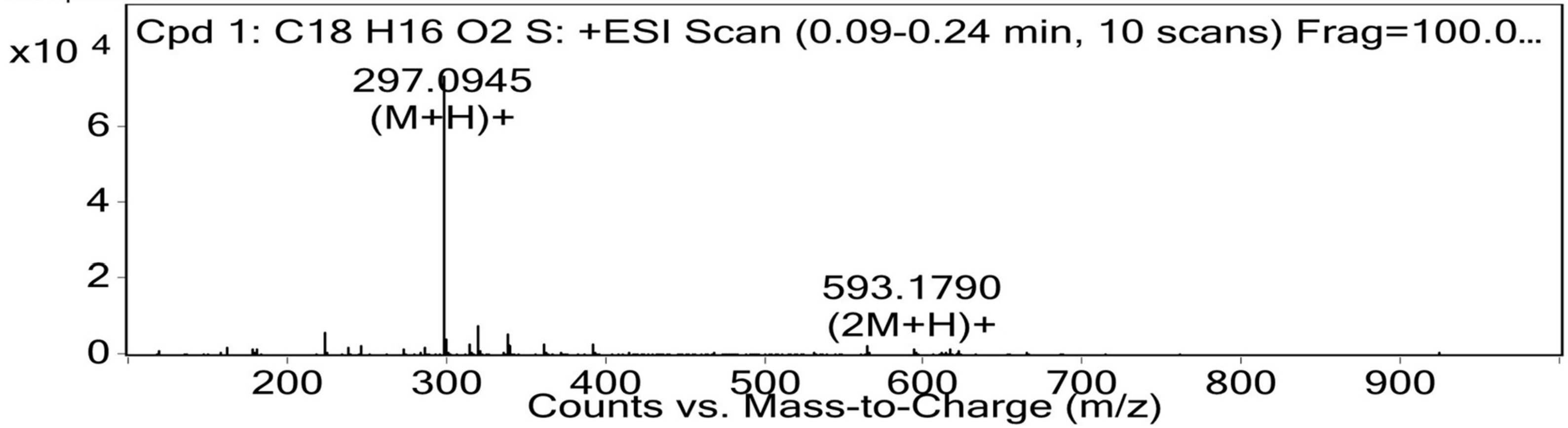

^{1}H NMR

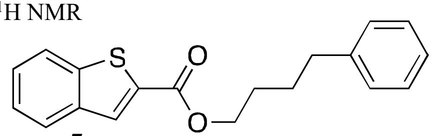

7

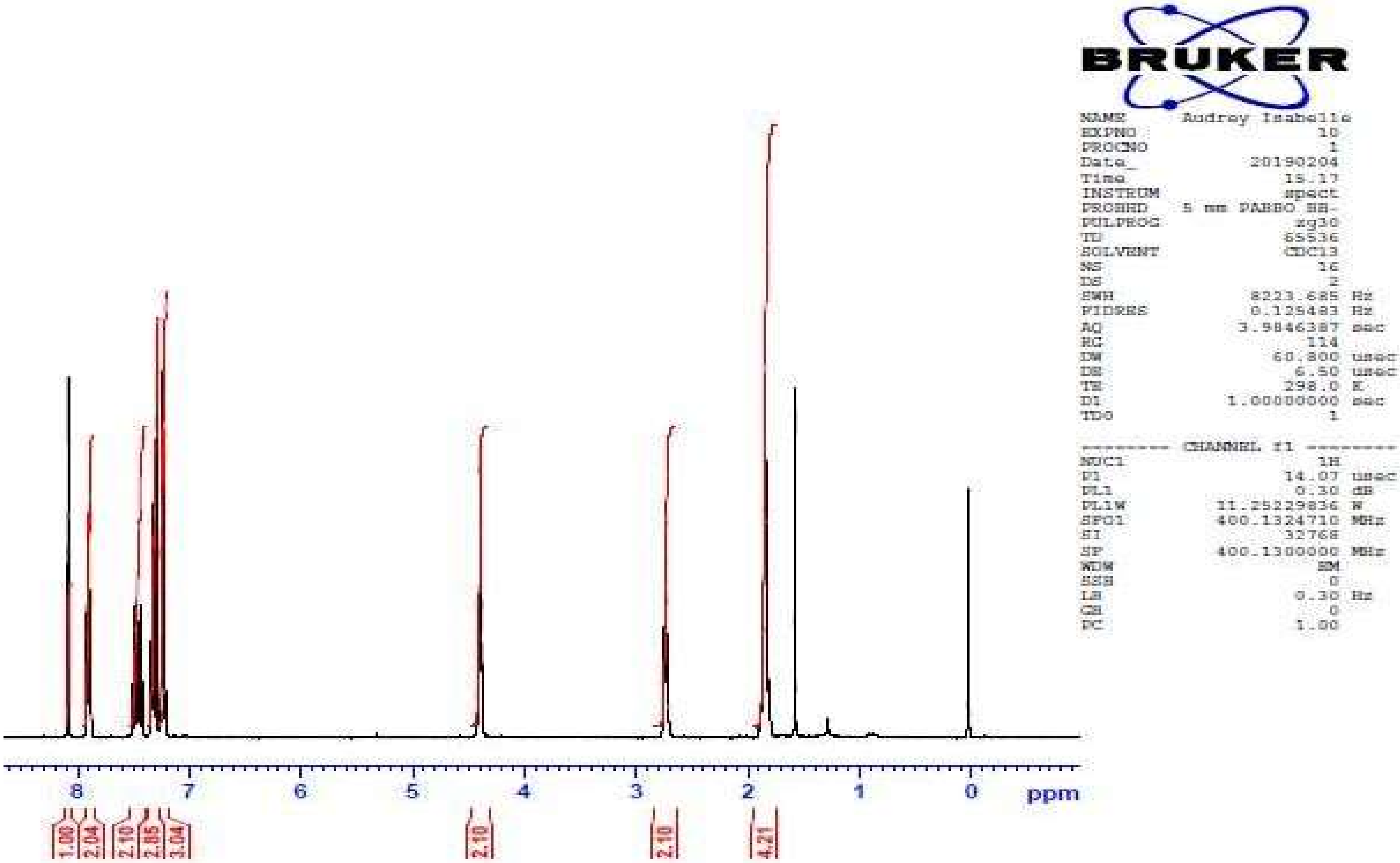
BRUKER
NAME Audrey Isabelle
EXPNO 10
PROCNO 1
Date_ 20190204
Time 15.17
INSTRUM spect
PROBHD 5 mm PABBO BB-
PULPROG zg30
TD 65536
SOLVENT CDCl3
NS 16
DS 2
SWH 8223.685 Hz
FIDRES 0.125483 Hz
AQ 3.9846387 sec
RG 114
DW 60.800 usec
DE 6.50 usec
TE 298.0 K
D1 1.00000000 sec
TD0 1
======== CHANNEL f1 ========
NUC1 1H
P1 14.07 usec
PL1 0.30 dB
PL1W 11.25229836 W
SFO1 400.1324710 MHz
SI 32768
SF 400.1300000 MHz
WDW EM
SSB 0
LB 0.30 Hz
GB 0
PC 1.00
8
7
6
5
4
3
2
1
0
ppm
1.00
2.04
2.10
2.85
3.04
2.10
2.10
4.21

^{13}C NMR

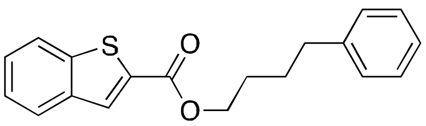

7

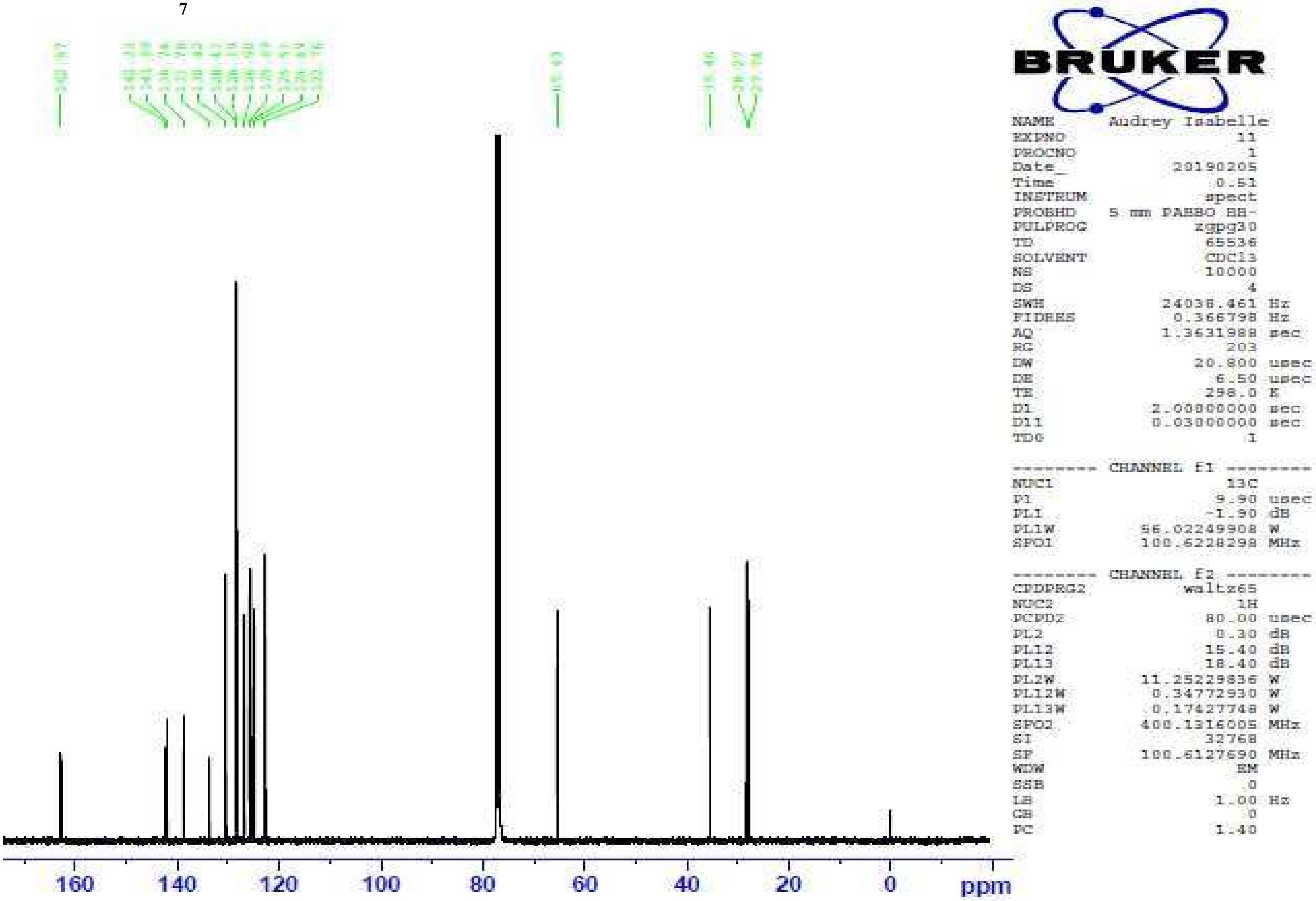
BRUKER
NAME Audrey Isabelle
EXPNO 11
PROCNO 1
Date_ 20190205
Time 0.51
INSTRUM spect
PROBHD 5 mm PABBO BB-
PULPROG zgpg30
TD 65536
SOLVENT CDCl3
NS 10000
DS 4
SWH 24038.461 Hz
FIDRES 0.366798 Hz
AQ 1.3631988 sec
RG 203
DW 20.800 usec
DE 6.50 usec
TE 298.0 K
D1 2.00000000 sec
D11 0.03000000 sec
TD0 1
======== CHANNEL f1 ========
NUC1 13C
P1 9.90 usec
PL1 -1.90 dB
PL1W 56.02249908 W
SFO1 100.6228298 MHz
======== CHANNEL f2 ========
CPDPRG2 waltz65
NUC2 1H
PCPD2 80.00 usec
PL2 0.30 dB
PL12 15.40 dB
PL13 18.40 dB
PL2W 11.25229836 W
PL12W 0.34772930 W
PL13W 0.17427748 W
SFO2 400.1316005 MHz
SI 32768
SF 100.6127690 MHz
WDW EM
SSB 0
LB 1.00 Hz
GB 0
PC 1.40
160
140
120
100
80
60
40
20
0
ppm

HRMS

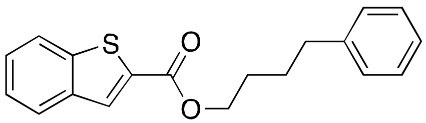

7

Compound Label	RT	Algorithm	Mass
Cpd 1: C19 H18 O2 S	0.12	Find By Formula	310.1023

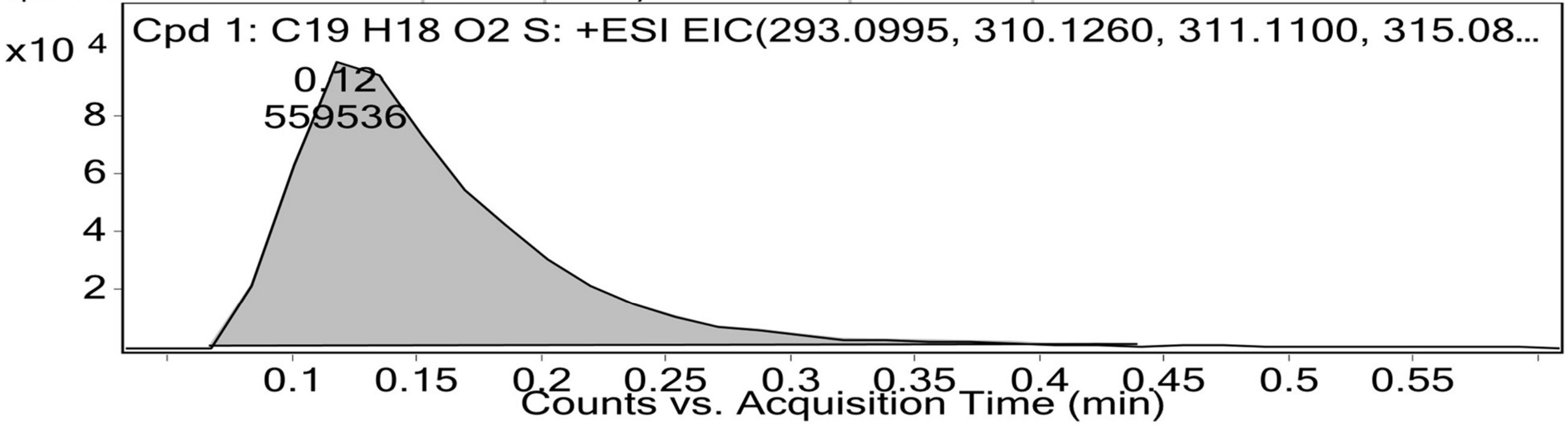

MS Spectrum

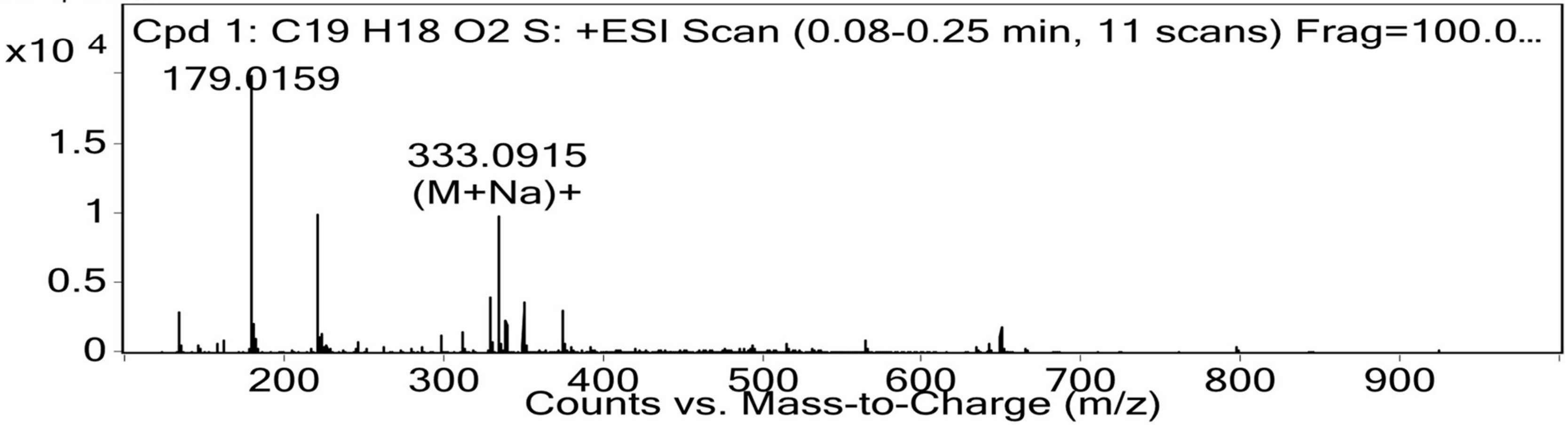

^1H NMR

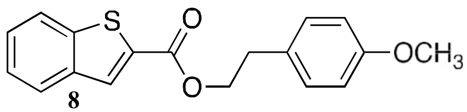

8

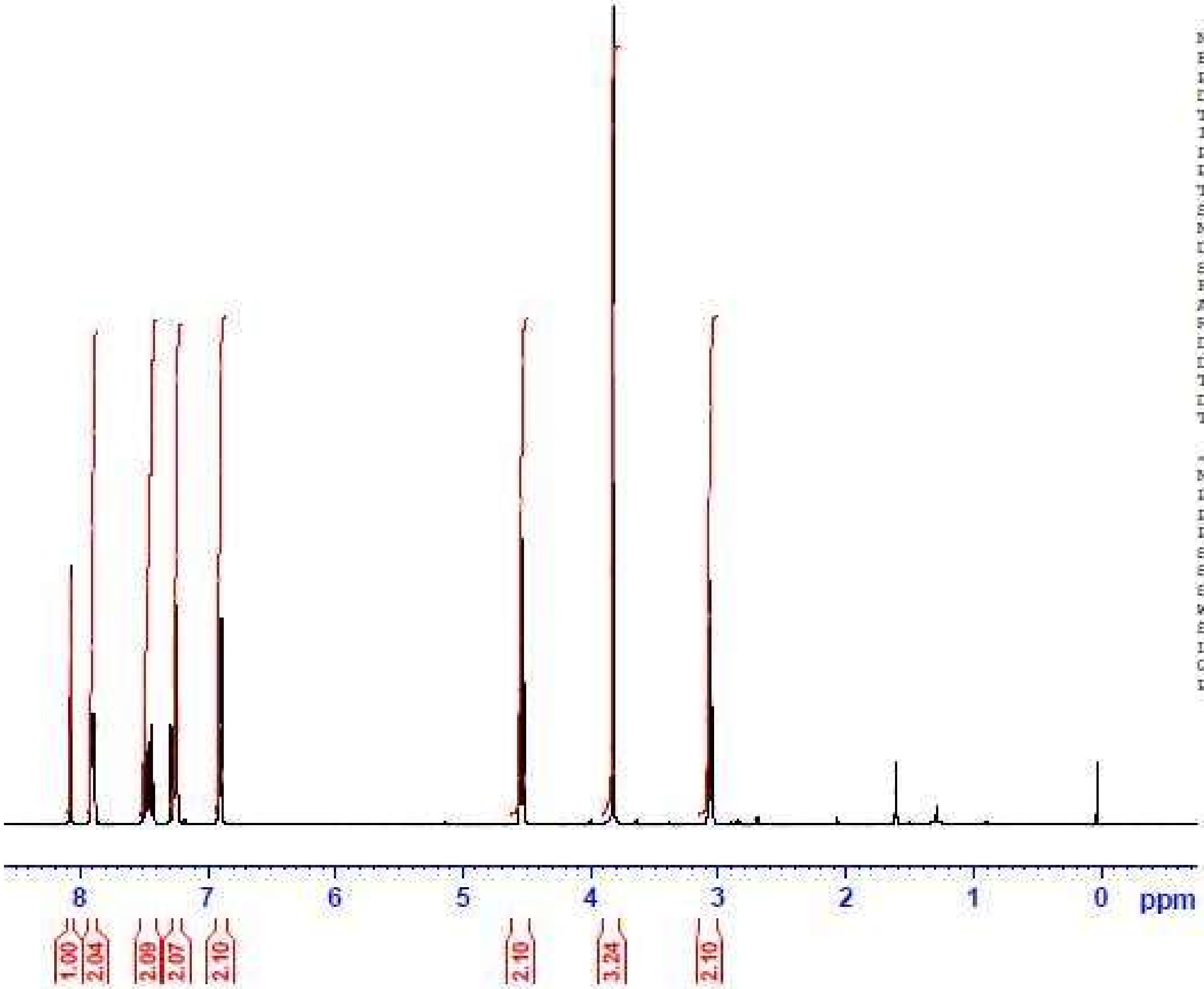

BRUKER

NAME	Audrey Isabelle
EXPNO	12
PROCNO	1
Date_	20190211
Time	10.35
INSTRUM	spect
PROBHD	5 mm PABBO BB-
PULPROG	zg30
TD	65536
SOLVENT	CDCl3
NS	16
DS	2
SWH	8223.685 Hz
FIDRES	0.125483 Hz
AQ	3.9846387 sec
RG	80.6
DW	60.800 usec
DE	6.50 usec
TE	298.0 K
D1	1.00000000 sec
TD0	1
======== CHANNEL f1 ========	
NUC1	1H
P1	14.07 usec
PL1	0.30 dB
PL1W	11.25229836 W
SFO1	400.1324710 MHz
SI	32768
SF	400.1300000 MHz
WDW	EM
SSB	0
LB	0.30 Hz
GB	0
PC	1.00

^{13}C NMR

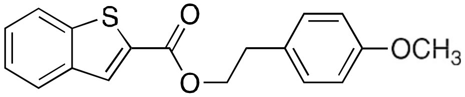
S
O
O
OCH3

8

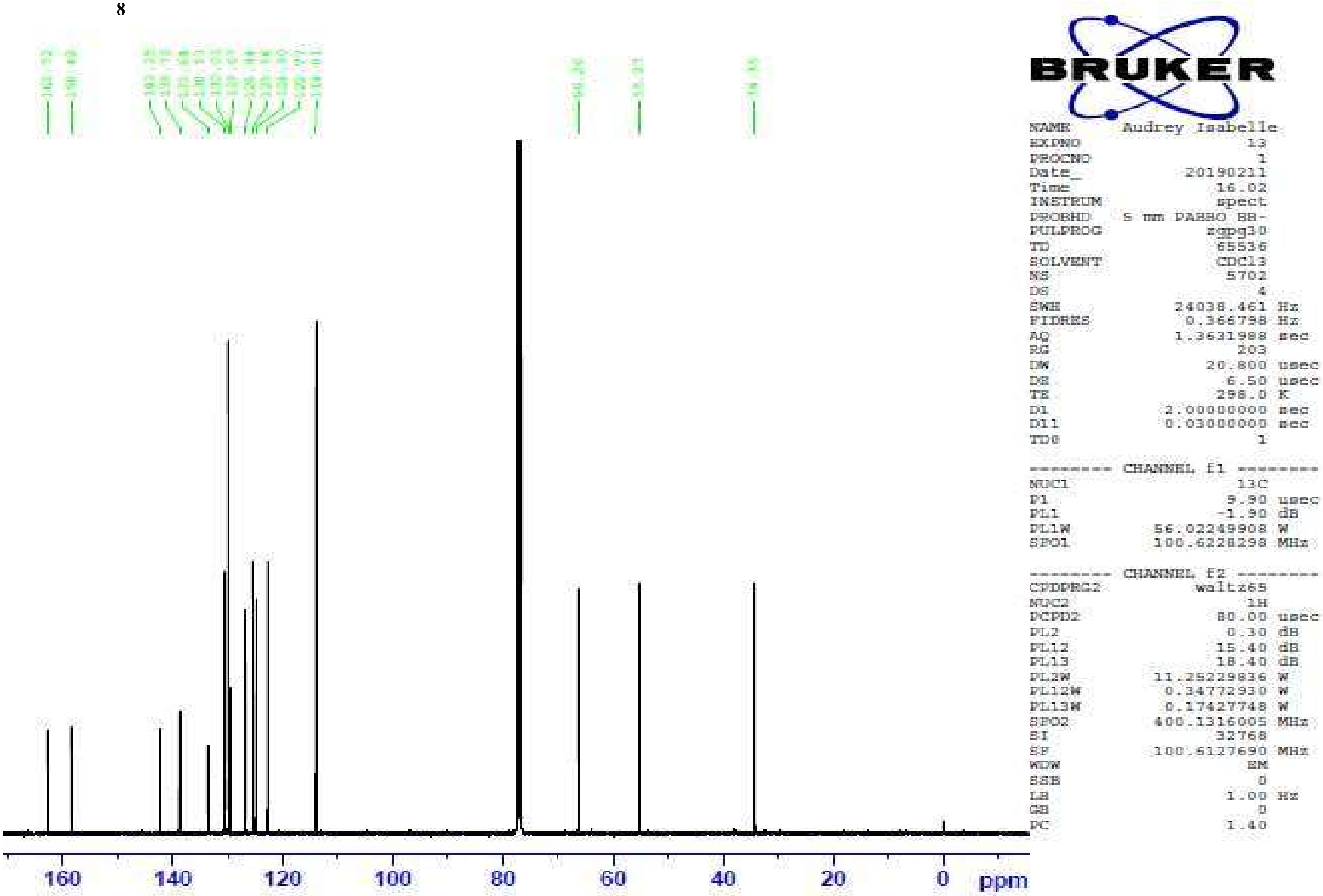
BRUKER
NAME Audrey Isabelle
EXPNO 13
PROCNO 1
Date_ 20190211
Time 16.02
INSTRUM spect
PROBHD 5 mm PABBO BB-
PULPROG zgpg30
TD 65536
SOLVENT CDCl3
NS 5702
DS 4
SWH 24038.461 Hz
FIDRES 0.366798 Hz
AQ 1.3631988 sec
RG 203
DW 20.800 usec
DE 6.50 usec
TE 298.0 K
D1 2.00000000 sec
D11 0.03000000 sec
TD0 1
======== CHANNEL f1 ========
NUC1 13C
P1 9.90 usec
PL1 -1.90 dB
PL1W 56.02249908 W
SFO1 100.6228298 MHz
======== CHANNEL f2 ========
CPDPRG2 waltz65
NUC2 1H
PCPD2 80.00 usec
PL2 0.30 dB
PL12 15.40 dB
PL13 18.40 dB
PL2W 11.25229836 W
PL12W 0.34772930 W
PL13W 0.17427748 W
SFO2 400.1316005 MHz
SI 32768
SF 100.6127690 MHz
WDW EM
SSB 0
LB 1.00 Hz
GB 0
PC 1.40
160
140
120
100
80
60
40
20
0
ppm

HRMS

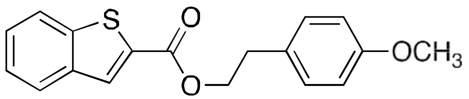

8

Compound Label	RT	Algorithm	Mass
Cpd 1: C18 H16 O3 S	0.12	Find By Formula	312.0813

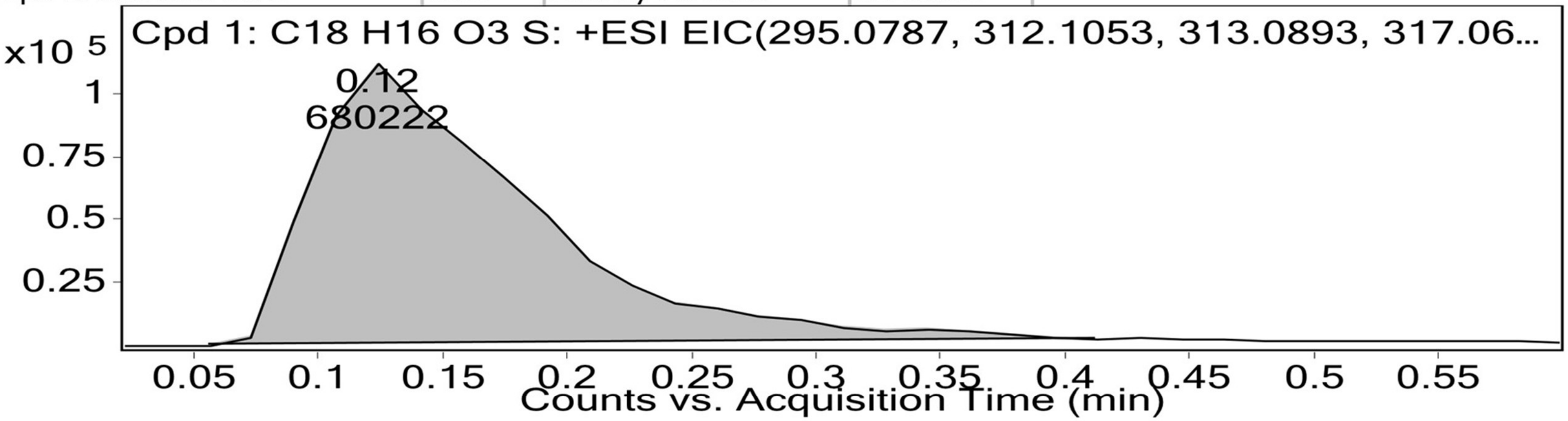

MS Spectrum

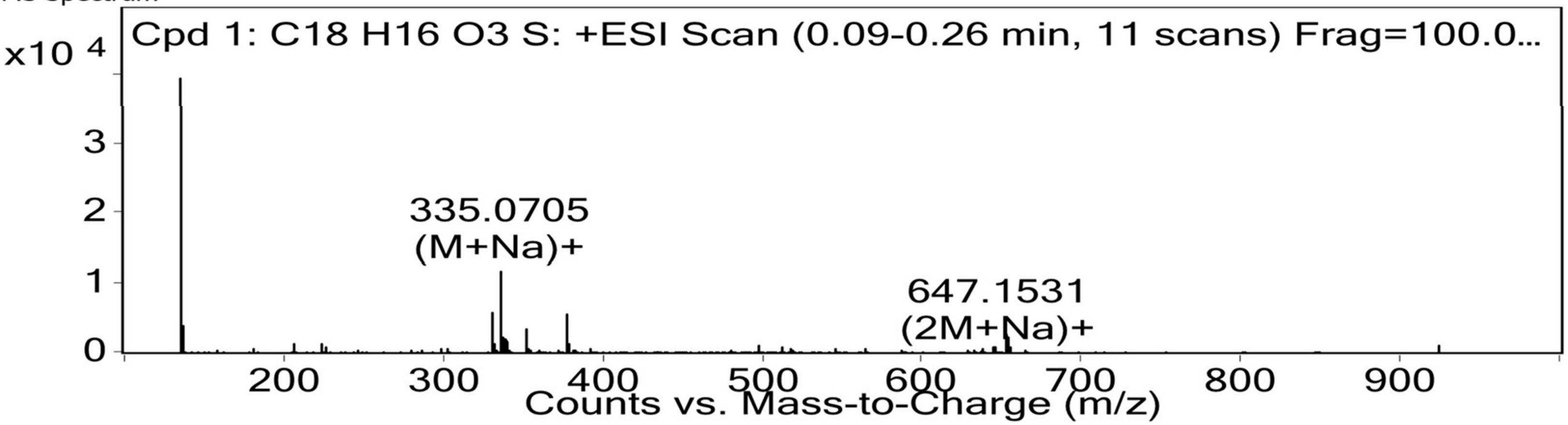

^{1}H NMR

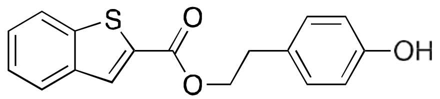

9

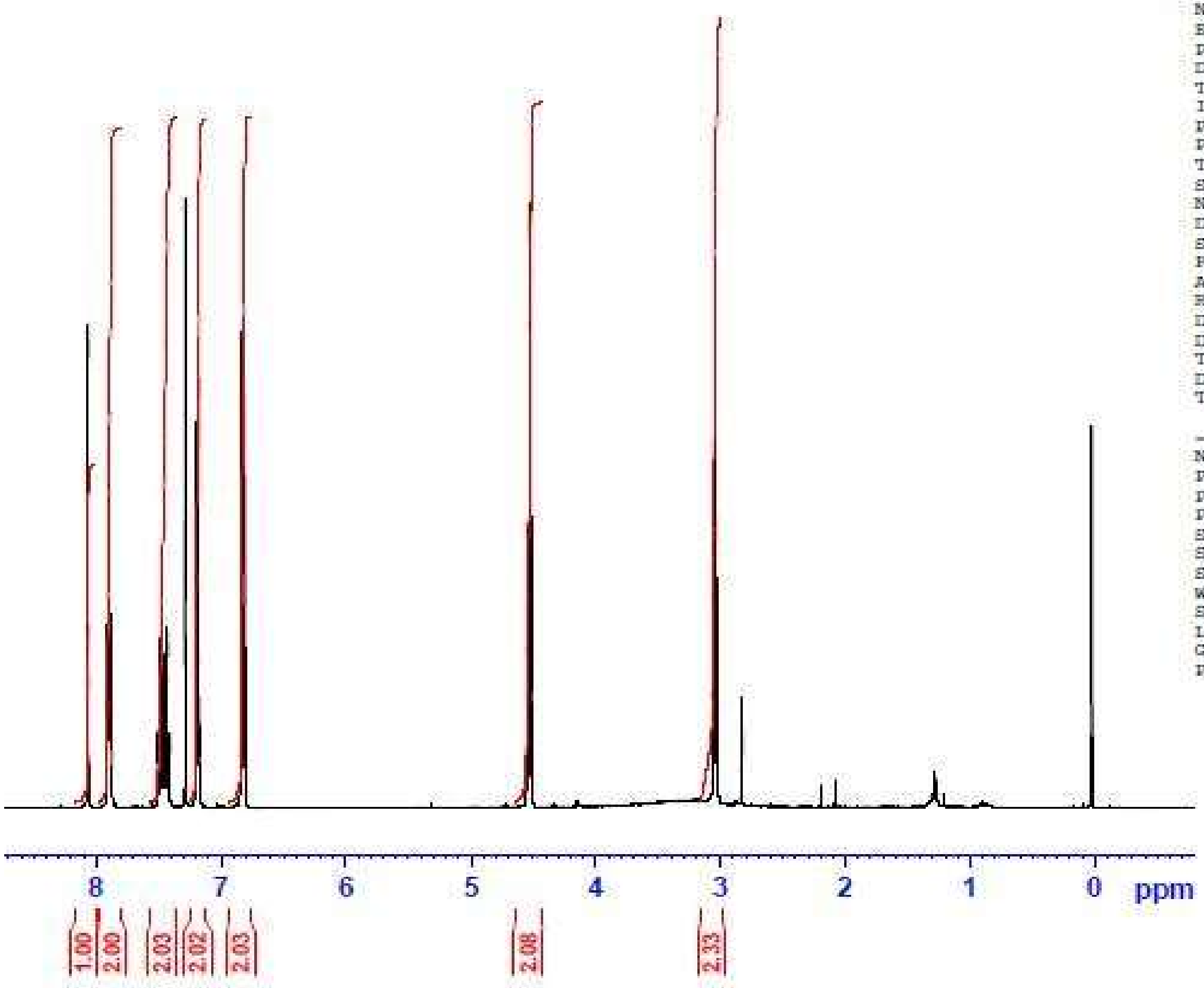

NAME	Audrey Isabelle
EXPNO	14
PROCNO	1
Date_	20190220
Time	15.04
INSTRUM	spect
PROBHD	5 mm PABBO BB-
PULPROG	zg30
TD	65536
SOLVENT	CDCl3
NS	16
DS	2
SWH	8223.685 Hz
FIDRES	0.125483 Hz
AQ	3.9846387 sec
RG	128
DW	60.800 usec
DE	6.50 usec
TE	298.0 K
D1	1.00000000 sec
TD0	1
======== CHANNEL f1 ========	
NUC1	1H
P1	14.07 usec
PL1	0.30 dB
PL1W	11.25229836 W
SFO1	400.1324710 MHz
SI	32768
SF	400.1300000 MHz
WDW	EM
SSB	0
LB	0.30 Hz
GB	0
PC	1.00

^{13}C NMR

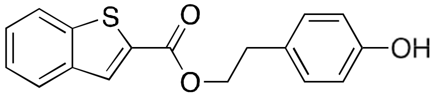
S
O
O
OH

9

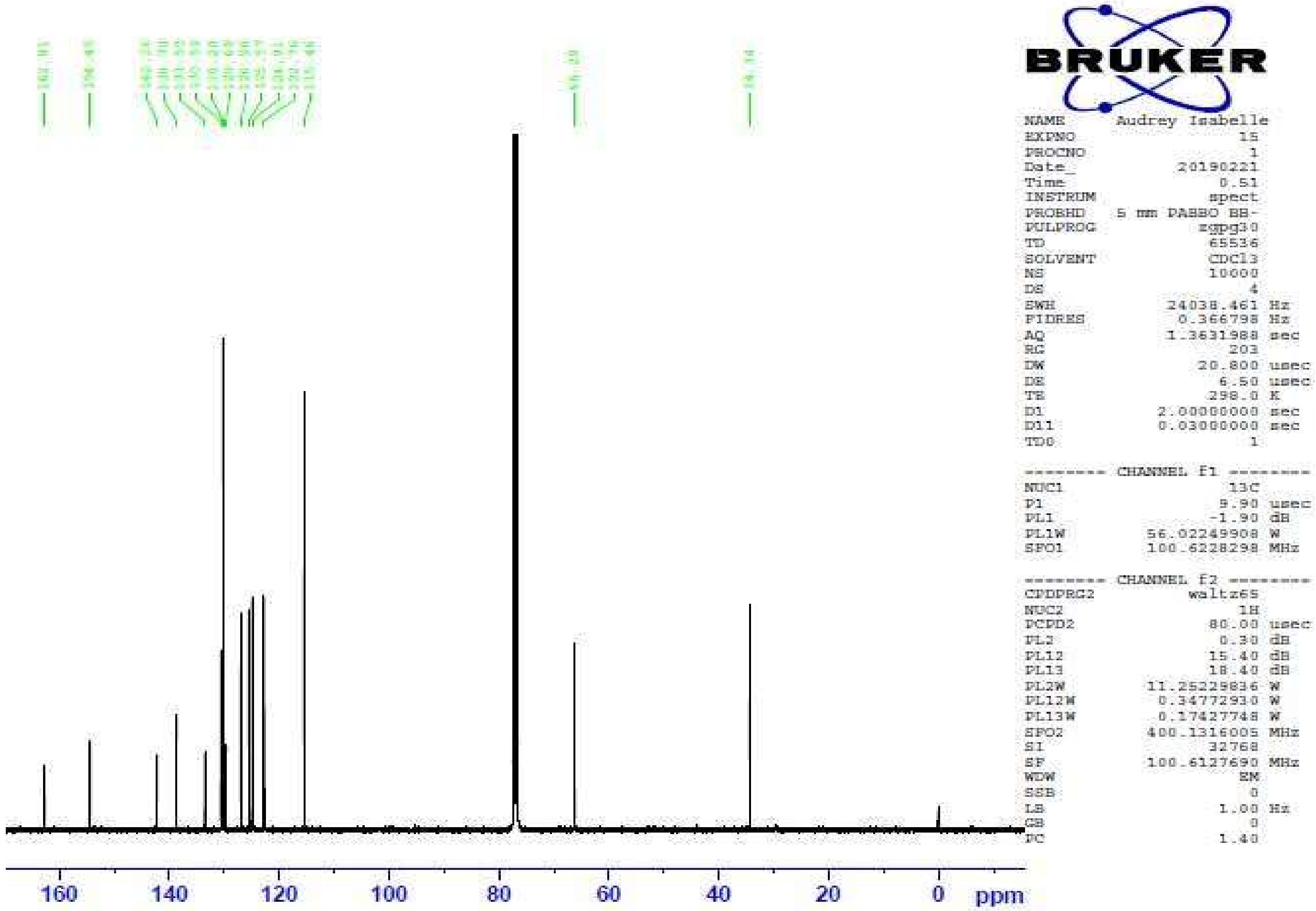
160
140
120
100
80
60
40
20
0
ppm
BRUKER
NAME Audrey Isabelle
EXPNO 15
PROCNO 1
Date_ 20190221
Time 0.51
INSTRUM spect
PROBHD 5 mm PABBO BB-
PULPROG zgpg30
TD 65536
SOLVENT CDCl3
NS 10000
DS 4
SWH 24038.461 Hz
FIDRES 0.366798 Hz
AQ 1.3631988 sec
RG 203
DW 20.800 usec
DE 6.50 usec
TE 298.0 K
D1 2.00000000 sec
D11 0.03000000 sec
TD0 1
======== CHANNEL f1 ========
NUC1 13C
P1 9.90 usec
PL1 -1.90 dB
PL1W 56.02249908 W
SFO1 100.6228298 MHz
======== CHANNEL f2 ========
CPDPRG2 waltz65
NUC2 1H
PCPD2 80.00 usec
PL2 0.30 dB
PL12 15.40 dB
PL13 18.40 dB
PL2W 11.25229836 W
PL12W 0.34772930 W
PL13W 0.17427748 W
SFO2 400.1316005 MHz
SI 32768
SF 100.6127690 MHz
WDW EM
SSB 0
LB 1.00 Hz
GB 0
PC 1.40

HRMS

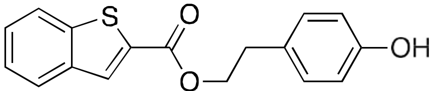

9

Fragmentor Voltage 100 **Collision Energy** 0 **Ionization Mode** Esi

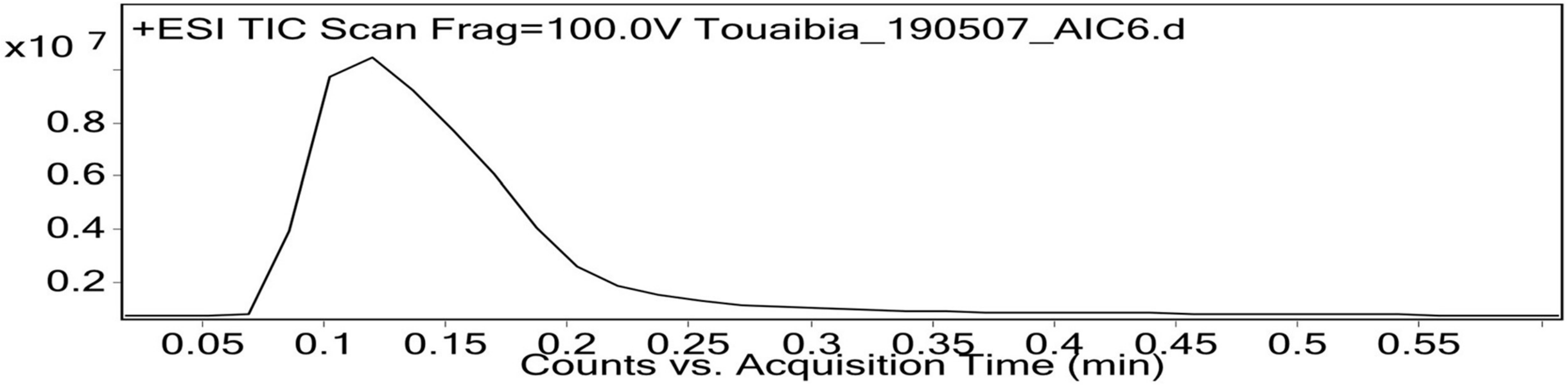

User Spectra

Fragmentor Voltage	Collision Energy	Ionization Mode
100	0	Esi

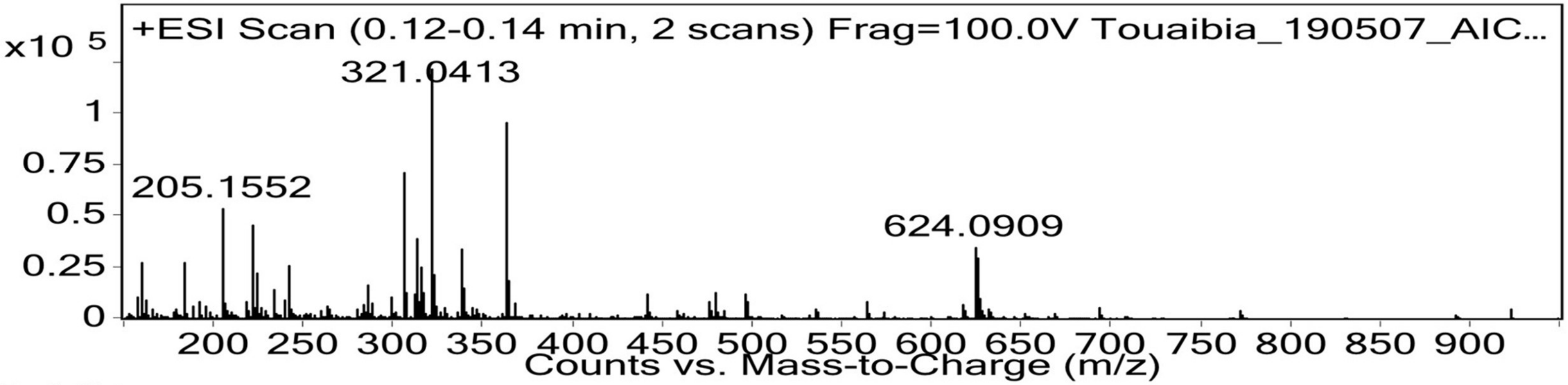

Peak List

^{1}H NMR

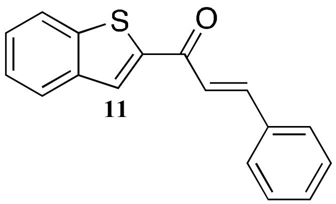

11

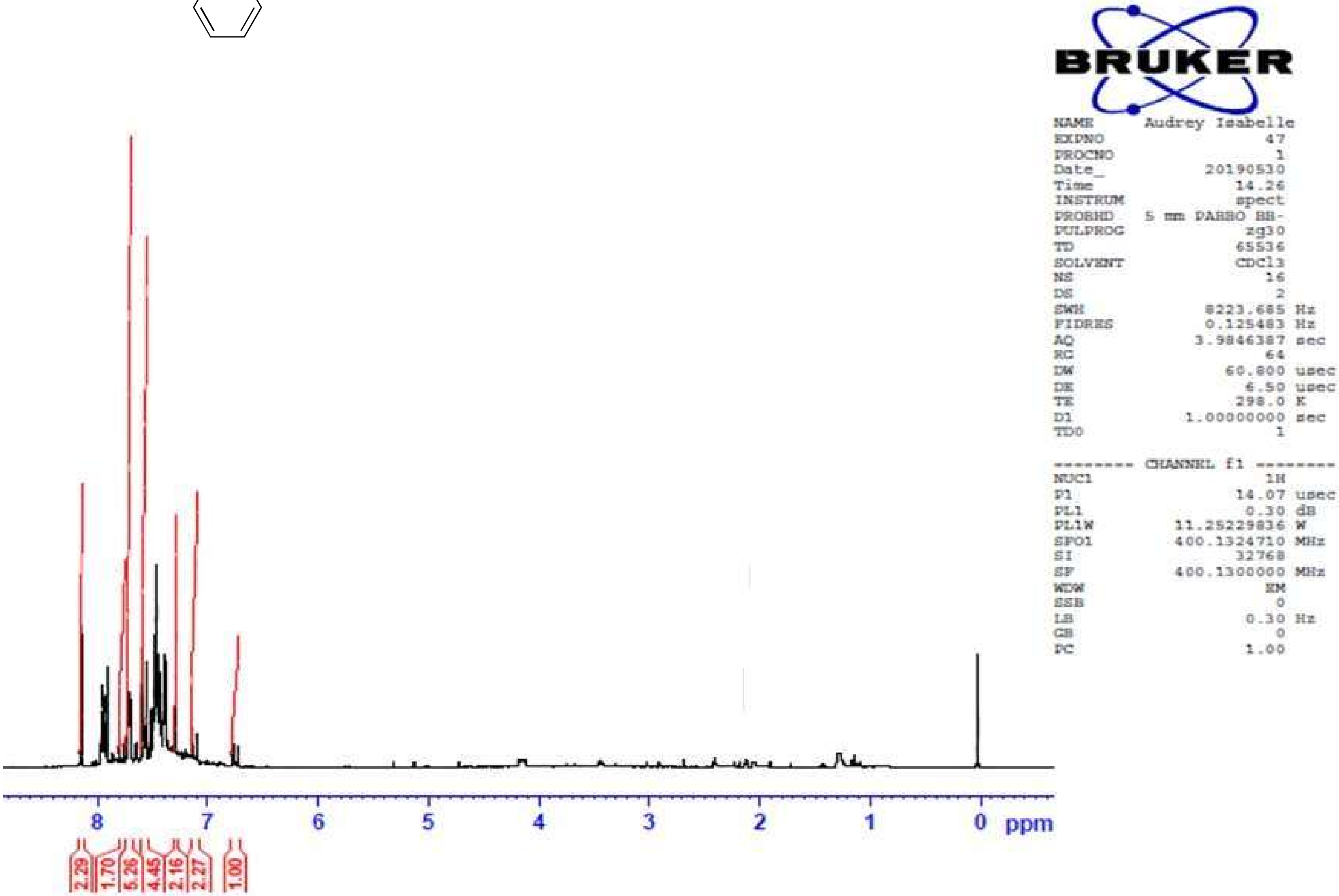
BRUKER
NAME Audrey Isabelle
EXPNO 47
PROCNO 1
Date_ 20190530
Time 14.26
INSTRUM spect
PROBHD 5 mm PABBO BB-
PULPROG zg30
TD 65536
SOLVENT CDCl3
NS 16
DS 2
SWH 8223.685 Hz
FIDRES 0.125483 Hz
AQ 3.9846387 sec
RG 64
DW 60.800 usec
DE 6.50 usec
TE 298.0 K
D1 1.00000000 sec
TD0 1
======== CHANNEL f1 ========
NUC1 1H
P1 14.07 usec
PL1 0.30 dB
PL1W 11.25229836 W
SFO1 400.1324710 MHz
SI 32768
SF 400.1300000 MHz
WDW EM
SSB 0
LB 0.30 Hz
GB 0
PC 1.00
8
7
6
5
4
3
2
1
0
ppm
2.29
1.70
5.26
4.45
2.16
2.27
1.00

^{13}C NMR

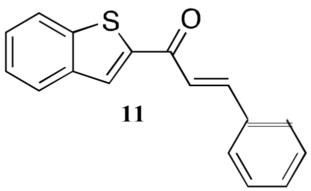

11

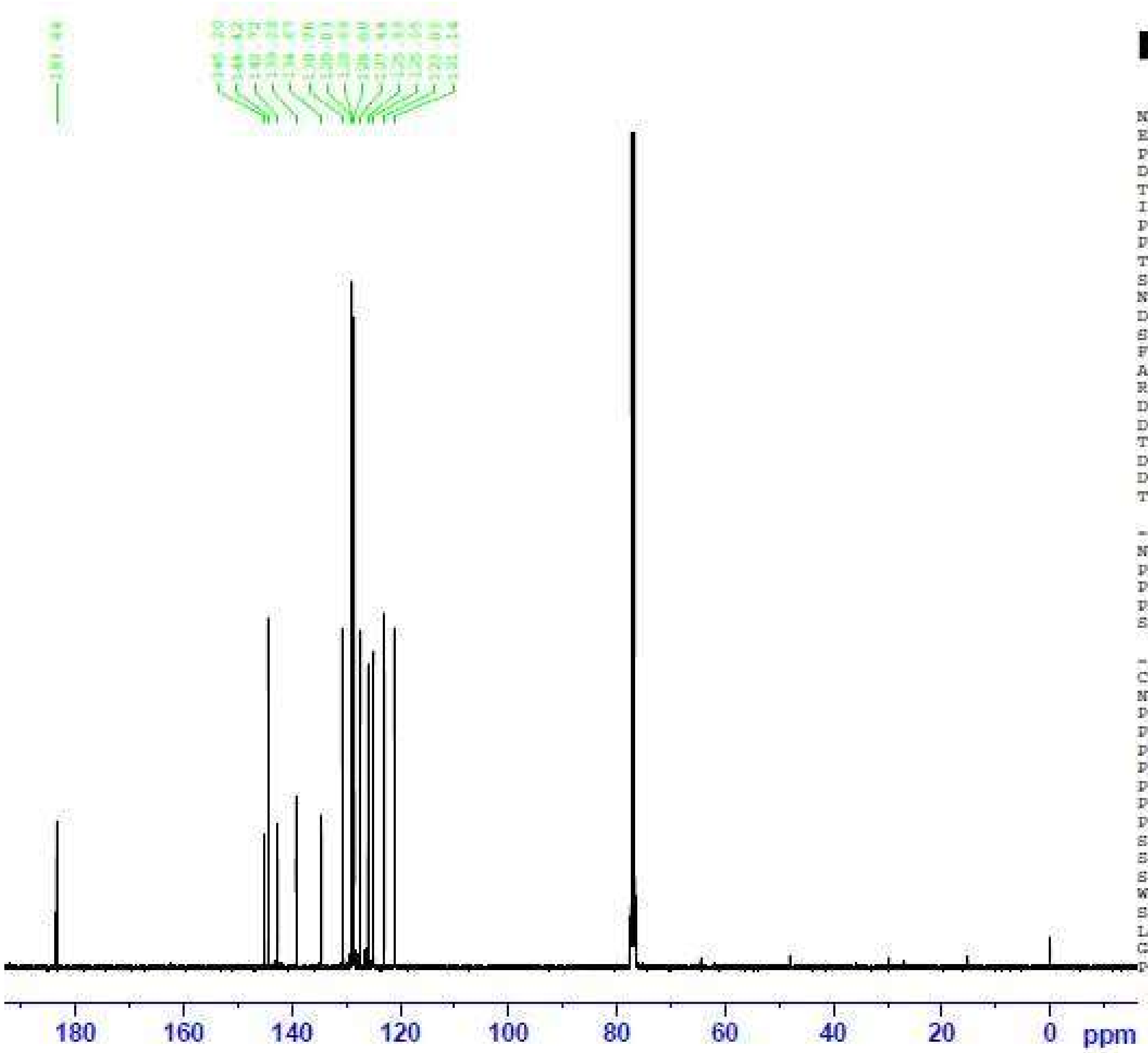

NAME	Audrey Isabelle
EXPNO	52
PROCNO	1
Date_	20190603
Time	23.37
INSTRUM	spect
PROBHD	5 mm PABBO BB-
PULPROG	zgpg30
TD	65536
SOLVENT	CDCl3
NS	10000
DS	4
SWH	24038.461 Hz
FIDRES	0.366798 Hz
AQ	1.3631988 sec
RG	203
DW	20.800 usec
DE	6.50 usec
TE	298.0 K
D1	2.00000000 sec
D11	0.03000000 sec
TD0	1
======== CHANNEL f1 ========	
NUC1	13C
P1	9.90 usec
PL1	-1.90 dB
PL1W	56.02249908 W
SFO1	100.6228298 MHz
======== CHANNEL f2 ========	
CPDPRG2	waltz65
NUC2	1H
PCPD2	80.00 usec
PL2	0.30 dB
PL12	15.40 dB
PL13	18.40 dB
PL2W	11.25229836 W
PL12W	0.34772930 W
PL13W	0.17427748 W
SFO2	400.1316005 MHz
SI	32768
SF	100.6127690 MHz
WDW	EM
SSB	0
LB	1.00 Hz
GB	0
PC	1.40

HRMS

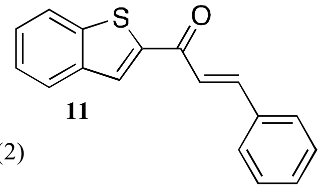

11

AIC 15(2)

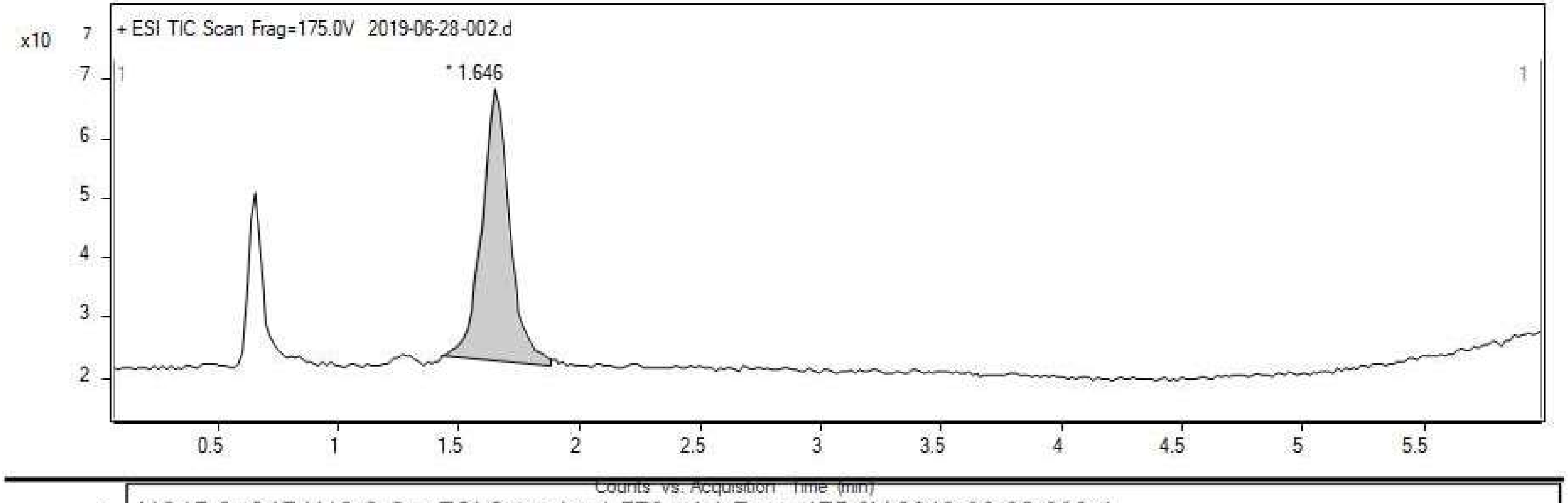

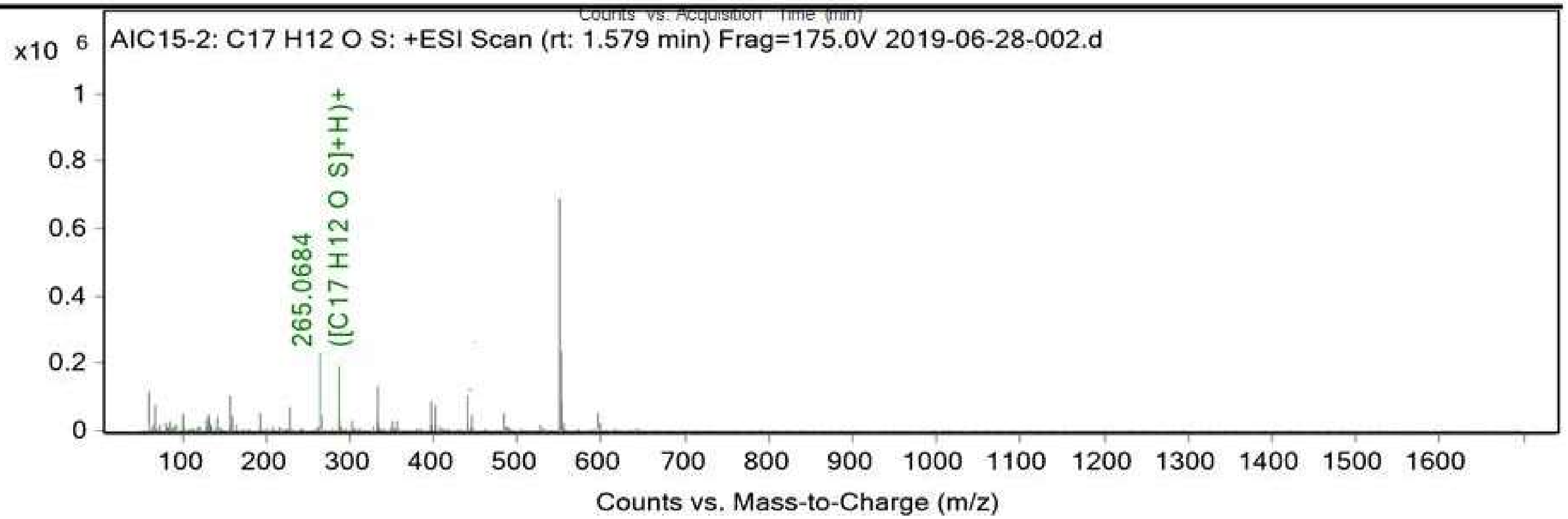

^{1}H NMR

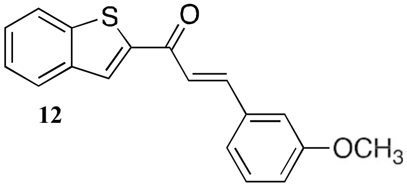

S
O
OCH3

12

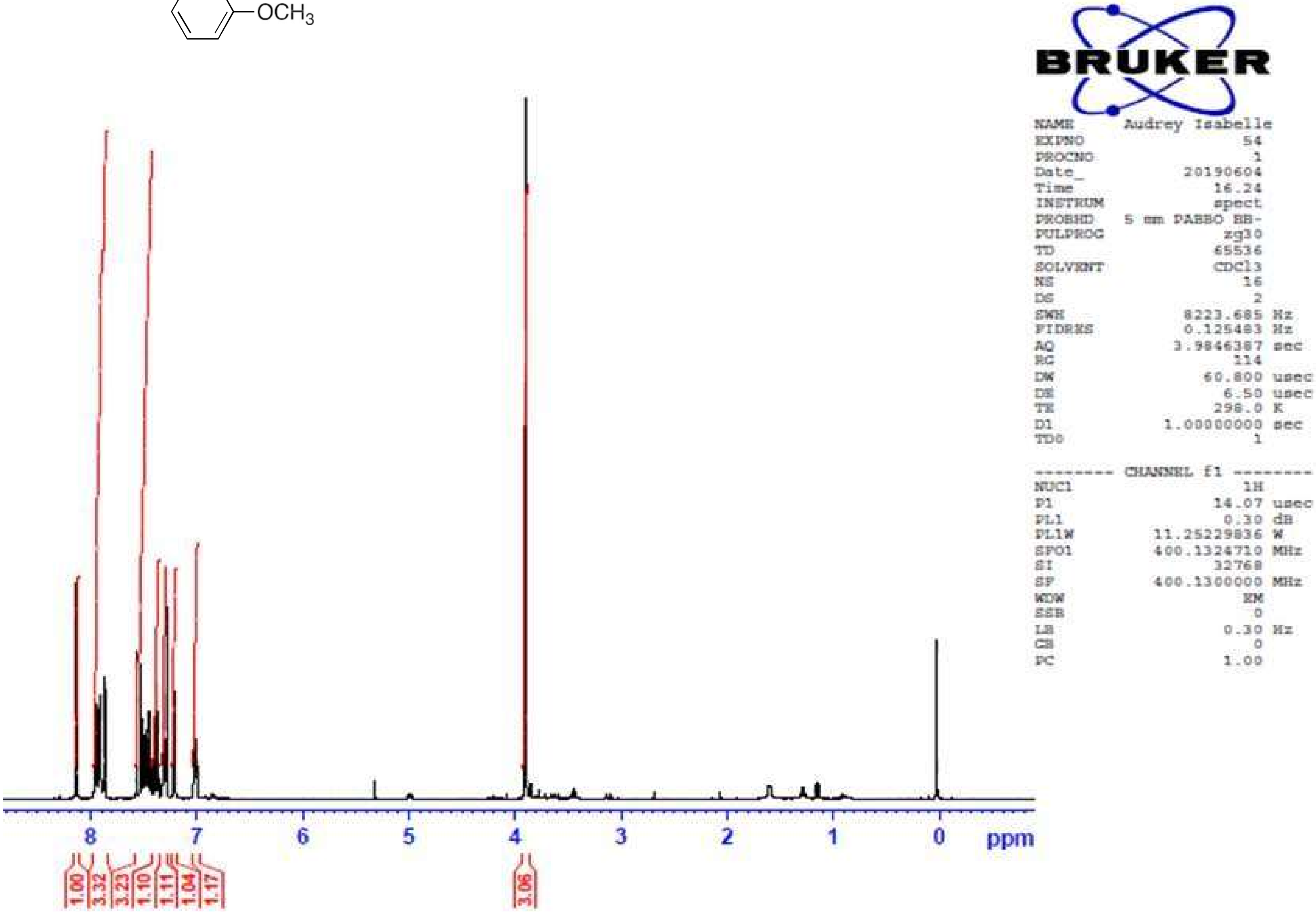

BRUKER
NAME Audrey Isabelle
EXPNO 54
PROCNO 1
Date_ 20190604
Time 16.24
INSTRUM spect
PROBHD 5 mm PABBO BB-
PULPROG zg30
TD 65536
SOLVENT CDCl3
NS 16
DS 2
SWH 8223.685 Hz
FIDRES 0.125483 Hz
AQ 3.9846387 sec
RG 114
DW 60.800 usec
DE 6.50 usec
TE 298.0 K
D1 1.00000000 sec
TD0 1
======== CHANNEL f1 ========
NUC1 1H
P1 14.07 usec
PL1 0.30 dB
PL1W 11.25229836 W
SFO1 400.1324710 MHz
SI 32768
SF 400.1300000 MHz
WDW EM
SSB 0
LB 0.30 Hz
GB 0
PC 1.00
8
7
6
5
4
3
2
1
0
ppm
1.00
3.32
3.23
1.10
1.11
1.04
1.17
3.06

^{13}C NMR

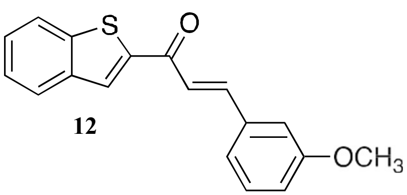

12

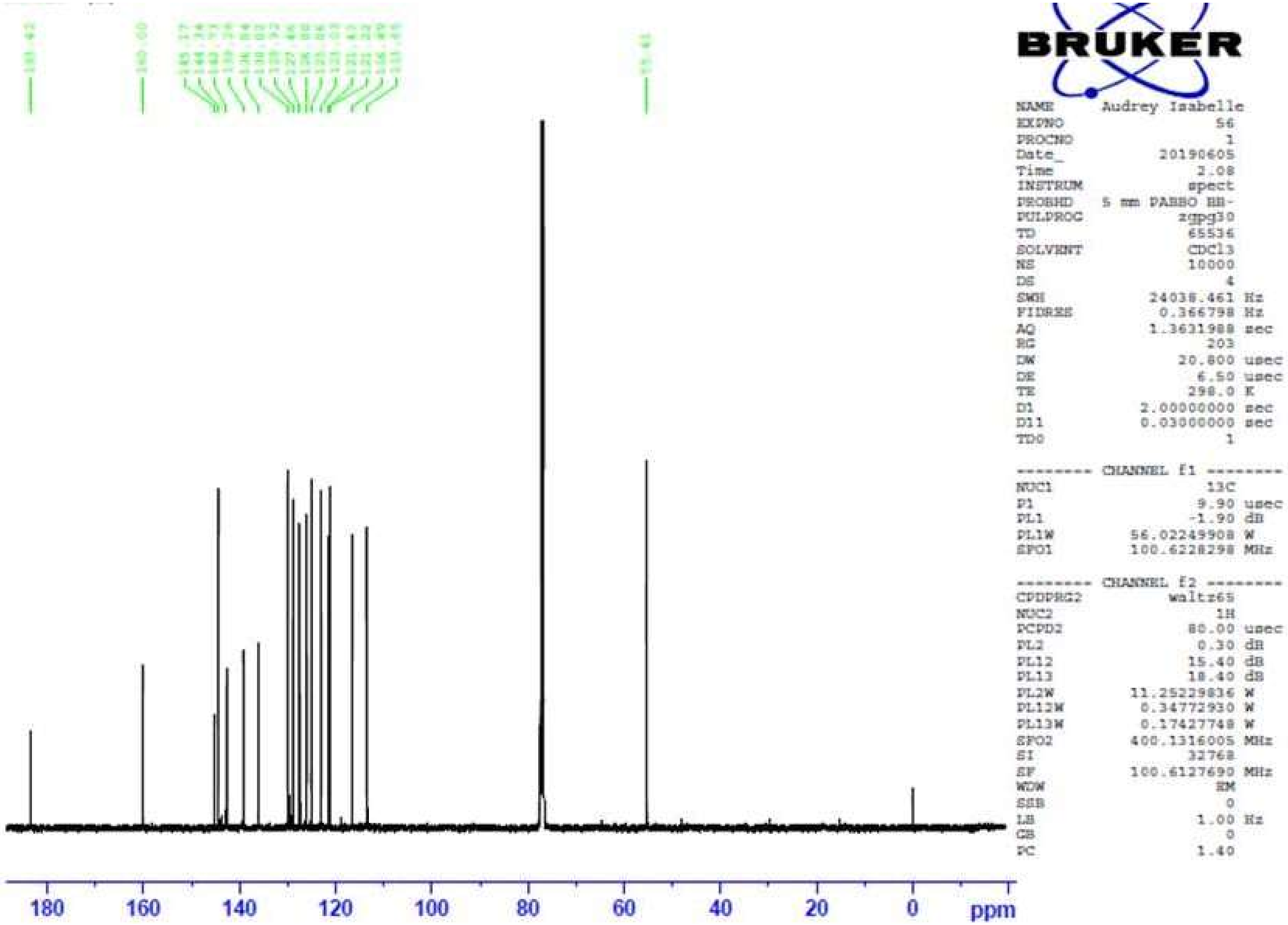
BRUKER
NAME Audrey Isabelle
EXPNO 56
PROCNO 1
Date_ 20190605
Time 2.08
INSTRUM spect
PROBHD 5 mm PABBO BB-
PULPROG zgpg30
TD 65536
SOLVENT CDCl3
NS 10000
DS 4
SWH 24038.461 Hz
FIDRES 0.366798 Hz
AQ 1.3631988 sec
RG 203
DW 20.800 usec
DE 6.50 usec
TE 298.0 K
D1 2.00000000 sec
D11 0.03000000 sec
TD0 1
======== CHANNEL f1 ========
NUC1 13C
P1 9.90 usec
PL1 -1.90 dB
PL1W 56.02249908 W
SFO1 100.6228298 MHz
======== CHANNEL f2 ========
CPDPRG2 waltz65
NUC2 1H
PCPD2 80.00 usec
PL2 0.30 dB
PL12 15.40 dB
PL13 18.40 dB
PL2W 11.25229836 W
PL12W 0.34772930 W
PL13W 0.17427748 W
SFO2 400.1316005 MHz
SI 32768
SF 100.6127690 MHz
WDW EM
SSB 0
LB 1.00 Hz
GB 0
PC 1.40
180
160
140
120
100
80
60
40
20
0
ppm

HRMS

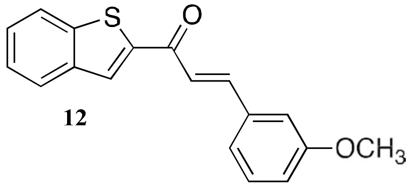
S
O
OCH3

12

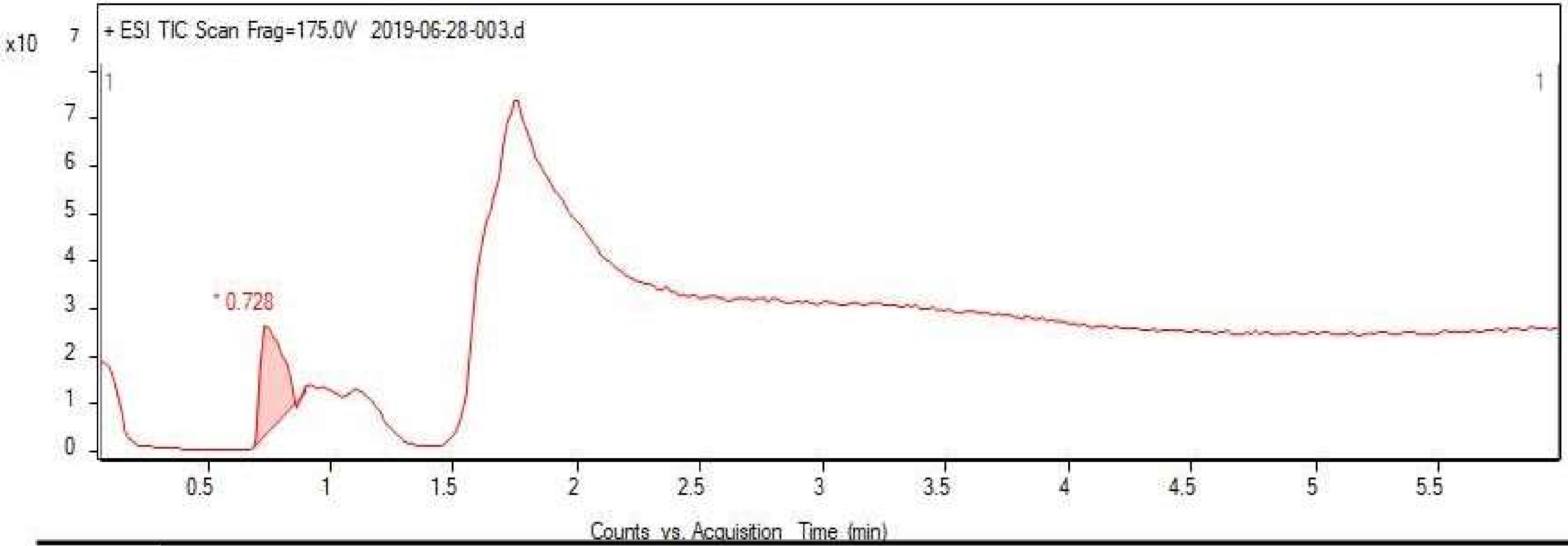
x10 7
+ ESI TIC Scan Frag=175.0V 2019-06-28-003.d
* 0.728
0
1
2
3
4
5
6
7
0.5
1
1.5
2
2.5
3
3.5
4
4.5
5
5.5
Counts vs. Acquisition Time (min)

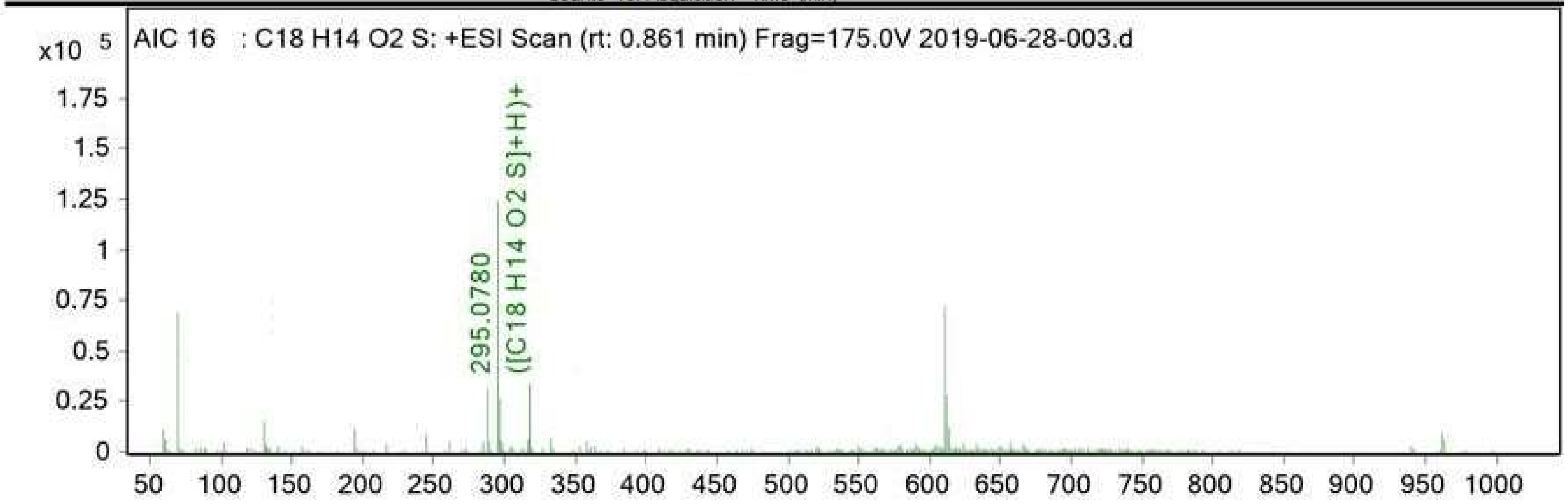
x10 5
AIC 16 : C18 H14 O2 S: +ESI Scan (rt: 0.861 min) Frag=175.0V 2019-06-28-003.d
295.0780
([C18 H14 O2 S]+H)+
0
0.25
0.5
0.75
1
1.25
1.5
1.75
50
100
150
200
250
300
350
400
450
500
550
600
650
700
750
800
850
900
950
1000

^{1}H NMR

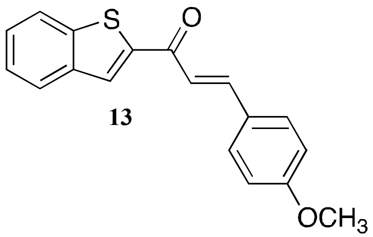

13

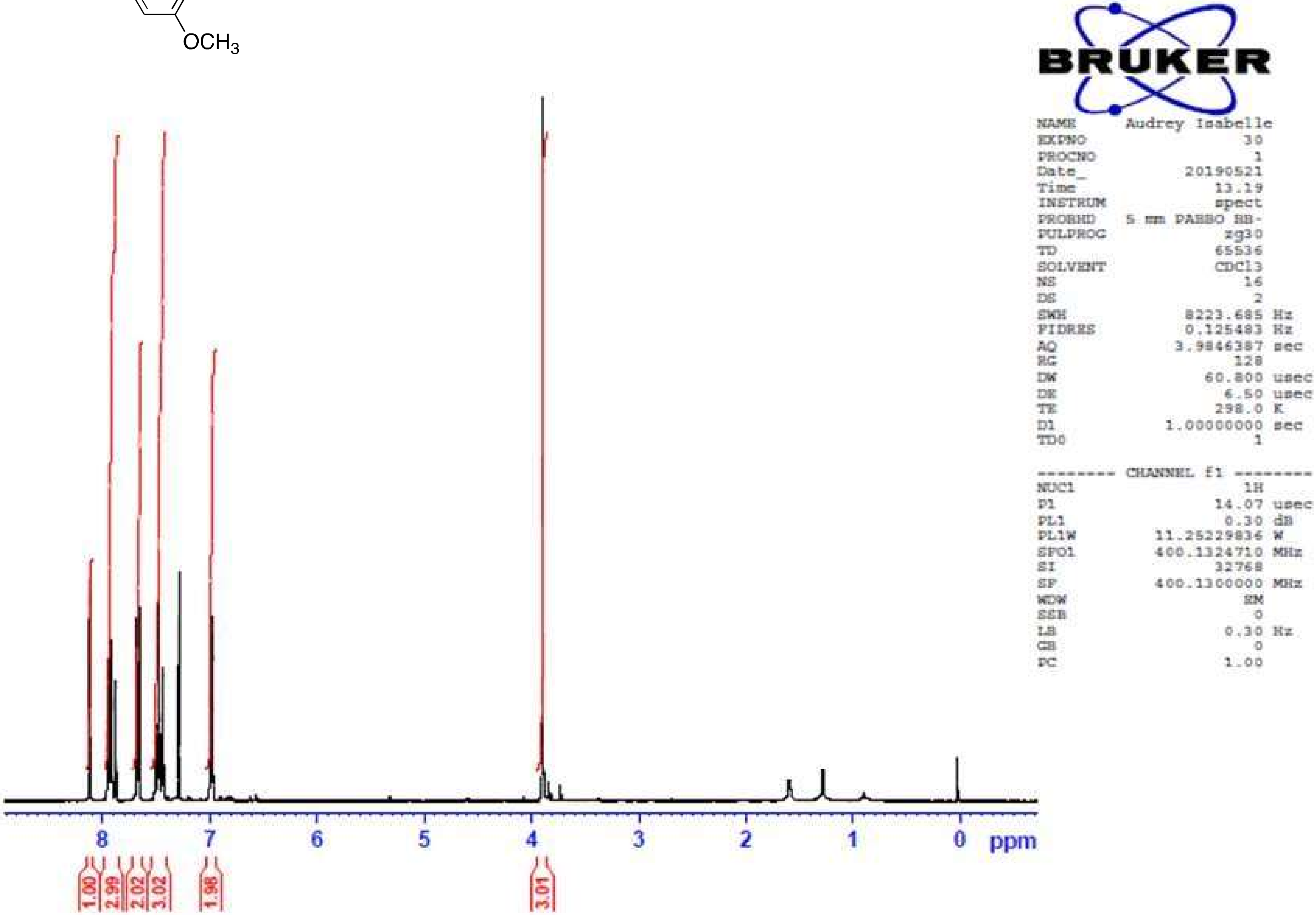
BRUKER
NAME Audrey Isabelle
EXPNO 30
PROCNO 1
Date_ 20190521
Time 13.19
INSTRUM spect
PROBHD 5 mm PABBO BB-
PULPROG zg30
TD 65536
SOLVENT CDCl3
NS 16
DS 2
SWH 8223.685 Hz
FIDRES 0.125483 Hz
AQ 3.9846387 sec
RG 128
DW 60.800 usec
DE 6.50 usec
TE 298.0 K
D1 1.00000000 sec
TD0 1
======== CHANNEL f1 ========
NUC1 1H
P1 14.07 usec
PL1 0.30 dB
PL1W 11.25229836 W
SFO1 400.1324710 MHz
SI 32768
SF 400.1300000 MHz
WDW EM
SSB 0
LB 0.30 Hz
GB 0
PC 1.00
8
7
6
5
4
3
2
1
0
ppm
1.00
2.99
2.02
3.02
1.98
3.01

^{13}C NMR

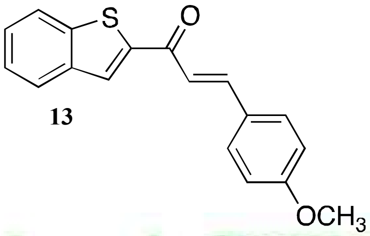
S
O
OCH3

13

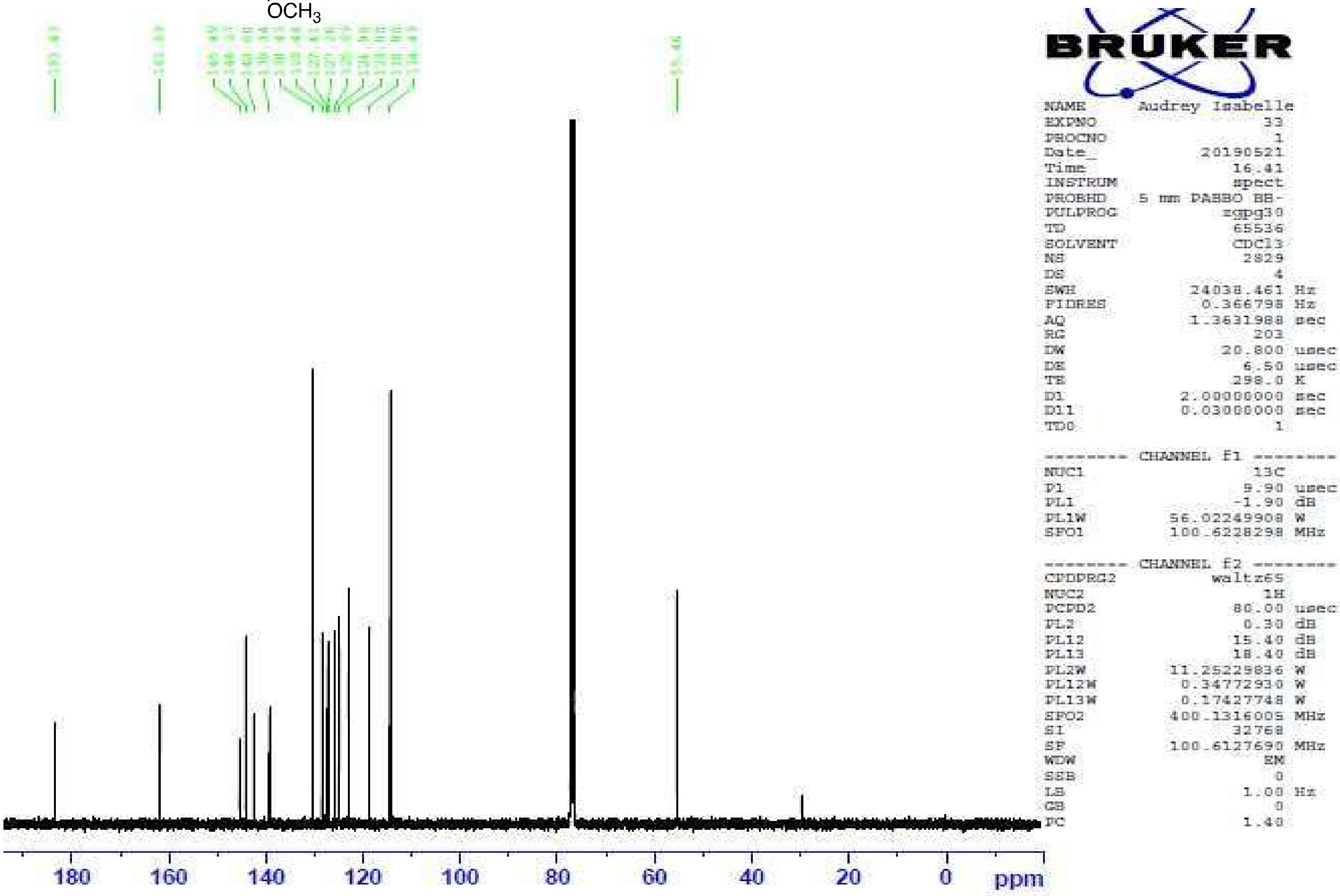
BRUKER
NAME Audrey Issabelle
EXPNO 33
PROCNO 1
Date_ 20190521
Time 16.41
INSTRUM spect
PROBHD 5 mm PABBO BB-
PULPROG zgpg30
TD 65536
SOLVENT CDCl3
NS 2829
DS 4
SWH 24038.461 Hz
FIDRES 0.366798 Hz
AQ 1.3631988 sec
RG 203
DW 20.800 usec
DE 6.50 usec
TE 298.0 K
D1 2.00000000 sec
D11 0.03000000 sec
TD0 1
======== CHANNEL f1 ========
NUC1 13C
P1 9.90 usec
PL1 -1.90 dB
PL1W 56.02249908 W
SFO1 100.6228298 MHz
======== CHANNEL f2 ========
CPDPRG2 waltz65
NUC2 1H
PCPD2 80.00 usec
PL2 0.30 dB
PL12 15.40 dB
PL13 18.40 dB
PL2W 11.25229836 W
PL12W 0.34772930 W
PL13W 0.17427748 W
SFO2 400.1316005 MHz
SI 32768
SF 100.6127690 MHz
WDW EM
SSB 0
LB 1.00 Hz
GB 0
PC 1.40
180
160
140
120
100
80
60
40
20
0
ppm

HRMS

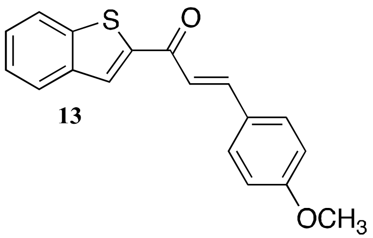
S
O
OCH3

13

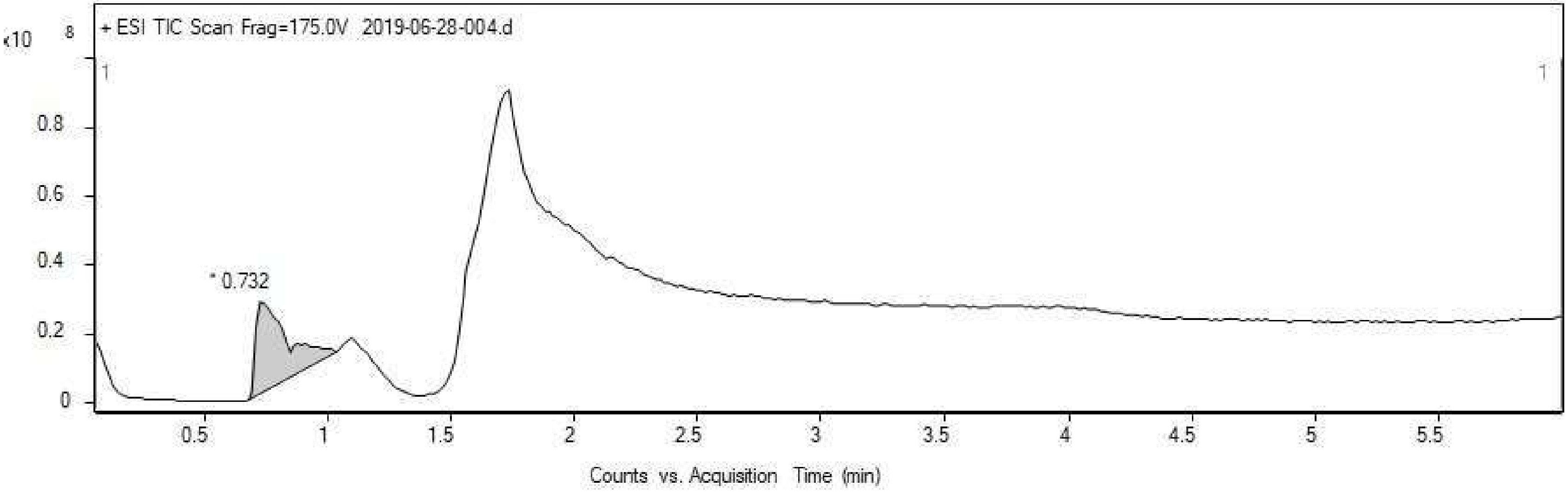
x10 8
+ ESI TIC Scan Frag=175.0V 2019-06-28-004.d
0.8
0.6
0.4
0.2
0
* 0.732
0.5
1
1.5
2
2.5
3
3.5
4
4.5
5
5.5
Counts vs. Acquisition Time (min)

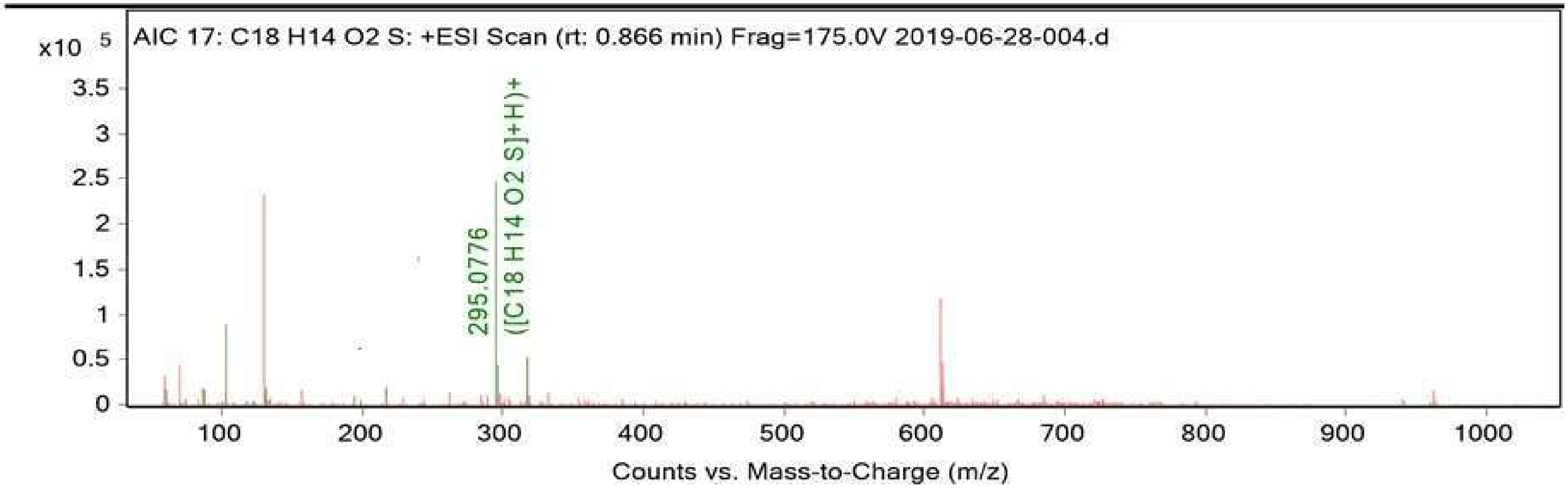
x10 5
AIC 17: C18 H14 O2 S: +ESI Scan (rt: 0.866 min) Frag=175.0V 2019-06-28-004.d
3.5
3
2.5
2
1.5
1
0.5
0
295.0776
([C18 H14 O2 S]+H)+
100
200
300
400
500
600
700
800
900
1000
Counts vs. Mass-to-Charge (m/z)

^{1}H NMR

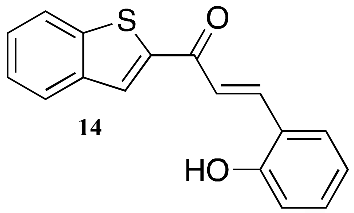

14

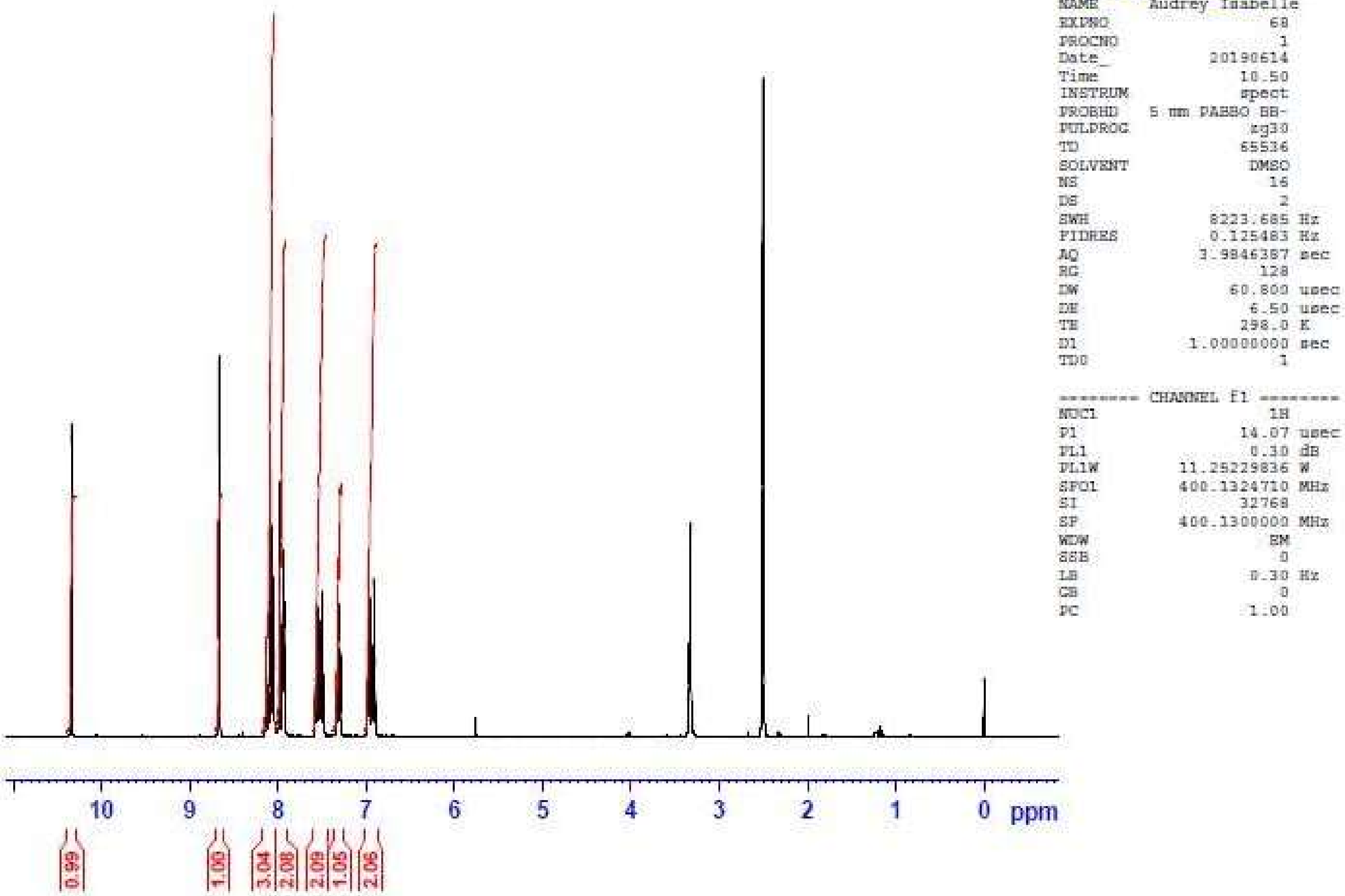

NAME Audrey Isabelle
EXPNO 68
PROCNO 1
Date_ 20190614
Time 10.50
INSTRUM spect
PROBHD 5 mm PABBO BB-
PULPROG zg30
TD 65536
SOLVENT DMSO
NS 16
DS 2
SWH 8223.685 Hz
FIDRES 0.125483 Hz
AQ 3.9846387 sec
RG 128
DW 60.800 usec
DE 6.50 usec
TE 298.0 K
D1 1.00000000 sec
TD0 1
======== CHANNEL f1 ========
NUC1 1H
P1 14.07 usec
PL1 0.30 dB
PL1W 11.25229836 W
SFO1 400.1324710 MHz
SI 32768
SF 400.1300000 MHz
WDW EM
SSB 0
LB 0.30 Hz
GB 0
PC 1.00
10
9
8
7
6
5
4
3
2
1
0
ppm
0.99
1.00
3.04
2.08
2.09
1.05
2.06

^{13}C NMR

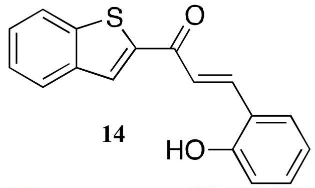

14

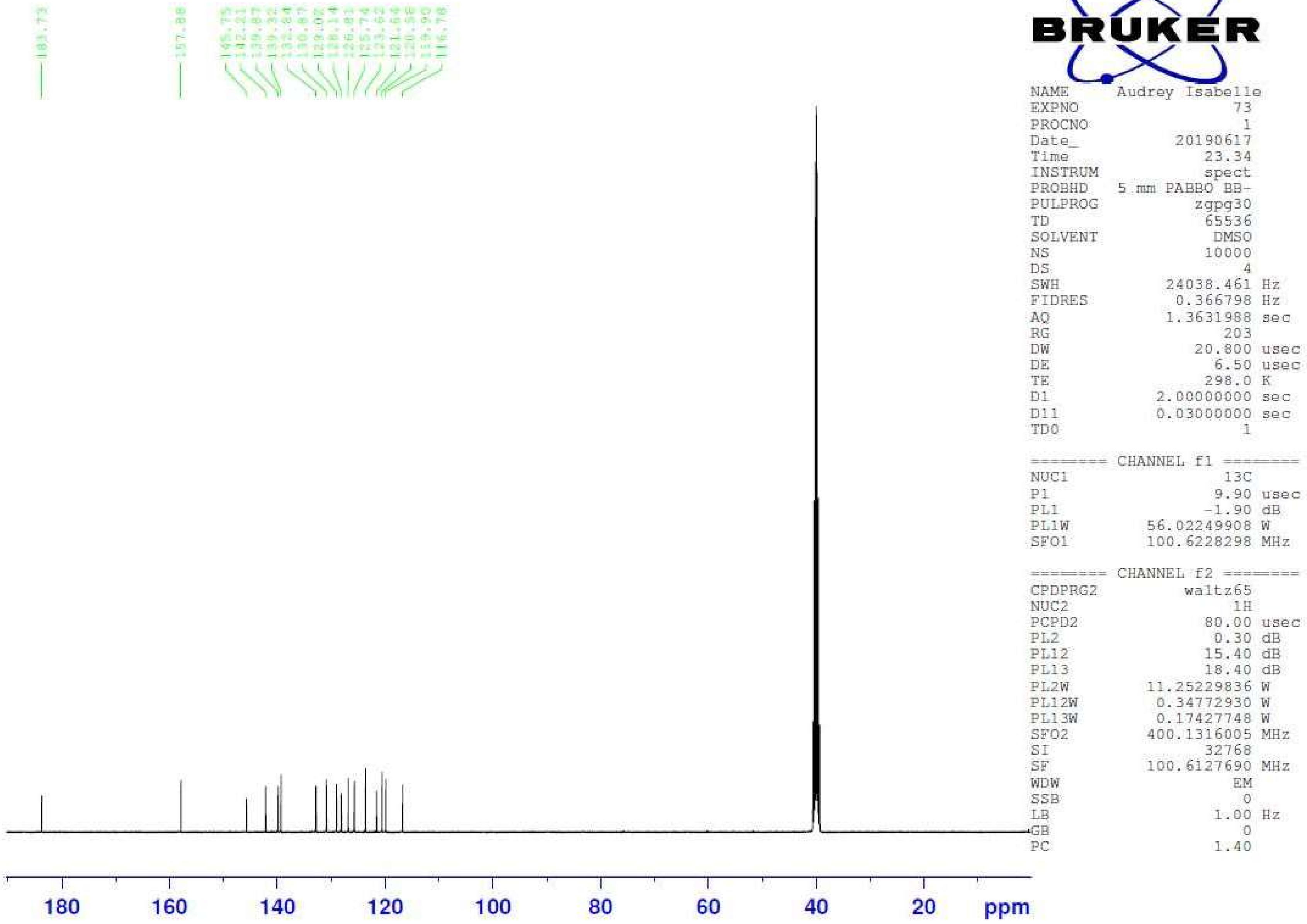
183.73
157.88
145.75
142.21
139.87
139.32
132.84
130.87
129.02
128.14
126.81
125.74
123.62
121.64
120.56
119.90
116.78
180
160
140
120
100
80
60
40
20
ppm
BRUKER
NAME Audrey Isabelle
EXPNO 73
PROCNO 1
Date_ 20190617
Time 23.34
INSTRUM spect
PROBHD 5 mm PABBO BB-
PULPROG zgpg30
TD 65536
SOLVENT DMSO
NS 10000
DS 4
SWH 24038.461 Hz
FIDRES 0.366798 Hz
AQ 1.3631988 sec
RG 203
DW 20.800 usec
DE 6.50 usec
TE 298.0 K
D1 2.00000000 sec
D11 0.03000000 sec
TD0 1
======== CHANNEL f1 ========
NUC1 13C
P1 9.90 usec
PL1 -1.90 dB
PL1W 56.02249908 W
SFO1 100.6228298 MHz
======== CHANNEL f2 ========
CPDPRG2 waltz65
NUC2 1H
PCPD2 80.00 usec
PL2 0.30 dB
PL12 15.40 dB
PL13 18.40 dB
PL2W 11.25229836 W
PL12W 0.34772930 W
PL13W 0.17427748 W
SFO2 400.1316005 MHz
SI 32768
SF 100.6127690 MHz
WDW EM
SSB 0
LB 1.00 Hz
GB 0
PC 1.40

HRMS

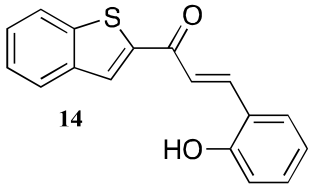
S
O
HO

14

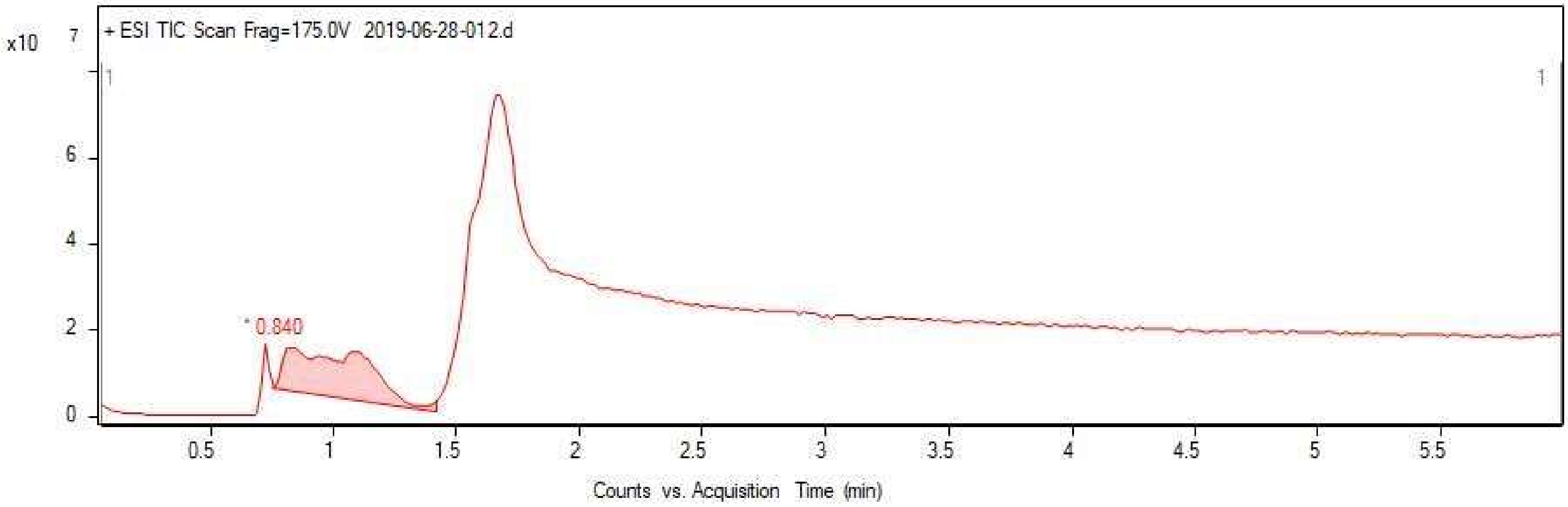
+ ESI TIC Scan Frag=175.0V 2019-06-28-012.d
x10 7
0
2
4
6
* 0.840
0.5
1
1.5
2
2.5
3
3.5
4
4.5
5
5.5
Counts vs. Acquisition Time (min)

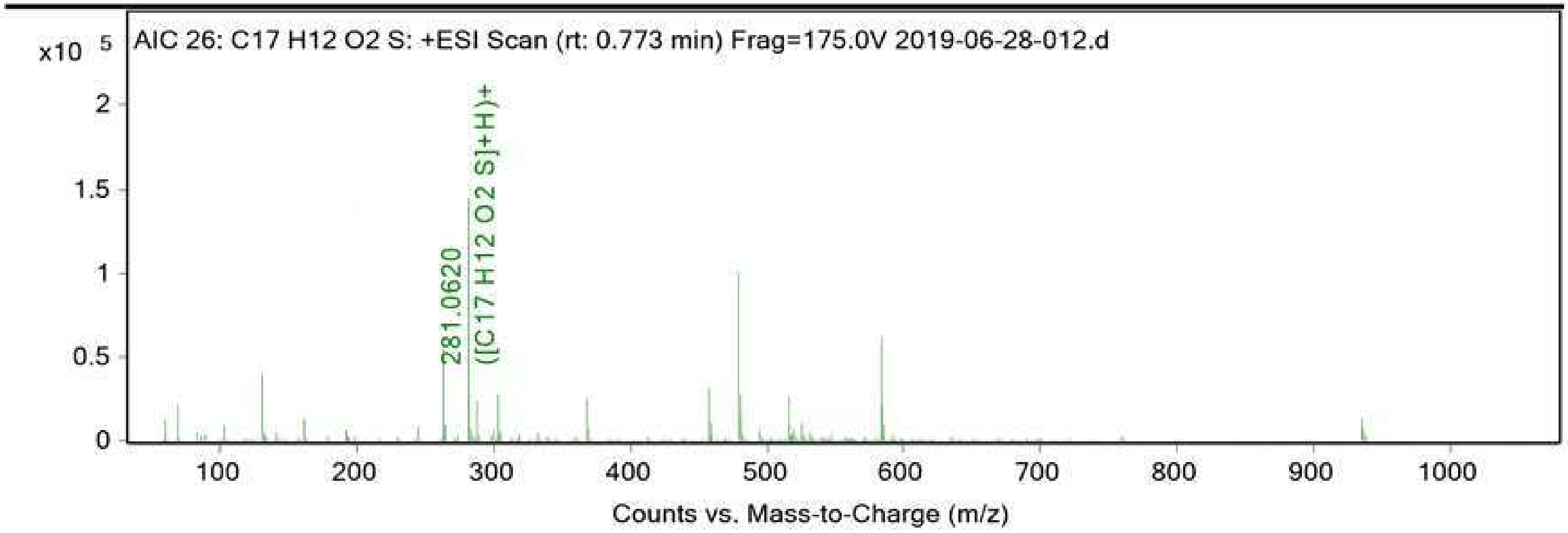
AIC 26: C17 H12 O2 S: +ESI Scan (rt: 0.773 min) Frag=175.0V 2019-06-28-012.d
x10 5
0
0.5
1
1.5
2
281.0620
([C17 H12 O2 S]+H)+
100
200
300
400
500
600
700
800
900
1000
Counts vs. Mass-to-Charge (m/z)

^{1}H NMR

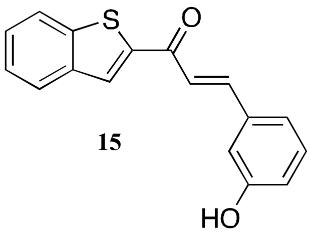

15

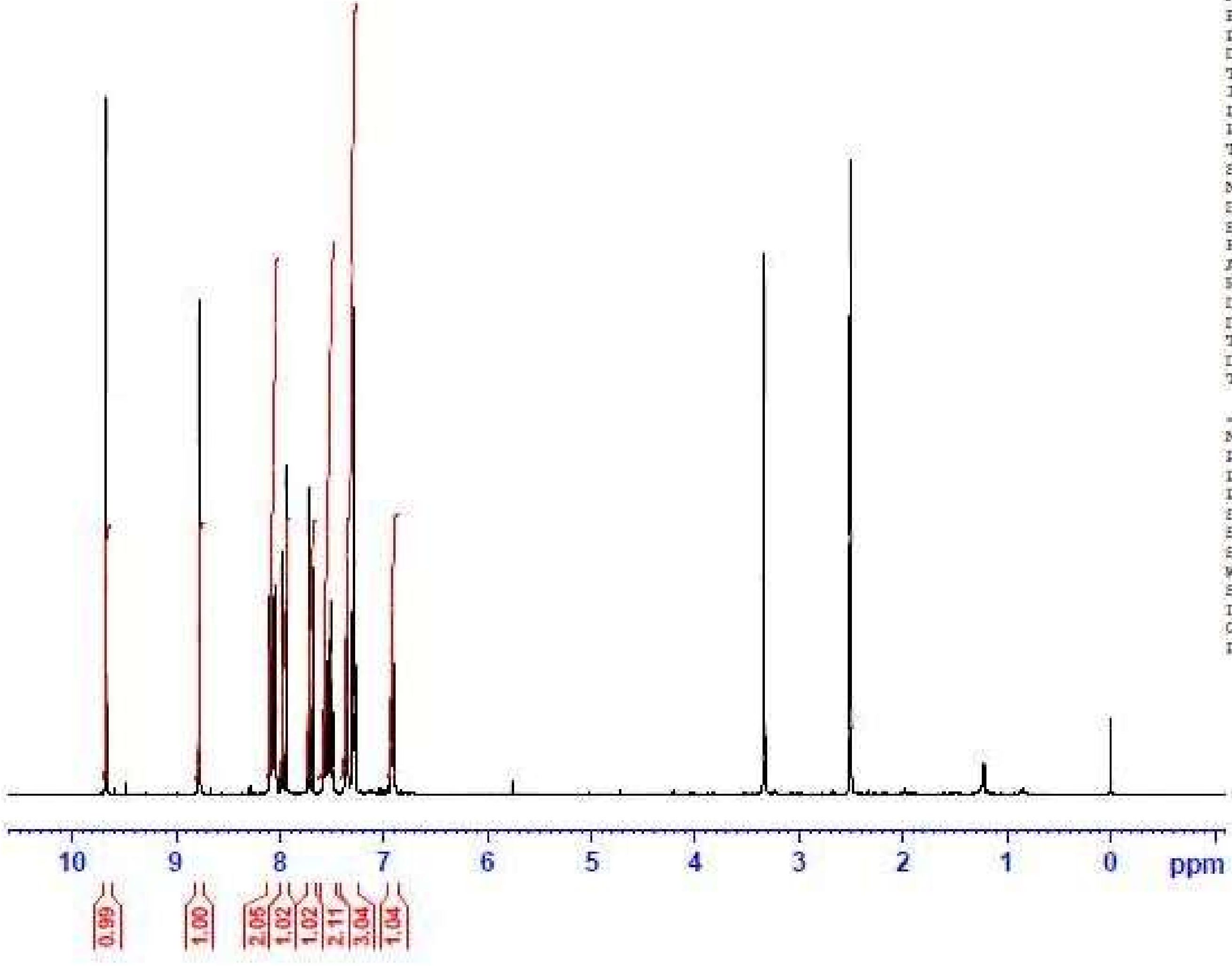

NAME	Audrey Isabelle
EXPNO	70
PROCNO	1
Date_	20190614
Time	15.50
INSTRUM	spect
PROBHD	5 mm PABBO BB-
PULPROG	zg30
TD	65536
SOLVENT	DMSO
NS	16
DS	2
SWH	8223.685 Hz
FIDRES	0.125483 Hz
AQ	3.9846387 sec
RG	114
DW	60.800 usec
DE	6.50 usec
TE	298.0 K
D1	1.00000000 sec
TD0	1
======== CHANNEL f1 ========	
NUC1	1H
P1	14.07 usec
PL1	0.30 dB
PL1W	11.25229836 W
SFO1	400.1324710 MHz
SI	32768
SF	400.1300000 MHz
WDW	EM
SSB	0
LB	0.30 Hz
GB	0
PC	1.00

^{13}C NMR

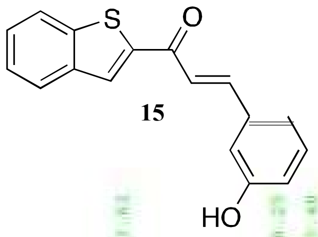

15

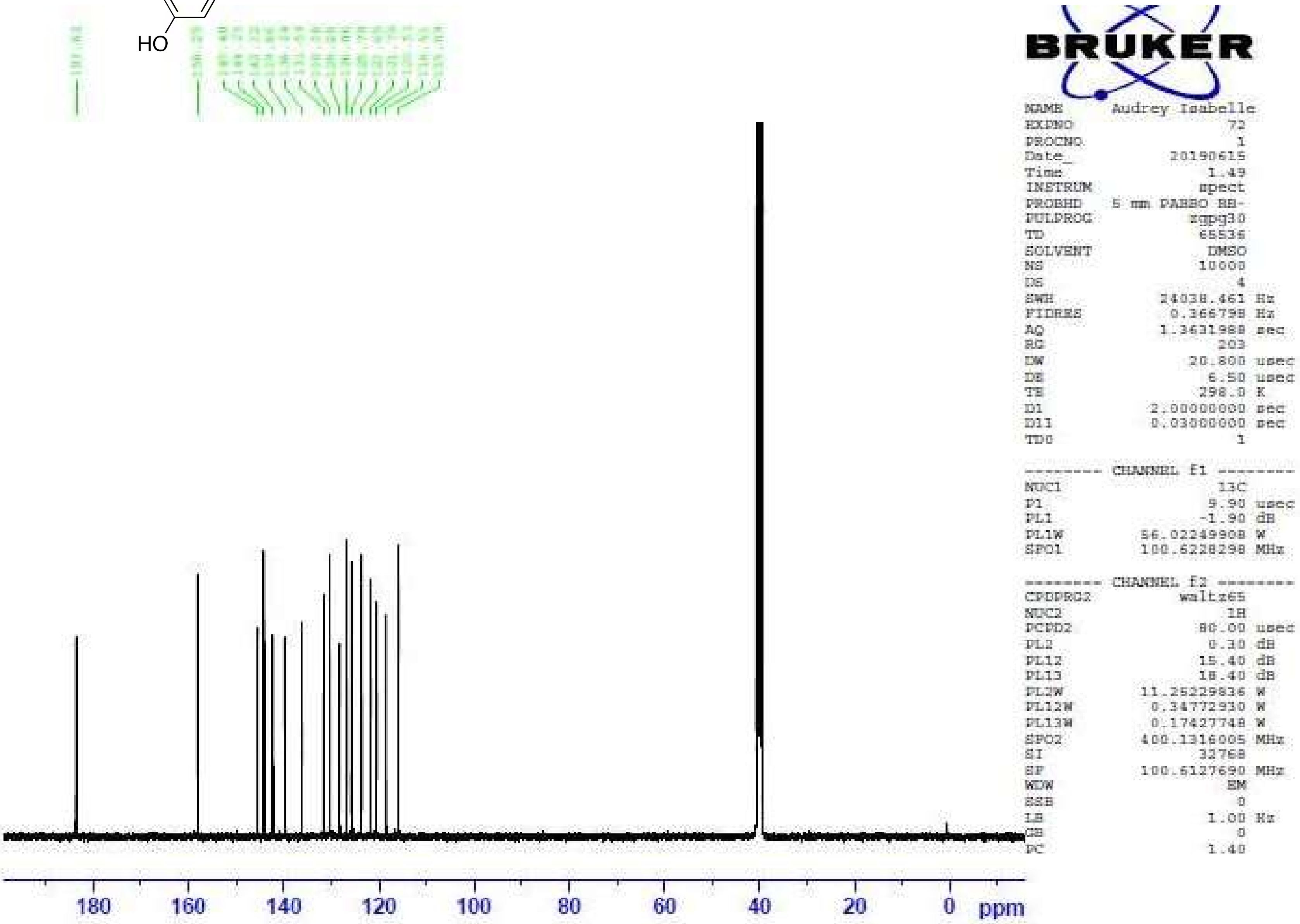

NAME Audrey Isabelle
EXPNO 72
PROCNO 1
Date_ 20190615
Time 1.49
INSTRUM spect
PROBHD 5 mm PABBO BB-
PULPROG zgpg30
TD 65536
SOLVENT DMSO
NS 10000
DS 4
SWH 24038.461 Hz
FIDRES 0.366798 Hz
AQ 1.3631988 sec
RG 203
DW 20.800 usec
DE 6.50 usec
TE 298.0 K
D1 2.00000000 sec
D11 0.03000000 sec
TD0 1
======== CHANNEL f1 ========
NUC1 13C
P1 9.90 usec
PL1 -1.90 dB
PL1W 56.02249908 W
SFO1 100.6228298 MHz
======== CHANNEL f2 ========
CPDPRG2 waltz65
NUC2 1H
PCPD2 80.00 usec
PL2 0.30 dB
PL12 15.40 dB
PL13 18.40 dB
PL2W 11.25229836 W
PL12W 0.34772930 W
PL13W 0.17427748 W
SFO2 400.1316005 MHz
SI 32768
SF 100.6127690 MHz
WDW EM
SSB 0
LB 1.00 Hz
GB 0
PC 1.40
180
160
140
120
100
80
60
40
20
0
ppm

HRMS

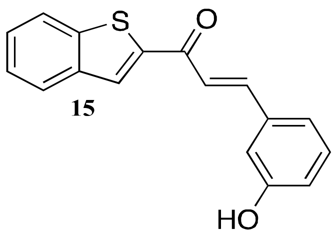
S
O
HO

15

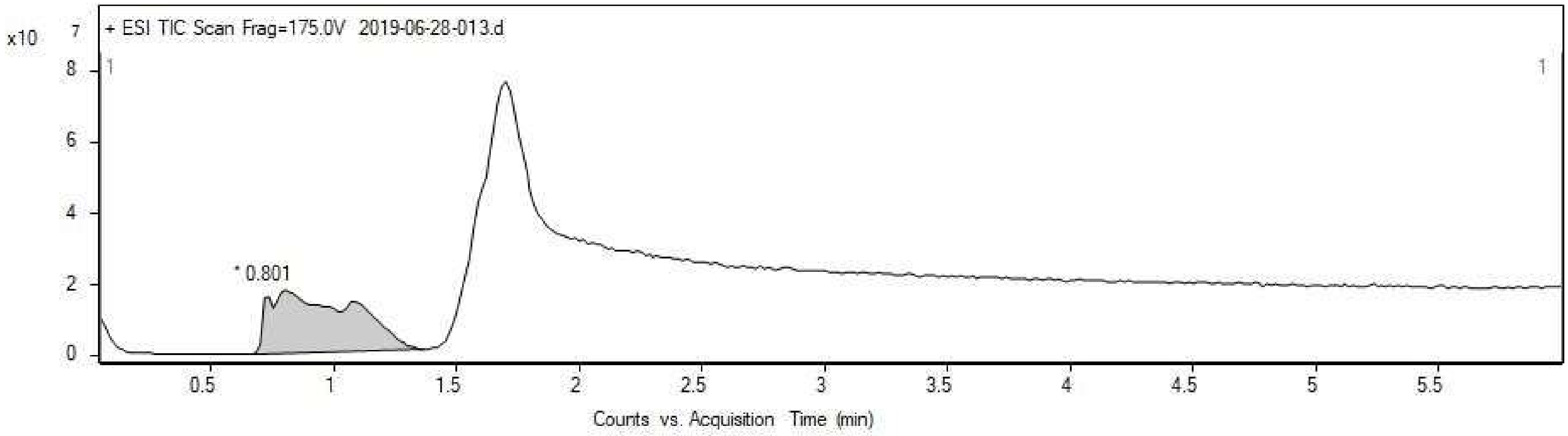
x10 7
+ ESI TIC Scan Frag=175.0V 2019-06-28-013.d
* 0.801
Counts vs. Acquisition Time (min)

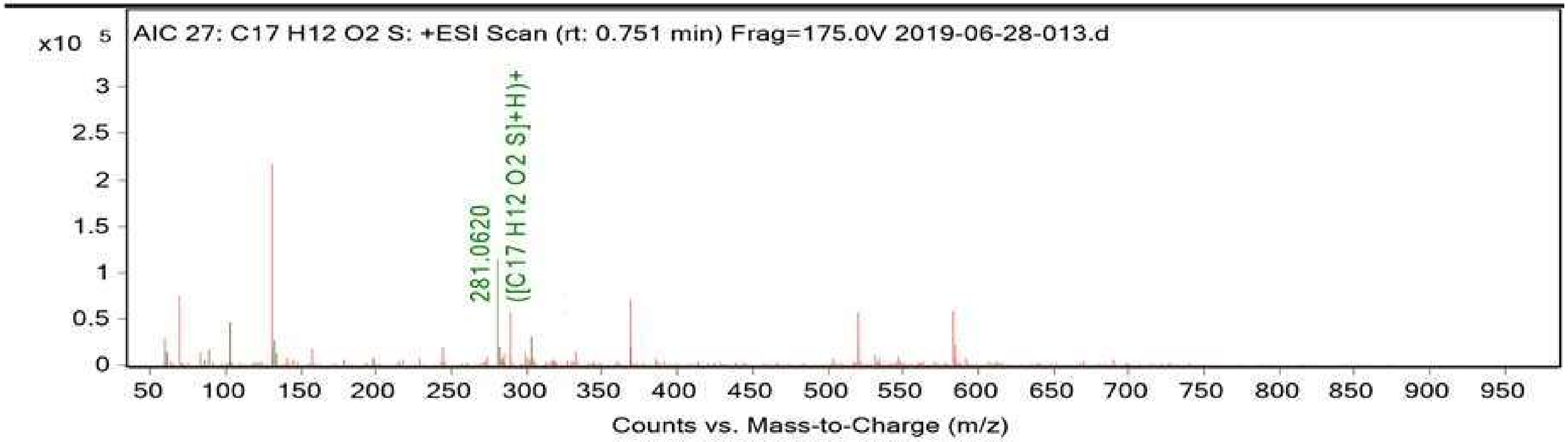
x10 5
AIC 27: C17 H12 O2 S: +ESI Scan (rt: 0.751 min) Frag=175.0V 2019-06-28-013.d
281.0620
([C17 H12 O2 S]+H)+
Counts vs. Mass-to-Charge (m/z)

^{1}H NMR

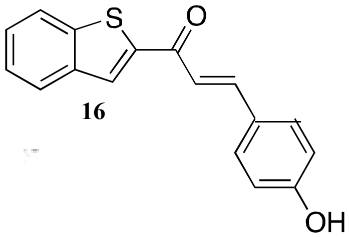

16

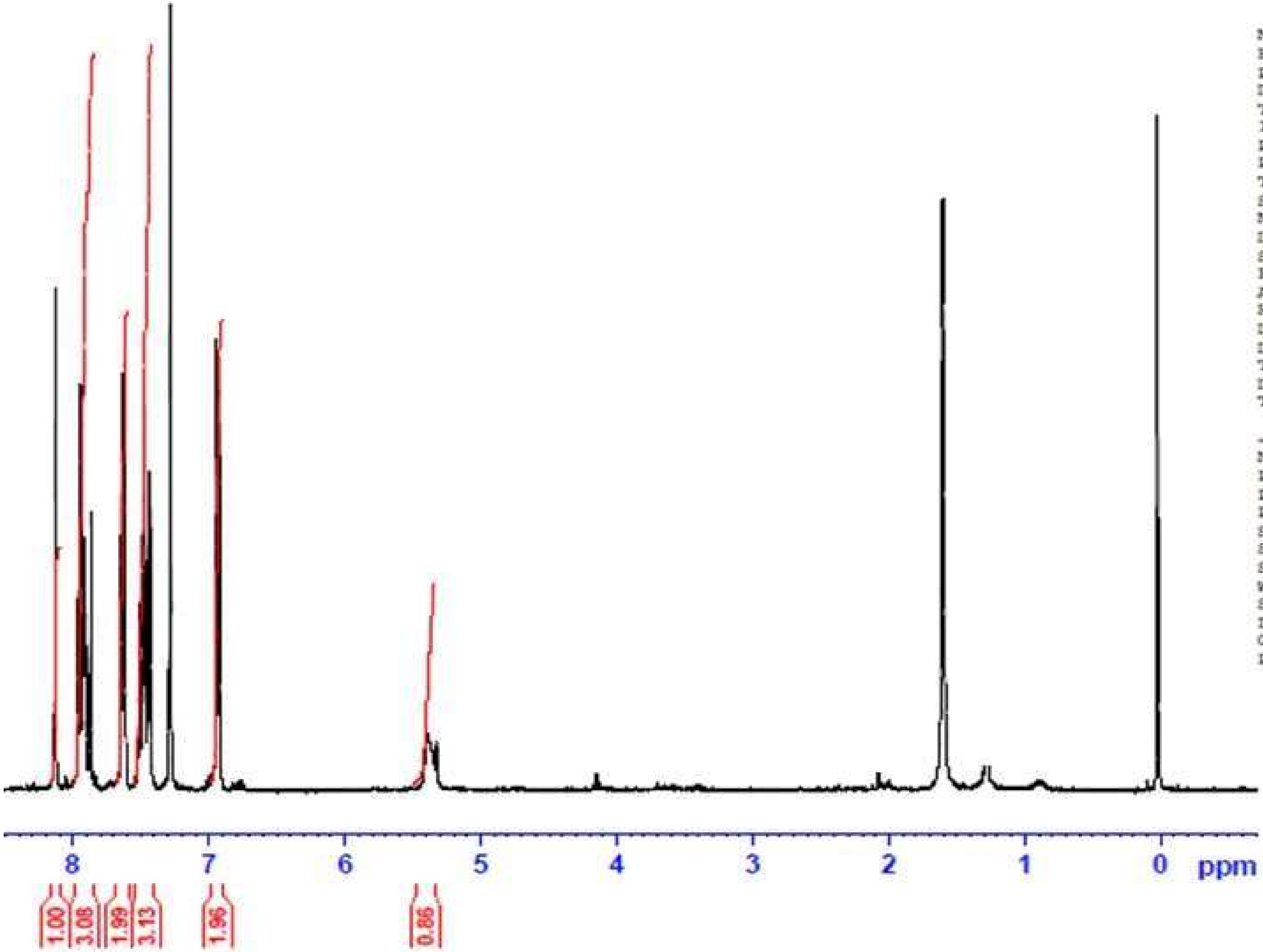

BRUKER

NAME	Audrey Isabelle
EXPNO	16
PROCNO	1
Date_	20190302
Time	11.01
INSTRUM	spect
PROBHD	5 mm PABBO BB-
PULPROG	zg30
TD	65536
SOLVENT	CDCl3
NS	16
DS	2
SWH	8223.685 Hz
FIDRES	0.125483 Hz
AQ	3.9846387 sec
RG	203
DW	60.800 usec
DE	6.50 usec
TE	298.0 K
D1	1.00000000 sec
TD0	1

======== CHANNEL f1 ========

NUC1	1H
P1	14.07 usec
PL1	0.30 dB
PL1W	11.25229836 W
SFO1	400.1324710 MHz
SI	32768
SF	400.1300000 MHz
WDW	EM
SSB	0
LB	0.30 Hz
GB	0
PC	1.00

^{13}C NMR

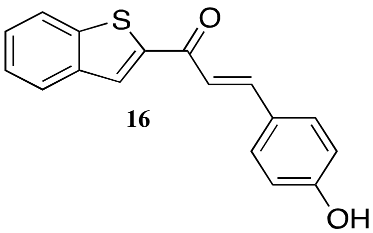

16

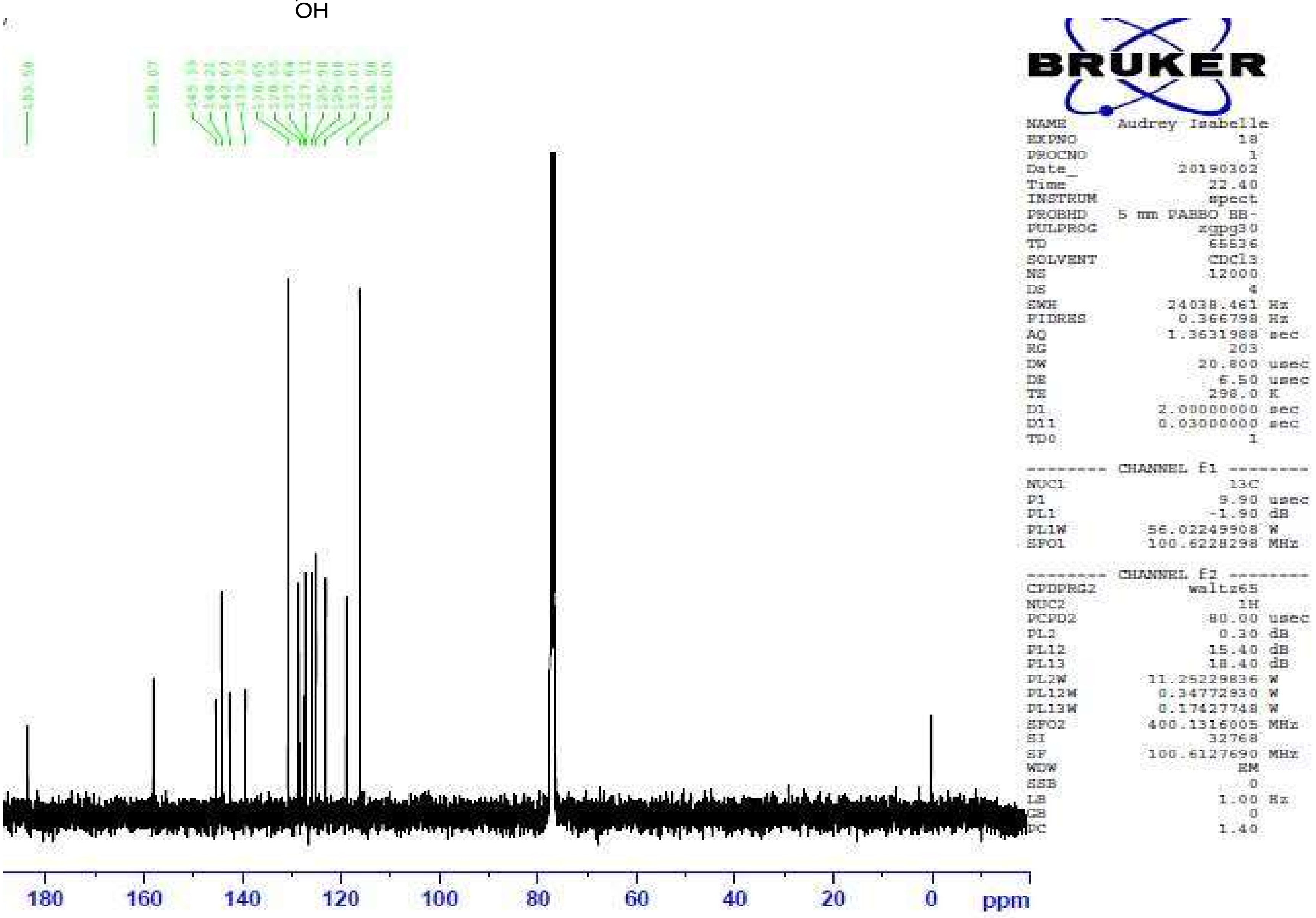

BRUKER
NAME Audrey Isabelle
EXPNO 18
PROCNO 1
Date_ 20190302
Time 22.40
INSTRUM spect
PROBHD 5 mm PABBO BB-
PULPROG zgpg30
TD 65536
SOLVENT CDCl3
NS 12000
DS 4
SWH 24038.461 Hz
FIDRES 0.366798 Hz
AQ 1.3631988 sec
RG 203
DW 20.800 usec
DE 6.50 usec
TE 298.0 K
D1 2.00000000 sec
D11 0.03000000 sec
TD0 1
======== CHANNEL f1 ========
NUC1 13C
P1 9.90 usec
PL1 -1.90 dB
PL1W 56.02249908 W
SFO1 100.6228298 MHz
======== CHANNEL f2 ========
CPDPRG2 waltz65
NUC2 1H
PCPD2 80.00 usec
PL2 0.30 dB
PL12 15.40 dB
PL13 18.40 dB
PL2W 11.25229836 W
PL12W 0.34772930 W
PL13W 0.17427748 W
SFO2 400.1316005 MHz
SI 32768
SF 100.6127690 MHz
WDW EM
SSB 0
LB 1.00 Hz
GB 0
PC 1.40
180 160 140 120 100 80 60 40 20 0 ppm

HRMS

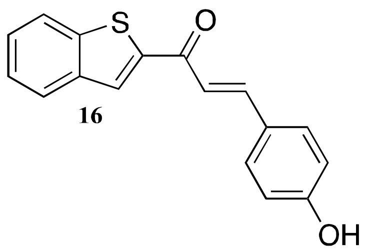

16

Compound Label	RT	Algorithm	Mass
Cpd 1: C17 H12 O2 S	0.13	Find By Formula	280.0555

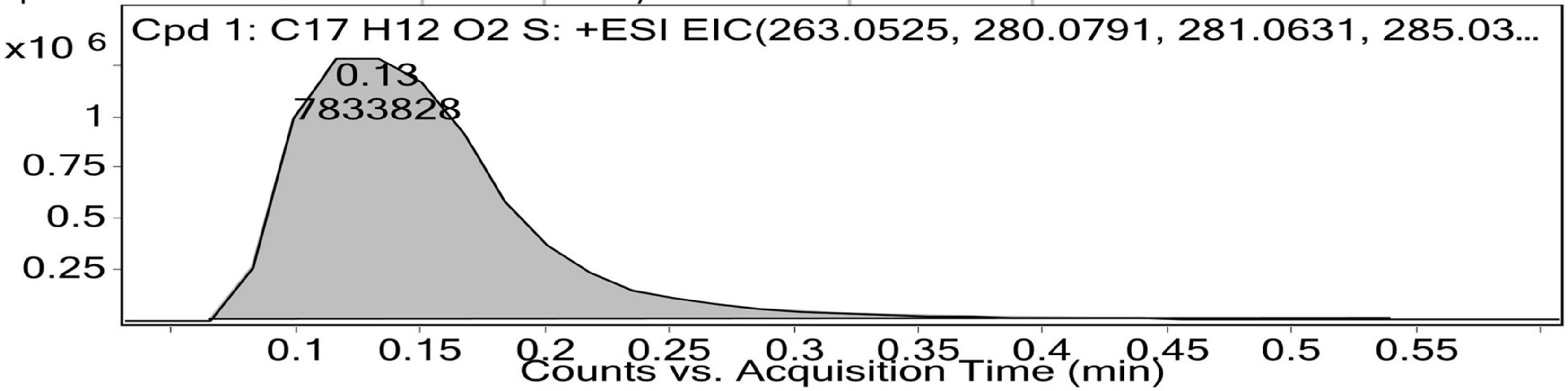

MS Spectrum

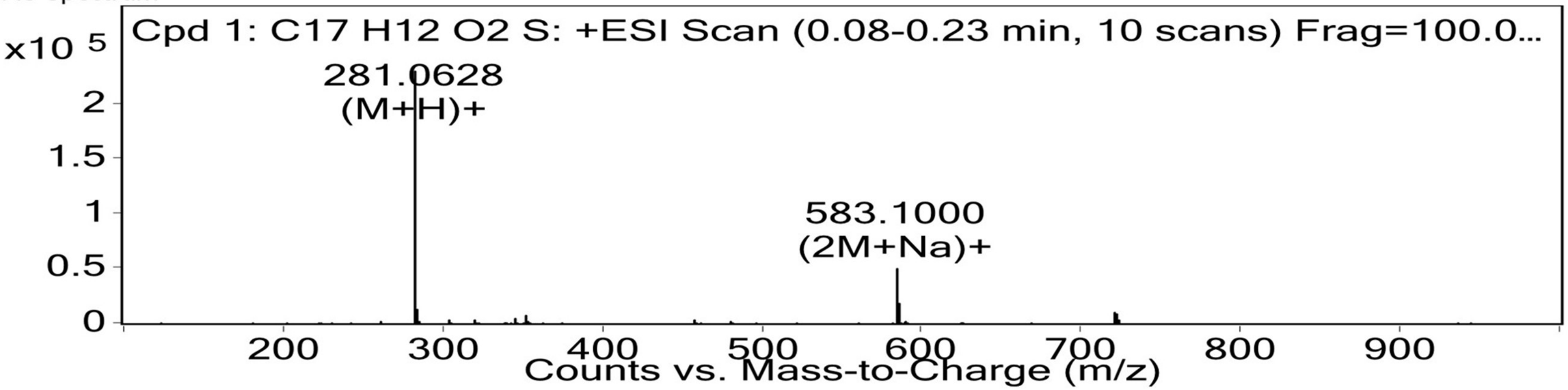

^{1}H NMR

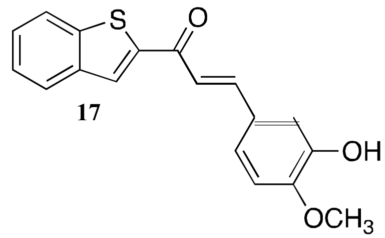

17

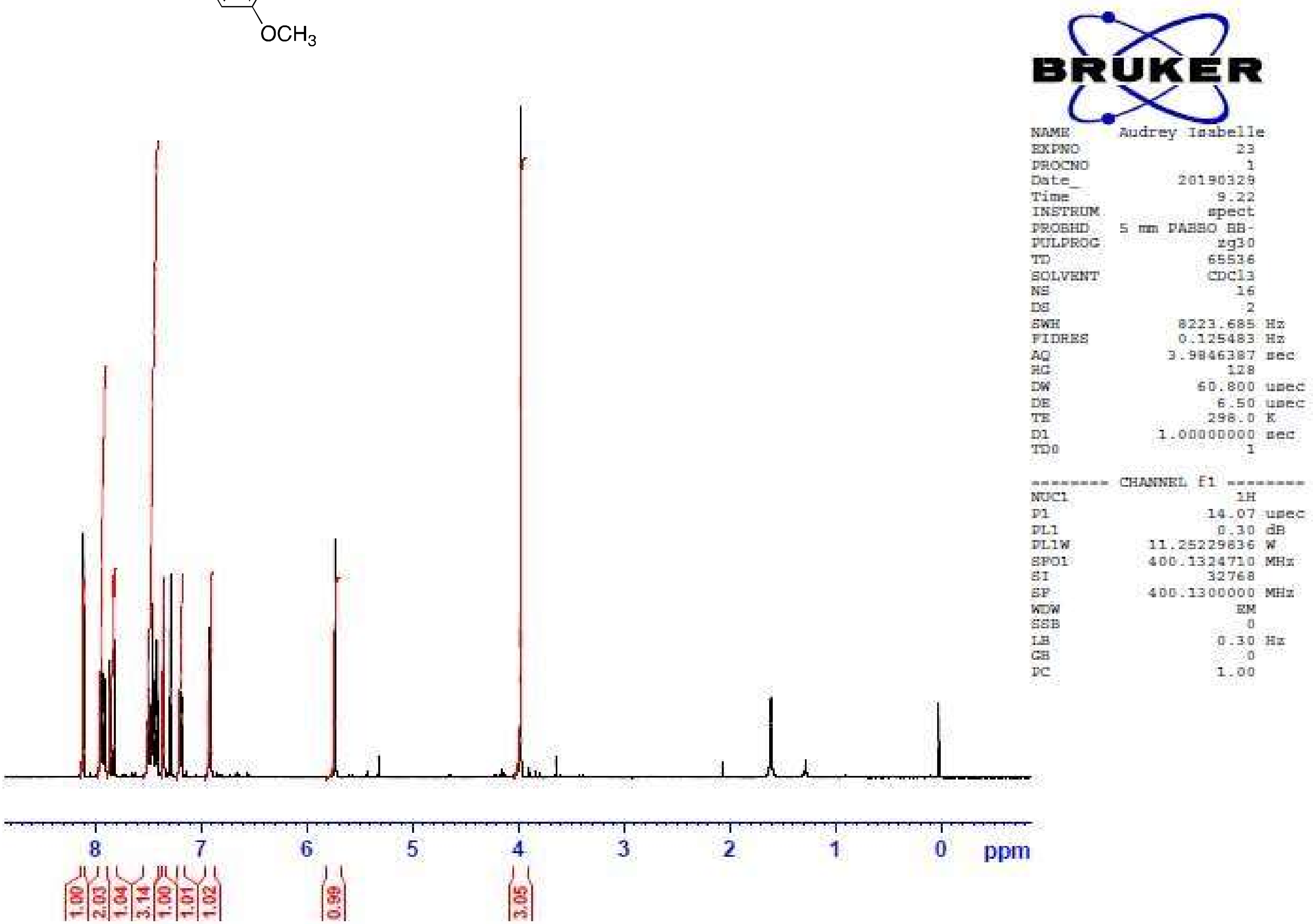
NAME Audrey Isabelle
EXPNO 23
PROCNO 1
Date_ 20190329
Time 9.22
INSTRUM spect
PROBHD 5 mm PABBO BB-
PULPROG zg30
TD 65536
SOLVENT CDCl3
NS 16
DS 2
SWH 8223.685 Hz
FIDRES 0.125483 Hz
AQ 3.9846387 sec
RG 128
DW 60.800 usec
DE 6.50 usec
TE 298.0 K
D1 1.00000000 sec
TD0 1
======== CHANNEL f1 ========
NUC1 1H
P1 14.07 usec
PL1 0.30 dB
PL1W 11.25229836 W
SFO1 400.1324710 MHz
SI 32768
SF 400.1300000 MHz
WDW EM
SSB 0
LB 0.30 Hz
GB 0
PC 1.00
8
7
6
5
4
3
2
1
0
ppm
1.00
2.03
1.04
3.14
1.00
1.01
1.02
0.99
3.05

^{13}C NMR

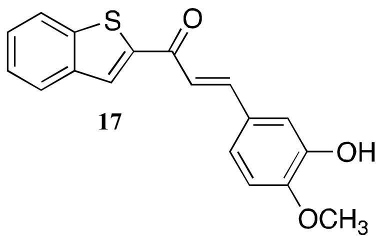

17

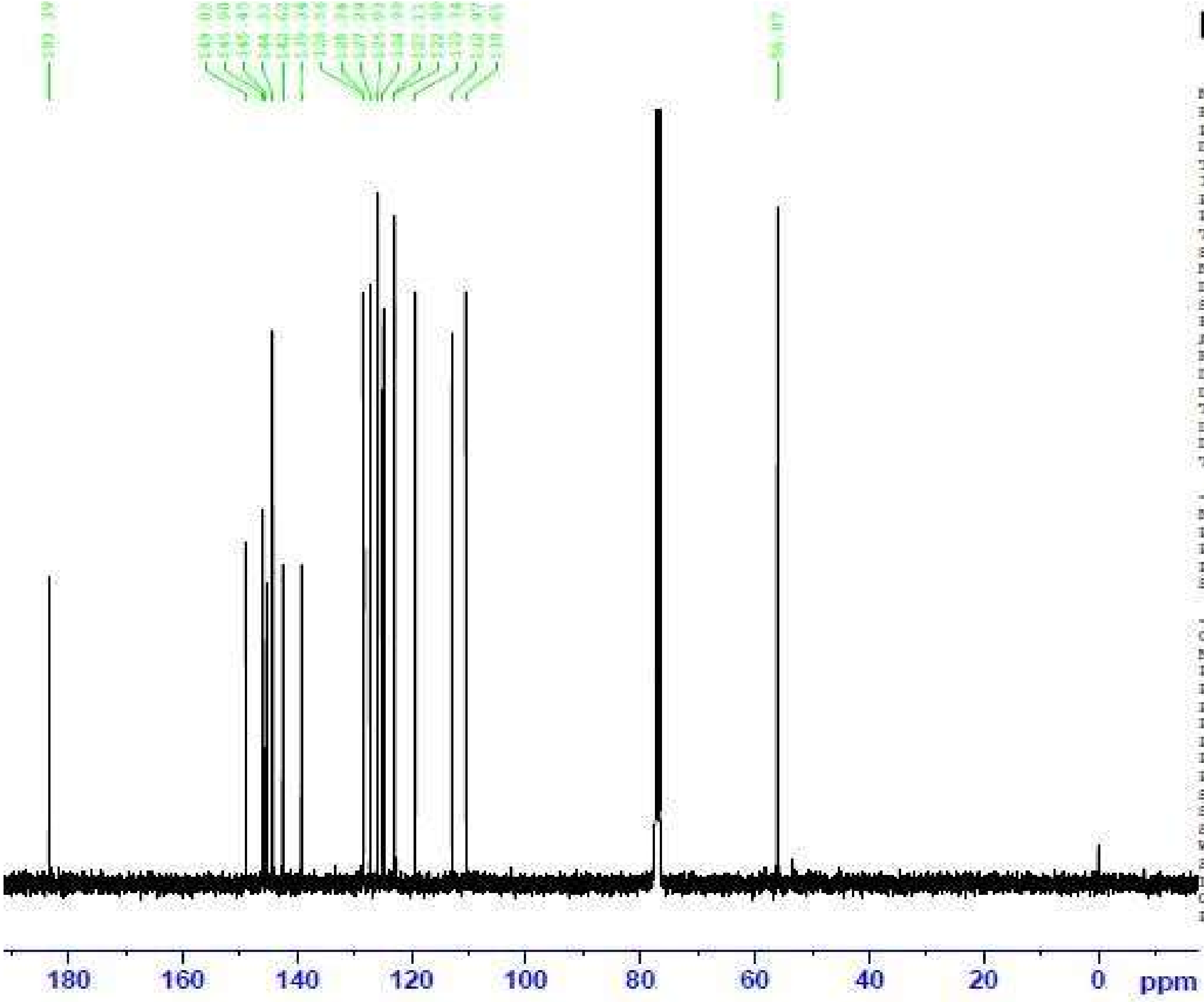


```
NAME       Audrey Isabelle
EXPNO                   24
PROCNO                   1
Date_             20190329
Time                 16.32
INSTRUM              spect
PROBHD   5 mm PABBO BB-
PULPROG            zgpg30
TD                   65536
SOLVENT              CDCl3
NS                    5629
DS                       4
SWH             24038.461 Hz
FIDRES           0.366798 Hz
AQ              1.3631988 sec
RG                     203
DW                  20.800 usec
DE                    6.50 usec
TE                   298.0 K
D1              2.00000000 sec
D11             0.03000000 sec
TD0                      1

======== CHANNEL f1 ========
NUC1                   13C
P1                    9.90 usec
PL1                  -1.90 dB
PL1W           56.02249908 W
SFO1           100.6228298 MHz

======== CHANNEL f2 ========
CPDPRG2            waltz65
NUC2                    1H
PCPD2                80.00 usec
PL2                   0.30 dB
PL12                 15.40 dB
PL13                 18.40 dB
PL2W           11.25229836 W
PL12W           0.34772930 W
PL13W           0.17427748 W
SFO2           400.1316005 MHz
SI                   32768
SF             100.6127690 MHz
WDW                     EM
SSB                      0
LB                    1.00 Hz
GB                       0
PC                    1.40
```

HRMS

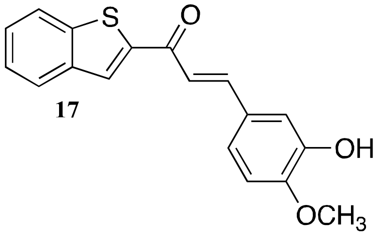

17

Compound Label	RT	Algorithm	Mass
Cpd 1: C18 H14 O3 S	0.12	Find By Formula	310.0657

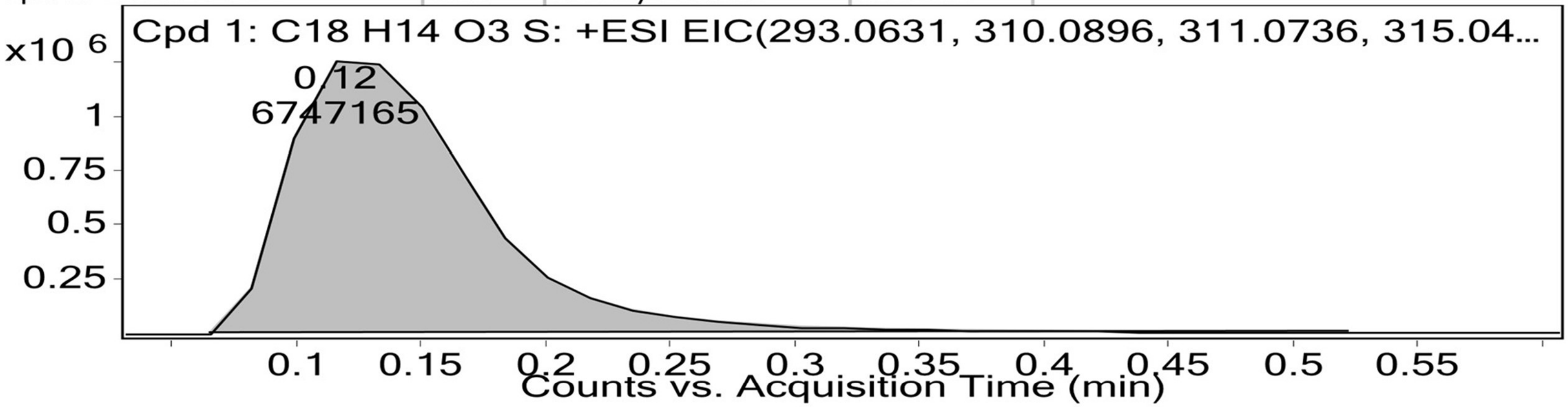

MS Spectrum

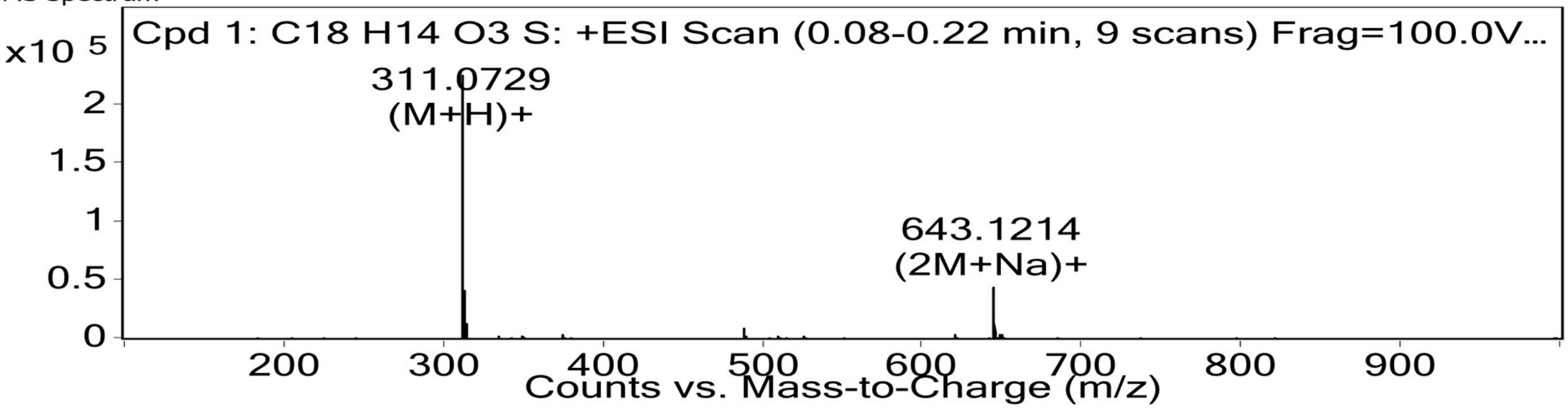

^{1}H NMR

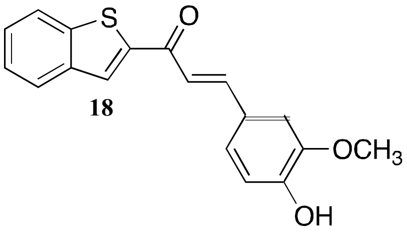

18

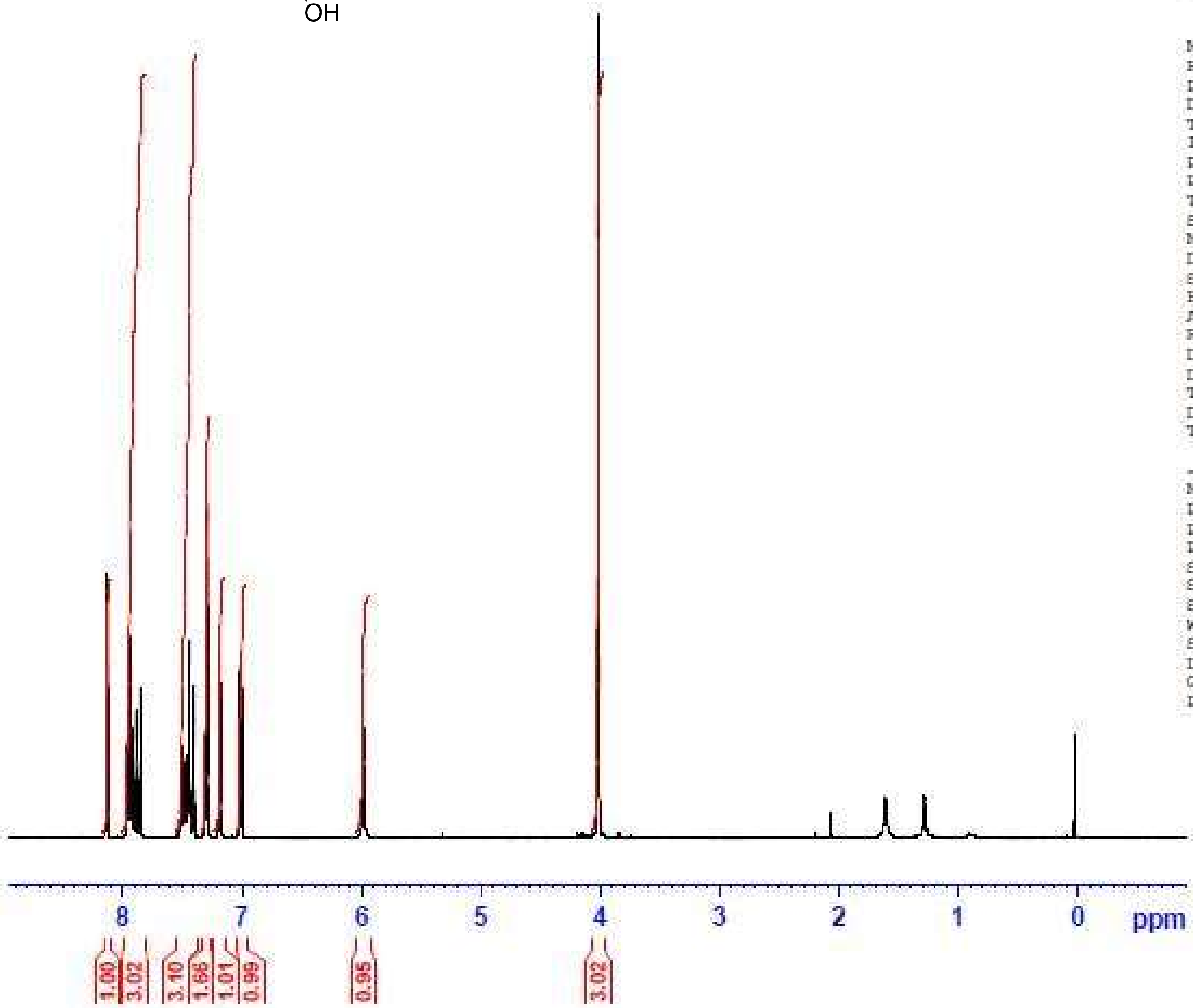

NAME	Audrey Isabelle
EXPNO	19
PROCNO	1
Date_	20190318
Time	9.03
INSTRUM	spect
PROBHD	5 mm PABBO BB-
PULPROG	zg30
TD	65536
SOLVENT	CDCl3
NS	16
DS	2
SWH	8223.685 Hz
FIDRES	0.125483 Hz
AQ	3.9846387 sec
RG	181
DW	60.800 usec
DE	6.50 usec
TE	298.0 K
D1	1.00000000 sec
TD0	1

======== CHANNEL f1 ========

NUC1	1H
P1	14.07 usec
PL1	0.30 dB
PL1W	11.25229836 W
SFO1	400.1324710 MHz
SI	32768
SF	400.1300000 MHz
WDW	EM
SSB	0
LB	0.30 Hz
GB	0
PC	1.00

^{13}C NMR

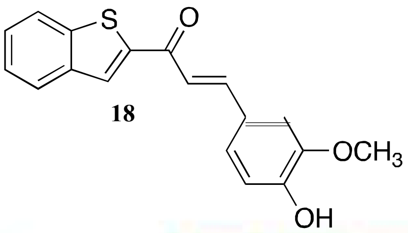
S
O
OCH3
OH

18

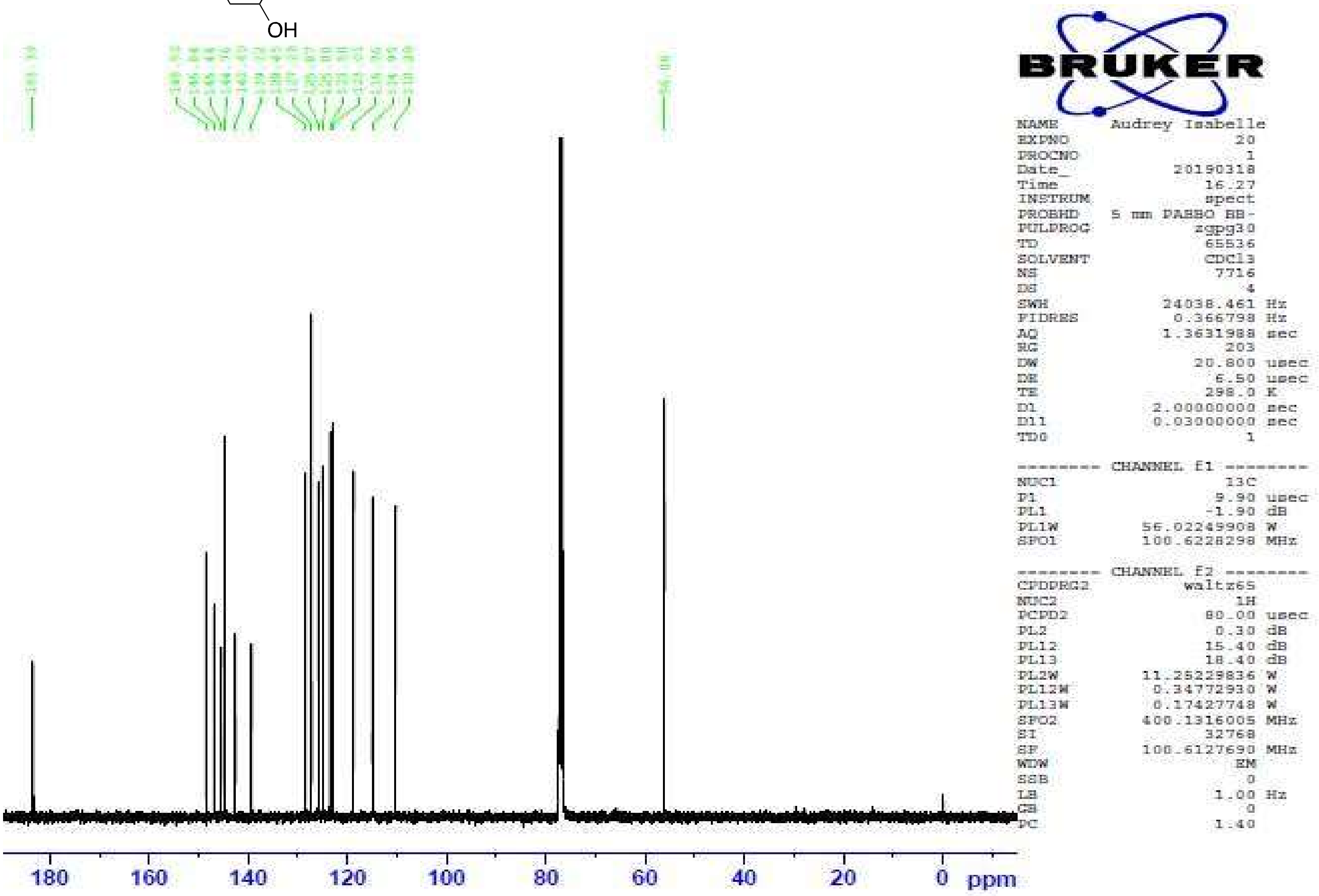
BRUKER
NAME Audrey Isabelle
EXPNO 20
PROCNO 1
Date_ 20190318
Time 16.27
INSTRUM spect
PROBHD 5 mm PABBO BB-
PULPROG zgpg30
TD 65536
SOLVENT CDCl3
NS 7716
DS 4
SWH 24038.461 Hz
FIDRES 0.366798 Hz
AQ 1.3631988 sec
RG 203
DW 20.800 usec
DE 6.50 usec
TE 298.0 K
D1 2.00000000 sec
D11 0.03000000 sec
TD0 1
======== CHANNEL f1 ========
NUC1 13C
P1 9.90 usec
PL1 -1.90 dB
PL1W 56.02249908 W
SFO1 100.6228298 MHz
======== CHANNEL f2 ========
CPDPRG2 waltz65
NUC2 1H
PCPD2 80.00 usec
PL2 0.30 dB
PL12 15.40 dB
PL13 18.40 dB
PL2W 11.25229836 W
PL12W 0.34772930 W
PL13W 0.17427748 W
SFO2 400.1316005 MHz
SI 32768
SF 100.6127690 MHz
WDW EM
SSB 0
LB 1.00 Hz
GB 0
PC 1.40
180 160 140 120 100 80 60 40 20 0 ppm

HRMS

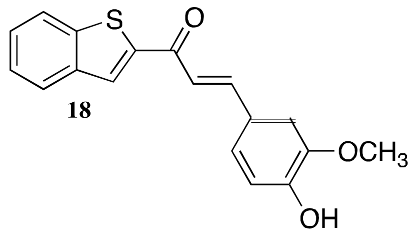

18

Compound Label	RT	Algorithm	Mass
Cpd 1: C18 H14 O3 S	0.13	Find By Formula	310.0663

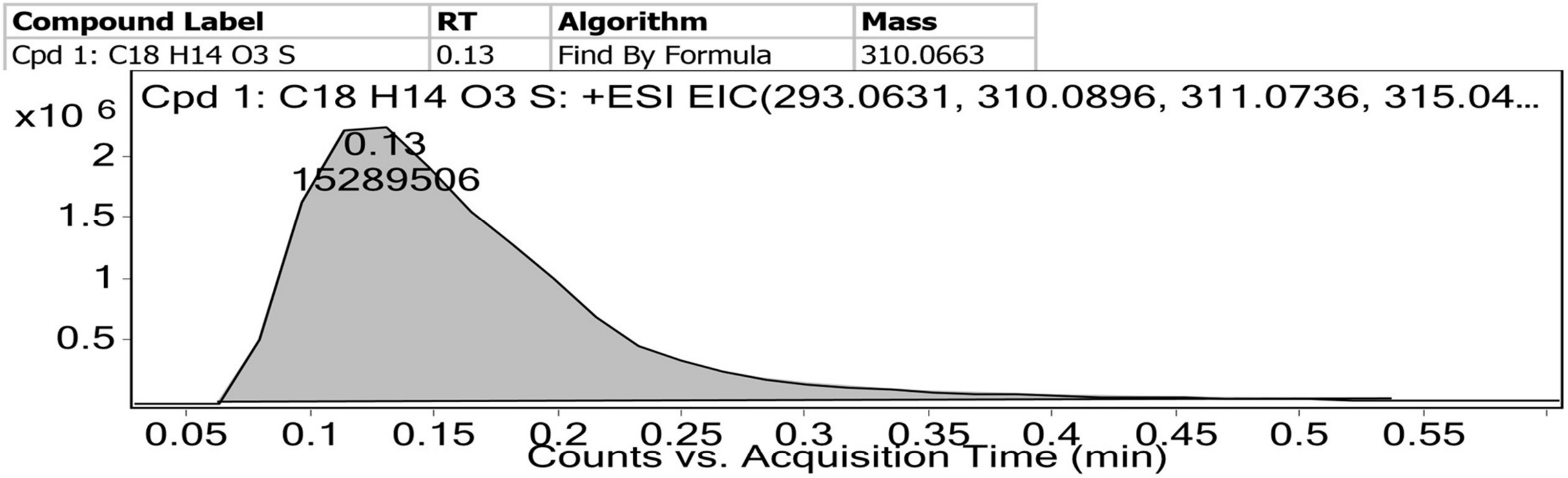

MS Spectrum

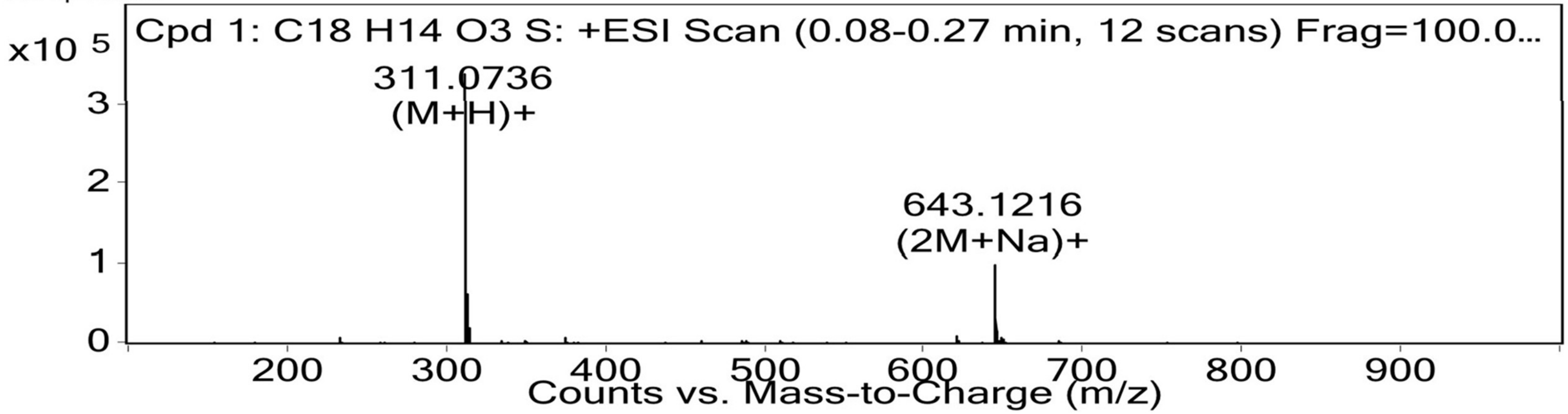

^{1}H NMR

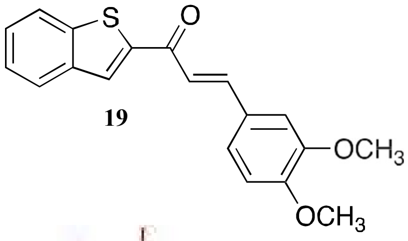
S
O
OCH3
OCH3

19

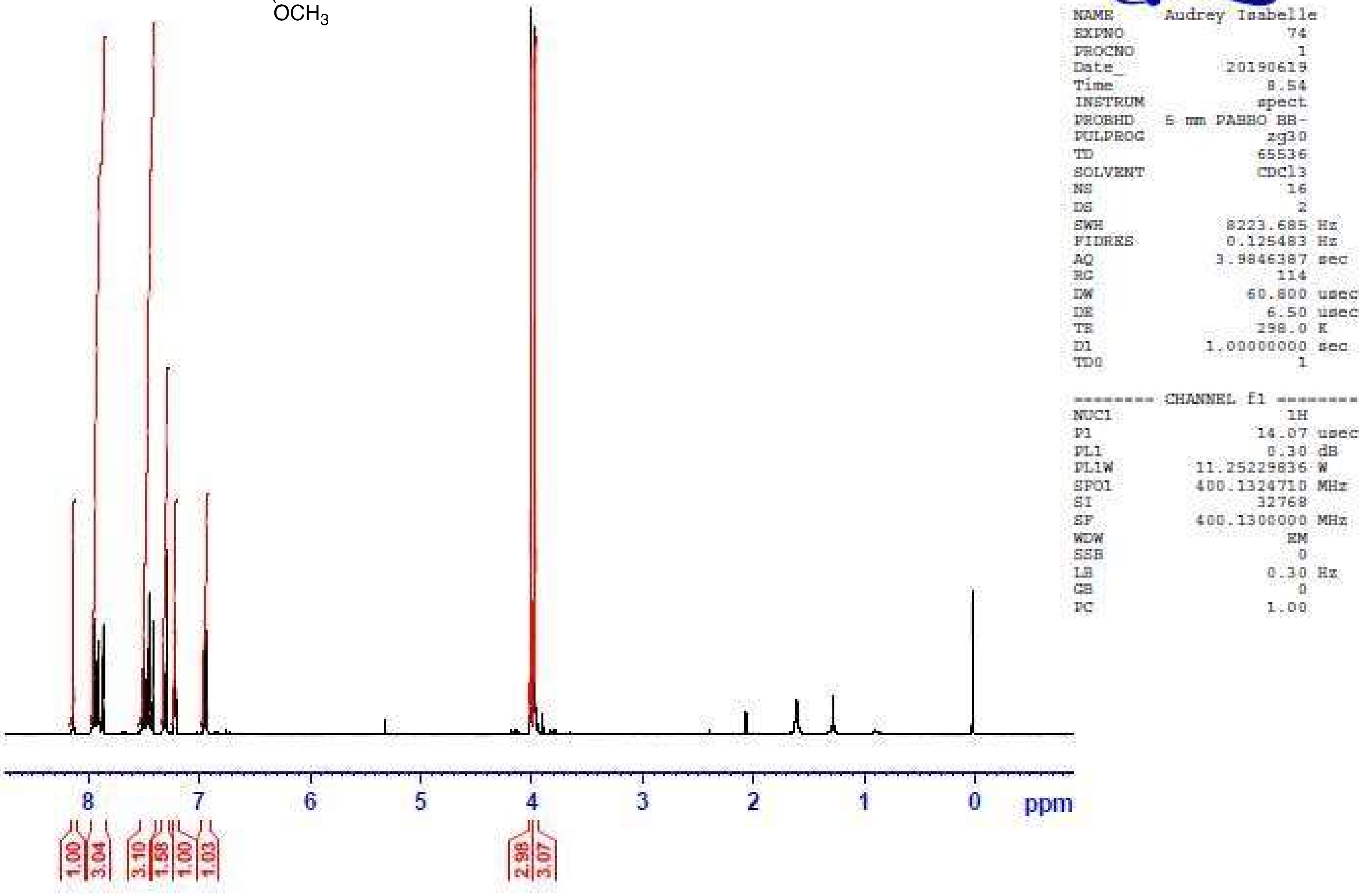
NAME Audrey Isabelle
EXPNO 74
PROCNO 1
Date_ 20190619
Time 8.54
INSTRUM spect
PROBHD 5 mm PABBO BB-
PULPROG zg30
TD 65536
SOLVENT CDCl3
NS 16
DS 2
SWH 8223.685 Hz
FIDRES 0.125483 Hz
AQ 3.9846387 sec
RG 114
DW 60.800 usec
DE 6.50 usec
TE 298.0 K
D1 1.00000000 sec
TD0 1
======== CHANNEL f1 ========
NUC1 1H
P1 14.07 usec
PL1 0.30 dB
PL1W 11.25229836 W
SFO1 400.1324710 MHz
SI 32768
SF 400.1300000 MHz
WDW EM
SSB 0
LB 0.30 Hz
GB 0
PC 1.00
8
7
6
5
4
3
2
1
0
ppm
1.00
3.04
3.10
1.58
1.00
1.03
2.98
3.07

^{13}C NMR

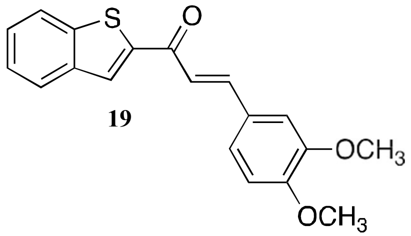

19

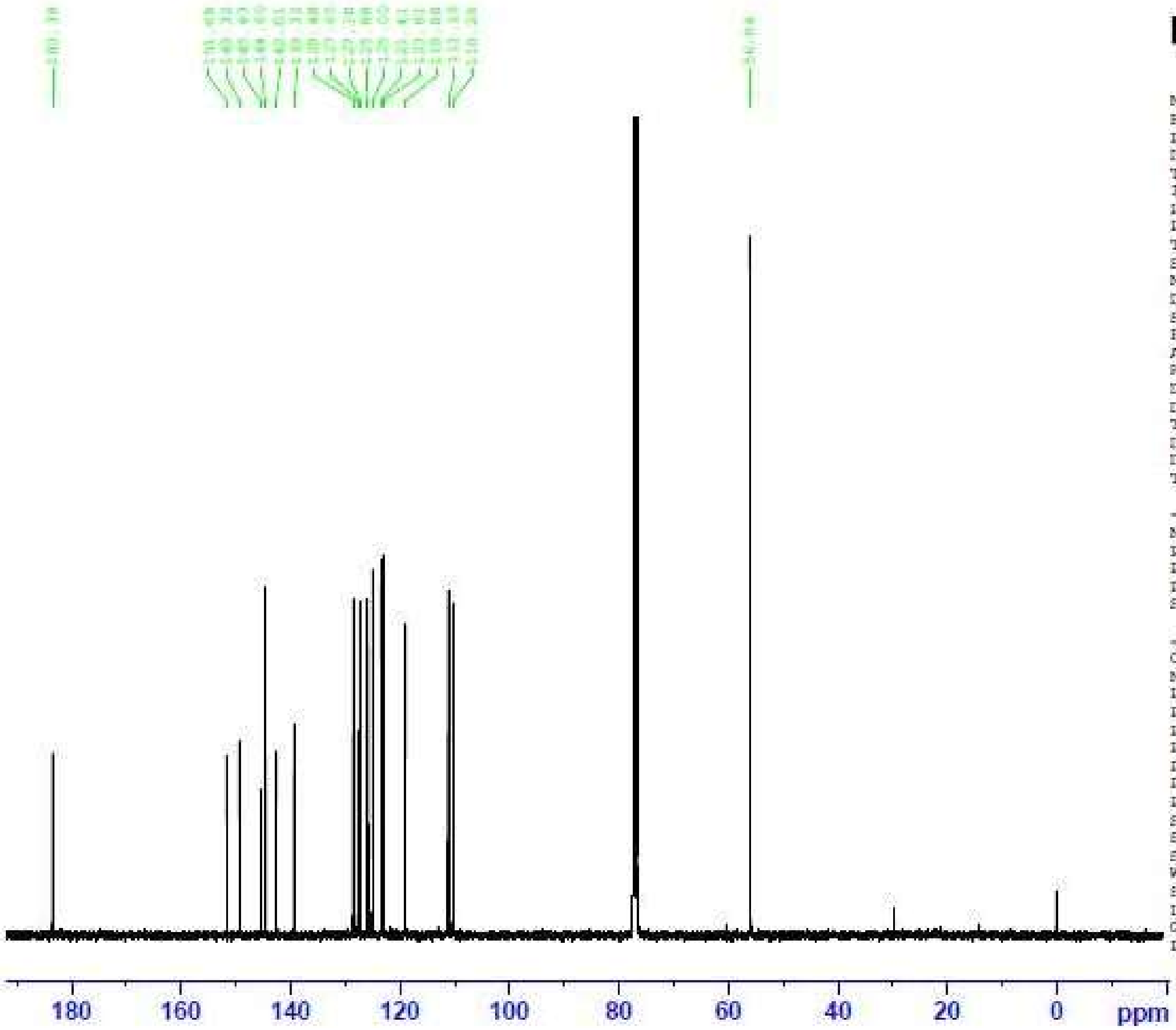

NAME	Audrey Isabelle
EXPNO	82
PROCNO	1
Date_	20190621
Time	2.09
INSTRUM	spect
PROBHD	5 mm PABBO BB-
PULPROG	zgpg30
TD	65536
SOLVENT	CDCl3
NS	10000
DS	4
SWH	24038.461 Hz
FIDRES	0.366798 Hz
AQ	1.3631988 sec
RG	203
DW	20.800 usec
DE	6.50 usec
TE	298.0 K
D1	2.00000000 sec
D11	0.03000000 sec
TD0	1
======== CHANNEL f1 ========	
NUC1	13C
P1	9.90 usec
PL1	-1.90 dB
PL1W	56.02249908 W
SFO1	100.6228298 MHz
======== CHANNEL f2 ========	
CPDPRG2	waltz65
NUC2	1H
PCPD2	80.00 usec
PL2	0.30 dB
PL12	15.40 dB
PL13	18.40 dB
PL2W	11.25229836 W
PL12W	0.34772930 W
PL13W	0.17427748 W
SFO2	400.1316005 MHz
SI	32768
SF	100.6127690 MHz
WDW	EM
SSB	0
LB	1.00 Hz
GB	0
PC	1.40

HRMS

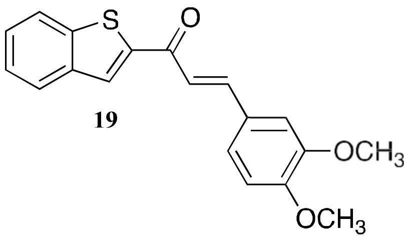
S
O
OCH3
OCH3

19

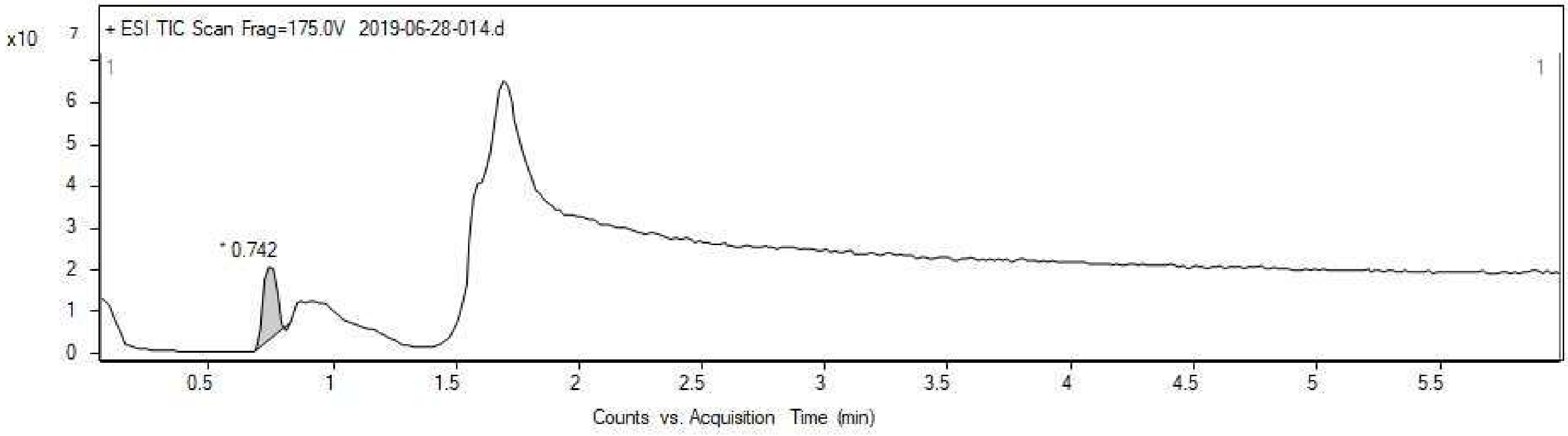
x10 7
+ ESI TIC Scan Frag=175.0V 2019-06-28-014.d
* 0.742
0.5
1
1.5
2
2.5
3
3.5
4
4.5
5
5.5
Counts vs. Acquisition Time (min)

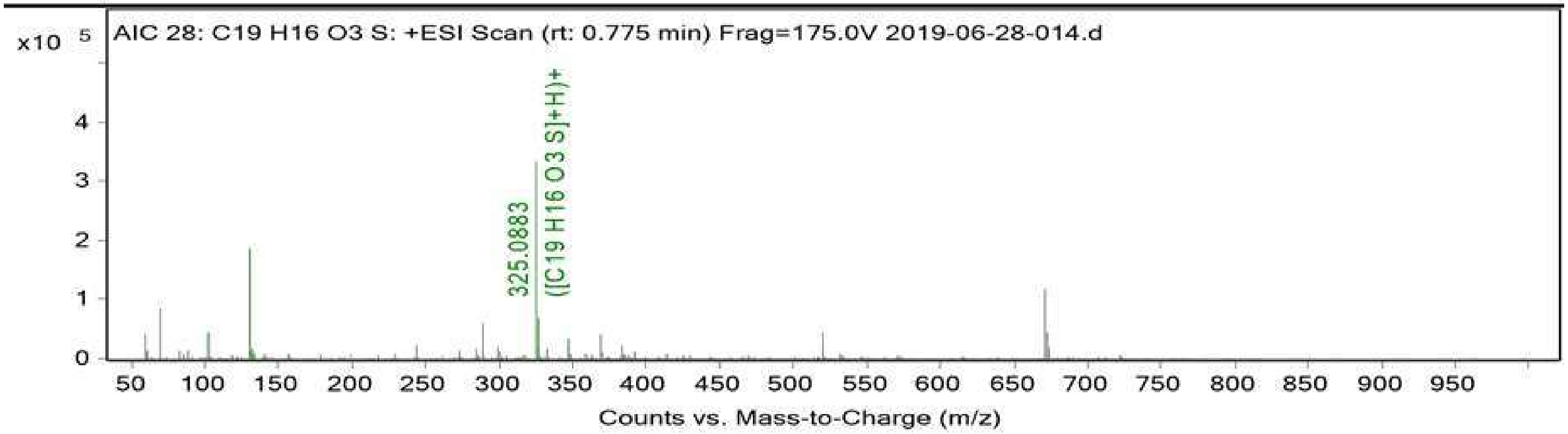
x10 5
AIC 28: C19 H16 O3 S: +ESI Scan (rt: 0.775 min) Frag=175.0V 2019-06-28-014.d
325.0883
([C19 H16 O3 S]+H)+
Counts vs. Mass-to-Charge (m/z)

^{1}H NMR

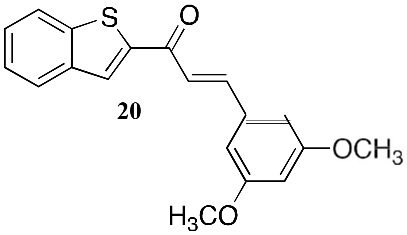

20

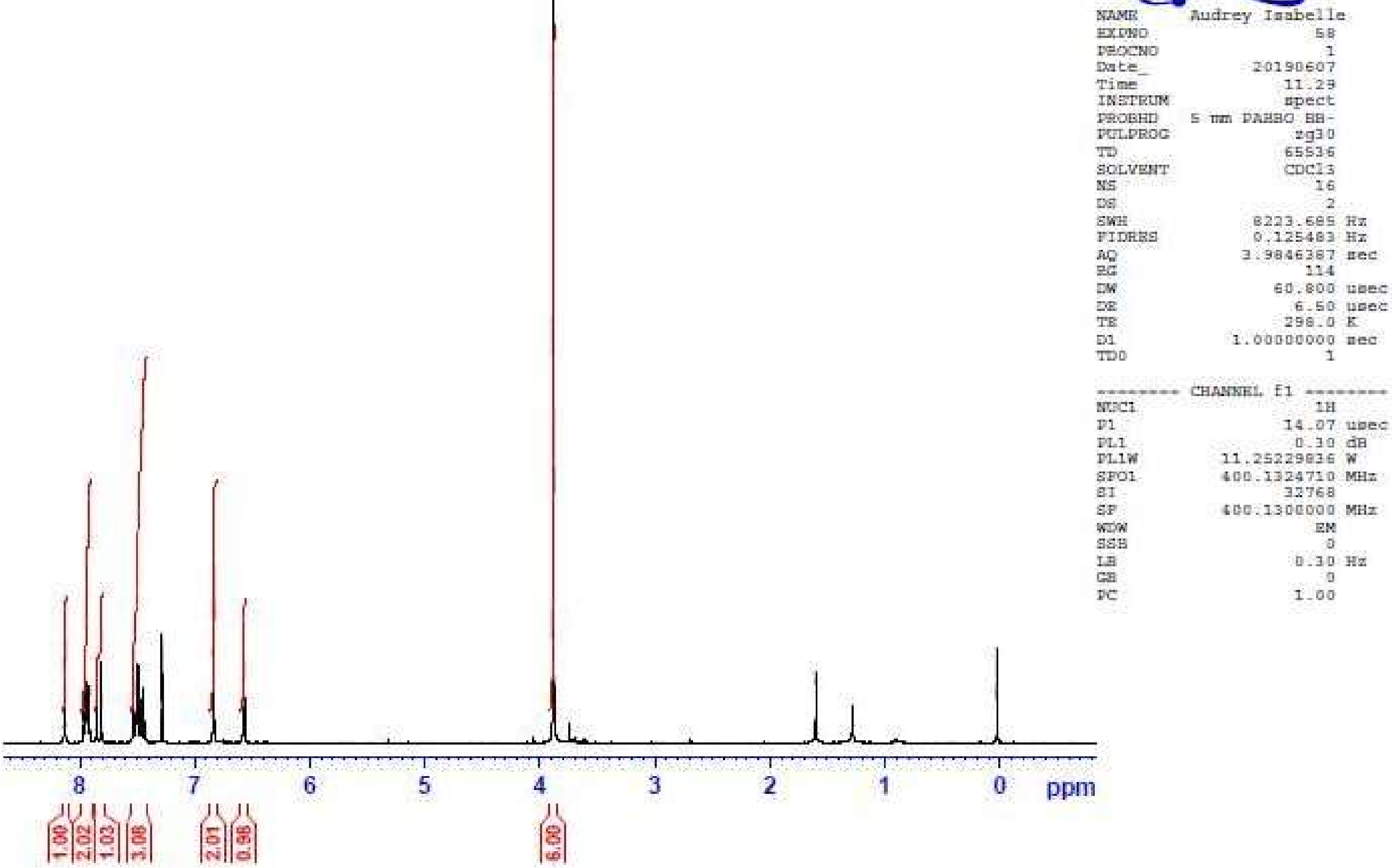

BRUKER
NAME Audrey Isabelle
EXPNO 58
PROCNO 1
Date_ 20190607
Time 11.29
INSTRUM spect
PROBHD 5 mm PABBO BB-
PULPROG zg30
TD 65536
SOLVENT CDCl3
NS 16
DS 2
SWH 8223.685 Hz
FIDRES 0.125483 Hz
AQ 3.9846387 sec
RG 114
DW 60.800 usec
DE 6.50 usec
TE 298.0 K
D1 1.00000000 sec
TD0 1
======== CHANNEL f1 ========
NUC1 1H
P1 14.07 usec
PL1 0.30 dB
PL1W 11.25229836 W
SFO1 400.1324710 MHz
SI 32768
SF 400.1300000 MHz
WDW EM
SSB 0
LB 0.30 Hz
GB 0
PC 1.00
8
7
6
5
4
3
2
1
0
ppm
1.00
2.02
1.03
3.08
2.01
0.98
6.00

^{13}C NMR

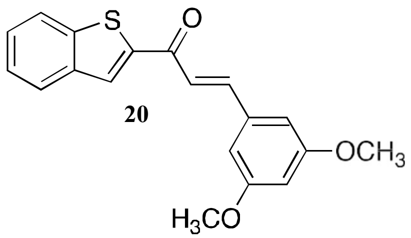

20

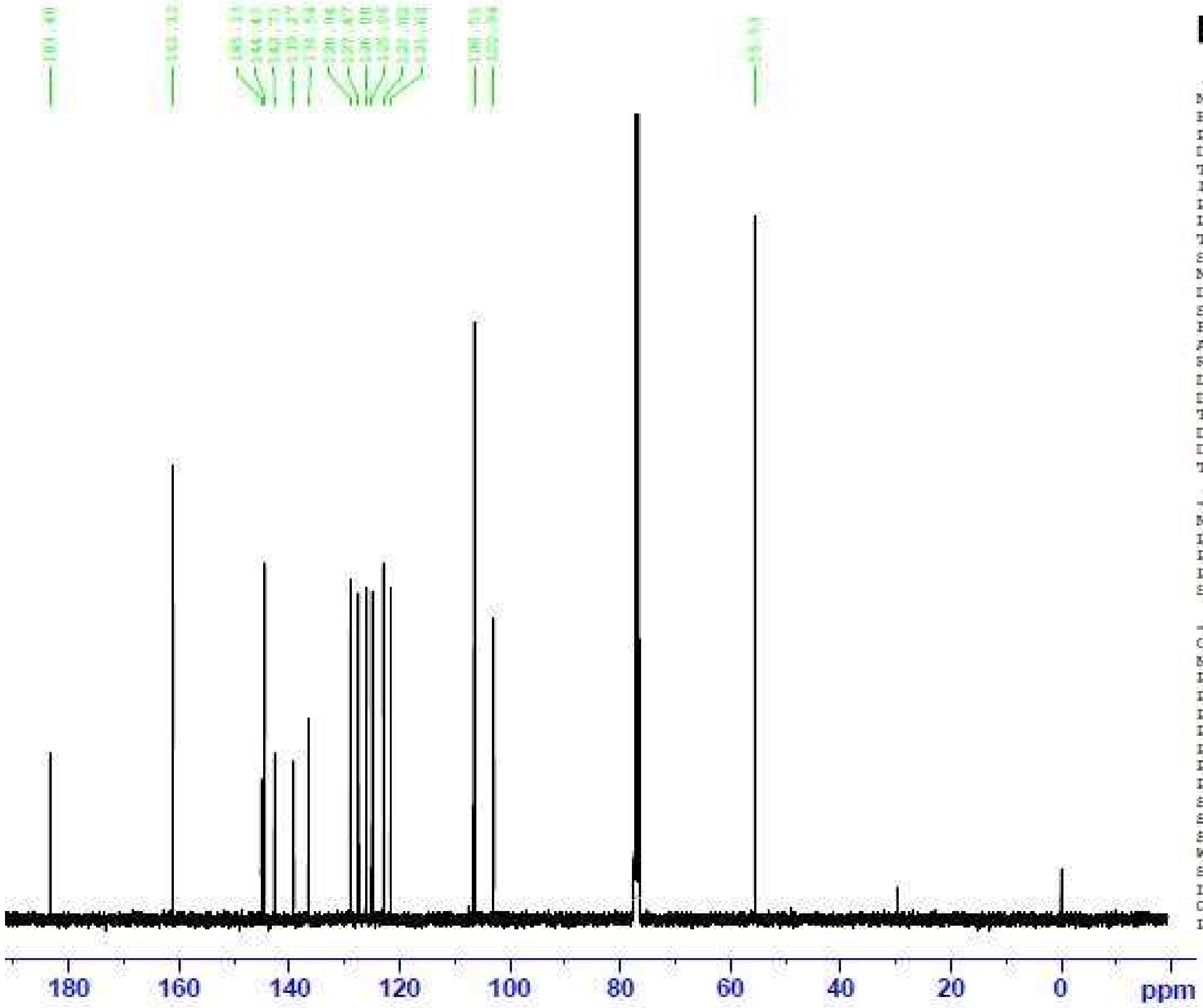

BRUKER

```
NAME        Audrey Isabelle
EXPNO                    59
PROCNO                    1
Date_              20190607
Time                  14.39
INSTRUM               spect
PROBHD     5 mm PABBO BB-
PULPROG             zgpg30
TD                   65536
SOLVENT              CDCl3
NS                    3292
DS                       4
SWH              24038.461 Hz
FIDRES            0.366798 Hz
AQ               1.3631988 sec
RG                     203
DW                  20.800 usec
DE                    6.50 usec
TE                   298.0 K
D1              2.00000000 sec
D11             0.03000000 sec
TD0                      1

======== CHANNEL f1 ========
NUC1                   13C
P1                    9.90 usec
PL1                  -1.90 dB
PL1W           56.02249908 W
SFO1           100.6228298 MHz

======== CHANNEL f2 ========
CPDPRG2            waltz65
NUC2                    1H
PCPD2                80.00 usec
PL2                   0.30 dB
PL12                 15.40 dB
PL13                 18.40 dB
PL2W           11.25229836 W
PL12W           0.34772930 W
PL13W           0.17427748 W
SFO2           400.1316005 MHz
SI                   32768
SF             100.6127690 MHz
WDW                     EM
SSB                      0
LB                    1.00 Hz
GB                       0
PC                    1.40
```

HRMS

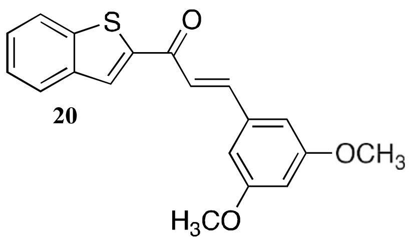
S
O
OCH3
H3CO

20

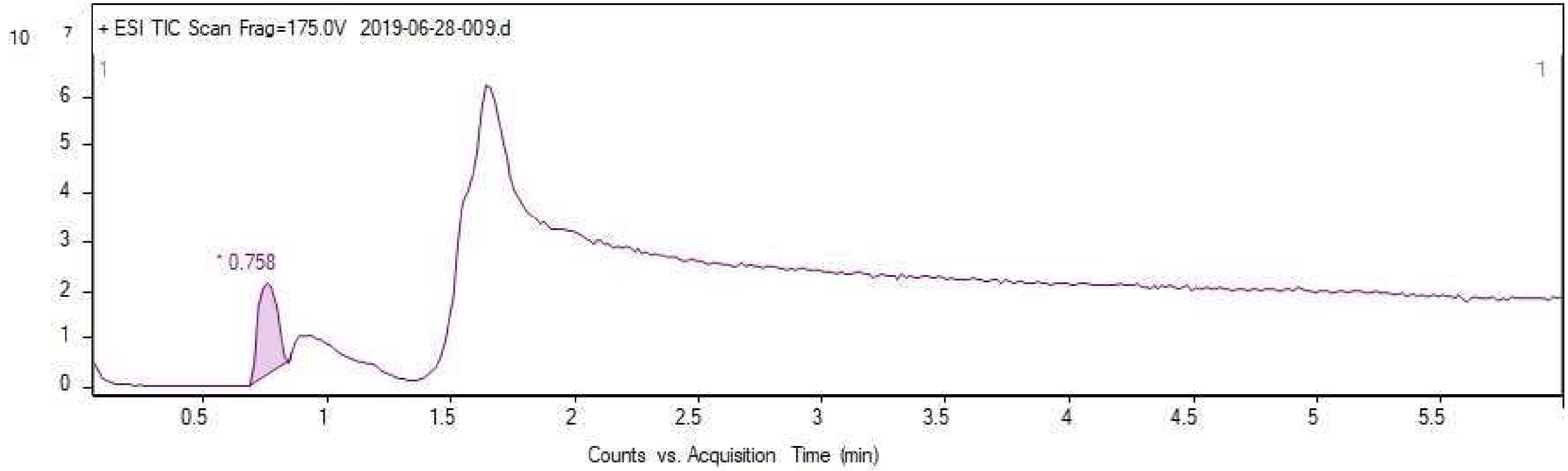
10 7
+ ESI TIC Scan Frag=175.0V 2019-06-28-009.d
* 0.758
Counts vs. Acquisition Time (min)

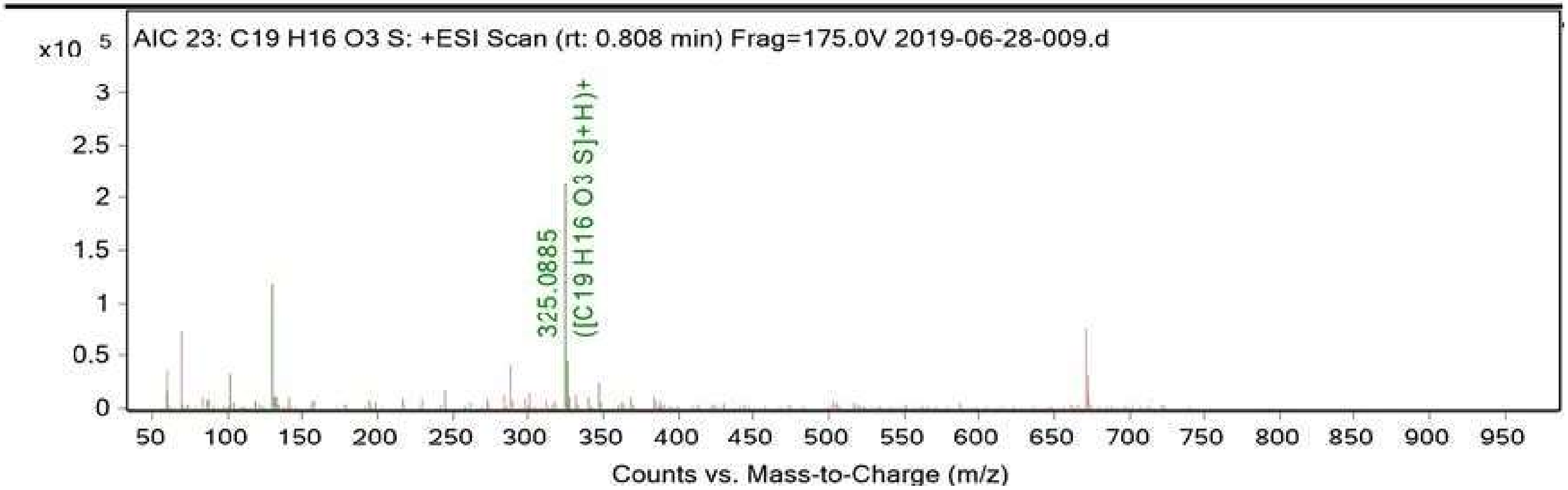
x10 5
AIC 23: C19 H16 O3 S: +ESI Scan (rt: 0.808 min) Frag=175.0V 2019-06-28-009.d
325.0885
([C19 H16 O3 S]+H)+
Counts vs. Mass-to-Charge (m/z)

^{1}H NMR

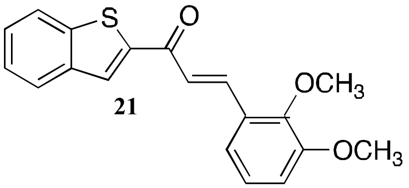

21

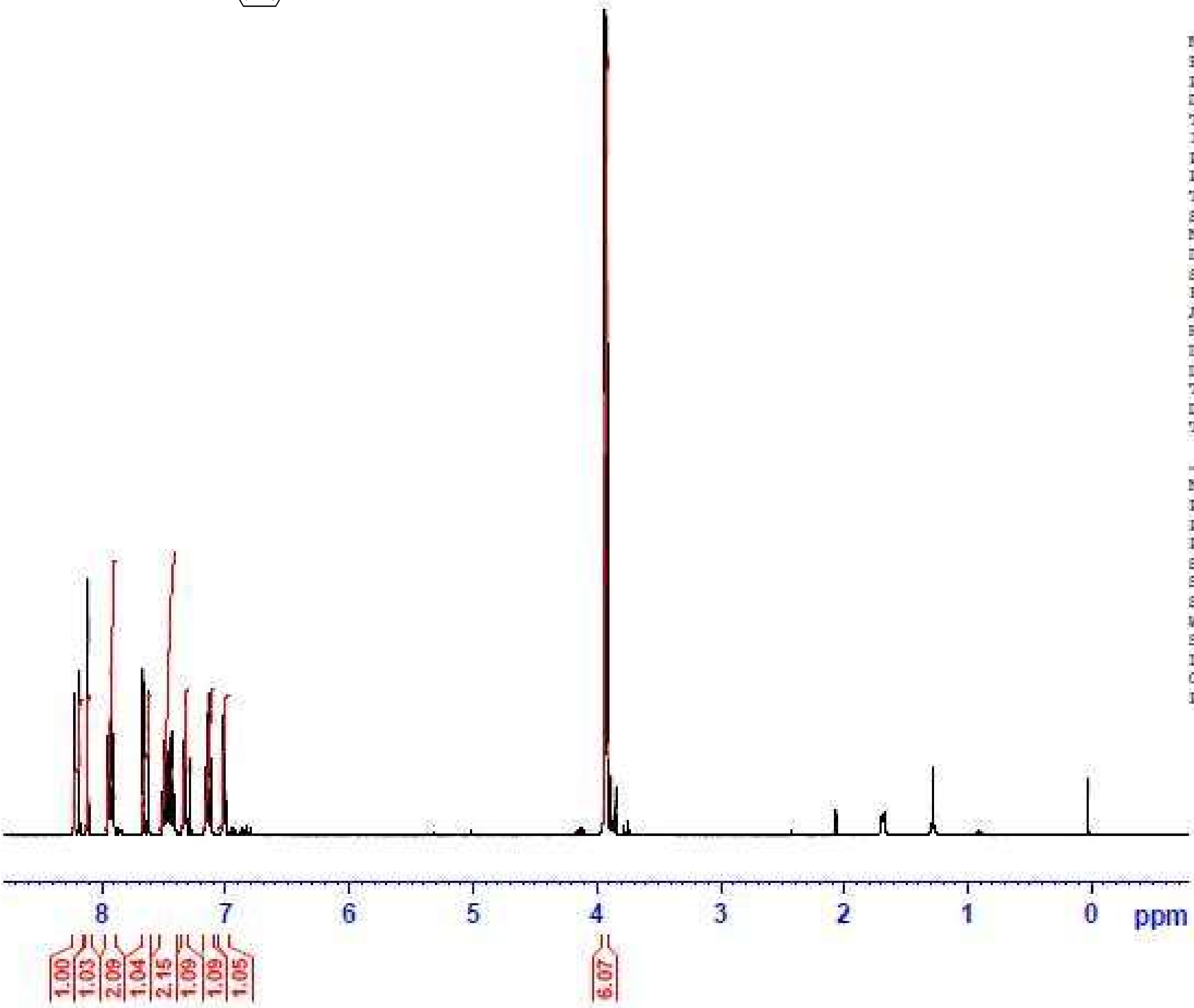

NAME	Audrey Isabelle
EXPNO	83
PROCNO	1
Date_	20190621
Time	8.27
INSTRUM	spect
PROBHD	5 mm PABBO BB-
PULPROG	zg30
TD	65536
SOLVENT	CDCl3
NS	16
DS	2
SWH	8223.685 Hz
FIDRES	0.125483 Hz
AQ	3.9846387 sec
RG	64
DW	60.800 usec
DE	6.50 usec
TE	298.0 K
D1	1.00000000 sec
TD0	1
======== CHANNEL f1 ========	
NUC1	1H
P1	14.07 usec
PL1	0.30 dB
PL1W	11.25229836 W
SFO1	400.1324710 MHz
SI	32768
SF	400.1300000 MHz
WDW	EM
SSB	0
LB	0.30 Hz
GB	0
PC	1.00

^{13}C NMR

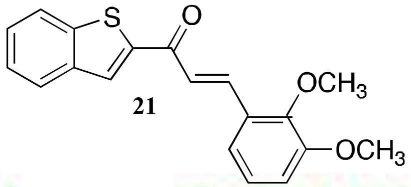

21

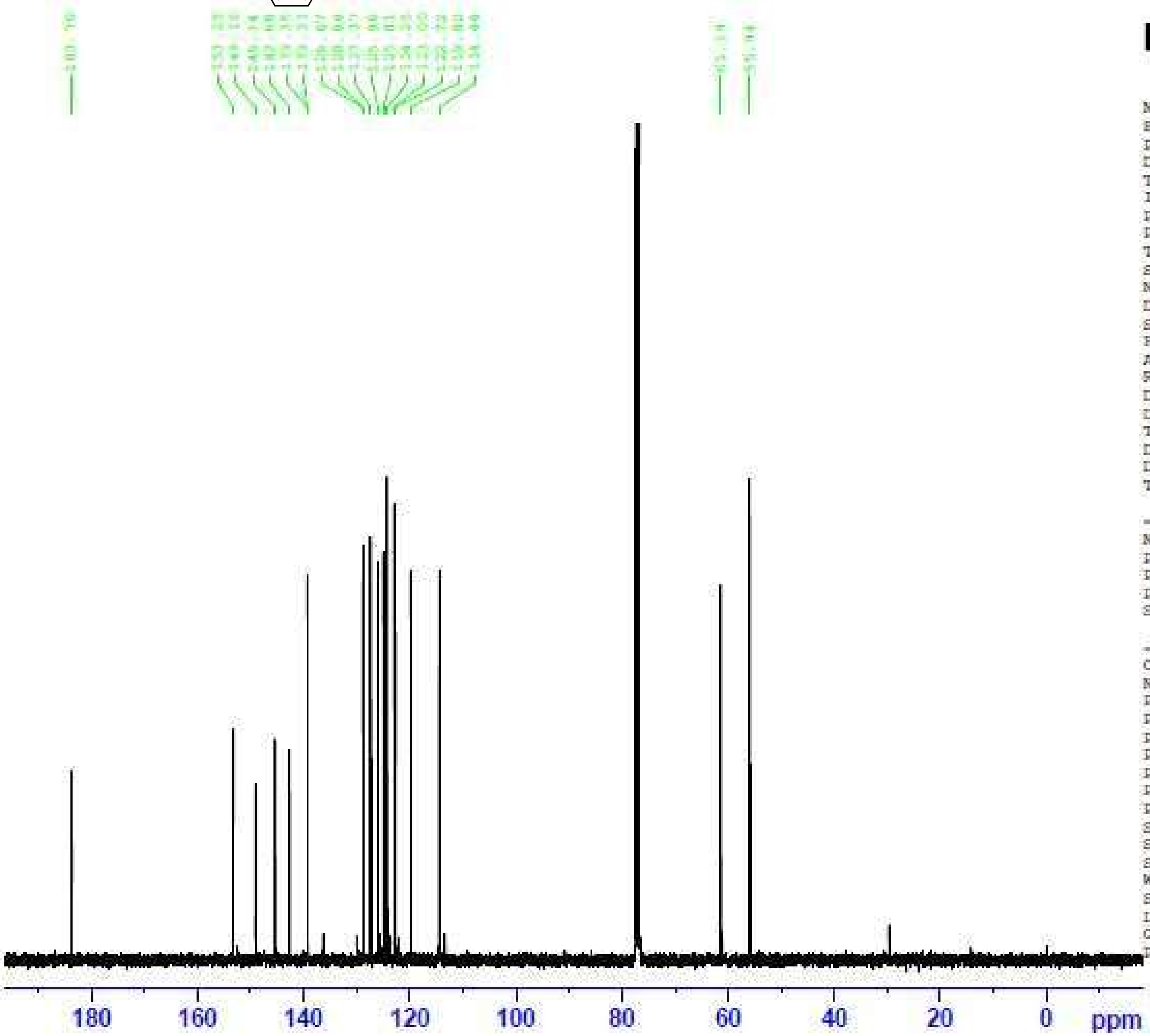

NAME	Audrey Isabelle	
EXPNO	85	
PROCNO	1	
Date_	20190621	
Time	9.49	
INSTRUM	spect	
PROBHD	5 mm PABBO BB-	
PULPROG	zgpg30	
TD	65536	
SOLVENT	CDCl3	
NS	637	
DS	4	
SWH	24038.461	Hz
FIDRES	0.366798	Hz
AQ	1.3631988	sec
RG	203	
DW	20.800	usec
DE	6.50	usec
TE	297.9	K
D1	2.00000000	sec
D11	0.03000000	sec
TD0	1	
======== CHANNEL f1 ========		
NUC1	13C	
P1	9.90	usec
PL1	-1.90	dB
PL1W	56.02249908	W
SFO1	100.6228298	MHz
======== CHANNEL f2 ========		
CPDPRG2	waltz65	
NUC2	1H	
PCPD2	80.00	usec
PL2	0.30	dB
PL12	15.40	dB
PL13	18.40	dB
PL2W	11.25229836	W
PL12W	0.34772930	W
PL13W	0.17427748	W
SFO2	400.1316005	MHz
SI	32768	
SF	100.6127690	MHz
WDW	EM	
SSB	0	
LB	1.00	Hz
GB	0	
PC	1.40	

HRMS

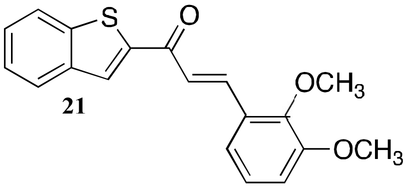
S
O
OCH3
OCH3

21

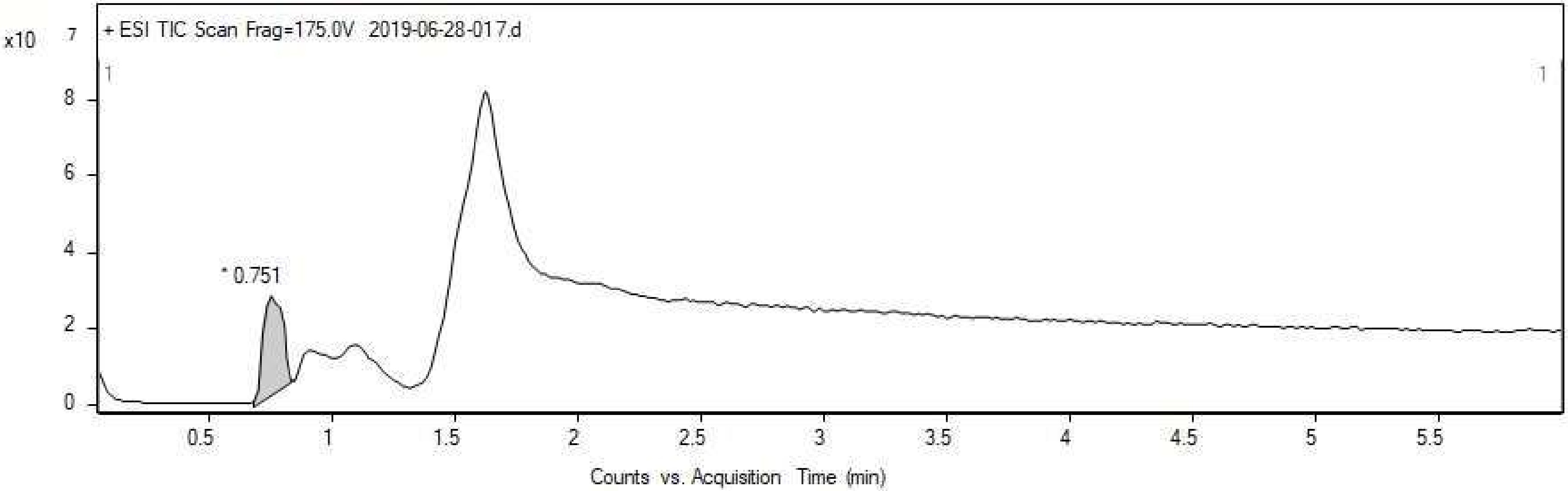
x10 7
+ ESI TIC Scan Frag=175.0V 2019-06-28-017.d
* 0.751
Counts vs. Acquisition Time (min)

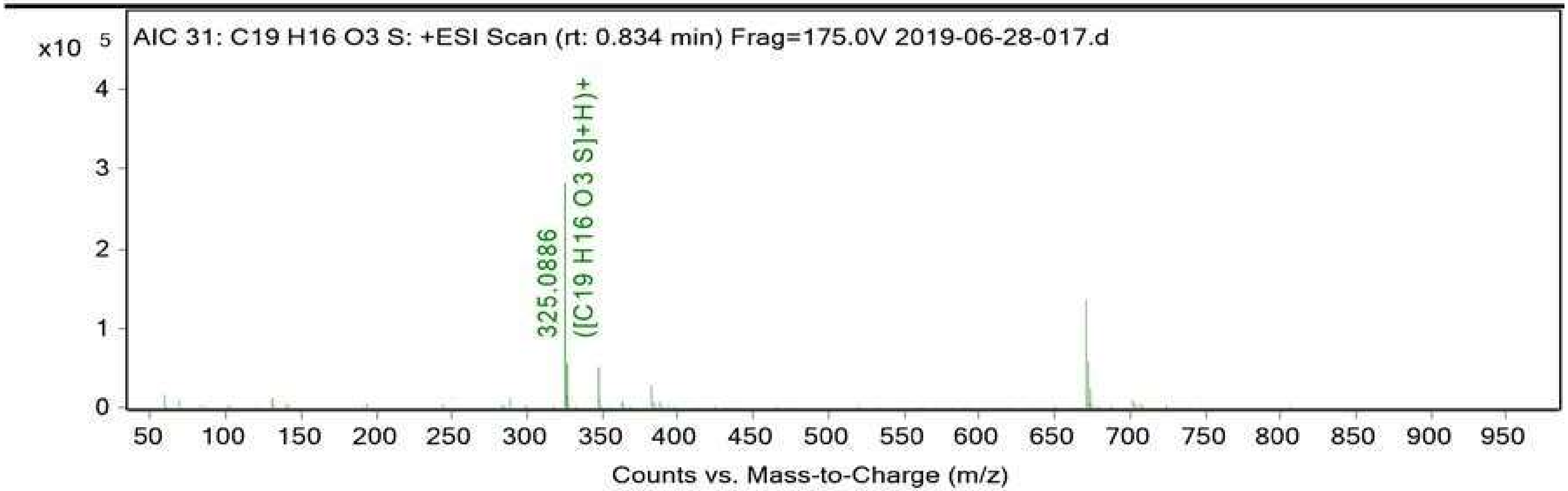
x10 5
AIC 31: C19 H16 O3 S: +ESI Scan (rt: 0.834 min) Frag=175.0V 2019-06-28-017.d
325.0886
([C19 H16 O3 S]+H)+
Counts vs. Mass-to-Charge (m/z)

^{1}H NMR

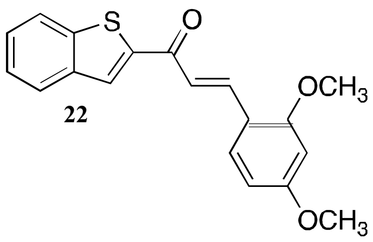

22

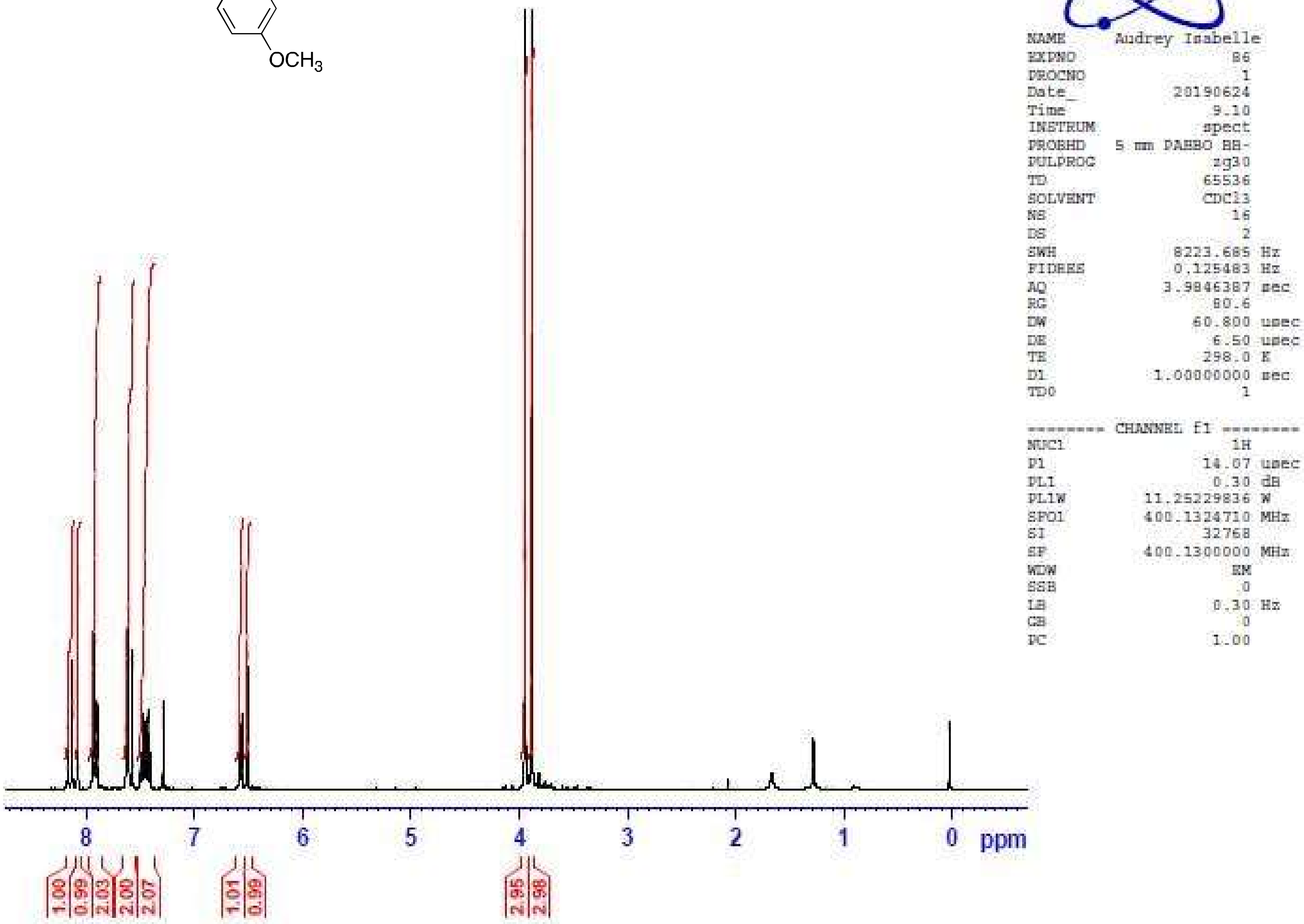
BRUKER
NAME Audrey Isabelle
EXPNO 86
PROCNO 1
Date_ 20190624
Time 9.10
INSTRUM spect
PROBHD 5 mm PABBO BB-
PULPROG zg30
TD 65536
SOLVENT CDCl3
NS 16
DS 2
SWH 8223.685 Hz
FIDRES 0.125483 Hz
AQ 3.9846387 sec
RG 80.6
DW 60.800 usec
DE 6.50 usec
TE 298.0 K
D1 1.00000000 sec
TD0 1
======== CHANNEL f1 ========
NUC1 1H
P1 14.07 usec
PL1 0.30 dB
PL1W 11.25229836 W
SFO1 400.1324710 MHz
SI 32768
SF 400.1300000 MHz
WDW EM
SSB 0
LB 0.30 Hz
GB 0
PC 1.00
8 7 6 5 4 3 2 1 0 ppm
1.00 0.99 2.03 2.00 2.07 1.01 0.99 2.95 2.98

^{13}C NMR

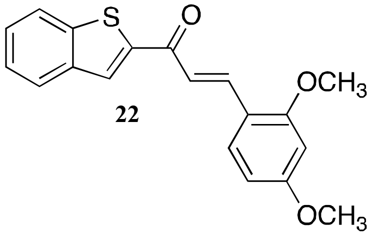

22

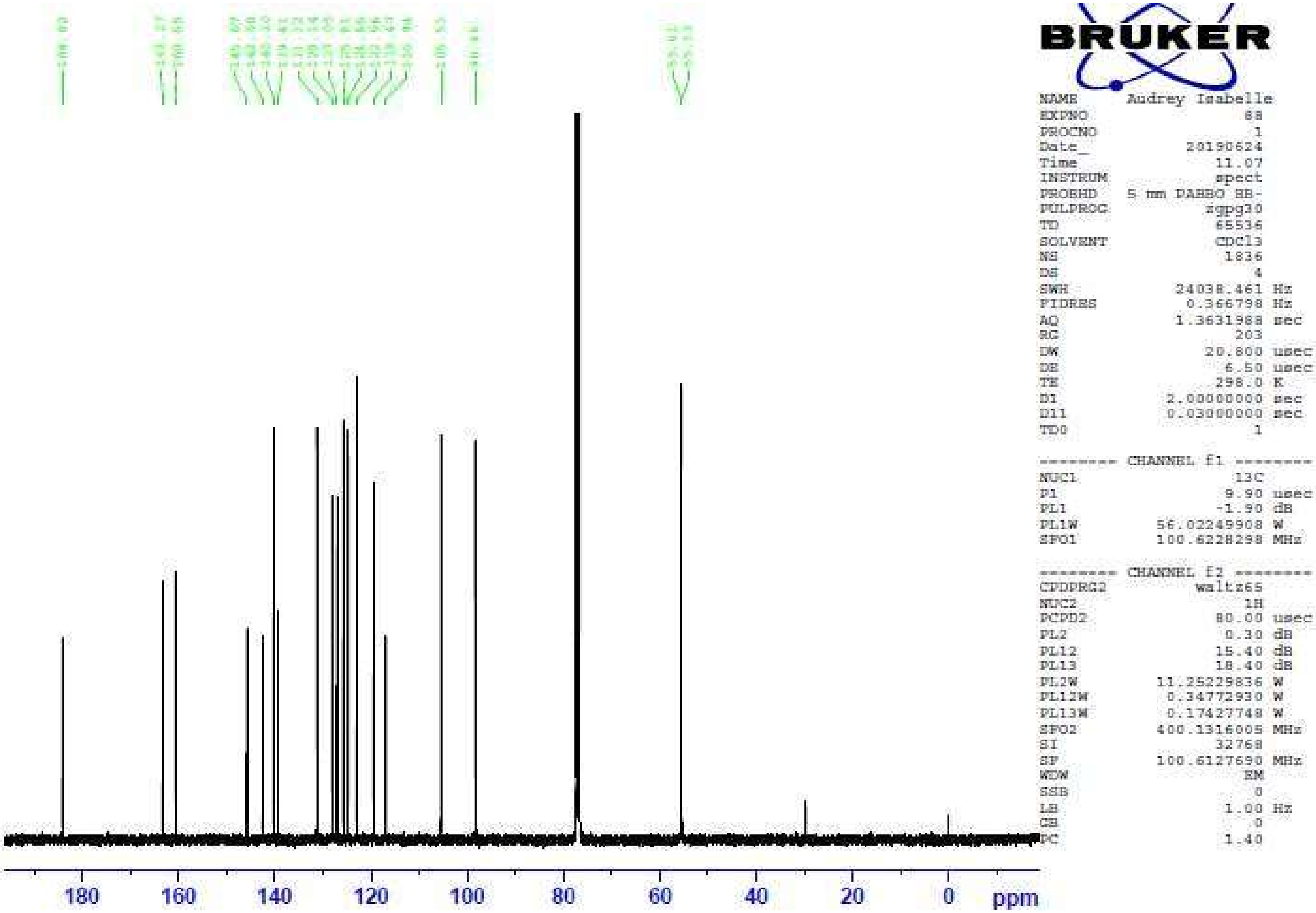
BRUKER
NAME Audrey Isabelle
EXPNO 88
PROCNO 1
Date_ 20190624
Time 11.07
INSTRUM spect
PROBHD 5 mm PABBO BB-
PULPROG zgpg30
TD 65536
SOLVENT CDCl3
NS 1836
DS 4
SWH 24038.461 Hz
FIDRES 0.366798 Hz
AQ 1.3631988 sec
RG 203
DW 20.800 usec
DE 6.50 usec
TE 298.0 K
D1 2.00000000 sec
D11 0.03000000 sec
TD0 1
======== CHANNEL f1 ========
NUC1 13C
P1 9.90 usec
PL1 -1.90 dB
PL1W 56.02249908 W
SFO1 100.6228298 MHz
======== CHANNEL f2 ========
CPDPRG2 waltz65
NUC2 1H
PCPD2 80.00 usec
PL2 0.30 dB
PL12 15.40 dB
PL13 18.40 dB
PL2W 11.25229836 W
PL12W 0.34772930 W
PL13W 0.17427748 W
SFO2 400.1316005 MHz
SI 32768
SF 100.6127690 MHz
WDW EM
SSB 0
LB 1.00 Hz
GB 0
PC 1.40
180 160 140 120 100 80 60 40 20 0 ppm

HRMS

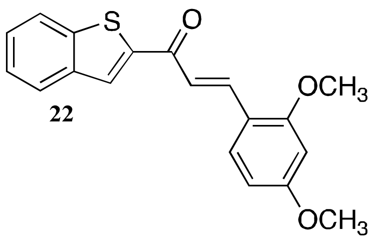

S
O
OCH3
OCH3

22

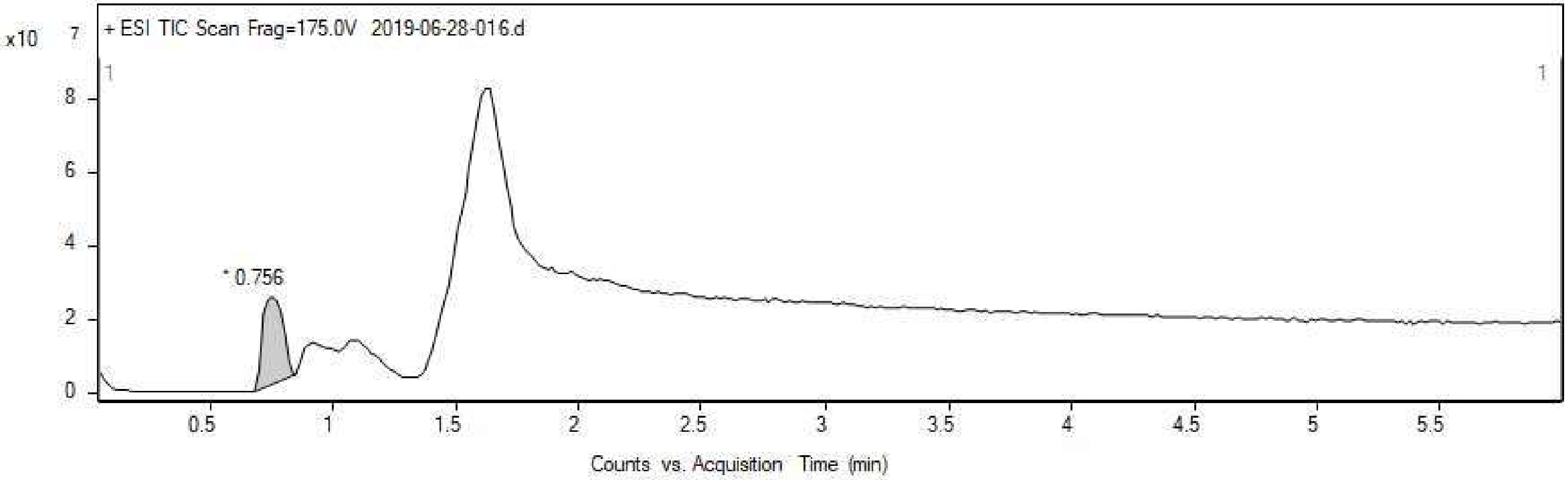

x10 7
+ ESI TIC Scan Frag=175.0V 2019-06-28-016.d
* 0.756
Counts vs. Acquisition Time (min)

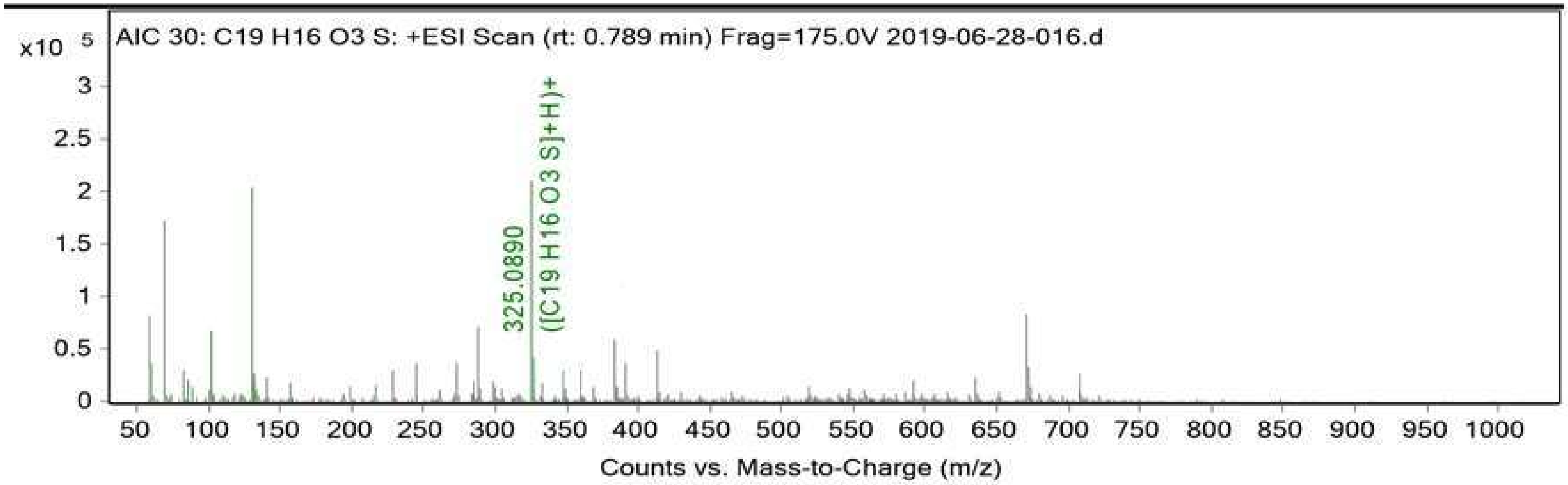

x10 5
AIC 30: C19 H16 O3 S: +ESI Scan (rt: 0.789 min) Frag=175.0V 2019-06-28-016.d
325.0890
([C19 H16 O3 S]+H)+
Counts vs. Mass-to-Charge (m/z)

^{1}H NMR

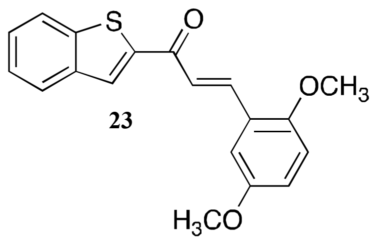

S
O
OCH3
H3CO

23

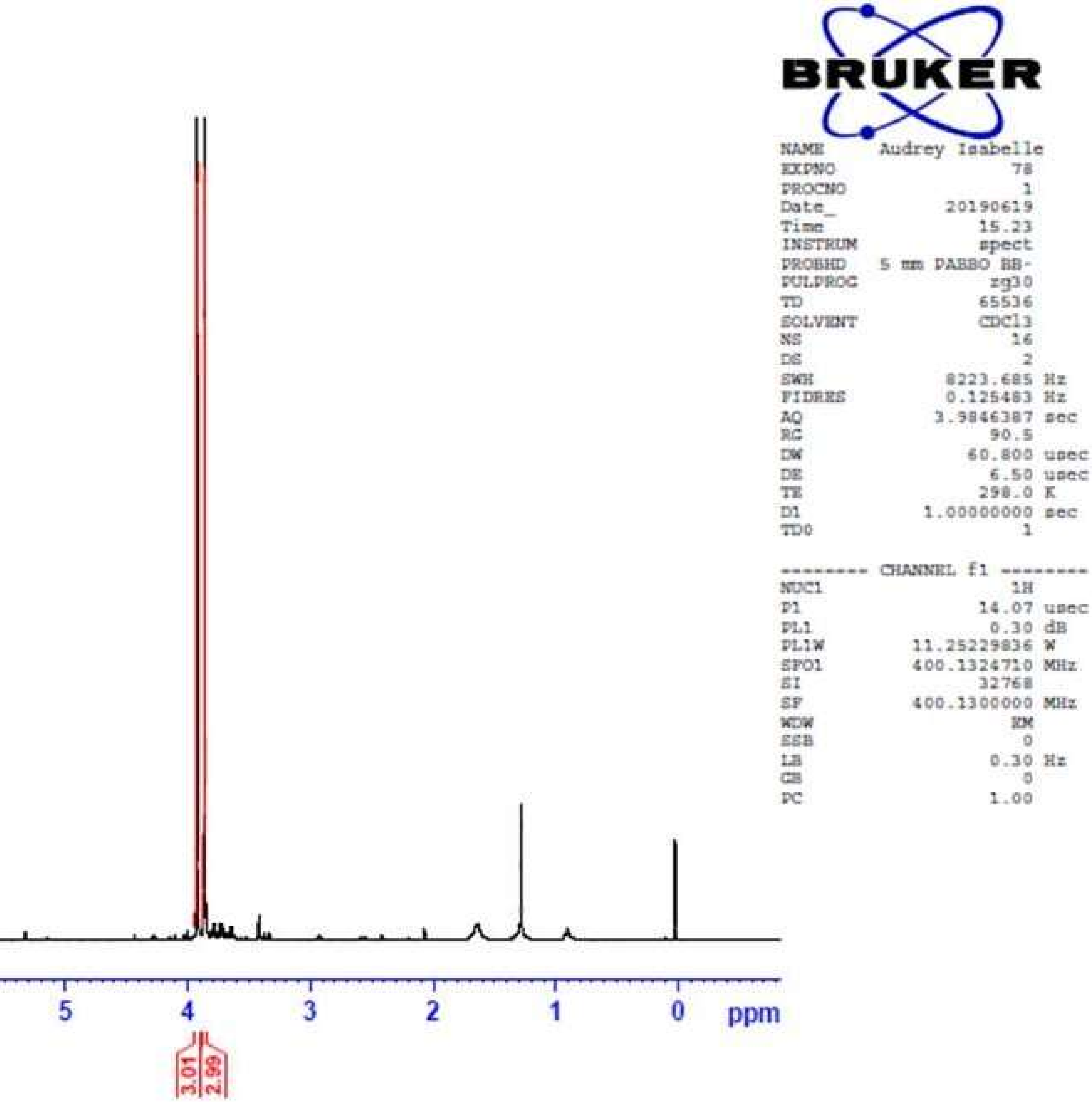

BRUKER
NAME Audrey Isabelle
EXPNO 78
PROCNO 1
Date_ 20190619
Time 15.23
INSTRUM spect
PROBHD 5 mm PABBO BB-
PULPROG zg30
TD 65536
SOLVENT CDCl3
NS 16
DS 2
SWH 8223.685 Hz
FIDRES 0.125483 Hz
AQ 3.9846387 sec
RG 90.5
DW 60.800 usec
DE 6.50 usec
TE 298.0 K
D1 1.00000000 sec
TD0 1
======== CHANNEL f1 ========
NUC1 1H
P1 14.07 usec
PL1 0.30 dB
PL1W 11.25229836 W
SFO1 400.1324710 MHz
SI 32768
SF 400.1300000 MHz
WDW EM
SSB 0
LB 0.30 Hz
GB 0
PC 1.00
8
7
6
5
4
3
2
1
0
ppm
1.00
1.00
2.06
1.07
2.17
1.07
1.03
1.02
3.01
2.99

^{13}C NMR

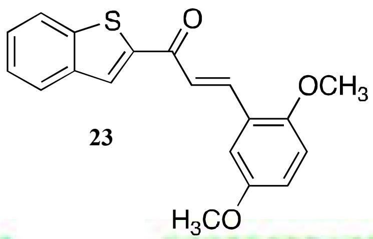

23

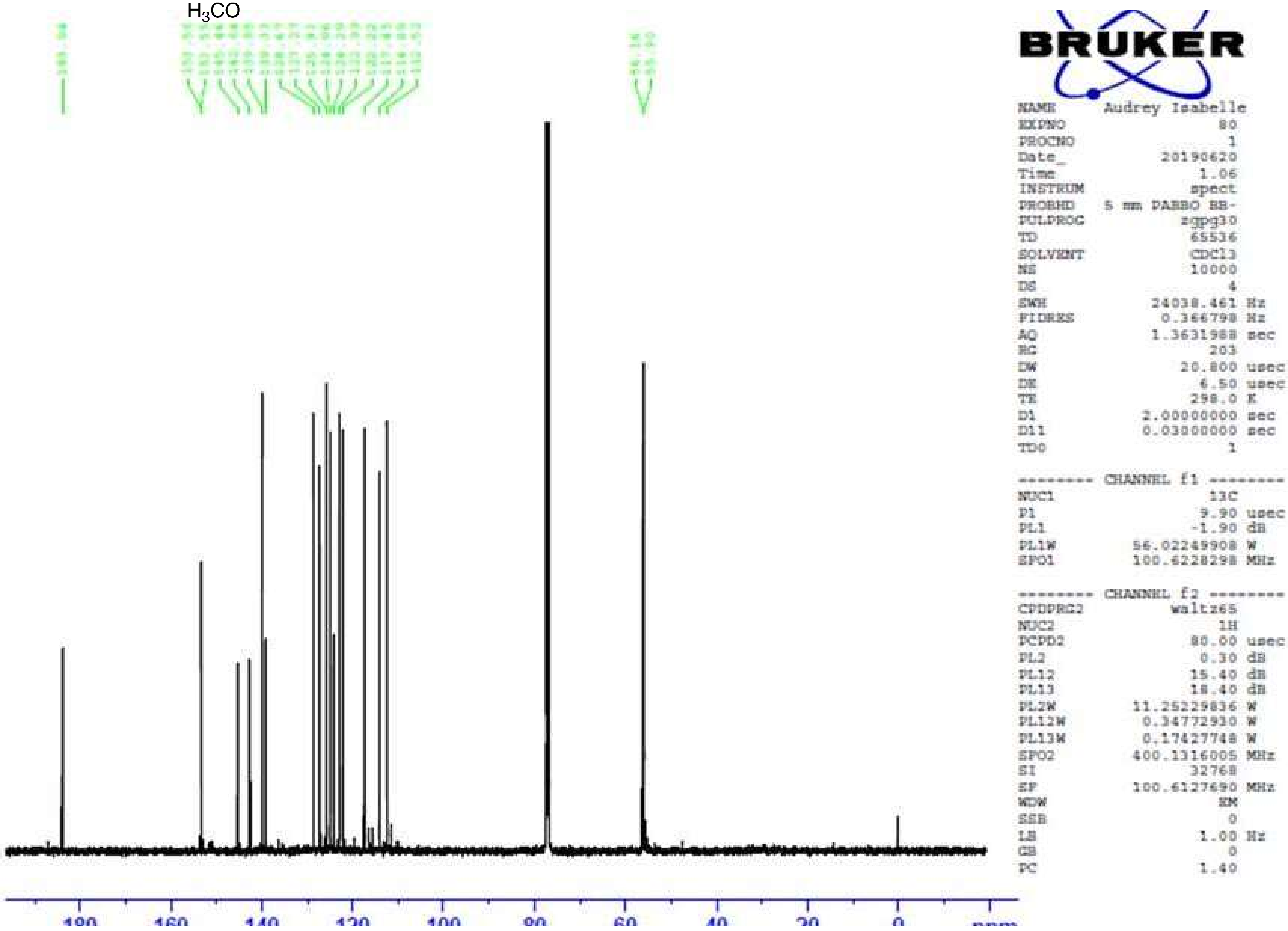
BRUKER
NAME Audrey Isabelle
EXPNO 80
PROCNO 1
Date_ 20190620
Time 1.06
INSTRUM spect
PROBHD 5 mm PABBO BB-
PULPROG zgpg30
TD 65536
SOLVENT CDCl3
NS 10000
DS 4
SWH 24038.461 Hz
FIDRES 0.366798 Hz
AQ 1.3631988 sec
RG 203
DW 20.800 usec
DE 6.50 usec
TE 298.0 K
D1 2.00000000 sec
D11 0.03000000 sec
TD0 1
======== CHANNEL f1 ========
NUC1 13C
P1 9.90 usec
PL1 -1.90 dB
PL1W 56.02249908 W
SFO1 100.6228298 MHz
======== CHANNEL f2 ========
CPDPRG2 waltz65
NUC2 1H
PCPD2 80.00 usec
PL2 0.30 dB
PL12 15.40 dB
PL13 18.40 dB
PL2W 11.25229836 W
PL12W 0.34772930 W
PL13W 0.17427748 W
SFO2 400.1316005 MHz
SI 32768
SF 100.6127690 MHz
WDW EM
SSB 0
LB 1.00 Hz
GB 0
PC 1.40
180
160
140
120
100
80
60
40
20
0
ppm

HRMS

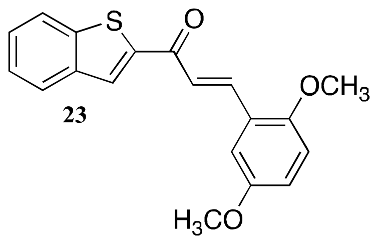
S
O
OCH3
H3CO

23

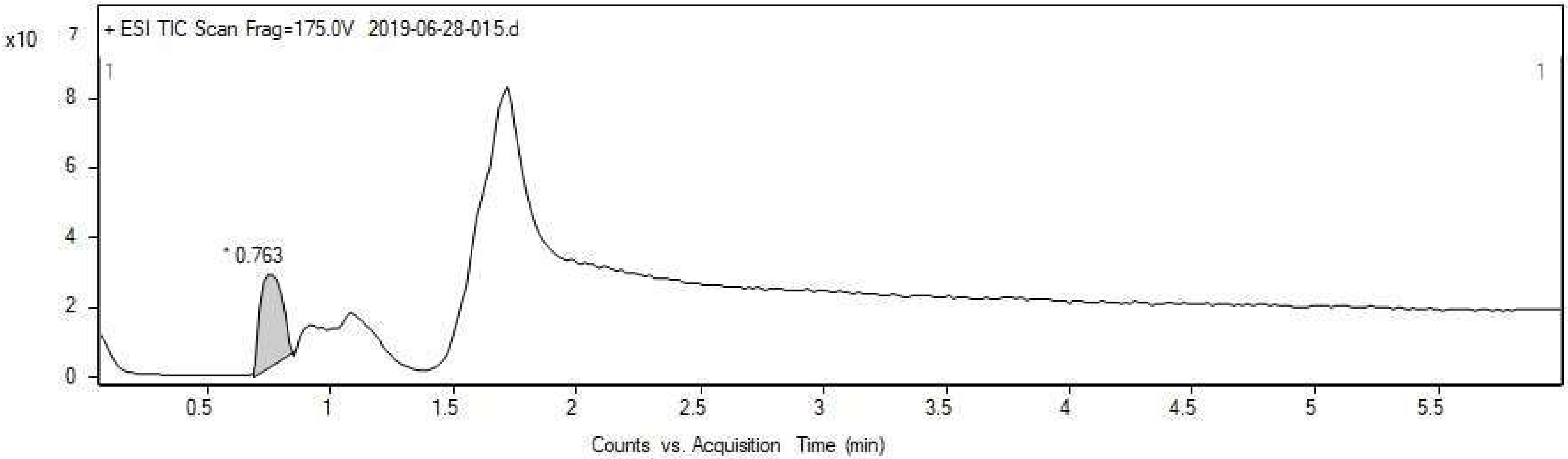
x10 7
+ ESI TIC Scan Frag=175.0V 2019-06-28-015.d
* 0.763
Counts vs. Acquisition Time (min)

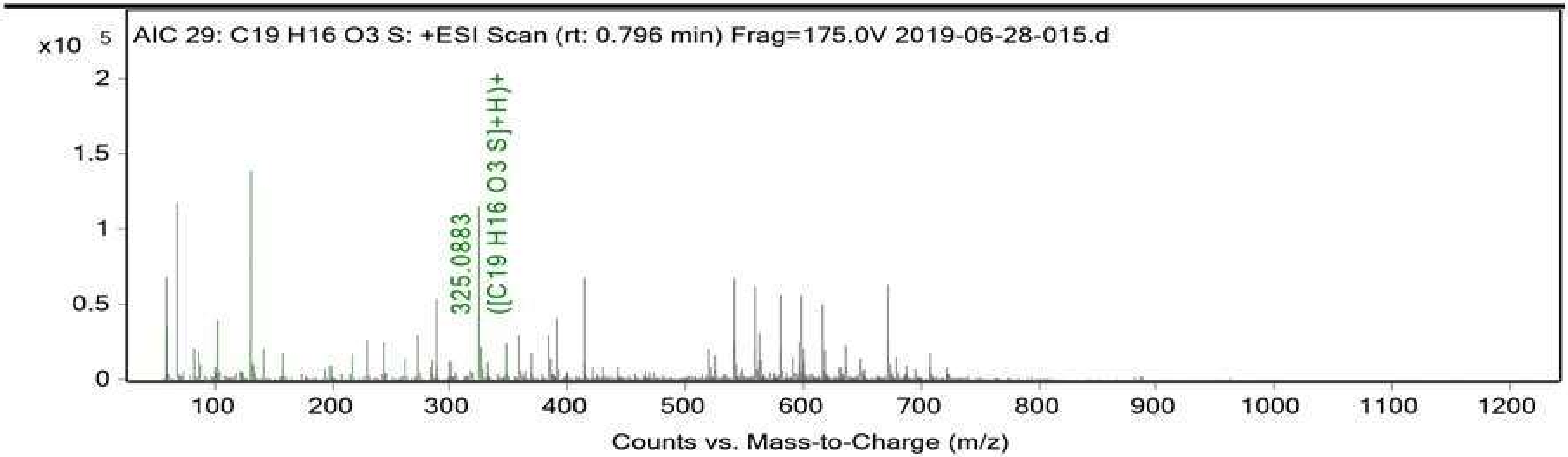
x10 5
AIC 29: C19 H16 O3 S: +ESI Scan (rt: 0.796 min) Frag=175.0V 2019-06-28-015.d
325.0883
([C19 H16 O3 S]+H)+
Counts vs. Mass-to-Charge (m/z)

^{1}H NMR

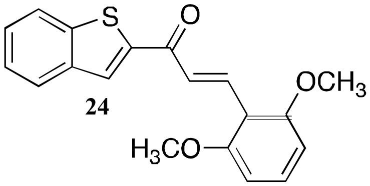

24

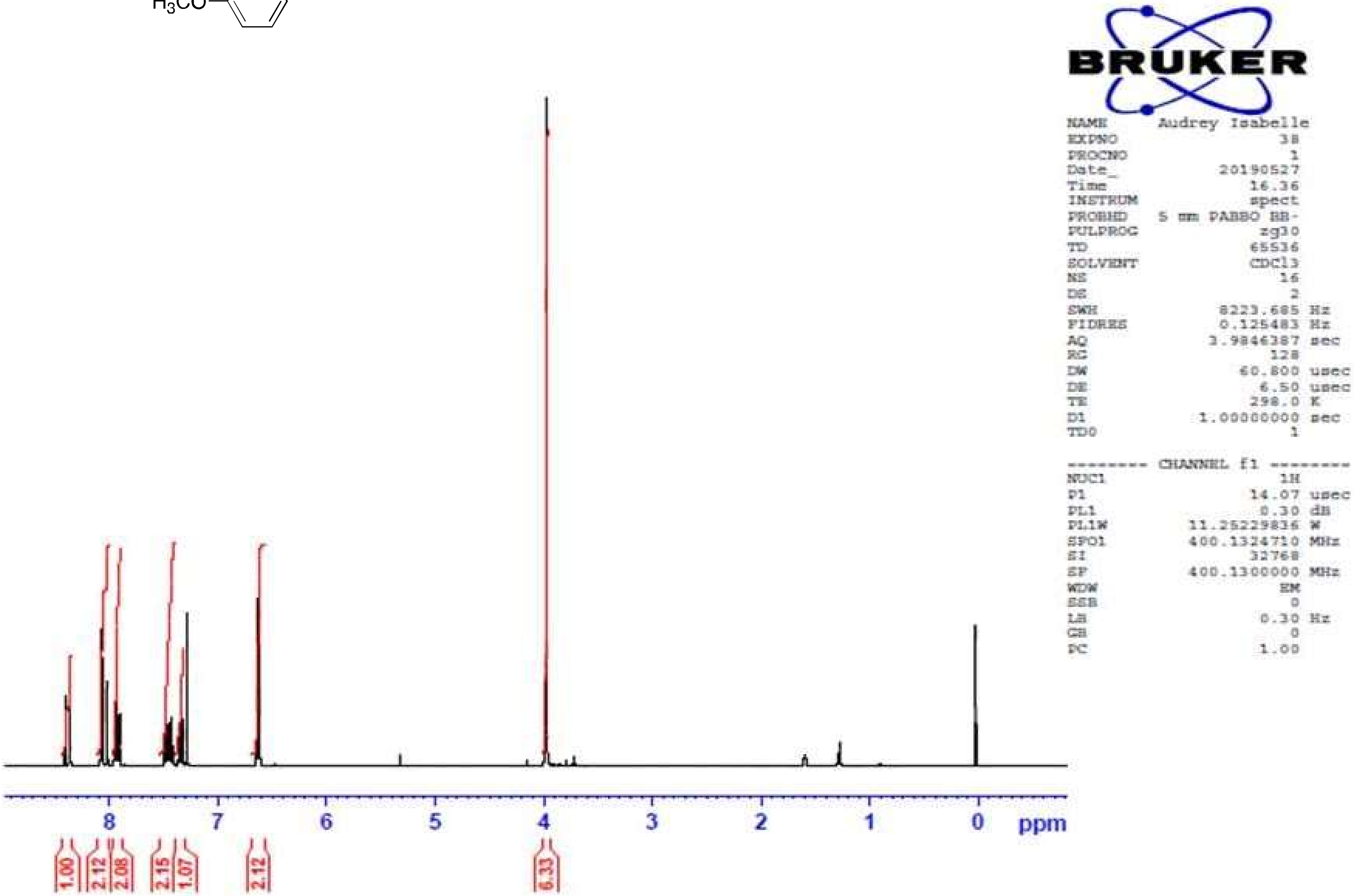
BRUKER
NAME Audrey Isabelle
EXPNO 38
PROCNO 1
Date_ 20190527
Time 16.36
INSTRUM spect
PROBHD 5 mm PABBO BB-
PULPROG zg30
TD 65536
SOLVENT CDCl3
NS 16
DS 2
SWH 8223.685 Hz
FIDRES 0.125483 Hz
AQ 3.9846387 sec
RG 128
DW 60.800 usec
DE 6.50 usec
TE 298.0 K
D1 1.00000000 sec
TD0 1
======== CHANNEL f1 ========
NUC1 1H
P1 14.07 usec
PL1 0.30 dB
PL1W 11.25229836 W
SFO1 400.1324710 MHz
SI 32768
SF 400.1300000 MHz
WDW EM
SSB 0
LB 0.30 Hz
GB 0
PC 1.00
8 7 6 5 4 3 2 1 0 ppm
1.00 2.12 2.08 2.15 1.07 2.12 6.33

^{13}C NMR

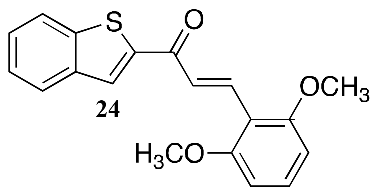

24

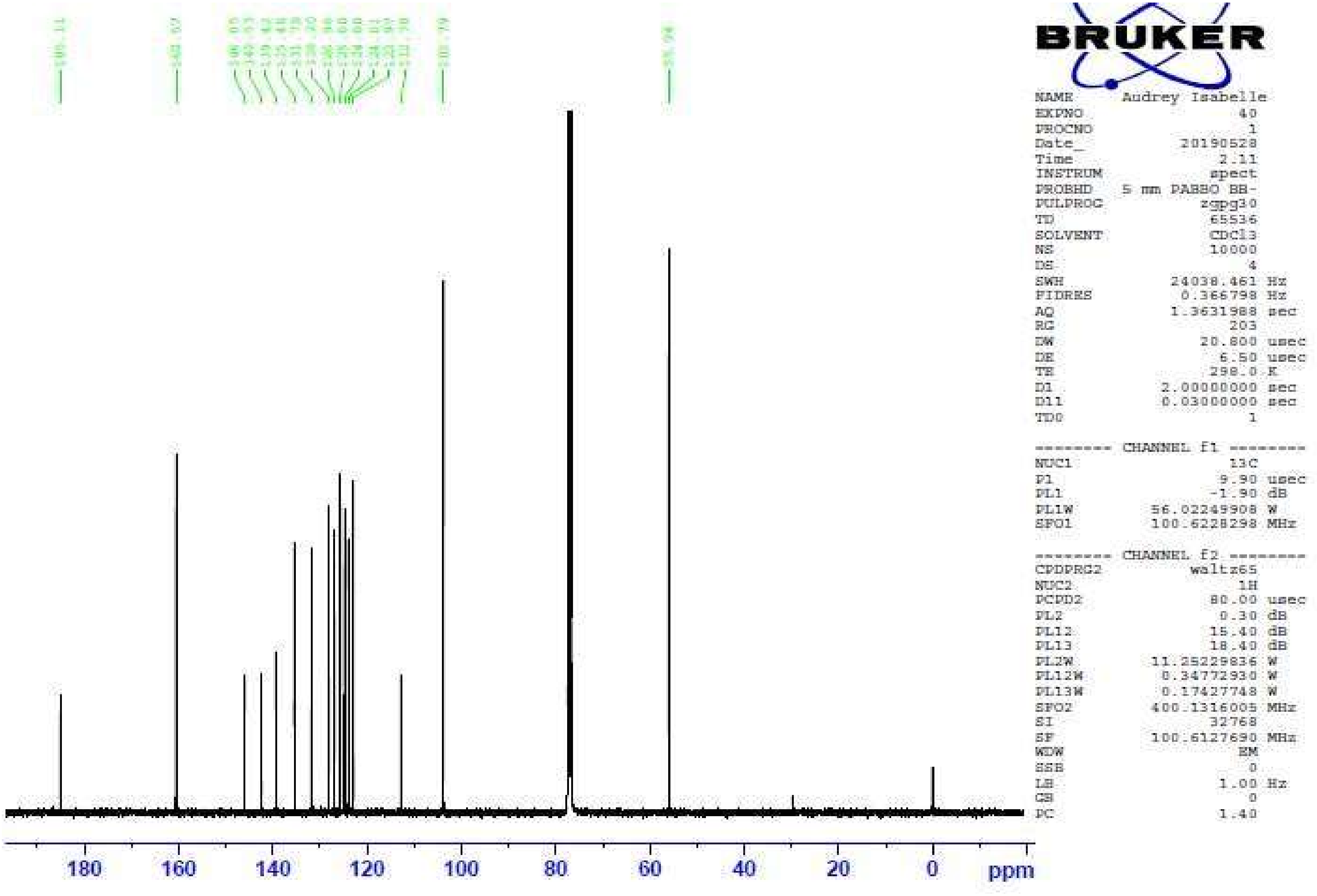
BRUKER
NAME Audrey Isabelle
EXPNO 40
PROCNO 1
Date_ 20190528
Time 2.11
INSTRUM spect
PROBHD 5 mm PABBO BB-
PULPROG zgpg30
TD 65536
SOLVENT CDCl3
NS 10000
DS 4
SWH 24038.461 Hz
FIDRES 0.366798 Hz
AQ 1.3631988 sec
RG 203
DW 20.800 usec
DE 6.50 usec
TE 298.0 K
D1 2.00000000 sec
D11 0.03000000 sec
TD0 1
======== CHANNEL f1 ========
NUC1 13C
P1 9.90 usec
PL1 -1.90 dB
PL1W 56.02249908 W
SFO1 100.6228298 MHz
======== CHANNEL f2 ========
CPDPRG2 waltz65
NUC2 1H
PCPD2 80.00 usec
PL2 0.30 dB
PL12 15.40 dB
PL13 18.40 dB
PL2W 11.25229836 W
PL12W 0.34772930 W
PL13W 0.17427748 W
SFO2 400.1316005 MHz
SI 32768
SF 100.6127690 MHz
WDW EM
SSB 0
LB 1.00 Hz
GB 0
PC 1.40
180
160
140
120
100
80
60
40
20
0
ppm

HRMS

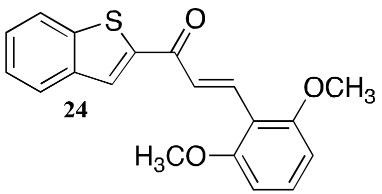

24

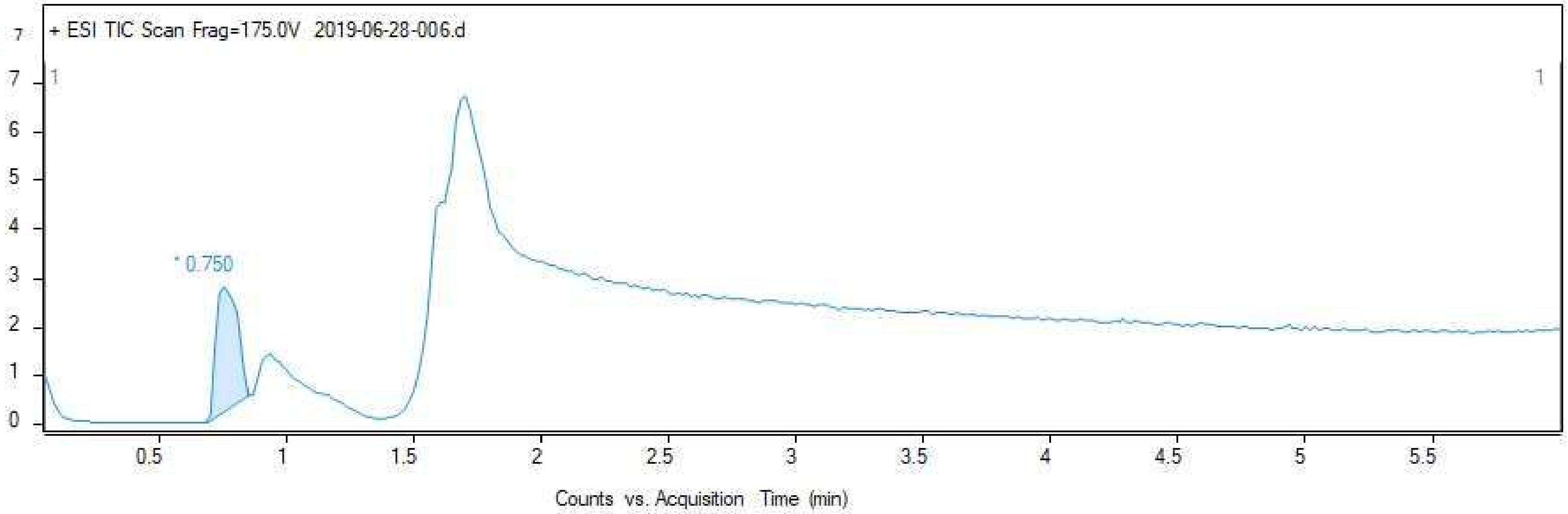
+ ESI TIC Scan Frag=175.0V 2019-06-28-006.d
* 0.750
Counts vs. Acquisition Time (min)

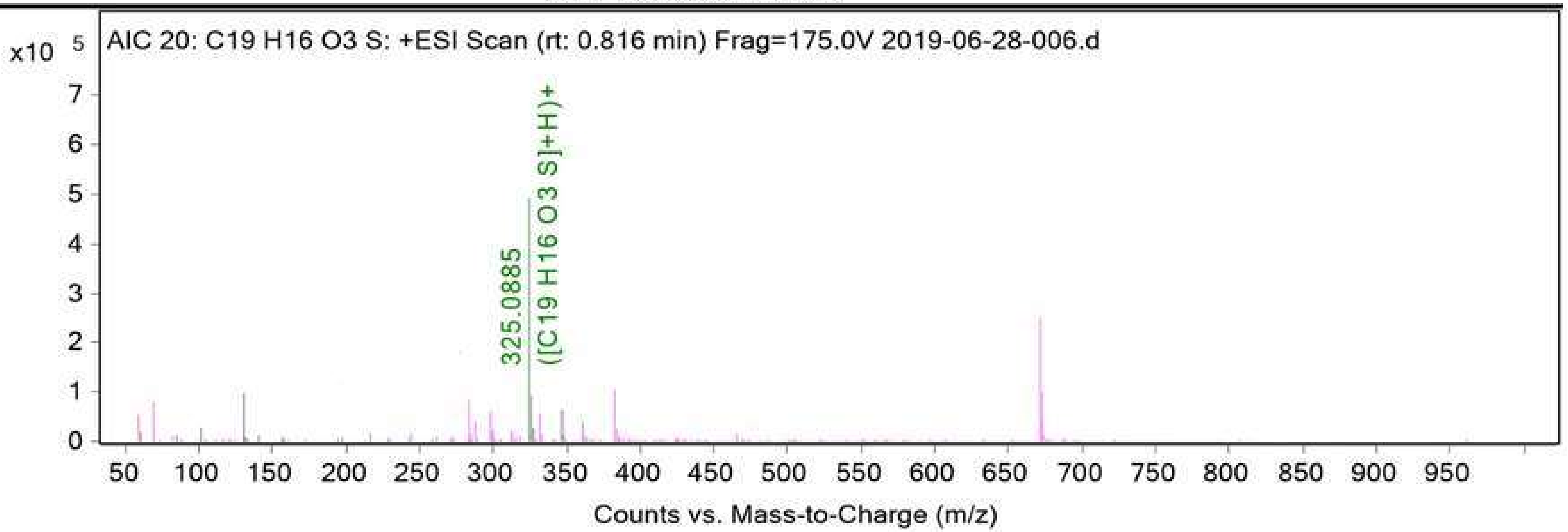
x10 5
AIC 20: C19 H16 O3 S: +ESI Scan (rt: 0.816 min) Frag=175.0V 2019-06-28-006.d
325.0885
([C19 H16 O3 S]+H)+
Counts vs. Mass-to-Charge (m/z)

^{1}H NMR

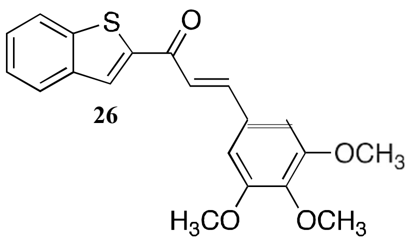

26

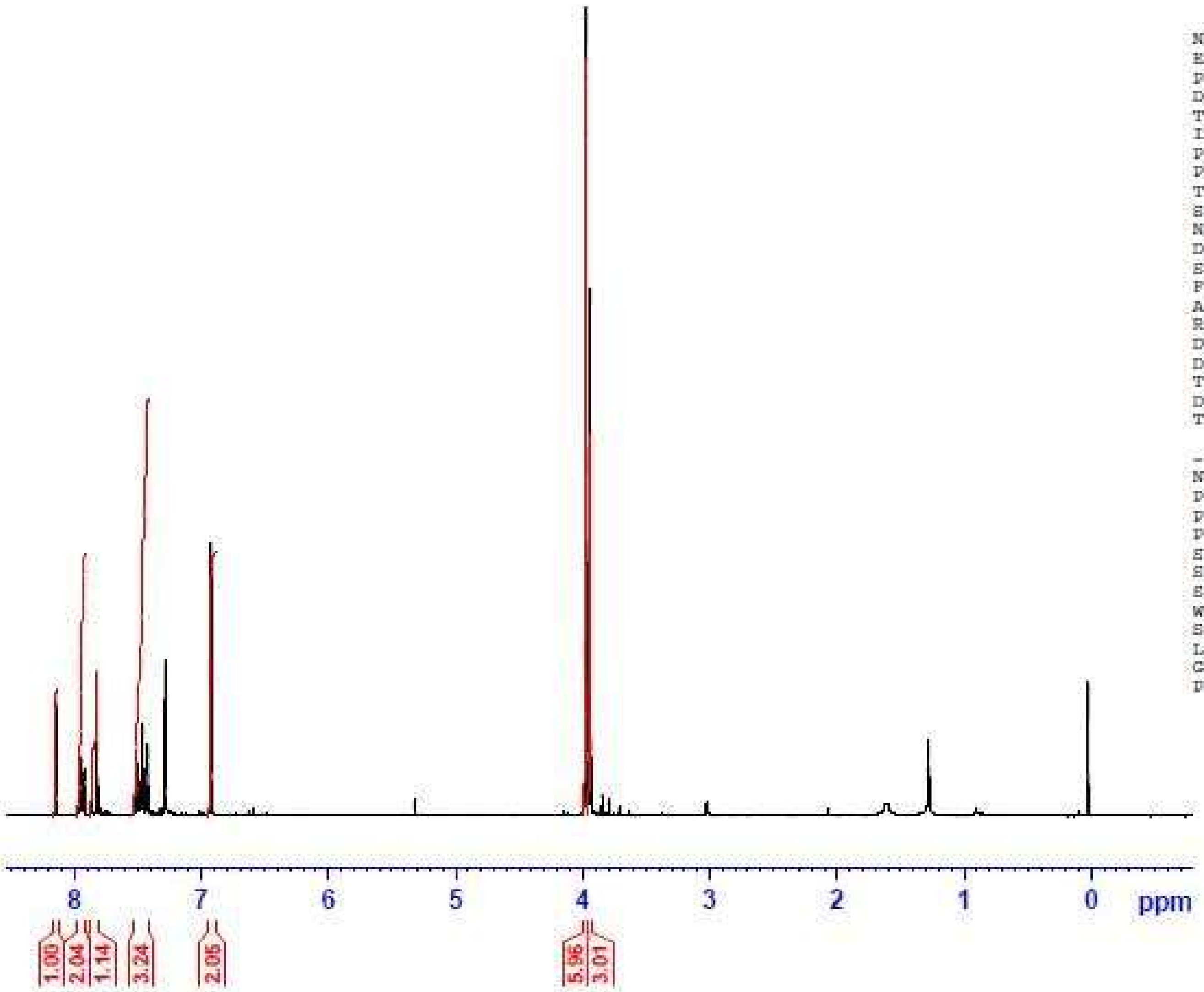

BRUKER

NAME	Audrey Isabelle
EXPNO	62
PROCNO	1
Date_	20190612
Time	8.58
INSTRUM	spect
PROBHD	5 mm PABBO BB-
PULPROG	zg30
TD	65536
SOLVENT	CDCl3
NS	16
DS	2
SWH	8223.685 Hz
FIDRES	0.125483 Hz
AQ	3.9846387 sec
RG	114
DW	60.800 usec
DE	6.50 usec
TE	298.0 K
D1	1.00000000 sec
TD0	1
======== CHANNEL f1 ========	
NUC1	1H
P1	14.07 usec
PL1	0.30 dB
PL1W	11.25229836 W
SFO1	400.1324710 MHz
SI	32768
SF	400.1300000 MHz
WDW	EM
SSB	0
LB	0.30 Hz
GB	0
PC	1.00

^{13}C NMR

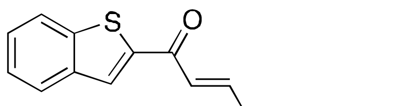
S
O
26
OCH3
H3CO
OCH3

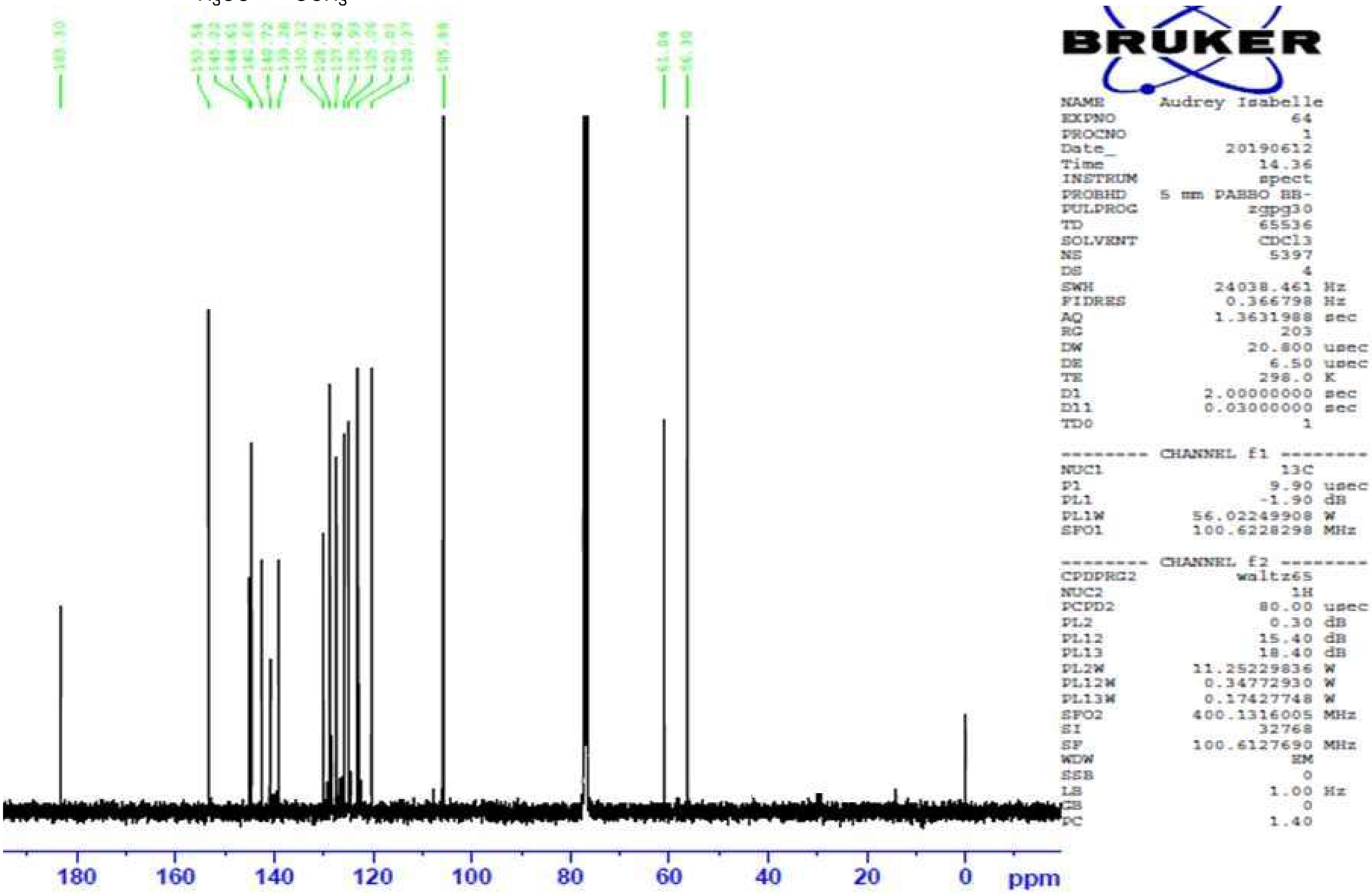
BRUKER
NAME Audrey Isabelle
EXPNO 64
PROCNO 1
Date_ 20190612
Time 14.36
INSTRUM spect
PROBHD 5 mm PABBO BB-
PULPROG zgpg30
TD 65536
SOLVENT CDCl3
NS 5397
DS 4
SWH 24038.461 Hz
FIDRES 0.366798 Hz
AQ 1.3631988 sec
RG 203
DW 20.800 usec
DE 6.50 usec
TE 298.0 K
D1 2.00000000 sec
D11 0.03000000 sec
TD0 1
======== CHANNEL f1 ========
NUC1 13C
P1 9.90 usec
PL1 -1.90 dB
PL1W 56.02249908 W
SFO1 100.6228298 MHz
======== CHANNEL f2 ========
CPDPRG2 waltz65
NUC2 1H
PCPD2 80.00 usec
PL2 0.30 dB
PL12 15.40 dB
PL13 18.40 dB
PL2W 11.25229836 W
PL12W 0.34772930 W
PL13W 0.17427748 W
SFO2 400.1316005 MHz
SI 32768
SF 100.6127690 MHz
WDW EM
SSB 0
LB 1.00 Hz
GB 0
PC 1.40
180
160
140
120
100
80
60
40
20
0
ppm

HRMS

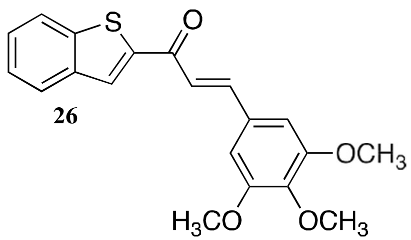
S
O
OCH3
H3CO
OCH3

26

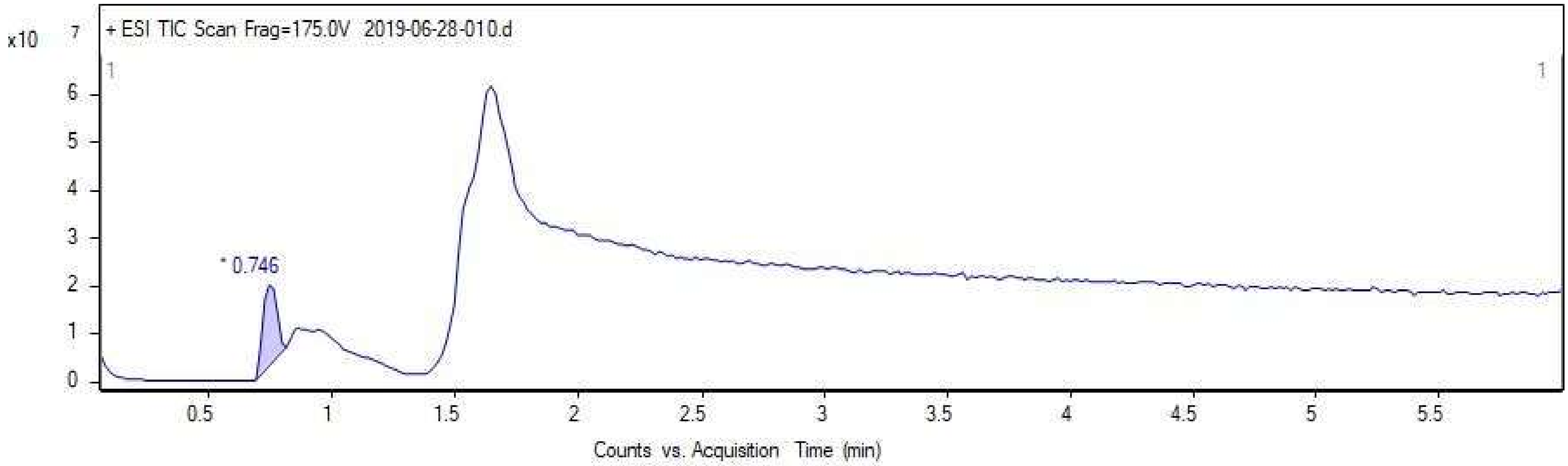
x10 7
+ ESI TIC Scan Frag=175.0V 2019-06-28-010.d
* 0.746
Counts vs. Acquisition Time (min)

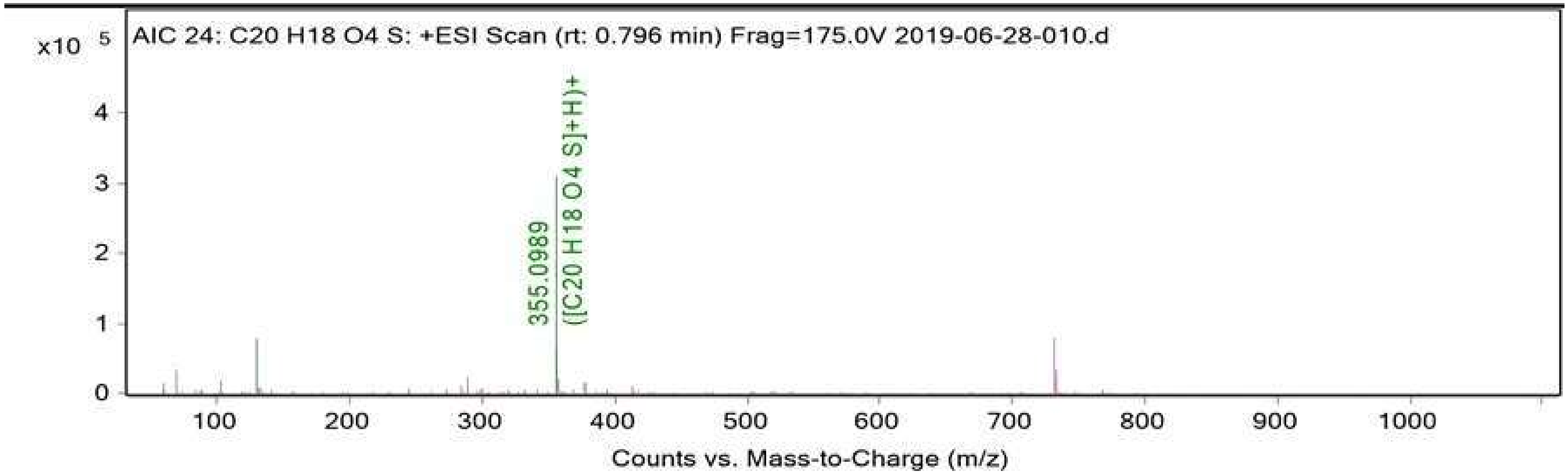
x10 5
AIC 24: C20 H18 O4 S: +ESI Scan (rt: 0.796 min) Frag=175.0V 2019-06-28-010.d
355.0989
([C20 H18 O4 S]+H)+
Counts vs. Mass-to-Charge (m/z)

^{1}H NMR

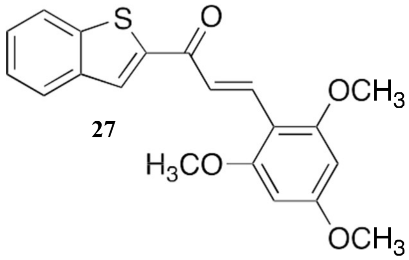

27

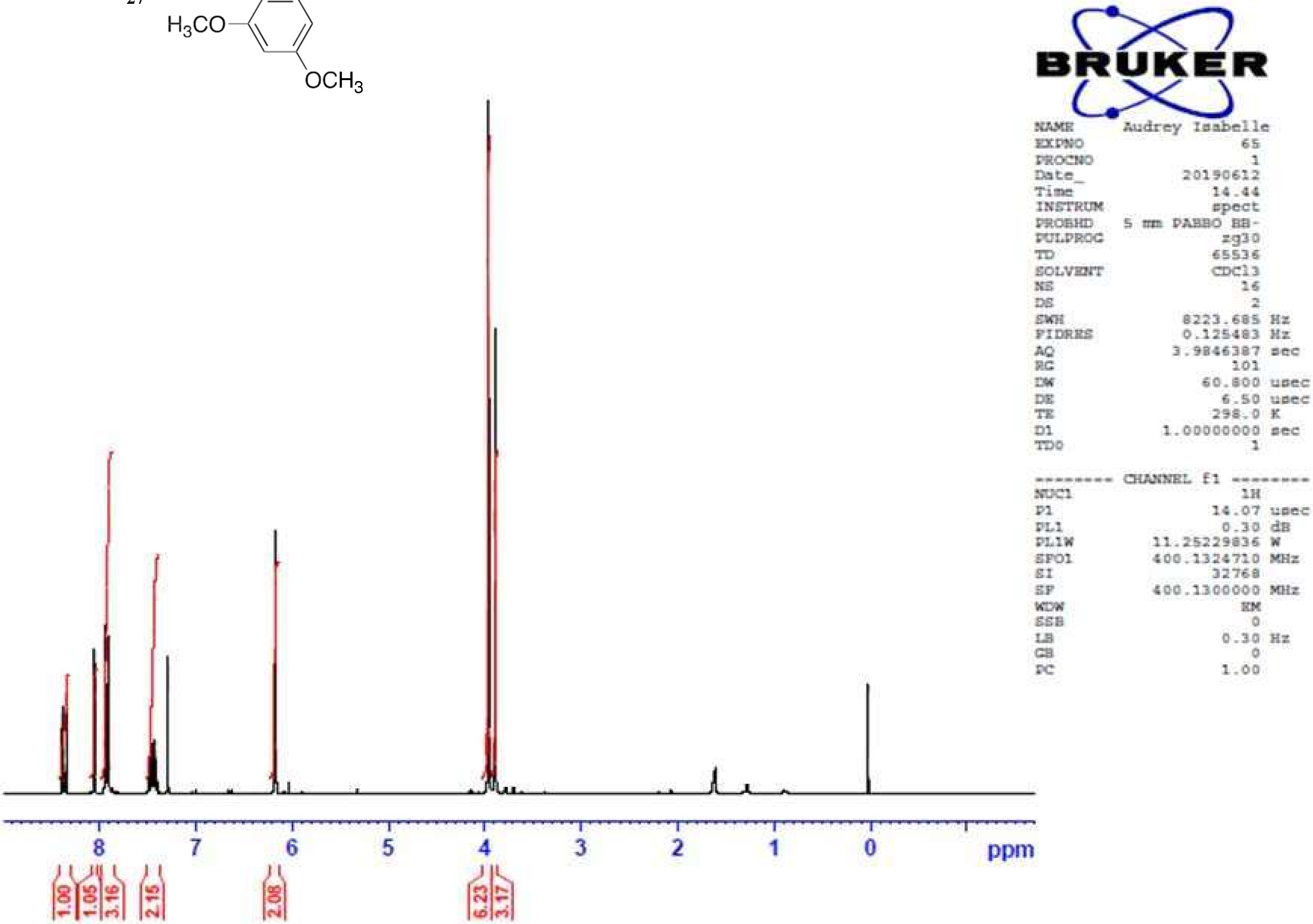
NAME Audrey Isabelle
EXPNO 65
PROCNO 1
Date_ 20190612
Time 14.44
INSTRUM spect
PROBHD 5 mm PABBO BB-
PULPROG zg30
TD 65536
SOLVENT CDCl3
NS 16
DS 2
SWH 8223.685 Hz
FIDRES 0.125483 Hz
AQ 3.9846387 sec
RG 101
DW 60.800 usec
DE 6.50 usec
TE 298.0 K
D1 1.00000000 sec
TD0 1
======== CHANNEL f1 ========
NUC1 1H
P1 14.07 usec
PL1 0.30 dB
PL1W 11.25229836 W
SFO1 400.1324710 MHz
SI 32768
SF 400.1300000 MHz
WDW EM
SSB 0
LB 0.30 Hz
GB 0
PC 1.00
8 7 6 5 4 3 2 1 0 ppm
1.00 1.05 3.16 2.15 2.08 6.23 3.17

^{13}C NMR

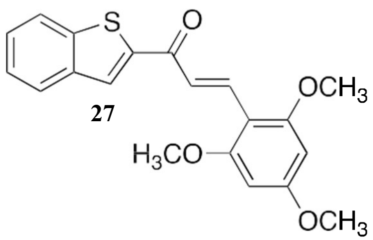

27

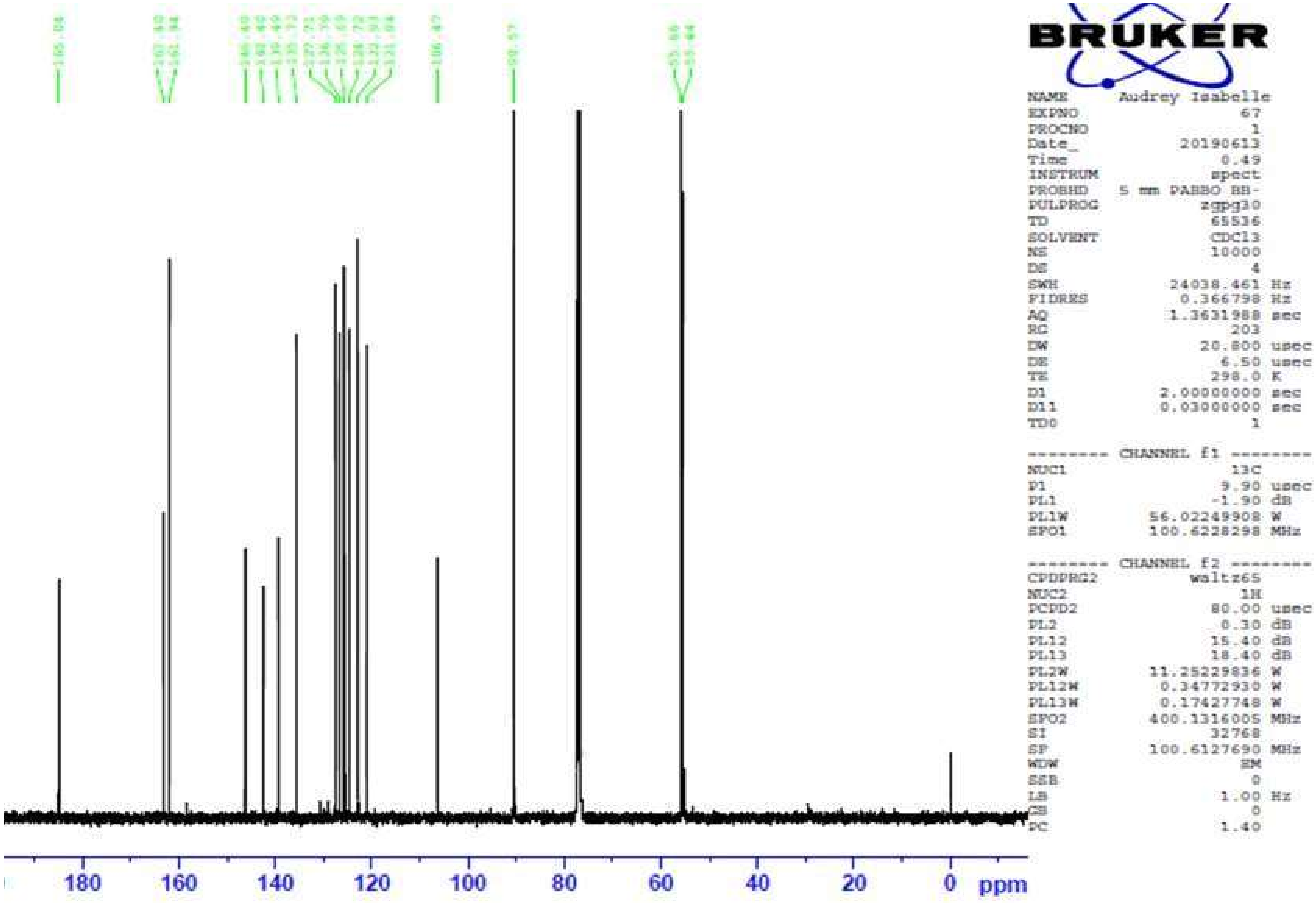
BRUKER
NAME Audrey Isabelle
EXPNO 67
PROCNO 1
Date_ 20190613
Time 0.49
INSTRUM spect
PROBHD 5 mm PABBO BB-
PULPROG zgpg30
TD 65536
SOLVENT CDCl3
NS 10000
DS 4
SWH 24038.461 Hz
FIDRES 0.366798 Hz
AQ 1.3631988 sec
RG 203
DW 20.800 usec
DE 6.50 usec
TE 298.0 K
D1 2.00000000 sec
D11 0.03000000 sec
TD0 1
======== CHANNEL f1 ========
NUC1 13C
P1 9.90 usec
PL1 -1.90 dB
PL1W 56.02249908 W
SFO1 100.6228298 MHz
======== CHANNEL f2 ========
CPDPRG2 waltz65
NUC2 1H
PCPD2 80.00 usec
PL2 0.30 dB
PL12 15.40 dB
PL13 18.40 dB
PL2W 11.25229836 W
PL12W 0.34772930 W
PL13W 0.17427748 W
SFO2 400.1316005 MHz
SI 32768
SF 100.6127690 MHz
WDW EM
SSB 0
LB 1.00 Hz
GB 0
PC 1.40
180
160
140
120
100
80
60
40
20
0
ppm

HRMS

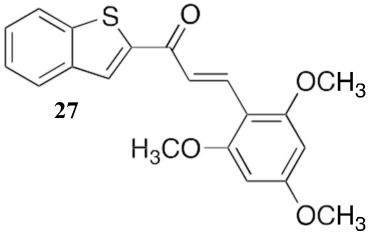
S
O
OCH3
H3CO
OCH3

27

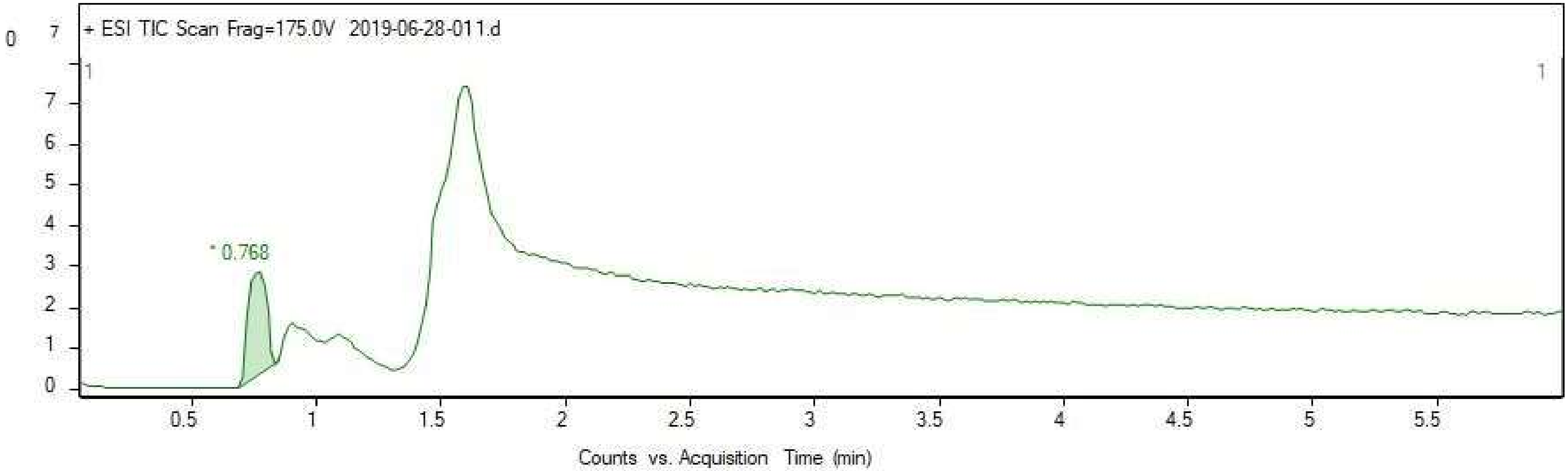
0 7
+ ESI TIC Scan Frag=175.0V 2019-06-28-011.d
* 0.768
Counts vs. Acquisition Time (min)

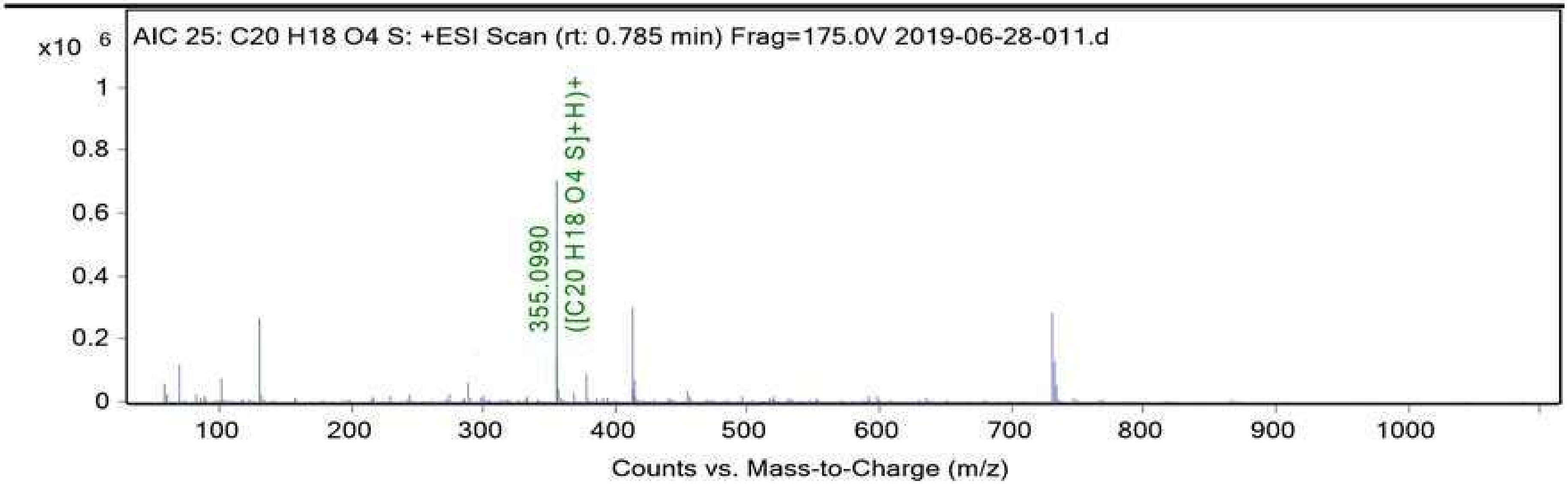
x10 6
AIC 25: C20 H18 O4 S: +ESI Scan (rt: 0.785 min) Frag=175.0V 2019-06-28-011.d
355.0990
([C20 H18 O4 S]+H)+
Counts vs. Mass-to-Charge (m/z)

^{1}H NMR

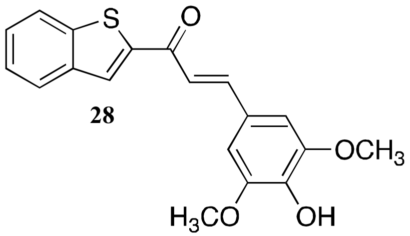

28

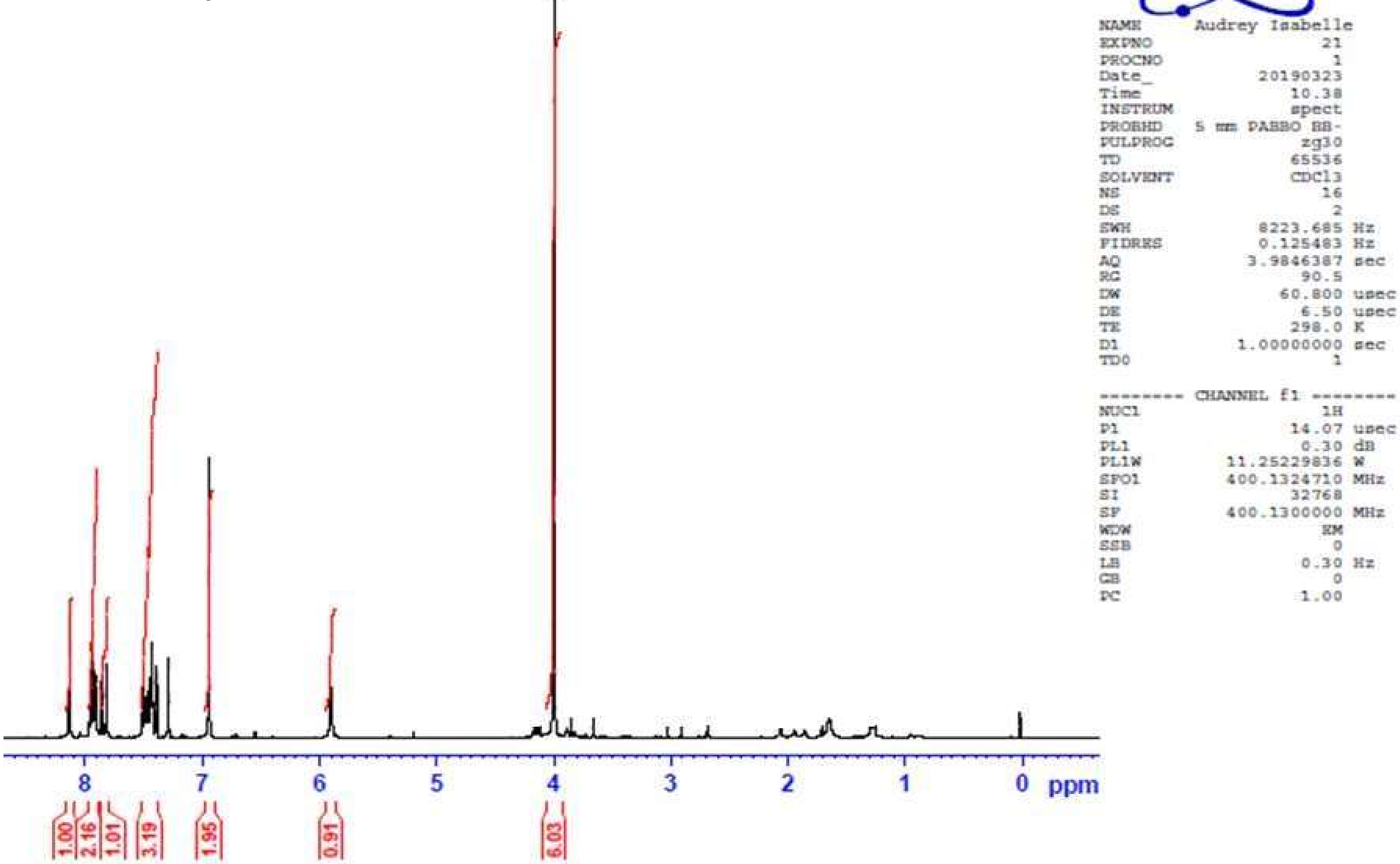

BRUKER
NAME Audrey Isabelle
EXPNO 21
PROCNO 1
Date_ 20190323
Time 10.38
INSTRUM spect
PROBHD 5 mm PABBO BB-
PULPROG zg30
TD 65536
SOLVENT CDCl3
NS 16
DS 2
SWH 8223.685 Hz
FIDRES 0.125483 Hz
AQ 3.9846387 sec
RG 90.5
DW 60.800 usec
DE 6.50 usec
TE 298.0 K
D1 1.00000000 sec
TD0 1
======== CHANNEL f1 ========
NUC1 1H
P1 14.07 usec
PL1 0.30 dB
PL1W 11.25229836 W
SFO1 400.1324710 MHz
SI 32768
SF 400.1300000 MHz
WDW EM
SSB 0
LB 0.30 Hz
GB 0
PC 1.00
8 7 6 5 4 3 2 1 0 ppm
1.00 2.16 1.01 3.19 1.95 0.91 6.03

^{13}C NMR

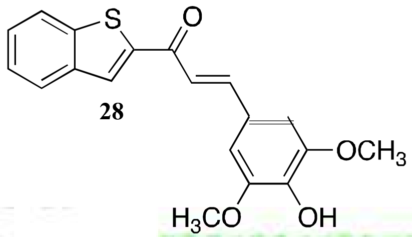

S
O
OCH3
H3CO
OH

28

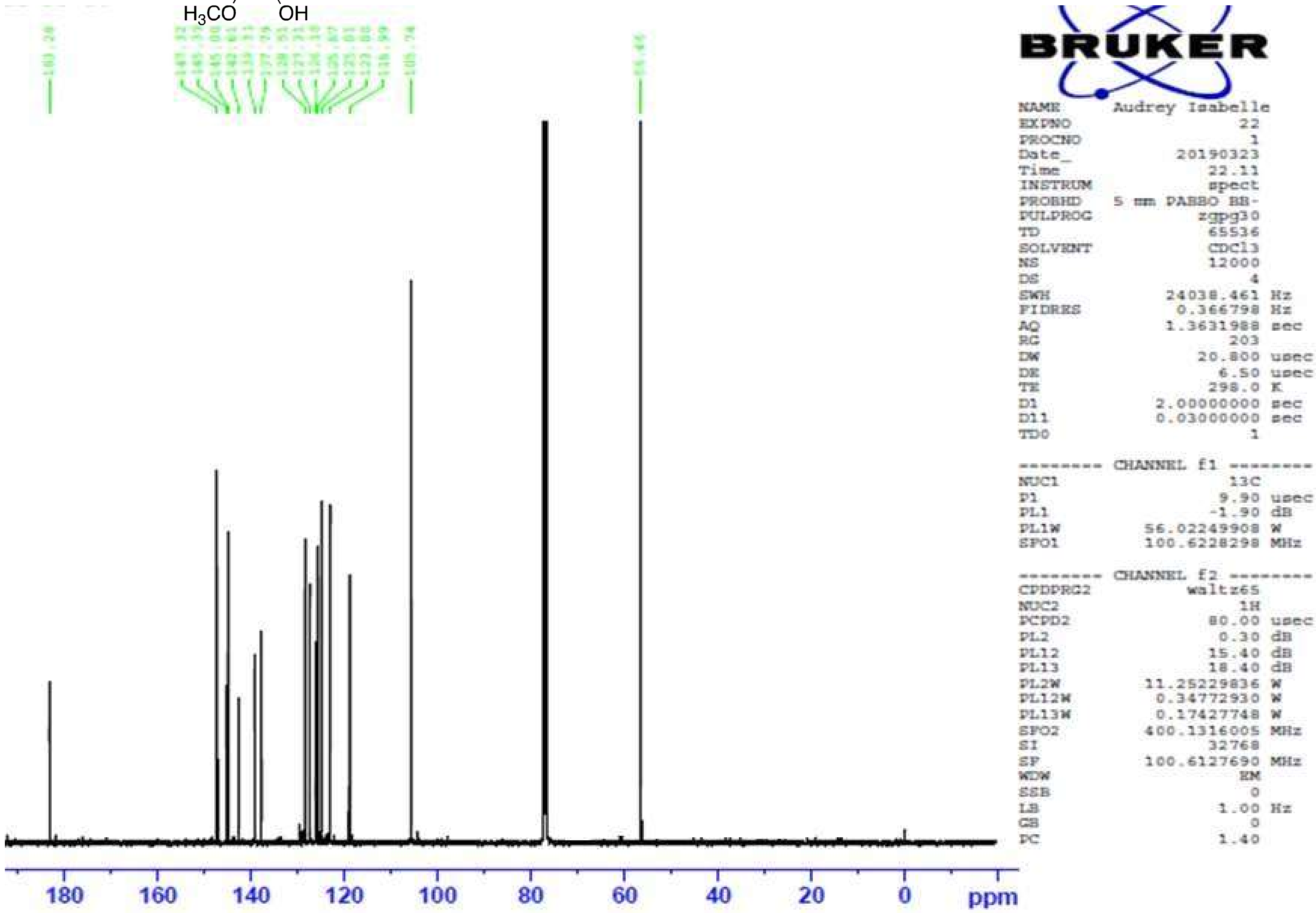

BRUKER
NAME Audrey Isabelle
EXPNO 22
PROCNO 1
Date_ 20190323
Time 22.11
INSTRUM spect
PROBHD 5 mm PABBO BB-
PULPROG zgpg30
TD 65536
SOLVENT CDCl3
NS 12000
DS 4
SWH 24038.461 Hz
FIDRES 0.366798 Hz
AQ 1.3631988 sec
RG 203
DW 20.800 usec
DE 6.50 usec
TE 298.0 K
D1 2.00000000 sec
D11 0.03000000 sec
TD0 1
======== CHANNEL f1 ========
NUC1 13C
P1 9.90 usec
PL1 -1.90 dB
PL1W 56.02249908 W
SFO1 100.6228298 MHz
======== CHANNEL f2 ========
CPDPRG2 waltz65
NUC2 1H
PCPD2 80.00 usec
PL2 0.30 dB
PL12 15.40 dB
PL13 18.40 dB
PL2W 11.25229836 W
PL12W 0.34772930 W
PL13W 0.17427748 W
SFO2 400.1316005 MHz
SI 32768
SF 100.6127690 MHz
WDW EM
SSB 0
LB 1.00 Hz
GB 0
PC 1.40
180
160
140
120
100
80
60
40
20
0
ppm

HRMS

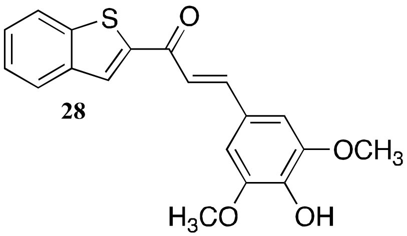

28

Compound Label	RT	Algorithm	Mass
Cpd 1: C19 H16 O4 S	0.12	Find By Formula	340.0769

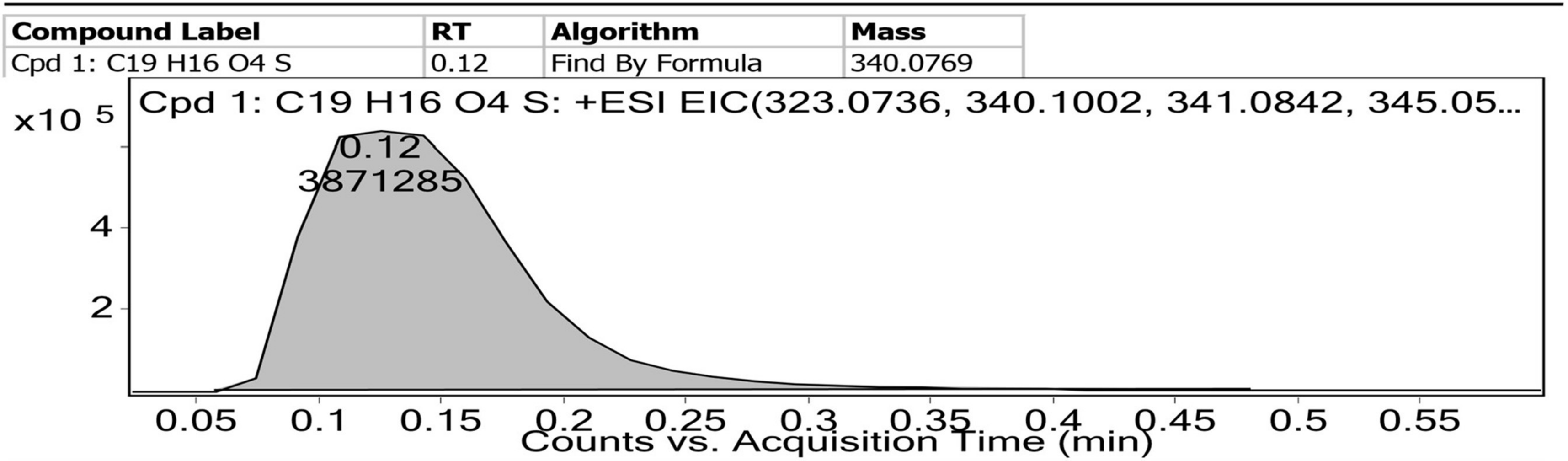

MS Spectrum

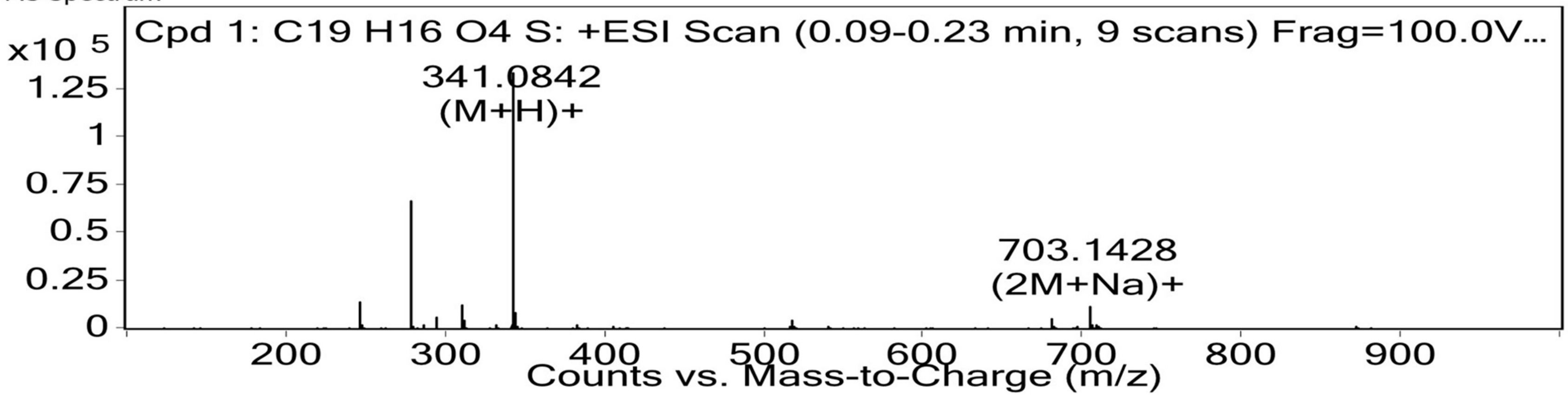

^{1}H NMR

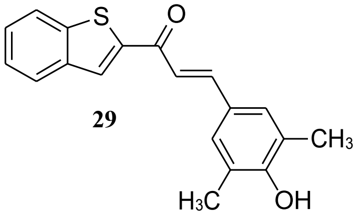

29

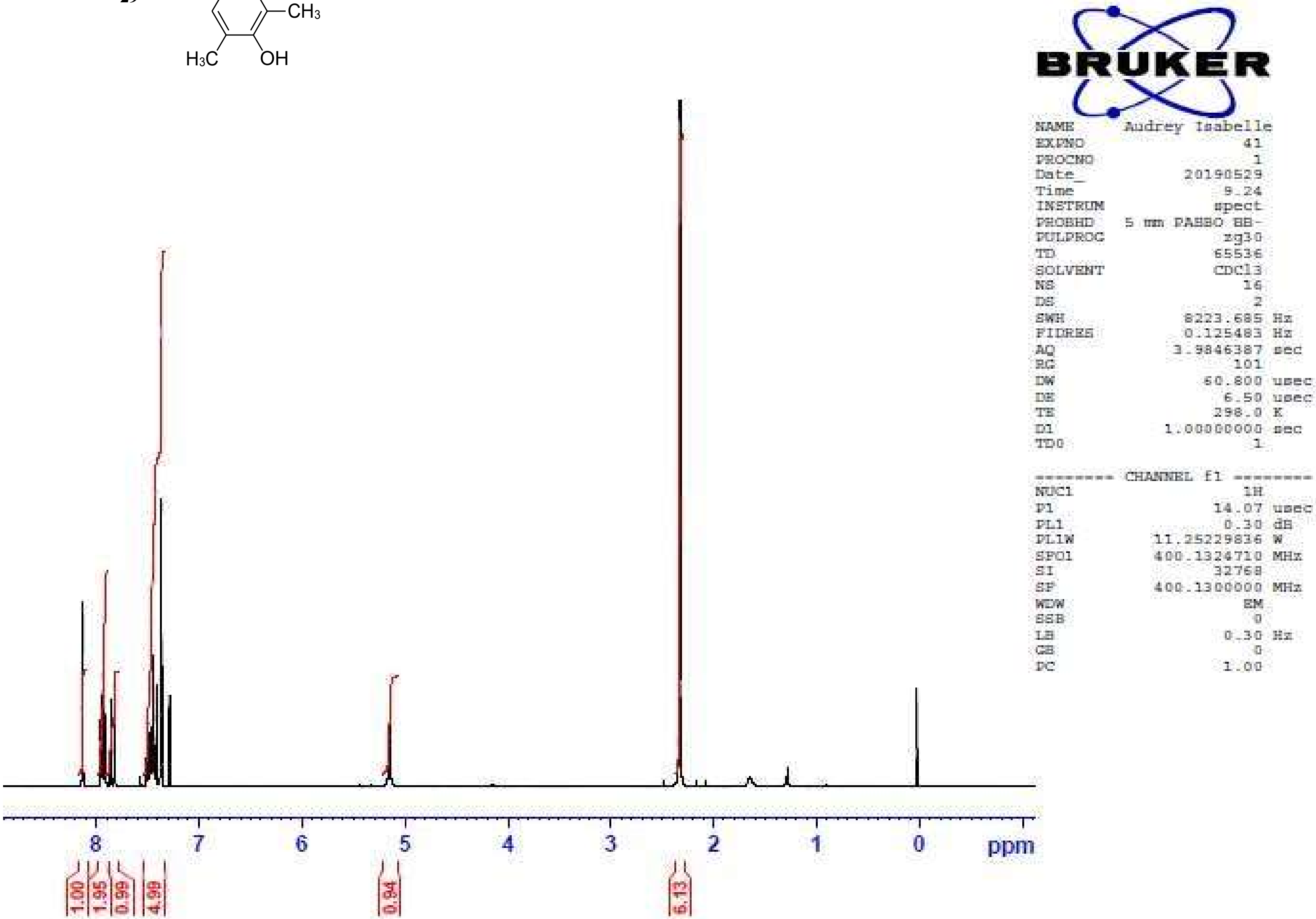
BRUKER
NAME Audrey Isabelle
EXPNO 41
PROCNO 1
Date_ 20190529
Time 9.24
INSTRUM spect
PROBHD 5 mm PABBO BB-
PULPROG zg30
TD 65536
SOLVENT CDCl3
NS 16
DS 2
SWH 8223.685 Hz
FIDRES 0.125483 Hz
AQ 3.9846387 sec
RG 101
DW 60.800 usec
DE 6.50 usec
TE 298.0 K
D1 1.00000000 sec
TD0 1
======== CHANNEL f1 ========
NUC1 1H
P1 14.07 usec
PL1 0.30 dB
PL1W 11.25229836 W
SFO1 400.1324710 MHz
SI 32768
SF 400.1300000 MHz
WDW EM
SSB 0
LB 0.30 Hz
GB 0
PC 1.00
8 7 6 5 4 3 2 1 0 ppm
1.00 1.95 0.99 4.99 0.94 6.13

^{13}C NMR

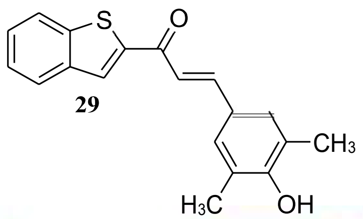

29

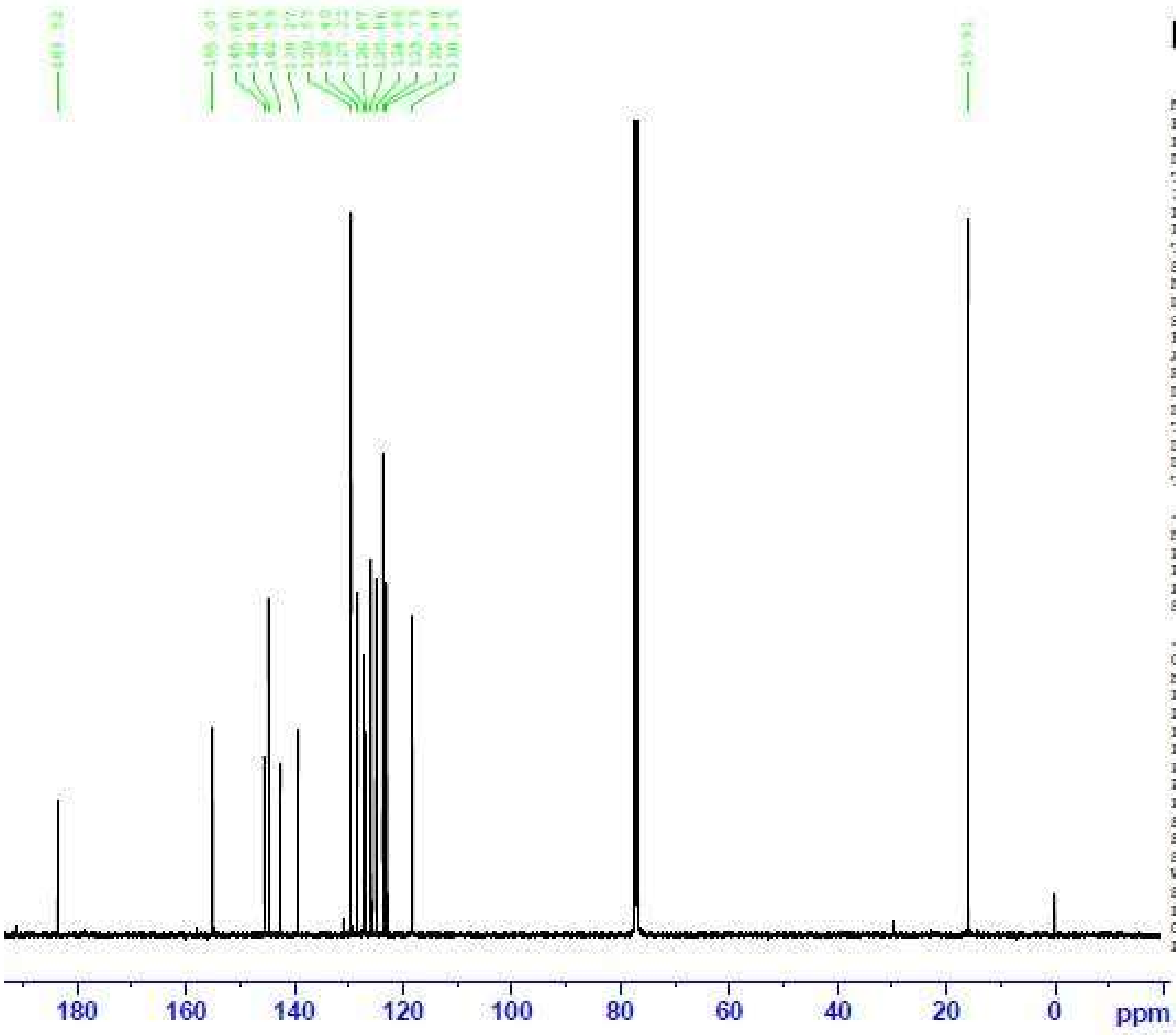

BRUKER

NAME	Audrey Isabelle
EXPNO	46
PROCNO	1
Date_	20190529
Time	23.08
INSTRUM	spect
PROBHD	5 mm PABBO BB-
PULPROG	zgpg30
TD	65536
SOLVENT	CDCl3
NS	10000
DS	4
SWH	24038.461 Hz
FIDRES	0.366798 Hz
AQ	1.3631988 sec
RG	203
DW	20.800 usec
DE	6.50 usec
TE	298.0 K
D1	2.00000000 sec
D11	0.03000000 sec
TD0	1
======== CHANNEL f1 ========	
NUC1	13C
P1	9.90 usec
PL1	-1.90 dB
PL1W	56.02249908 W
SFO1	100.6228298 MHz
======== CHANNEL f2 ========	
CPDPRG2	waltz65
NUC2	1H
PCPD2	80.00 usec
PL2	0.30 dB
PL12	15.40 dB
PL13	18.40 dB
PL2W	11.25229836 W
PL12W	0.34772930 W
PL13W	0.17427748 W
SFO2	400.1316005 MHz
SI	32768
SF	100.6127690 MHz
WDW	EM
SSB	0
LB	1.00 Hz
GB	0
PC	1.40

HRMS

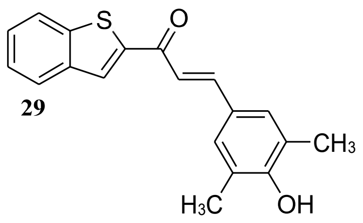
S
O
CH3
H3C
OH

29

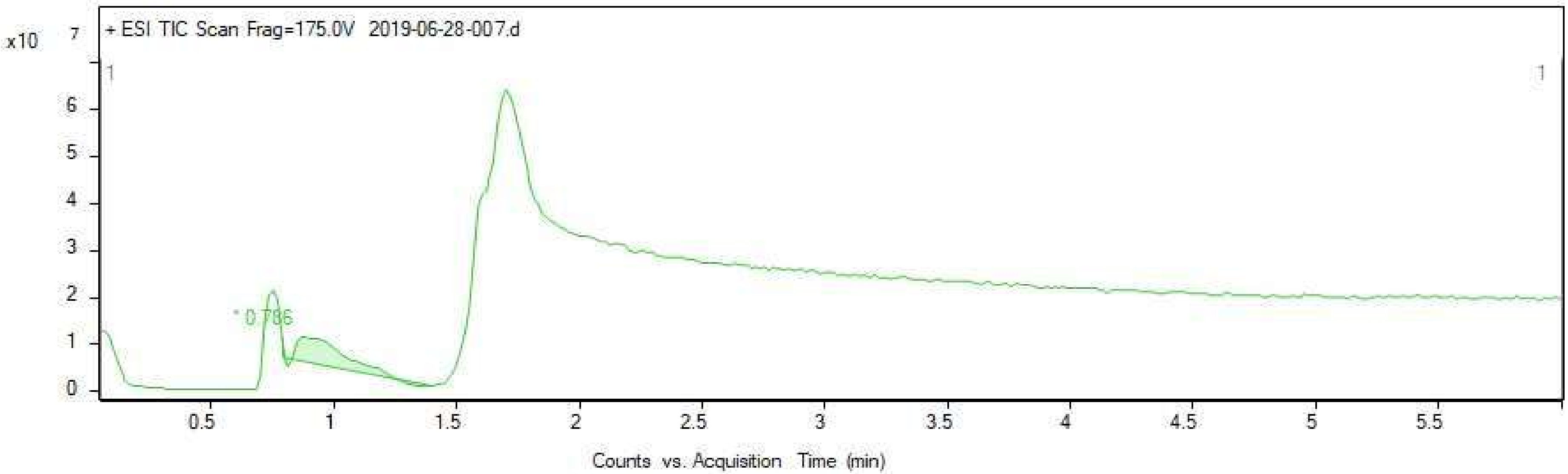
x10 7
+ ESI TIC Scan Frag=175.0V 2019-06-28-007.d
* 0.786
Counts vs. Acquisition Time (min)

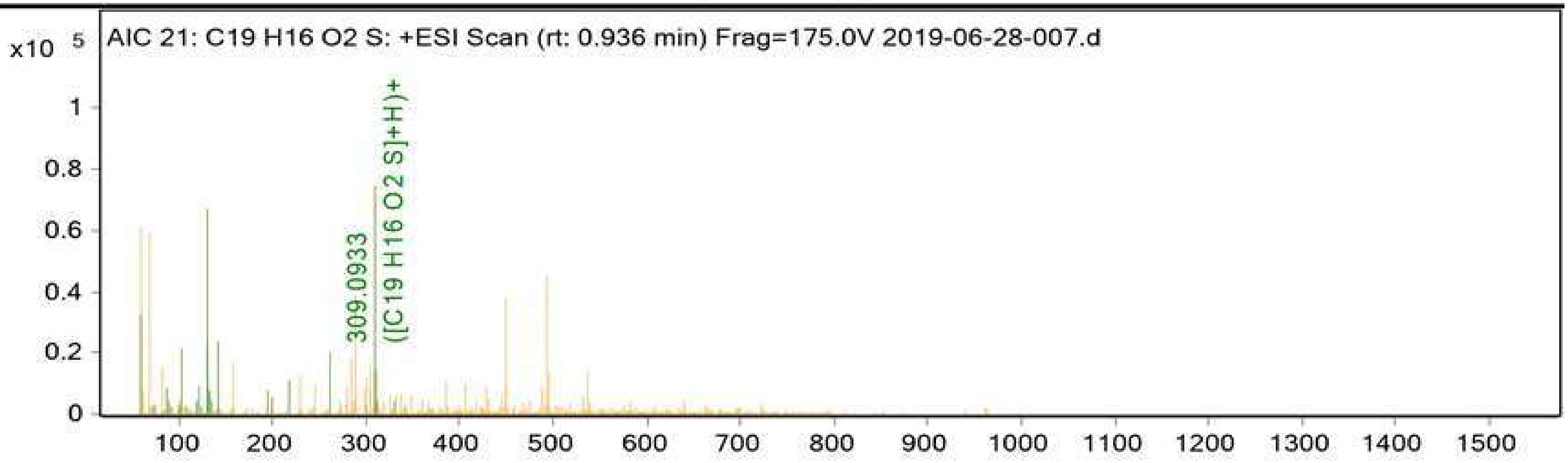
x10 5
AIC 21: C19 H16 O2 S: +ESI Scan (rt: 0.936 min) Frag=175.0V 2019-06-28-007.d
309.0933
([C19 H16 O2 S]+H)+

^{1}H NMR

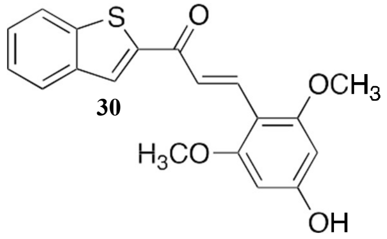

30

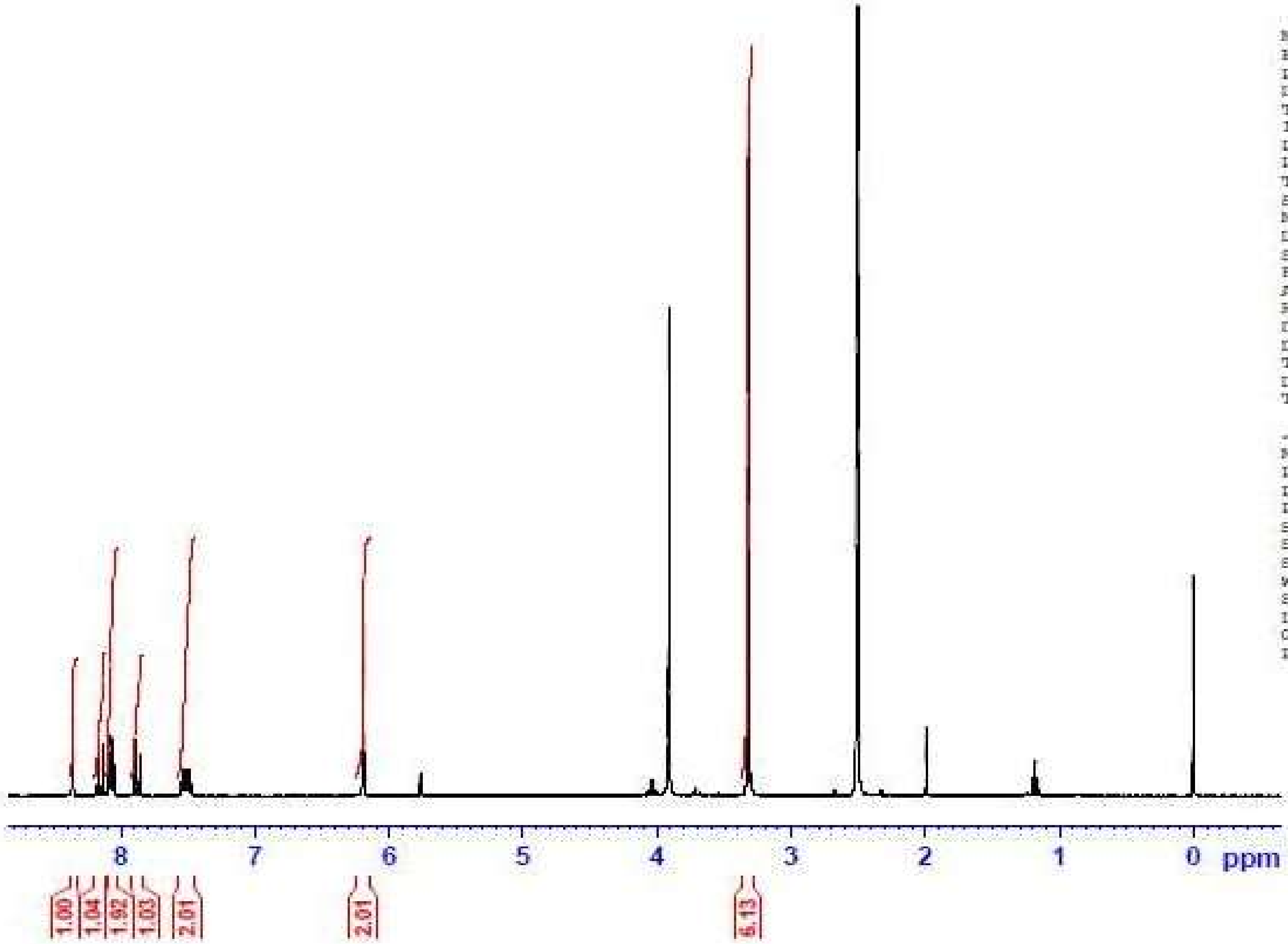

BRUKER

NAME	Audrey Isabelle
EXPNO	34
PROCNO	1
Date_	20190522
Time	13.29
INSTRUM	spect
PROBHD	5 mm PABBO BB-
PULPROG	zg30
TD	65536
SOLVENT	DMSO
NS	16
DS	2
SWH	8223.685 Hz
FIDRES	0.125483 Hz
AQ	3.9846387 sec
RG	203
DW	60.800 usec
DE	6.50 usec
TE	298.0 K
D1	1.00000000 sec
TD0	1
======== CHANNEL f1 ========	
NUC1	1H
P1	14.07 usec
PL1	0.30 dB
PL1W	11.25229836 W
SFO1	400.1324710 MHz
SI	32768
SF	400.1300000 MHz
WDW	EM
SSB	0
LB	0.30 Hz
GB	0
PC	1.00

^{13}C NMR

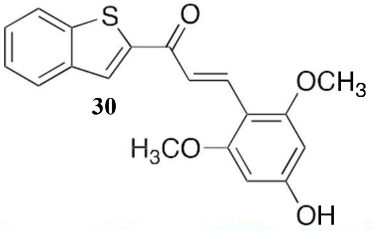

30

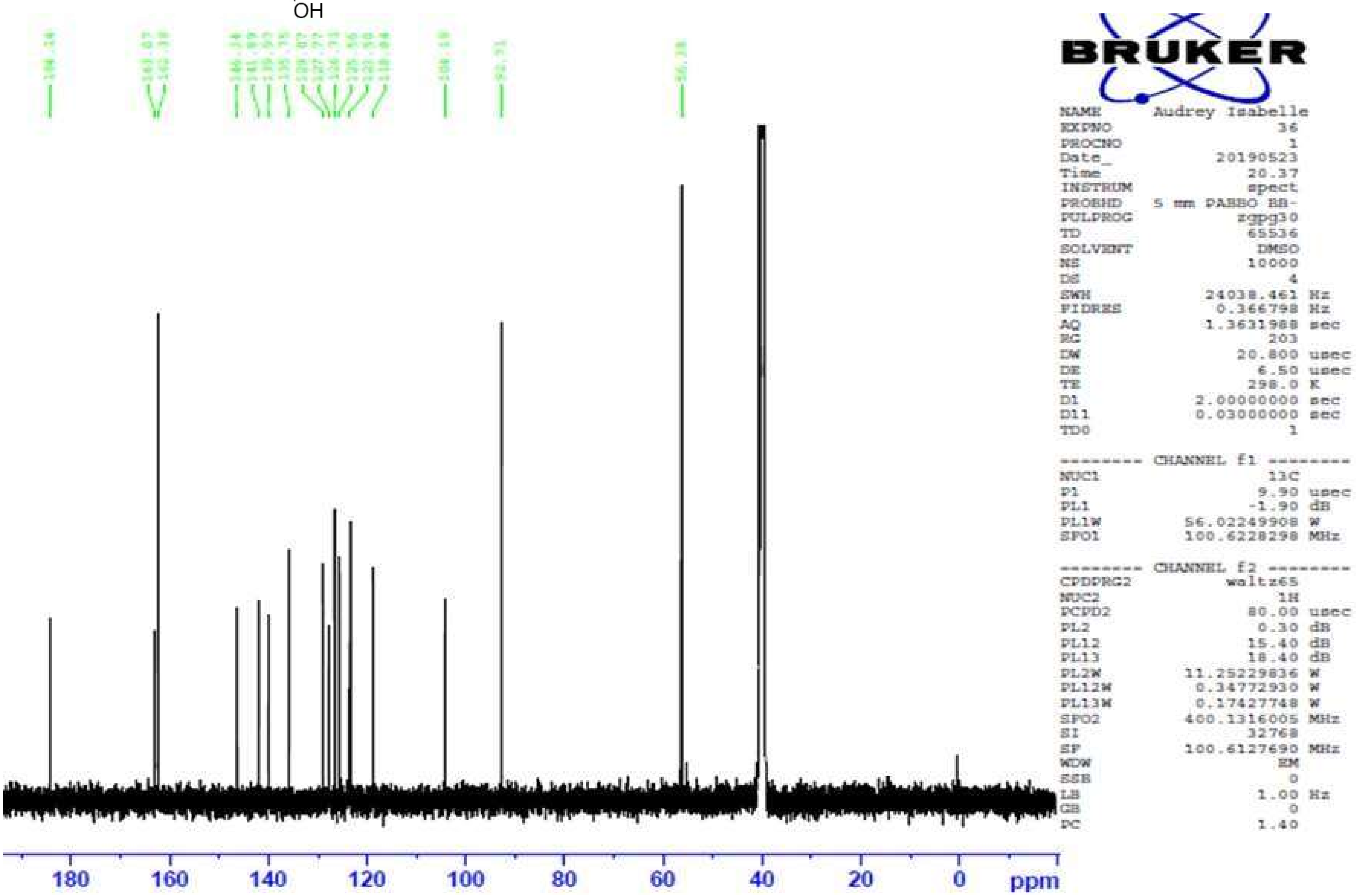
BRUKER
NAME Audrey Isabelle
EXPNO 36
PROCNO 1
Date_ 20190523
Time 20.37
INSTRUM spect
PROBHD 5 mm PABBO BB-
PULPROG zgpg30
TD 65536
SOLVENT DMSO
NS 10000
DS 4
SWH 24038.461 Hz
FIDRES 0.366798 Hz
AQ 1.3631988 sec
RG 203
DW 20.800 usec
DE 6.50 usec
TE 298.0 K
D1 2.00000000 sec
D11 0.03000000 sec
TD0 1
======== CHANNEL f1 ========
NUC1 13C
P1 9.90 usec
PL1 -1.90 dB
PL1W 56.02249908 W
SFO1 100.6228298 MHz
======== CHANNEL f2 ========
CPDPRG2 waltz65
NUC2 1H
PCPD2 80.00 usec
PL2 0.30 dB
PL12 15.40 dB
PL13 18.40 dB
PL2W 11.25229836 W
PL12W 0.34772930 W
PL13W 0.17427748 W
SFO2 400.1316005 MHz
SI 32768
SF 100.6127690 MHz
WDW EM
SSB 0
LB 1.00 Hz
GB 0
PC 1.40
180
160
140
120
100
80
60
40
20
0
ppm

HRMS

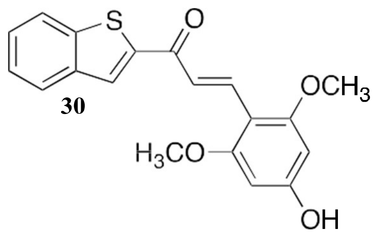
S
O
OCH3
H3CO
OH

30

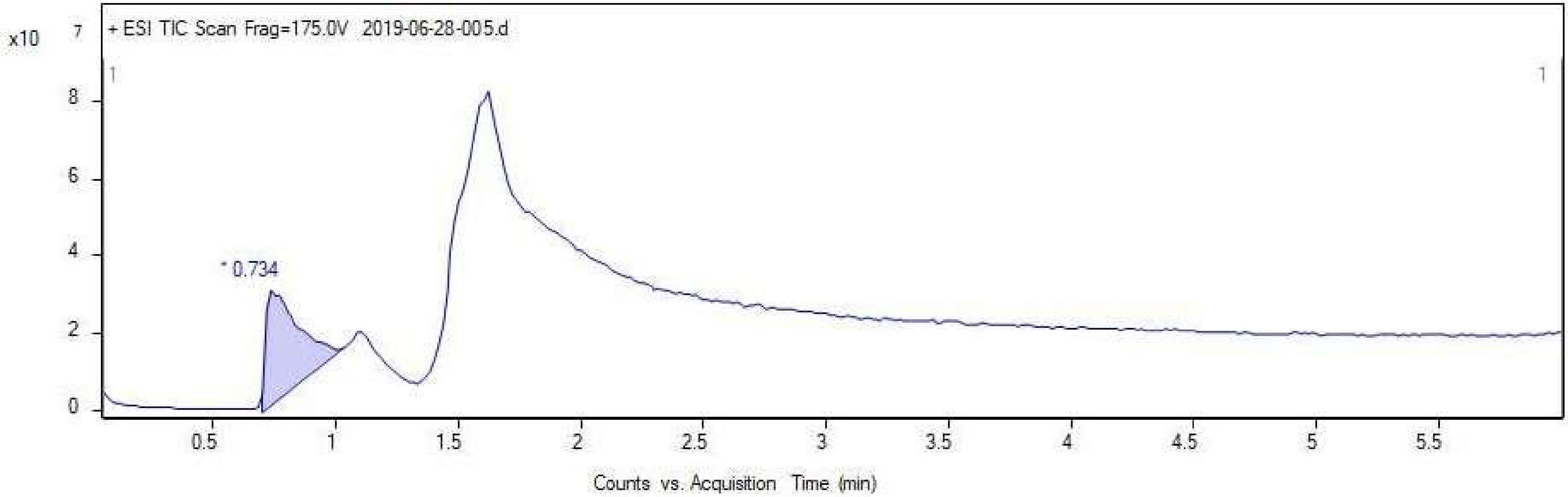
x10 7
+ ESI TIC Scan Frag=175.0V 2019-06-28-005.d
* 0.734
0
2
4
6
8
0.5
1
1.5
2
2.5
3
3.5
4
4.5
5
5.5
Counts vs. Acquisition Time (min)

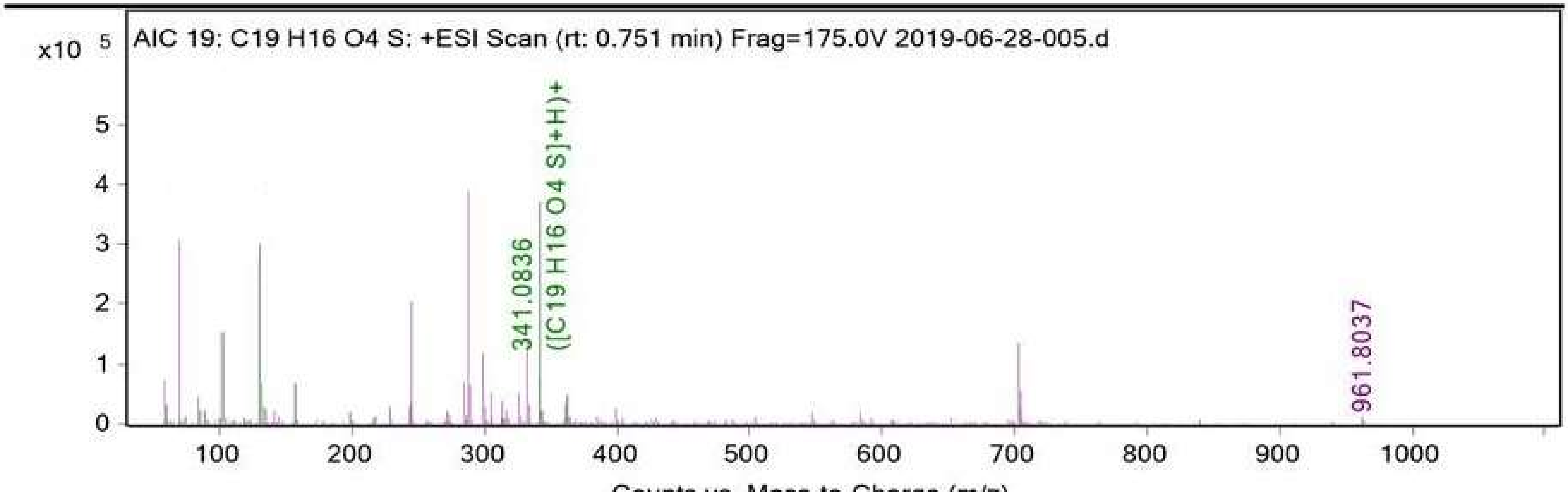
x10 5
AIC 19: C19 H16 O4 S: +ESI Scan (rt: 0.751 min) Frag=175.0V 2019-06-28-005.d
341.0836
([C19 H16 O4 S]+H)+
961.8037
0
1
2
3
4
5
100
200
300
400
500
600
700
800
900
1000

^{1}H NMR

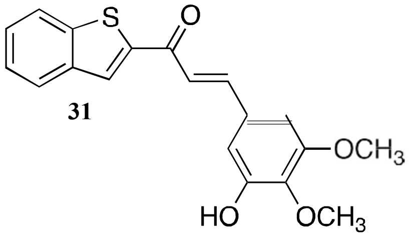

31

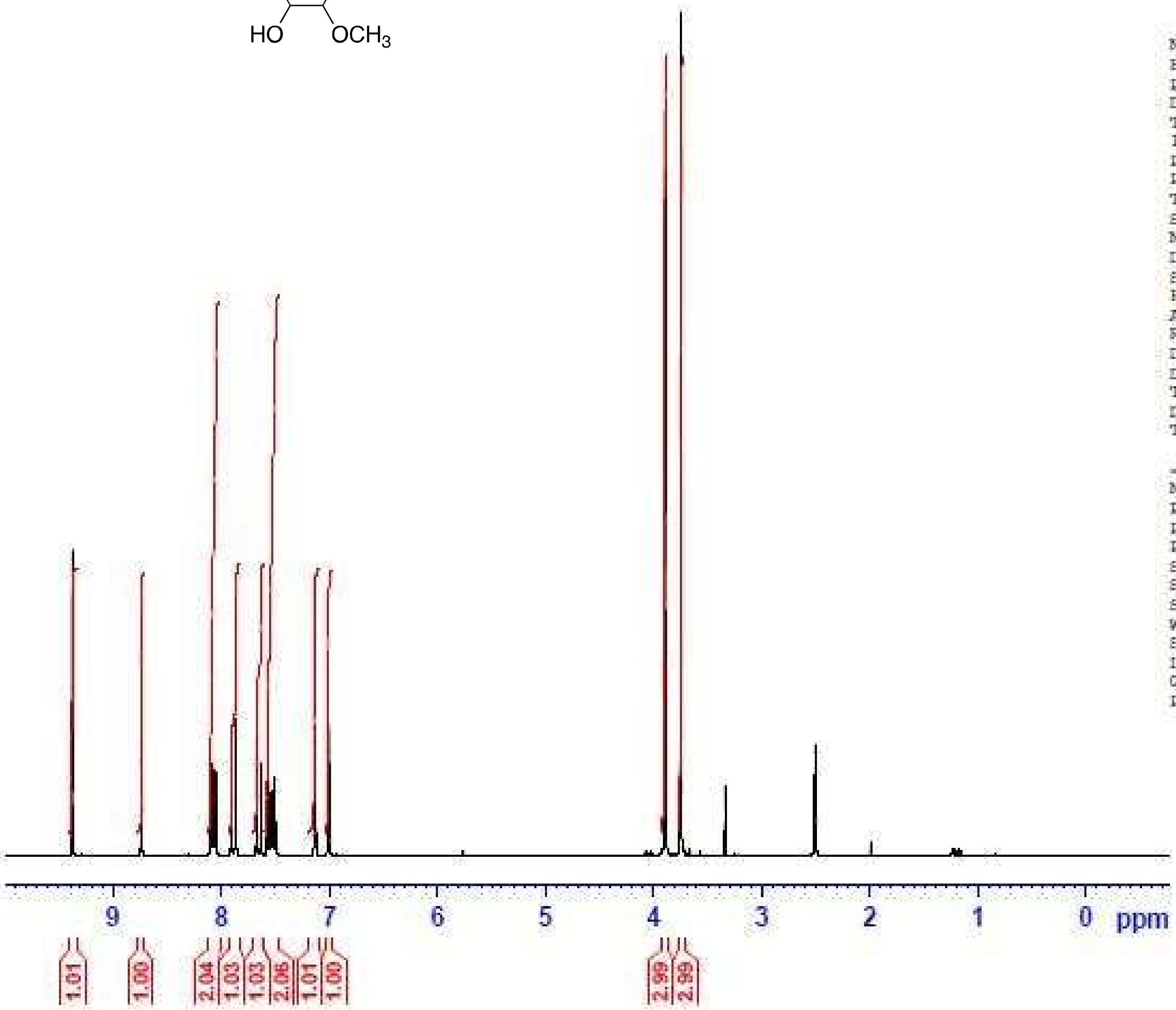


```
NAME       Audrey Isabelle
EXPNO                   89
PROCNO                   1
Date_             20190625
Time                  8.21
INSTRUM              spect
PROBHD     5 mm PABBO BB-
PULPROG               zg30
TD                   65536
SOLVENT               DMSO
NS                      16
DS                       2
SWH               8223.685 Hz
FIDRES            0.125483 Hz
AQ               3.9846387 sec
RG                     101
DW                  60.800 usec
DE                    6.50 usec
TE                   298.0 K
D1              1.00000000 sec
TD0                      1

======== CHANNEL f1 ========
NUC1                    1H
P1                   14.07 usec
PL1                   0.30 dB
PL1W           11.25229836 W
SFO1           400.1324710 MHz
SI                   32768
SF             400.1300000 MHz
WDW                     EM
SSB                      0
LB                    0.30 Hz
GB                       0
PC                    1.00
```

^{13}C NMR

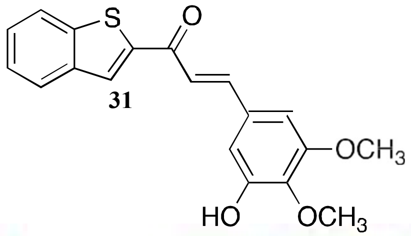

31

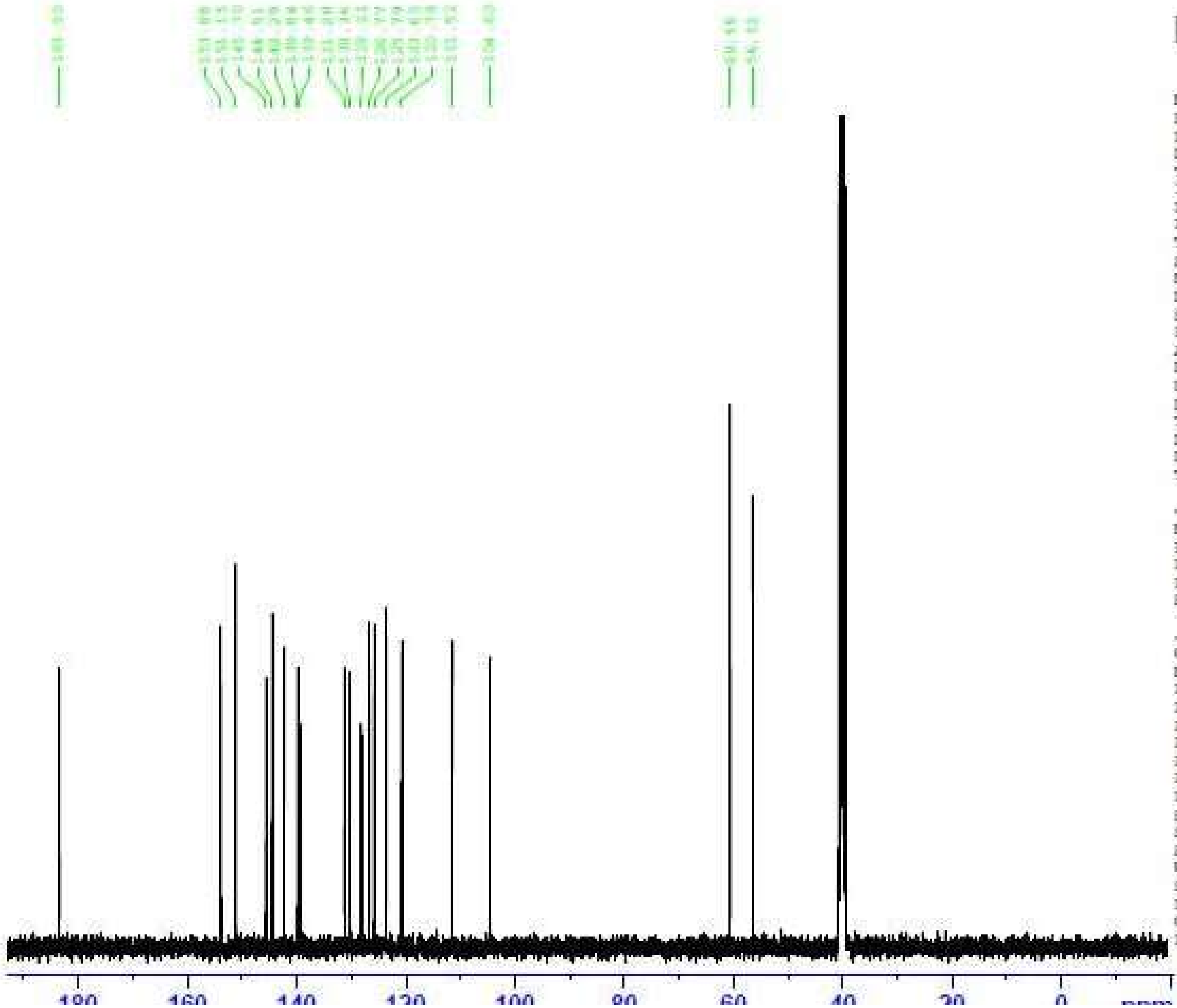

NAME	Audrey Isabelle
EXPNO	91
PROCNO	1
Date_	20190625
Time	10.03
INSTRUM	spect
PROBHD	5 mm PABBO BB-
PULPROG	zgpg30
TD	65536
SOLVENT	DMSO
NS	1574
DS	4
SWH	24038.461 Hz
FIDRES	0.366798 Hz
AQ	1.3631988 sec
RG	203
DW	20.800 usec
DE	6.50 usec
TE	298.0 K
D1	2.00000000 sec
D11	0.03000000 sec
TD0	1
======== CHANNEL f1 ========	
NUC1	13C
P1	9.90 usec
PL1	-1.90 dB
PL1W	56.02249908 W
SFO1	100.6228298 MHz
======== CHANNEL f2 ========	
CPDPRG2	waltz65
NUC2	1H
PCPD2	80.00 usec
PL2	0.30 dB
PL12	15.40 dB
PL13	18.40 dB
PL2W	11.25229836 W
PL12W	0.34772930 W
PL13W	0.17427748 W
SFO2	400.1316005 MHz
SI	32768
SF	100.6127690 MHz
WDW	EM
SSB	0
LB	1.00 Hz
GB	0
PC	1.40

HRMS

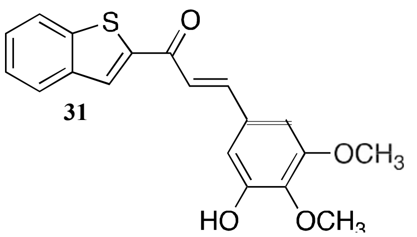

31

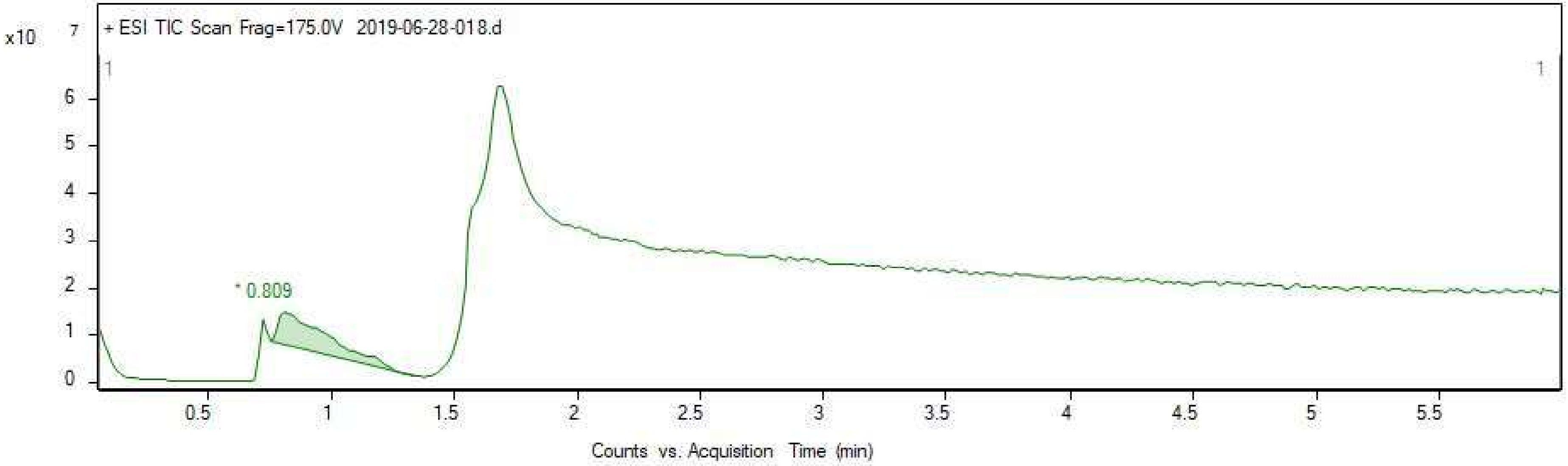
x10 7
+ ESI TIC Scan Frag=175.0V 2019-06-28-018.d
* 0.809
Counts vs. Acquisition Time (min)

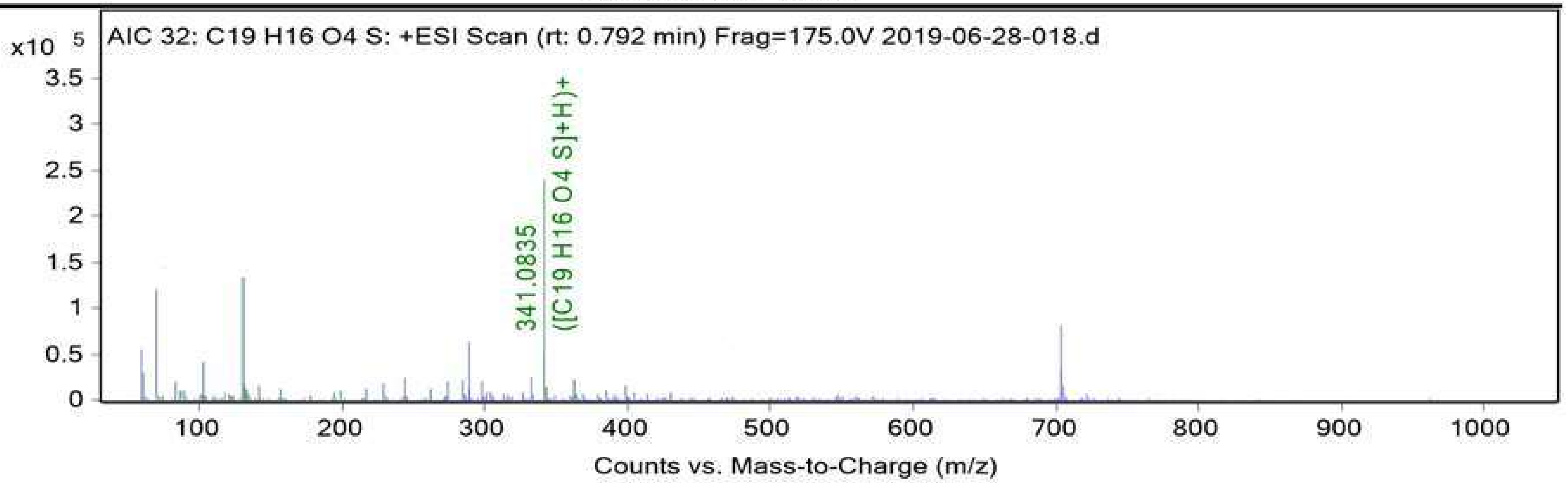
x10 5
AIC 32: C19 H16 O4 S: +ESI Scan (rt: 0.792 min) Frag=175.0V 2019-06-28-018.d
341.0835
([C19 H16 O4 S]+H)+
Counts vs. Mass-to-Charge (m/z)

^{1}H NMR

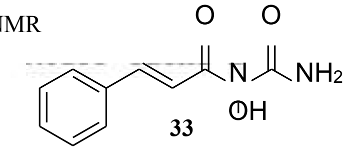
O
O
N
NH2
OH

33

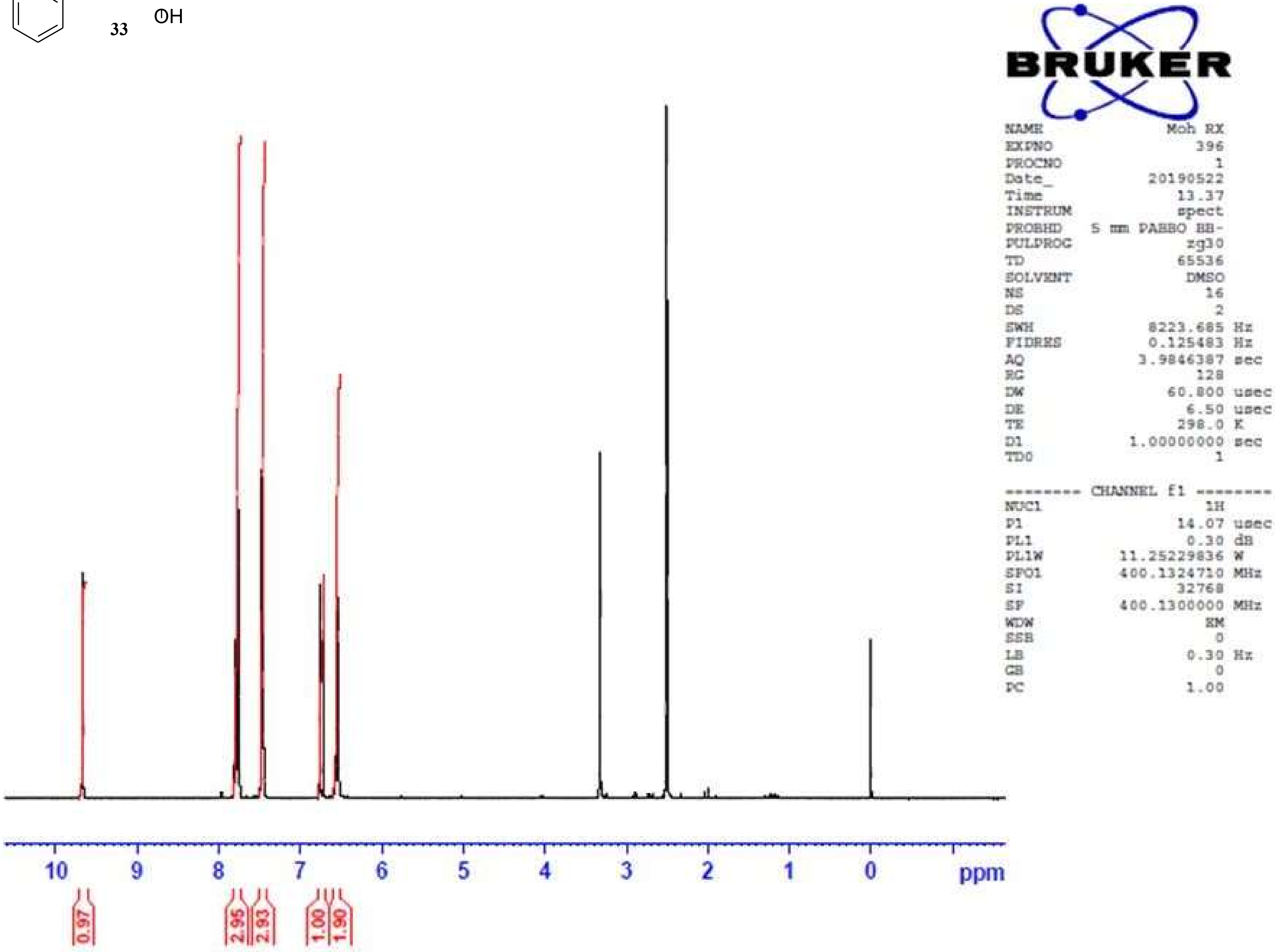
BRUKER
NAME Moh RX
EXPNO 396
PROCNO 1
Date_ 20190522
Time 13.37
INSTRUM spect
PROBHD 5 mm PABBO BB-
PULPROG zg30
TD 65536
SOLVENT DMSO
NS 16
DS 2
SWH 8223.685 Hz
FIDRES 0.125483 Hz
AQ 3.9846387 sec
RG 128
DW 60.800 usec
DE 6.50 usec
TE 298.0 K
D1 1.00000000 sec
TD0 1
======== CHANNEL f1 ========
NUC1 1H
P1 14.07 usec
PL1 0.30 dB
PL1W 11.25229836 W
SFO1 400.1324710 MHz
SI 32768
SF 400.1300000 MHz
WDW EM
SSB 0
LB 0.30 Hz
GB 0
PC 1.00
10
9
8
7
6
5
4
3
2
1
0
ppm
0.97
2.95
2.93
1.00
1.90

^{13}C NMR

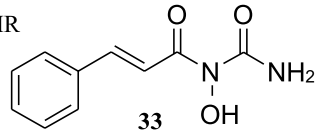

O
O
N
NH2
OH

33

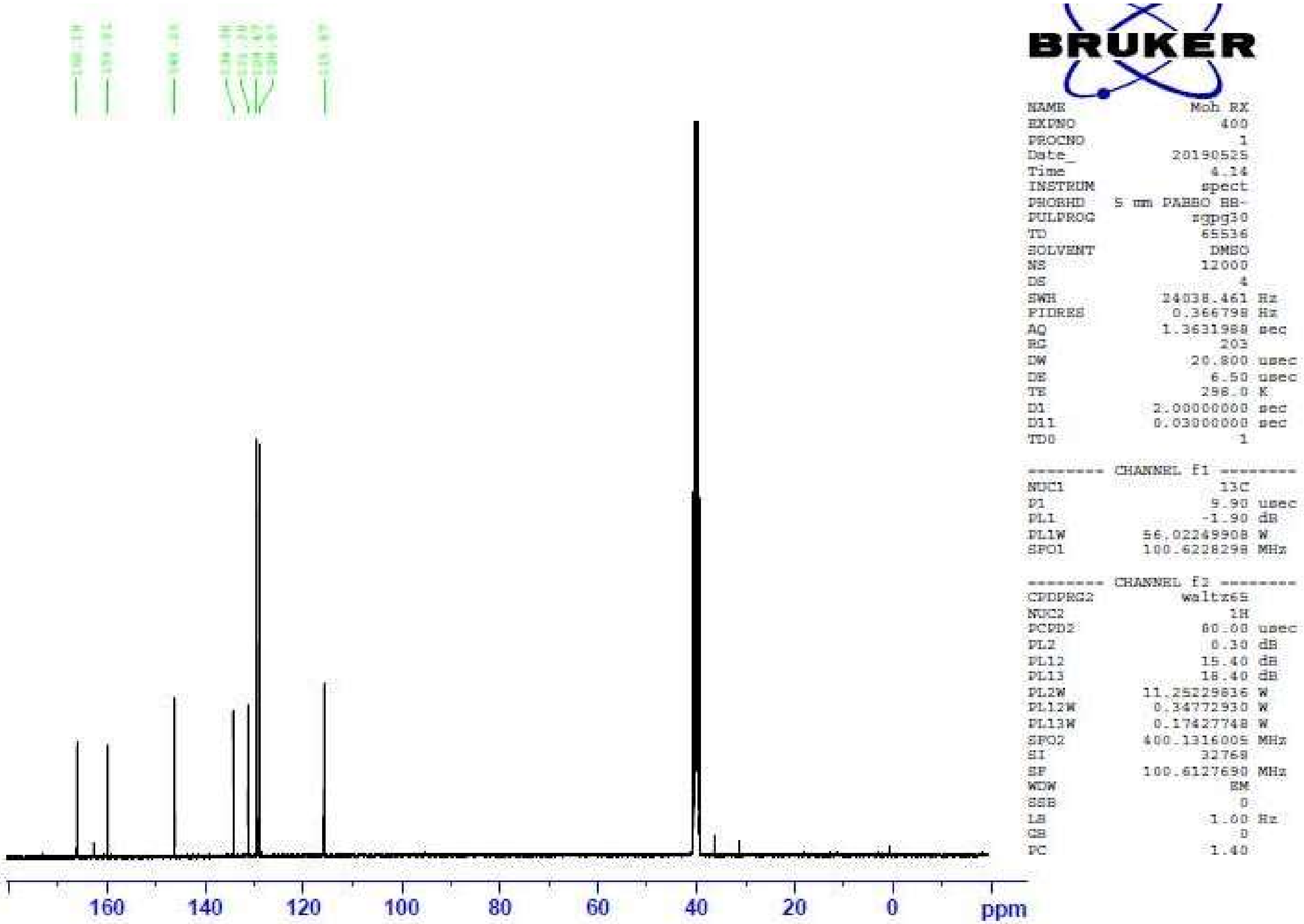

BRUKER
NAME Moh RX
EXPNO 400
PROCNO 1
Date_ 20190525
Time 4.14
INSTRUM spect
PROBHD 5 mm PABBO BB-
PULPROG zgpg30
TD 65536
SOLVENT DMSO
NS 12000
DS 4
SWH 24038.461 Hz
FIDRES 0.366798 Hz
AQ 1.3631988 sec
RG 203
DW 20.800 usec
DE 6.50 usec
TE 298.0 K
D1 2.00000000 sec
D11 0.03000000 sec
TD0 1
======== CHANNEL f1 ========
NUC1 13C
P1 9.90 usec
PL1 -1.90 dB
PL1W 56.02249908 W
SFO1 100.6228298 MHz
======== CHANNEL f2 ========
CPDPRG2 waltz65
NUC2 1H
PCPD2 80.00 usec
PL2 0.30 dB
PL12 15.40 dB
PL13 18.40 dB
PL2W 11.25229836 W
PL12W 0.34772930 W
PL13W 0.17427748 W
SFO2 400.1316005 MHz
SI 32768
SF 100.6127690 MHz
WDW EM
SSB 0
LB 1.00 Hz
GB 0
PC 1.40
160
140
120
100
80
60
40
20
0
ppm

HRMS

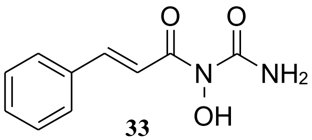
O
O
N
NH2
OH

33

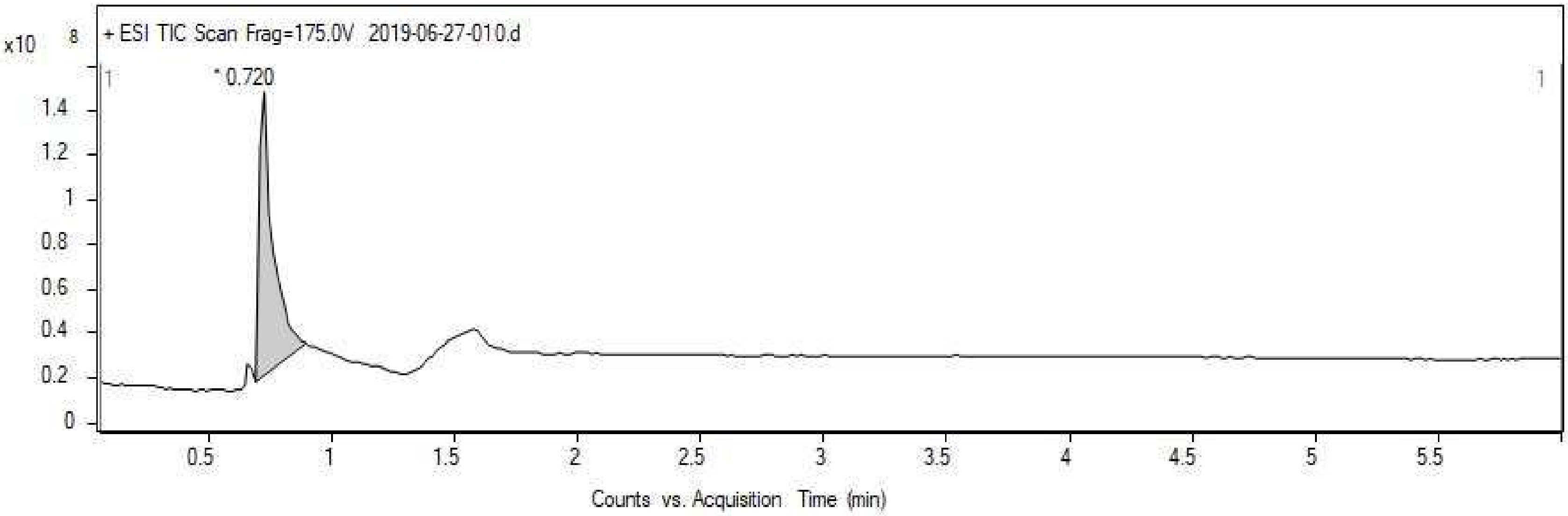
x10 8
+ ESI TIC Scan Frag=175.0V 2019-06-27-010.d
* 0.720
1.4
1.2
1
0.8
0.6
0.4
0.2
0
0.5
1
1.5
2
2.5
3
3.5
4
4.5
5
5.5
Counts vs. Acquisition Time (min)

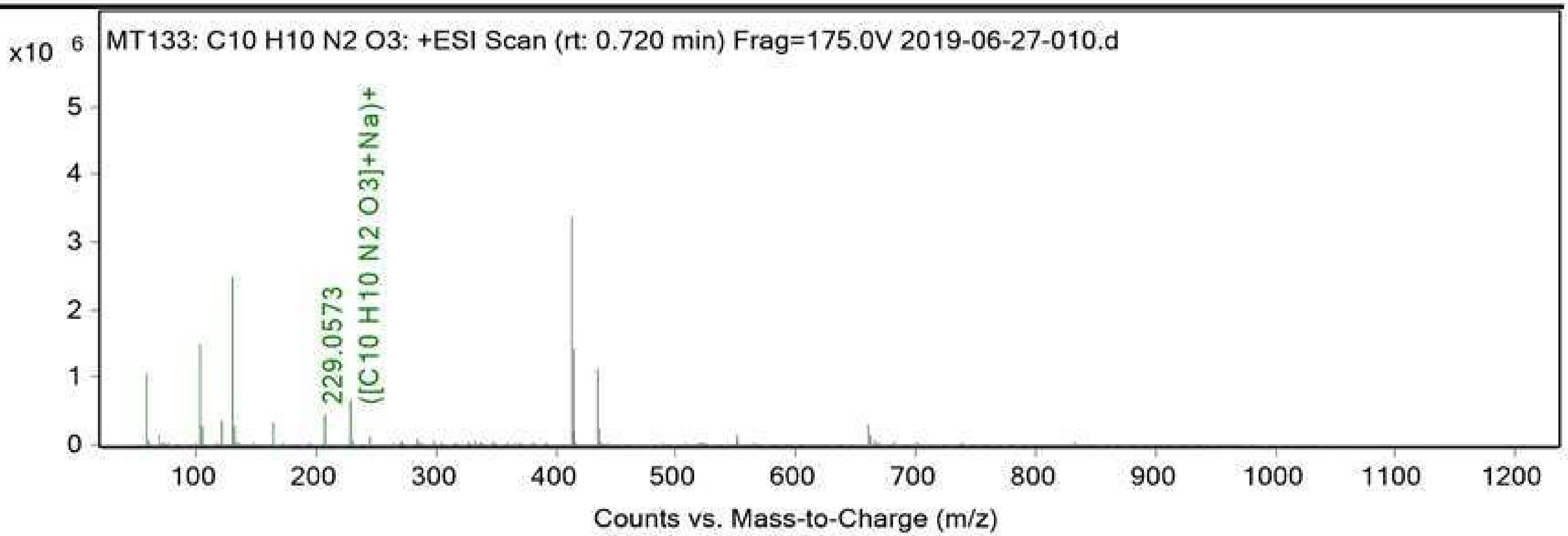
x10 6
MT133: C10 H10 N2 O3: +ESI Scan (rt: 0.720 min) Frag=175.0V 2019-06-27-010.d
5
4
3
2
1
0
229.0573
([C10 H10 N2 O3]+Na)+
100
200
300
400
500
600
700
800
900
1000
1100
1200
Counts vs. Mass-to-Charge (m/z)

^1H NMR

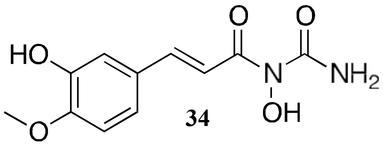
HO
O
O
O
N
NH2
OH

34

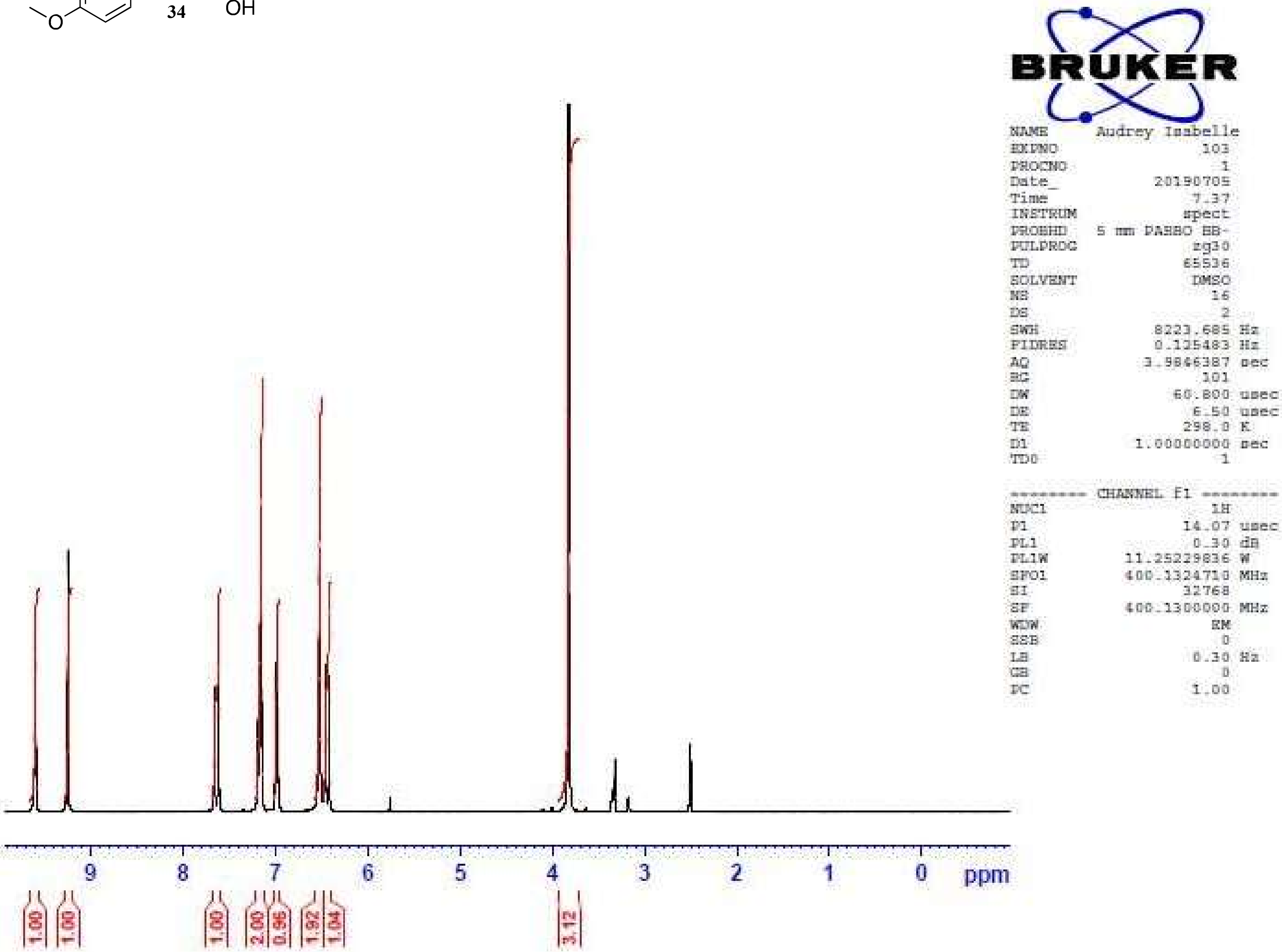
BRUKER
NAME Audrey Issbelle
EXPNO 103
PROCNO 1
Date_ 20190705
Time 7.37
INSTRUM spect
PROBHD 5 mm PABBO BB-
PULPROG zg30
TD 65536
SOLVENT DMSO
NS 16
DS 2
SWH 8223.685 Hz
FIDRES 0.125483 Hz
AQ 3.9846387 sec
RG 101
DW 60.800 usec
DE 6.50 usec
TE 298.0 K
D1 1.00000000 sec
TD0 1
======== CHANNEL f1 ========
NUC1 1H
P1 14.07 usec
PL1 0.30 dB
PL1W 11.25229836 W
SFO1 400.1324710 MHz
SI 32768
SF 400.1300000 MHz
WDW EM
SSB 0
LB 0.30 Hz
GB 0
PC 1.00
9
8
7
6
5
4
3
2
1
0
ppm
1.00
1.00
1.00
2.00
0.96
1.92
1.04
3.12

^{13}C NMR

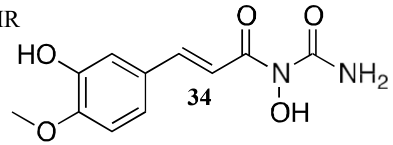

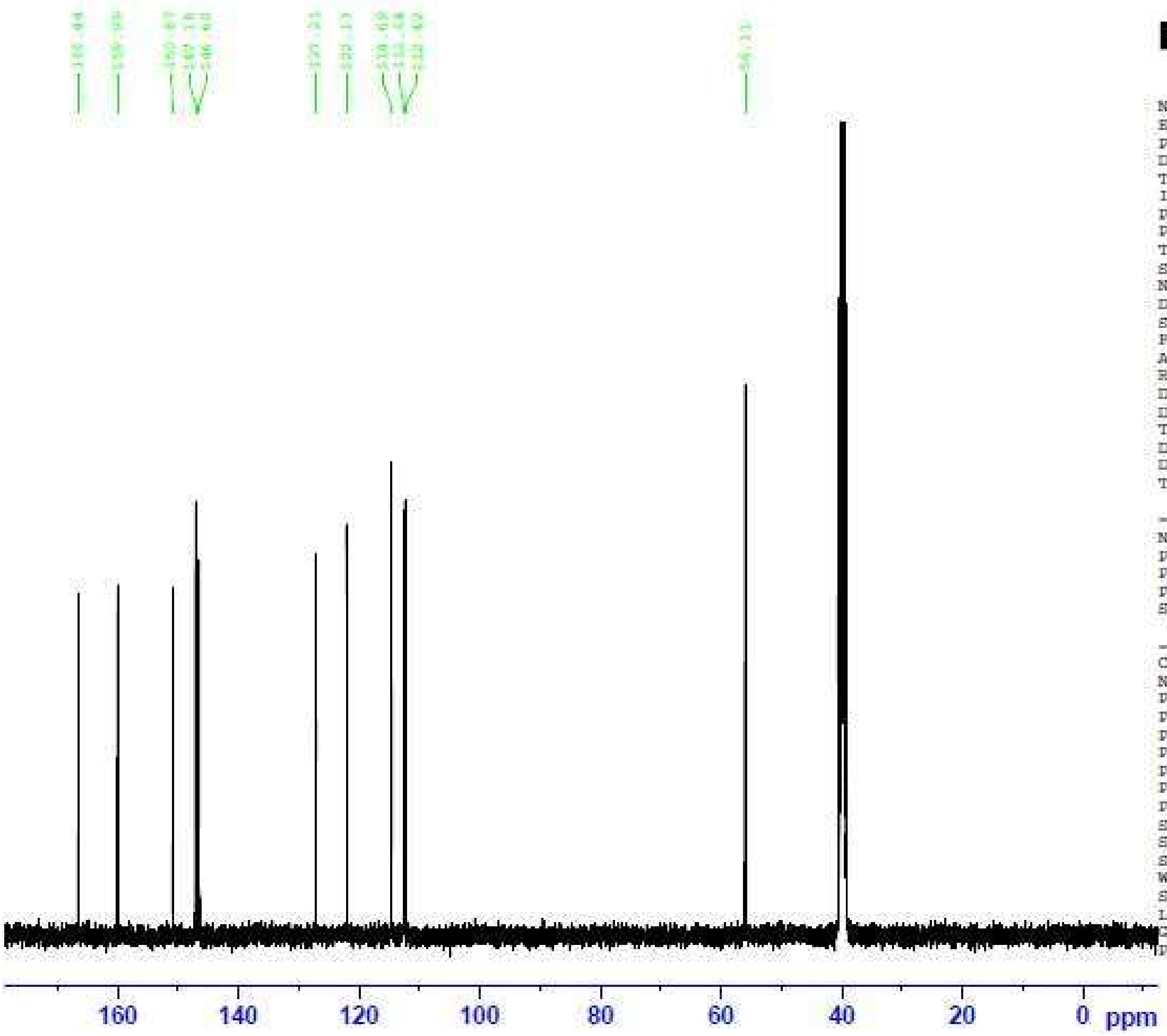

NAME	Audrey Isabelle
EXPNO	105
PROCNO	1
Date_	20190705
Time	9.57
INSTRUM	spect
PROBHD	5 mm PABBO BB-
PULPROG	zgpg30
TD	65536
SOLVENT	DMSO
NS	2136
DS	4
SWH	24038.461 Hz
FIDRES	0.366798 Hz
AQ	1.3631988 sec
RG	203
DW	20.800 usec
DE	6.50 usec
TE	298.0 K
D1	2.00000000 sec
D11	0.03000000 sec
TD0	1
======== CHANNEL f1 ========	
NUC1	13C
P1	9.90 usec
PL1	-1.90 dB
PL1W	56.02249908 W
SFO1	100.6228298 MHz
======== CHANNEL f2 ========	
CPDPRG2	waltz65
NUC2	1H
PCPD2	80.00 usec
PL2	0.30 dB
PL12	15.40 dB
PL13	18.40 dB
PL2W	11.25229836 W
PL12W	0.34772930 W
PL13W	0.17427748 W
SFO2	400.1316005 MHz
SI	32768
SF	100.6127690 MHz
WDW	EM
SSB	0
LB	1.00 Hz
GB	0
PC	1.40

HRMS

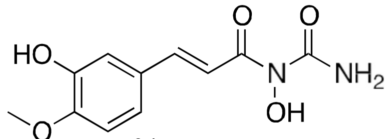
HO
O
O
N
NH2
OH
O

34

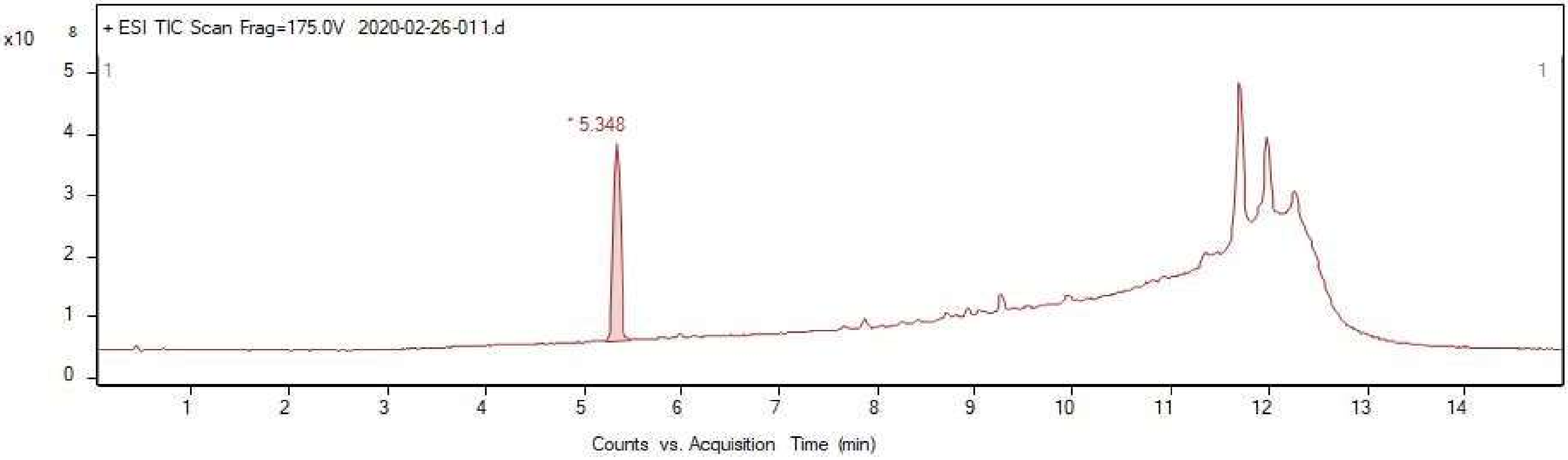
x10 8
+ ESI TIC Scan Frag=175.0V 2020-02-26-011.d
* 5.348
Counts vs. Acquisition Time (min)

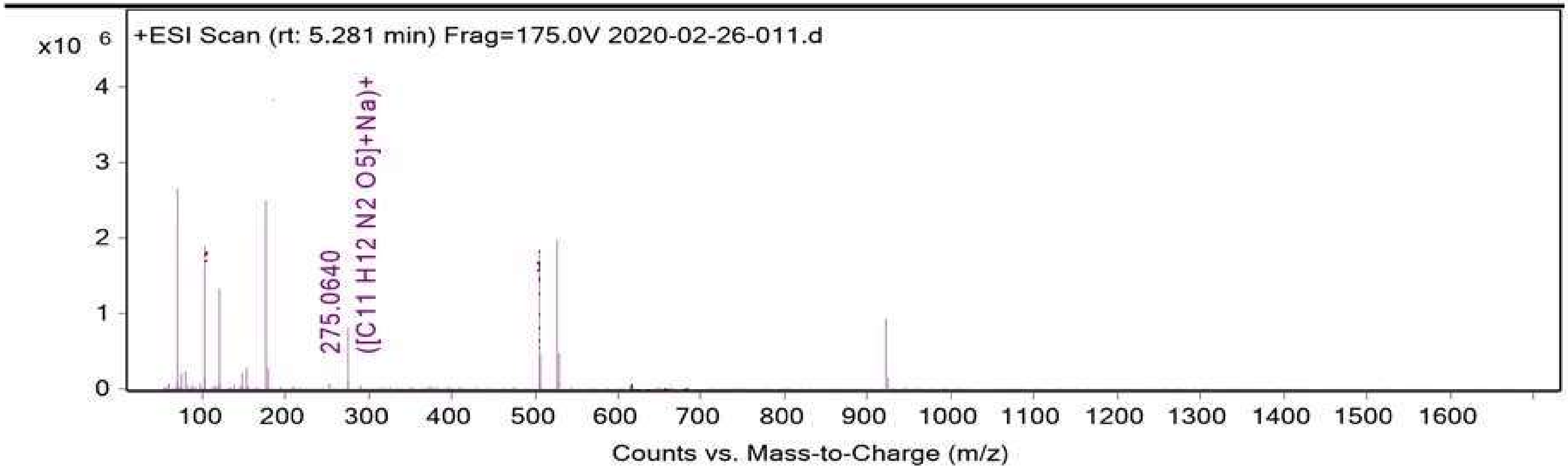
x10 6
+ESI Scan (rt: 5.281 min) Frag=175.0V 2020-02-26-011.d
275.0640
([C11 H12 N2 O5]+Na)+
Counts vs. Mass-to-Charge (m/z)

^{1}H NMR

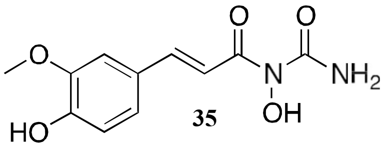
O
O
O
N
NH2
OH
HO
35

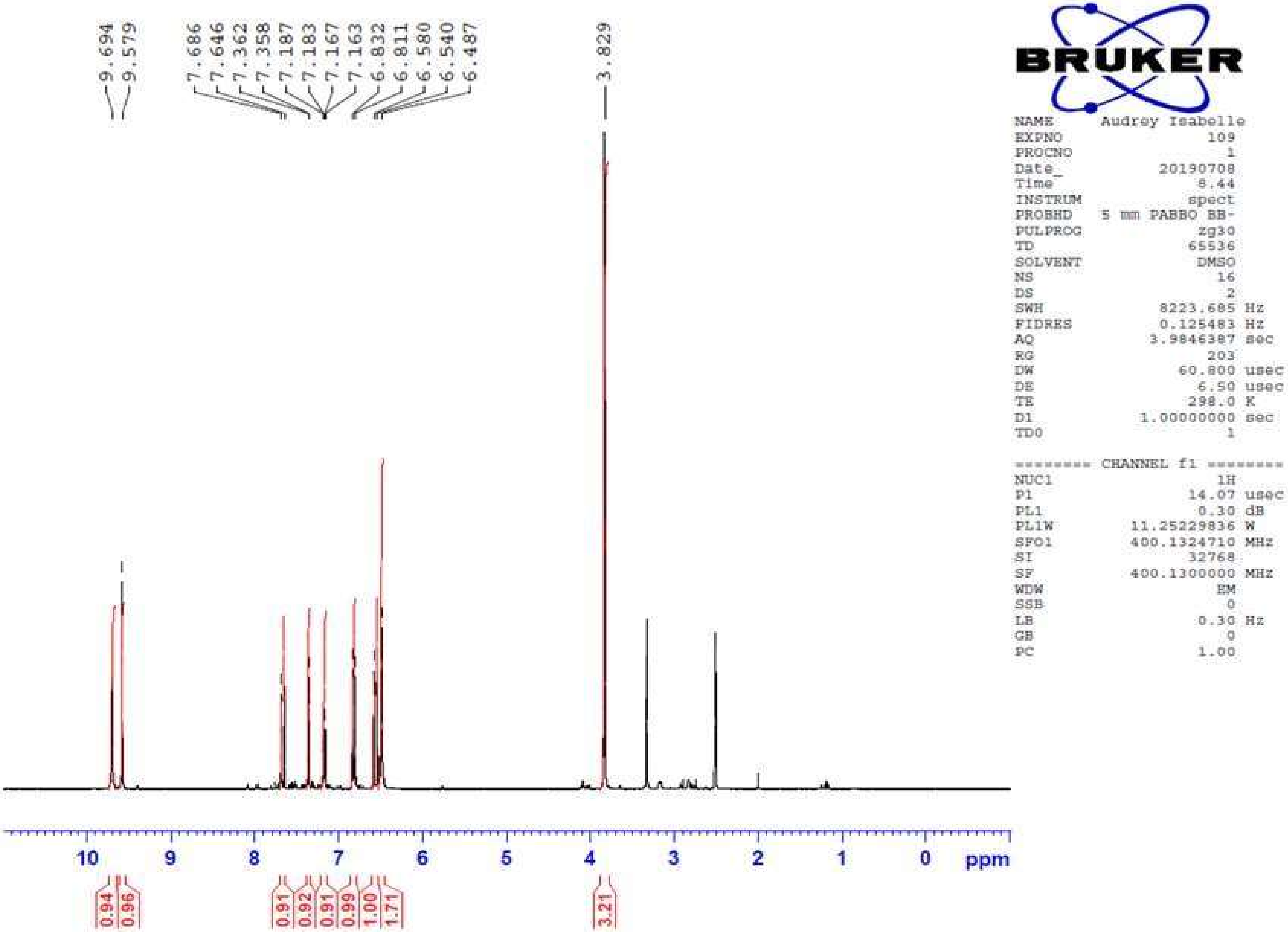
9.694
9.579
7.686
7.646
7.362
7.358
7.187
7.183
7.167
7.163
6.832
6.811
6.580
6.540
6.487
3.829
BRUKER
NAME Audrey Isabelle
EXPNO 109
PROCNO 1
Date_ 20190708
Time 8.44
INSTRUM spect
PROBHD 5 mm PABBO BB-
PULPROG zg30
TD 65536
SOLVENT DMSO
NS 16
DS 2
SWH 8223.685 Hz
FIDRES 0.125483 Hz
AQ 3.9846387 sec
RG 203
DW 60.800 usec
DE 6.50 usec
TE 298.0 K
D1 1.00000000 sec
TD0 1
======== CHANNEL f1 ========
NUC1 1H
P1 14.07 usec
PL1 0.30 dB
PL1W 11.25229836 W
SFO1 400.1324710 MHz
SI 32768
SF 400.1300000 MHz
WDW EM
SSB 0
LB 0.30 Hz
GB 0
PC 1.00
10
9
8
7
6
5
4
3
2
1
0
ppm
0.94
0.96
0.91
0.92
0.91
0.99
1.00
1.71
3.21

^{13}C NMR

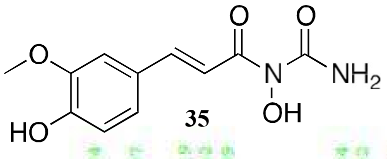
O
O
O
N
NH2
HO
OH
35

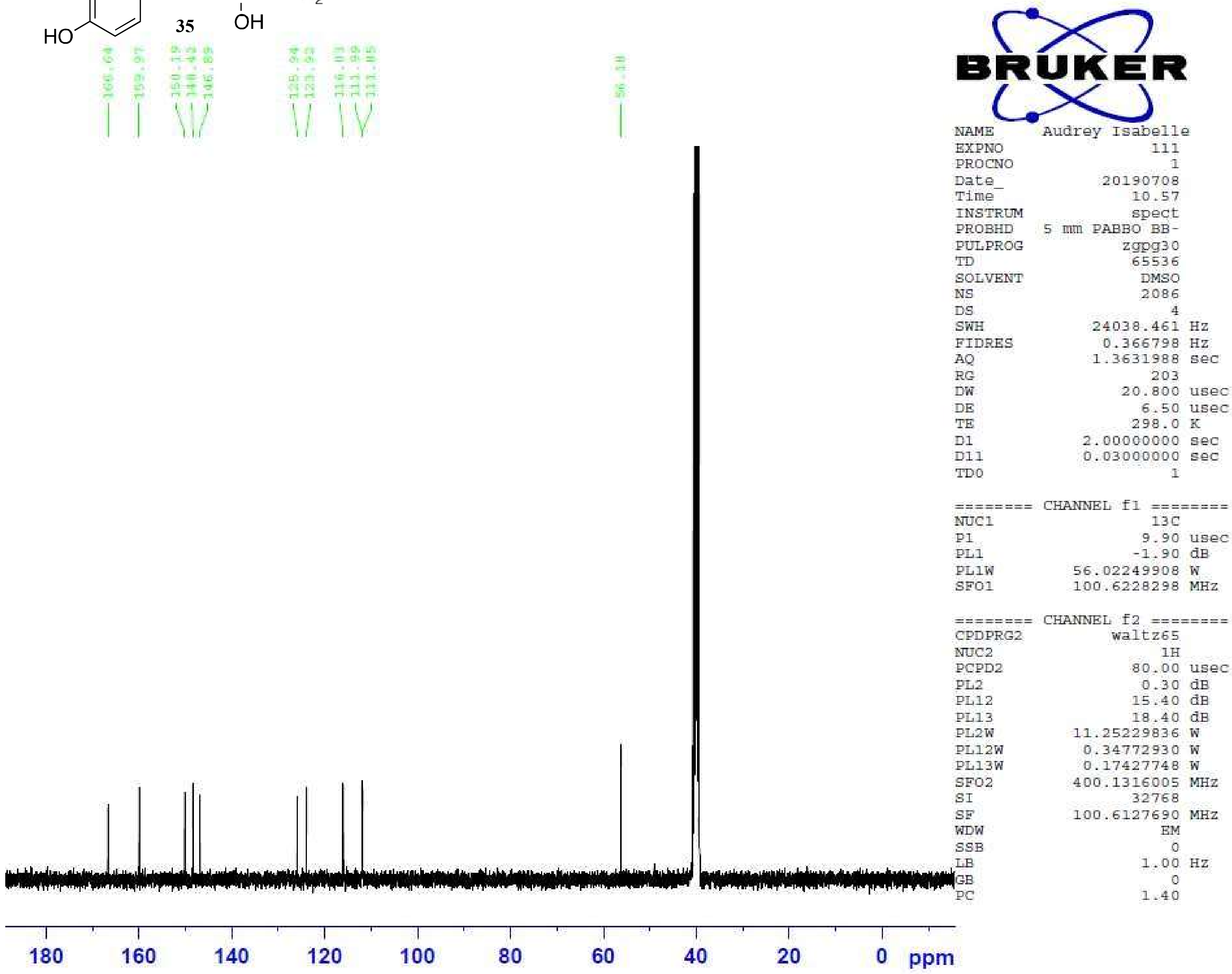
166.64
159.97
150.19
148.43
146.89
125.94
123.92
116.03
111.99
111.85
56.18
180
160
140
120
100
80
60
40
20
0
ppm
BRUKER
NAME Audrey Isabelle
EXPNO 111
PROCNO 1
Date_ 20190708
Time 10.57
INSTRUM spect
PROBHD 5 mm PABBO BB-
PULPROG zgpg30
TD 65536
SOLVENT DMSO
NS 2086
DS 4
SWH 24038.461 Hz
FIDRES 0.366798 Hz
AQ 1.3631988 sec
RG 203
DW 20.800 usec
DE 6.50 usec
TE 298.0 K
D1 2.00000000 sec
D11 0.03000000 sec
TD0 1
======== CHANNEL f1 ========
NUC1 13C
P1 9.90 usec
PL1 -1.90 dB
PL1W 56.02249908 W
SFO1 100.6228298 MHz
======== CHANNEL f2 ========
CPDPRG2 waltz65
NUC2 1H
PCPD2 80.00 usec
PL2 0.30 dB
PL12 15.40 dB
PL13 18.40 dB
PL2W 11.25229836 W
PL12W 0.34772930 W
PL13W 0.17427748 W
SFO2 400.1316005 MHz
SI 32768
SF 100.6127690 MHz
WDW EM
SSB 0
LB 1.00 Hz
GB 0
PC 1.40

HRMS

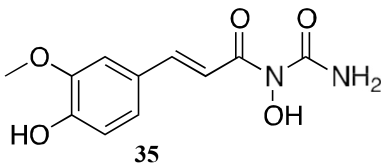

35

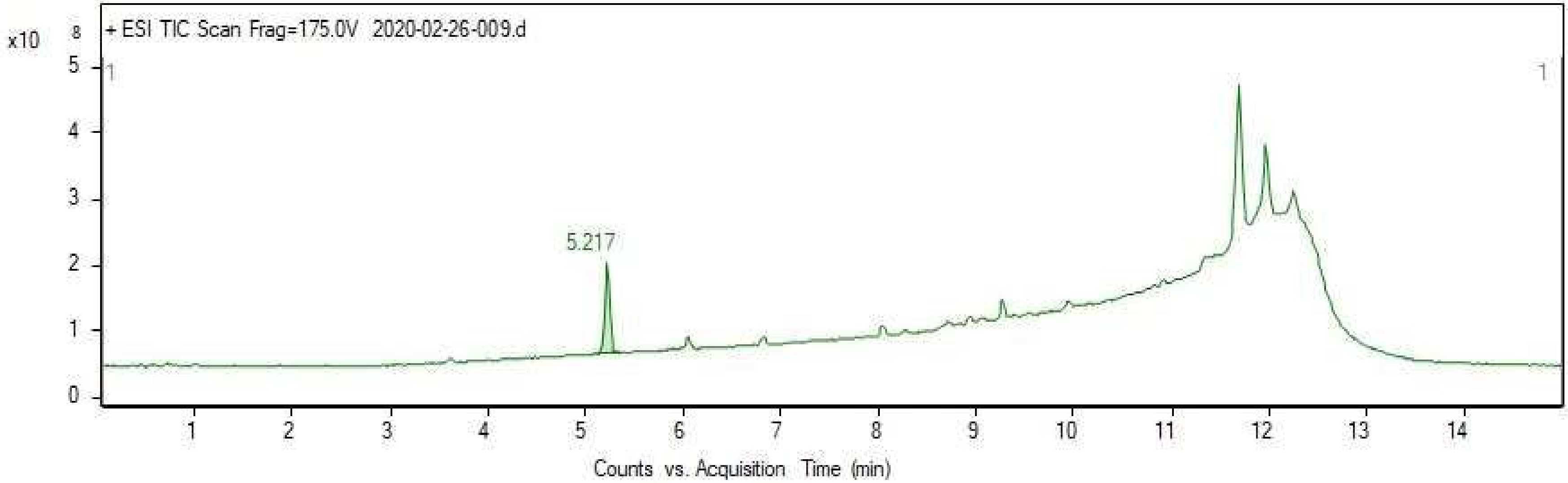

x10 8
+ESI TIC Scan Frag=175.0V 2020-02-26-009.d
5.217
Counts vs. Acquisition Time (min)

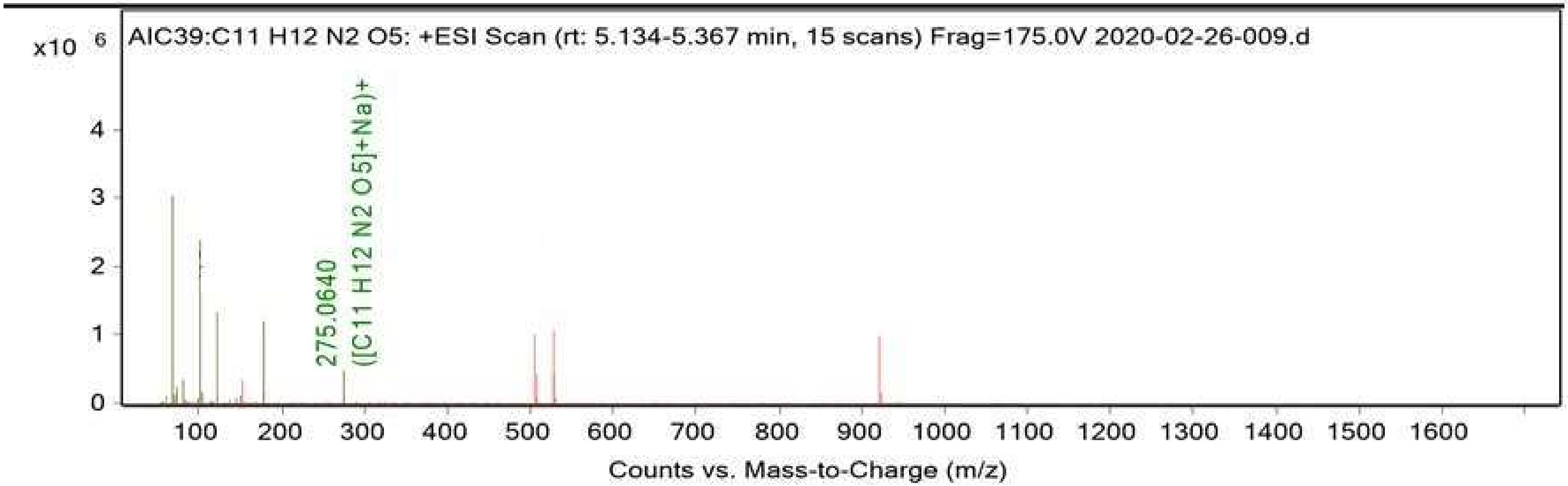

x10 6
AIC39:C11 H12 N2 O5: +ESI Scan (rt: 5.134-5.367 min, 15 scans) Frag=175.0V 2020-02-26-009.d
275.0640
([C11 H12 N2 O5]+Na)+
Counts vs. Mass-to-Charge (m/z)

^{1}H NMR

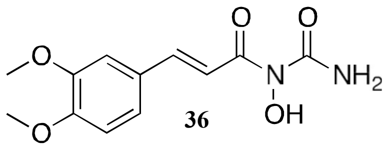

36

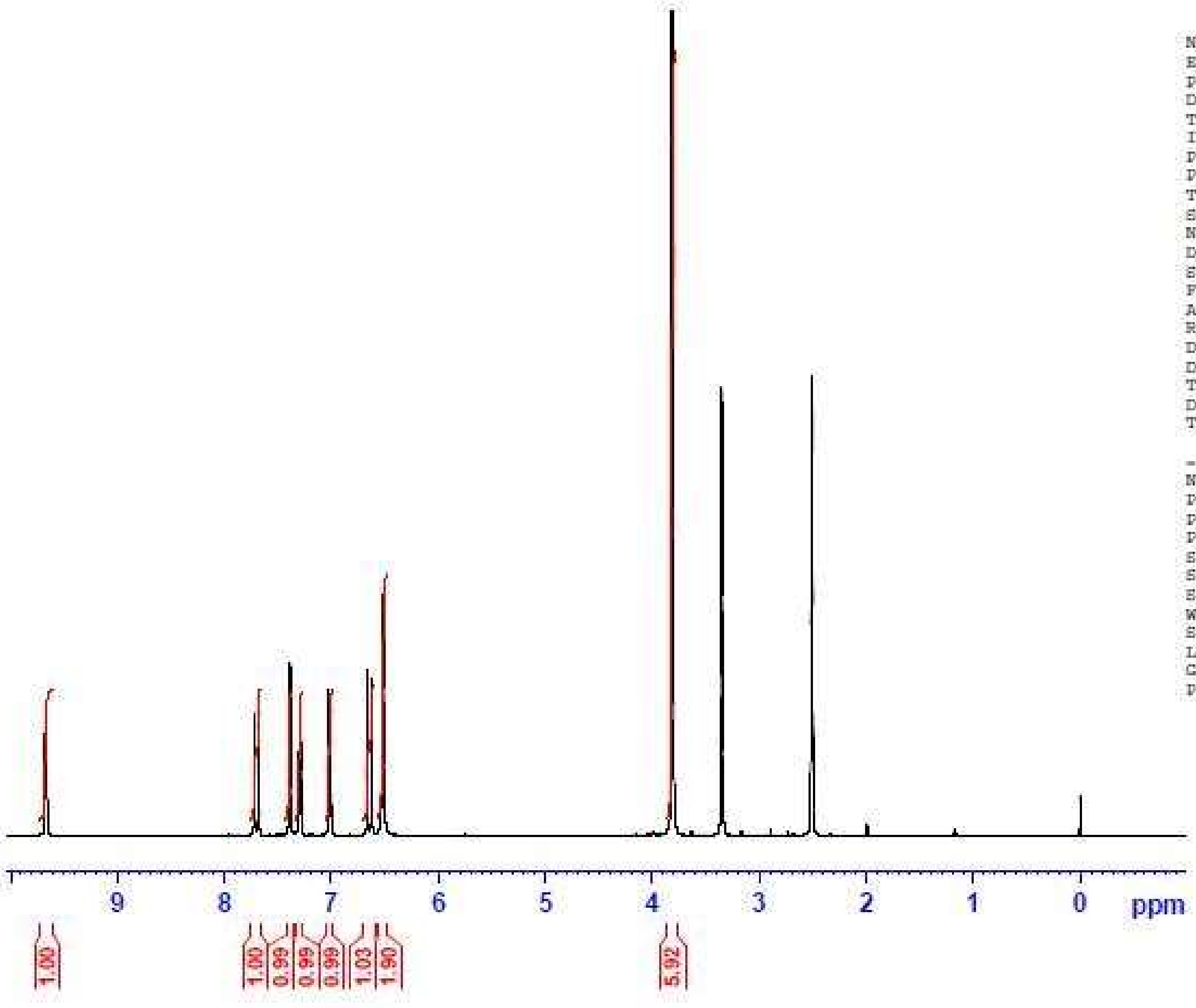

BRUKER

NAME	Audrey Isabelle
EXPNO	94
PROCNO	1
Date_	20190628
Time	16.15
INSTRUM	spect
PROBHD	5 mm PABBO BB-
PULPROG	zg30
TD	65536
SOLVENT	DMSO
NS	16
DS	2
SWH	8223.685 Hz
FIDRES	0.125483 Hz
AQ	3.9846387 sec
RG	114
DW	60.800 usec
DE	6.50 usec
TE	298.0 K
D1	1.00000000 sec
TD0	1
======== CHANNEL f1 ========	
NUC1	1H
P1	14.07 usec
PL1	0.30 dB
PL1W	11.25229836 W
SFO1	400.1324710 MHz
SI	32768
SF	400.1300000 MHz
WDW	EM
SSB	0
LB	0.30 Hz
GB	0
PC	1.00

^{13}C NMR

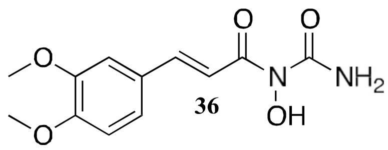

36

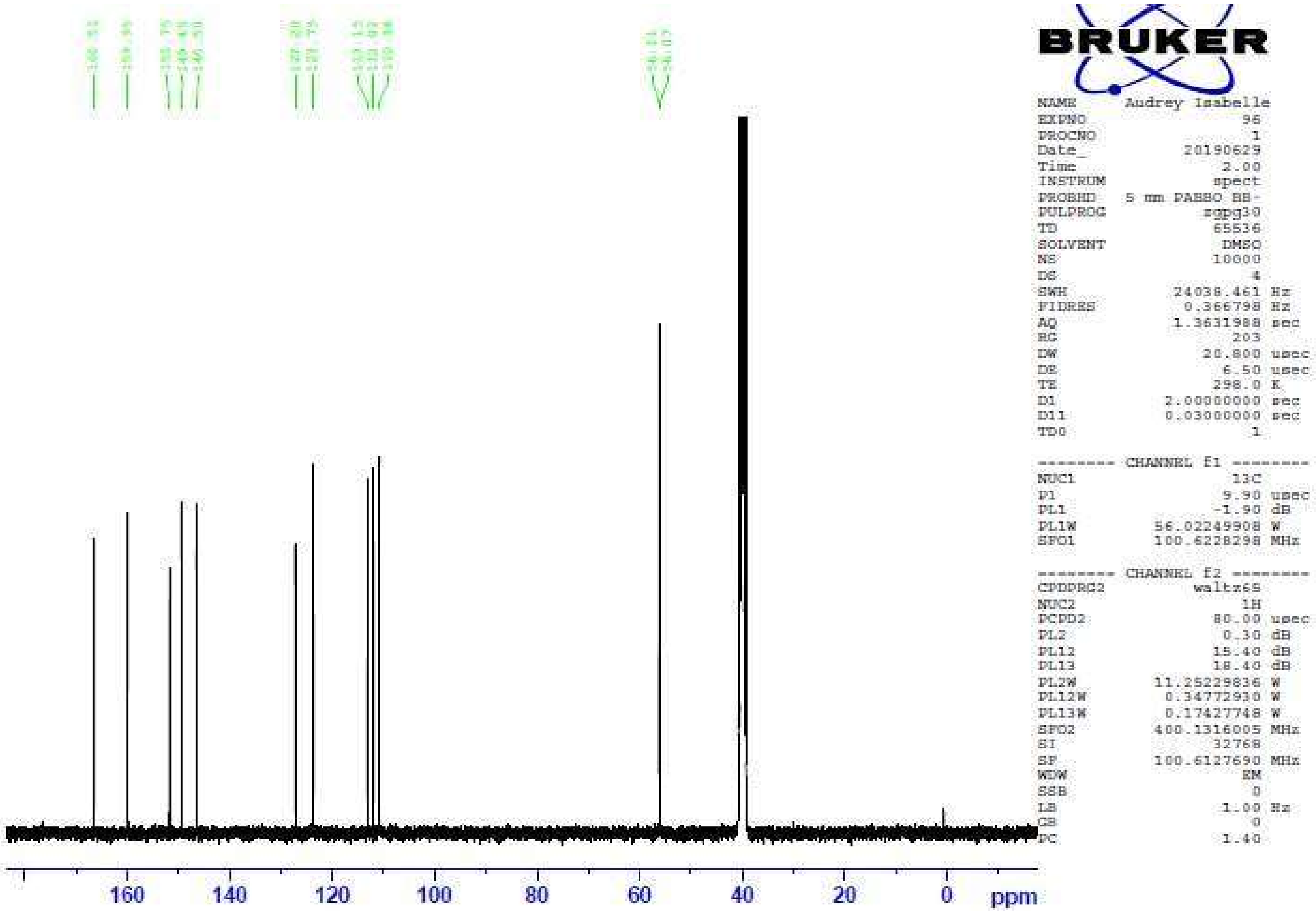
BRUKER
NAME Audrey Isabelle
EXPNO 96
PROCNO 1
Date_ 20190629
Time 2.00
INSTRUM spect
PROBHD 5 mm PABBO BB-
PULPROG zgpg30
TD 65536
SOLVENT DMSO
NS 10000
DS 4
SWH 24038.461 Hz
FIDRES 0.366798 Hz
AQ 1.3631988 sec
RG 203
DW 20.800 usec
DE 6.50 usec
TE 298.0 K
D1 2.00000000 sec
D11 0.03000000 sec
TD0 1
======== CHANNEL f1 ========
NUC1 13C
P1 9.90 usec
PL1 -1.90 dB
PL1W 56.02249908 W
SFO1 100.6228298 MHz
======== CHANNEL f2 ========
CPDPRG2 waltz16
NUC2 1H
PCPD2 80.00 usec
PL2 0.30 dB
PL12 15.40 dB
PL13 18.40 dB
PL2W 11.25229836 W
PL12W 0.34772930 W
PL13W 0.17427748 W
SFO2 400.1316005 MHz
SI 32768
SF 100.6127690 MHz
WDW EM
SSB 0
LB 1.00 Hz
GB 0
PC 1.40
160
140
120
100
80
60
40
20
0
ppm

HRMS

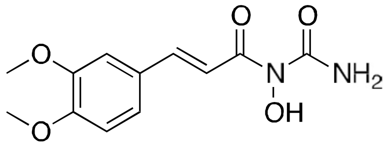
O
O
O
N
NH2
OH
O

36

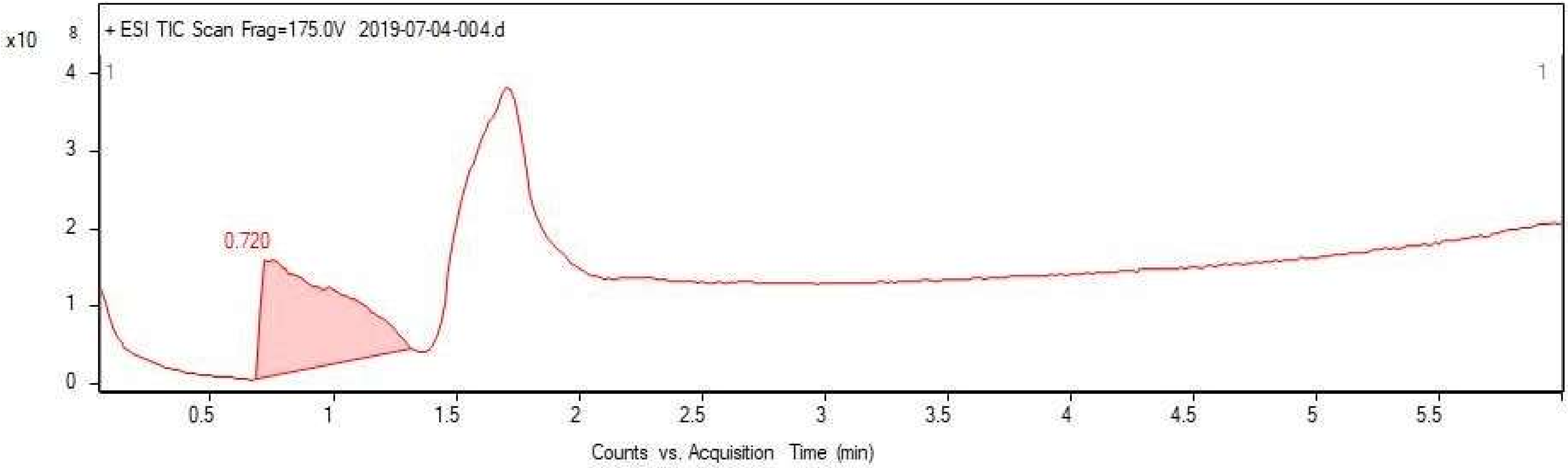
x10 8
+ ESI TIC Scan Frag=175.0V 2019-07-04-004.d
0.720
Counts vs. Acquisition Time (min)

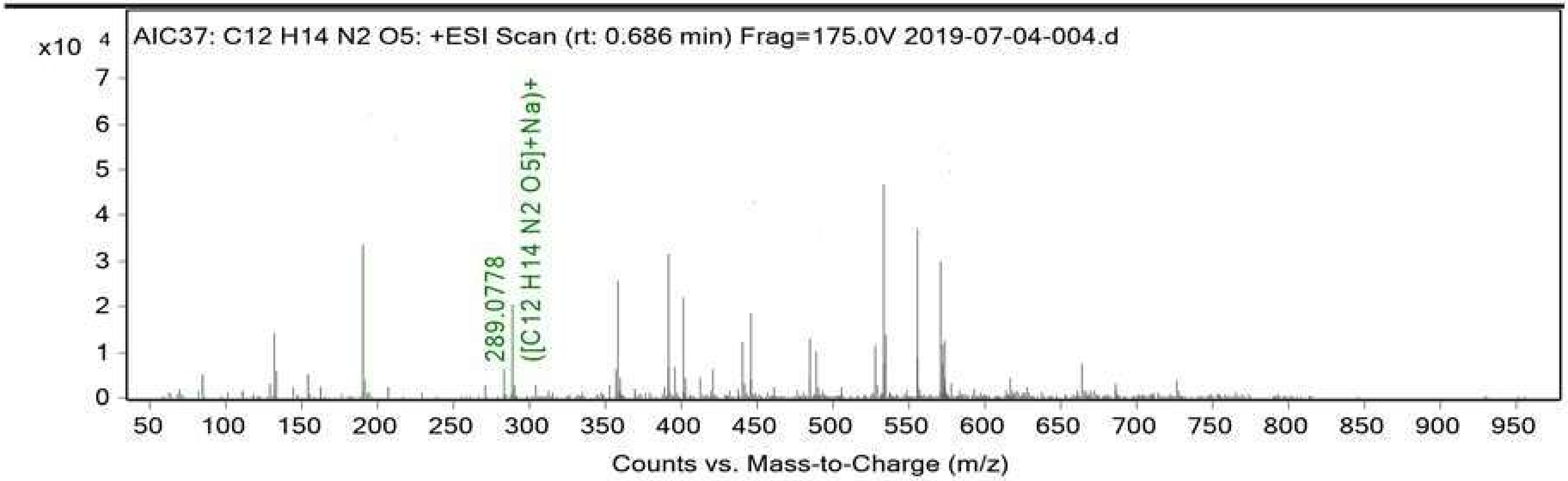
x10 4
AIC37: C12 H14 N2 O5: +ESI Scan (rt: 0.686 min) Frag=175.0V 2019-07-04-004.d
289.0778
([C12 H14 N2 O5]+Na)+
Counts vs. Mass-to-Charge (m/z)

^{1}H NMR

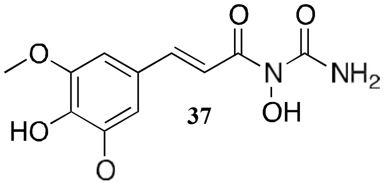

37

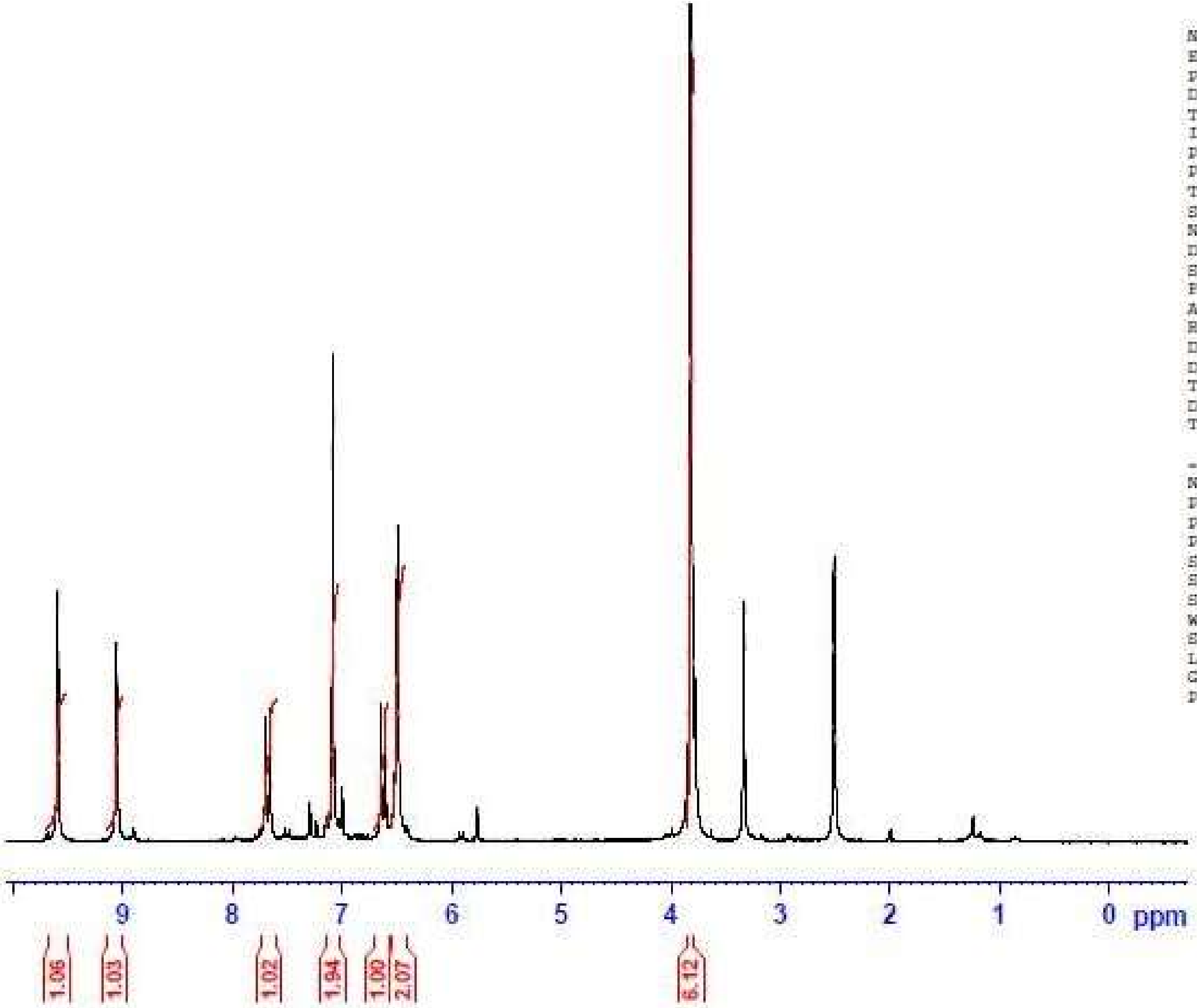

NAME	Audrey Isabelle
EXPNO	99
PROCNO	1
Date_	20190704
Time	8.17
INSTRUM	spect
PROBHD	5 mm PABBO BB-
PULPROG	zg30
TD	65536
SOLVENT	DMSO
NS	16
DS	2
SWH	8223.685 Hz
FIDRES	0.125483 Hz
AQ	3.9846387 sec
RG	114
DW	60.800 usec
DE	6.50 usec
TE	298.0 K
D1	1.00000000 sec
TD0	1
======== CHANNEL f1 ========	
NUC1	1H
P1	14.07 usec
PL1	0.30 dB
PL1W	11.25229836 W
SFO1	400.1324710 MHz
SI	32768
SF	400.1300000 MHz
WDW	EM
SSB	0
LB	0.30 Hz
GB	0
PC	1.00

^{13}C NMR

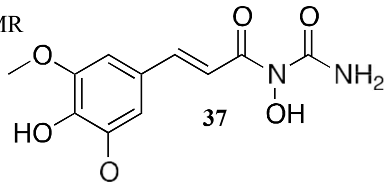

O
O
O
N
NH2
HO
OH
O
37

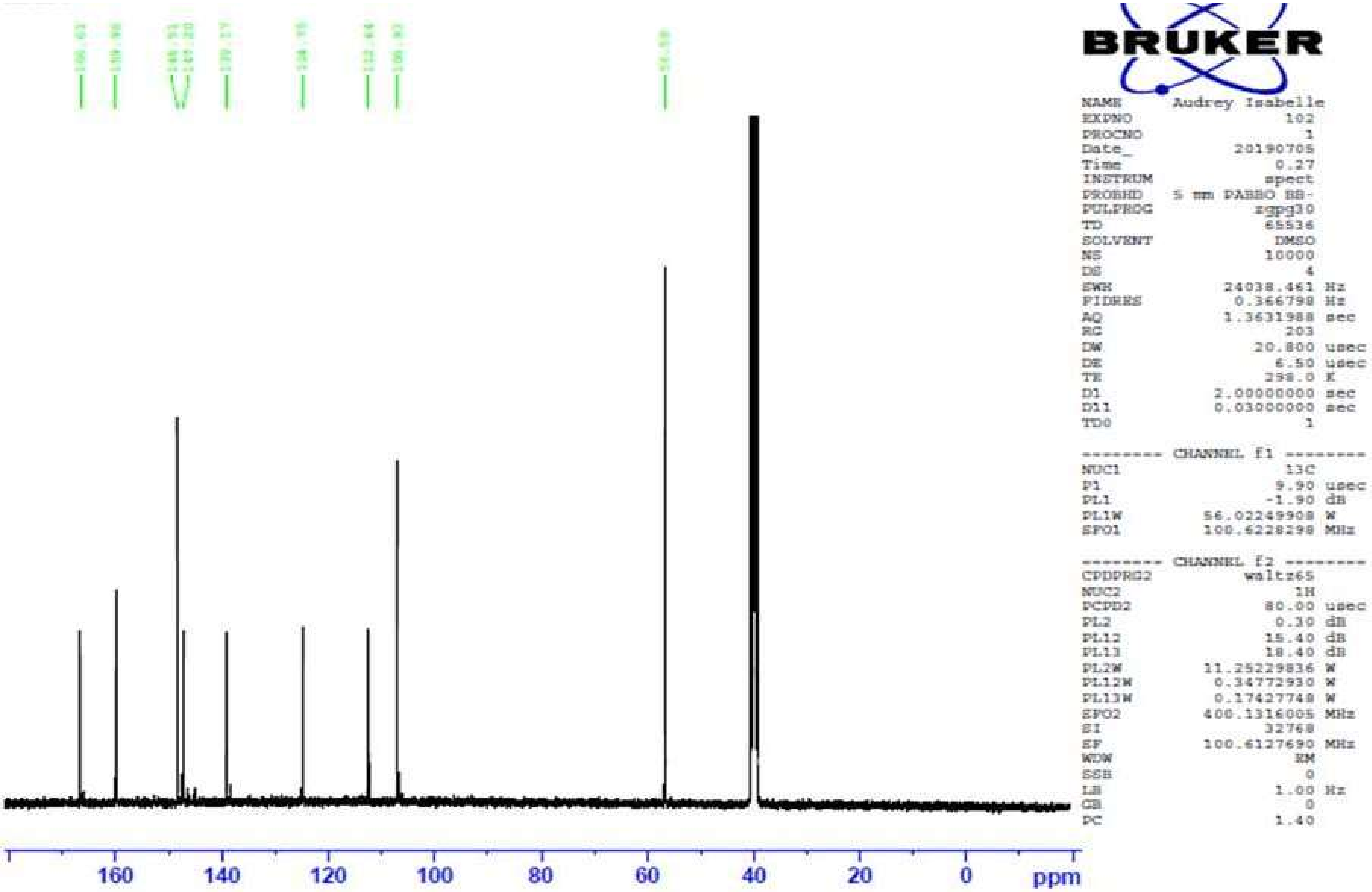

160
140
120
100
80
60
40
20
0
ppm
BRUKER
NAME Audrey Isabelle
EXPNO 102
PROCNO 1
Date_ 20190705
Time 0.27
INSTRUM spect
PROBHD 5 mm PABBO BB-
PULPROG zgpg30
TD 65536
SOLVENT DMSO
NS 10000
DS 4
SWH 24038.461 Hz
FIDRES 0.366798 Hz
AQ 1.3631988 sec
RG 203
DW 20.800 usec
DE 6.50 usec
TE 298.0 K
D1 2.00000000 sec
D11 0.03000000 sec
TD0 1
======== CHANNEL f1 ========
NUC1 13C
P1 9.90 usec
PL1 -1.90 dB
PL1W 56.02249908 W
SFO1 100.6228298 MHz
======== CHANNEL f2 ========
CPDPRG2 waltz65
NUC2 1H
PCPD2 80.00 usec
PL2 0.30 dB
PL12 15.40 dB
PL13 18.40 dB
PL2W 11.25229836 W
PL12W 0.34772930 W
PL13W 0.17427748 W
SFO2 400.1316005 MHz
SI 32768
SF 100.6127690 MHz
WDW EM
SSB 0
LB 1.00 Hz
GB 0
PC 1.40

HRMS

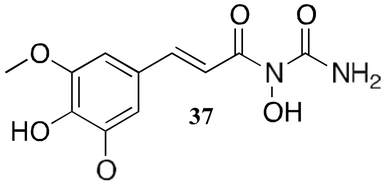

O
O
O
N
NH2
HO
OH
O

37

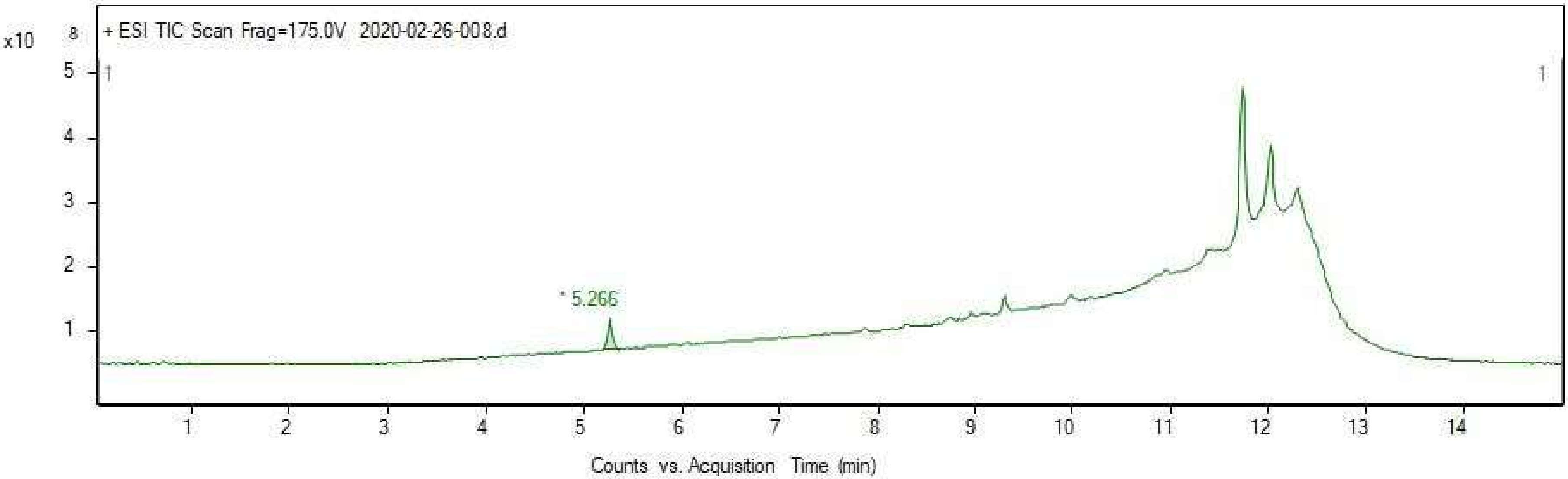

+ ESI TIC Scan Frag=175.0V 2020-02-26-008.d
x10 8
* 5.266
Counts vs. Acquisition Time (min)

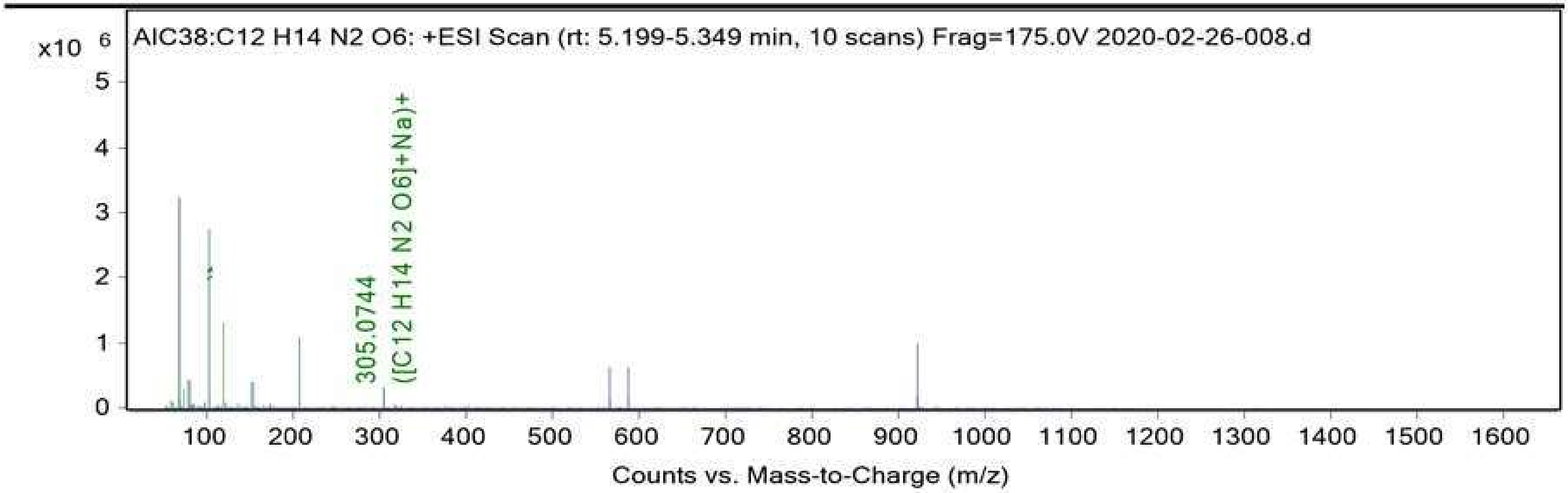

AIC38:C12 H14 N2 O6: +ESI Scan (rt: 5.199-5.349 min, 10 scans) Frag=175.0V 2020-02-26-008.d
x10 6
305.0744
([C12 H14 N2 O6]+Na)+
Counts vs. Mass-to-Charge (m/z)

^{1}H NMR

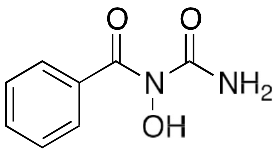

O
O
N
NH2
OH

38

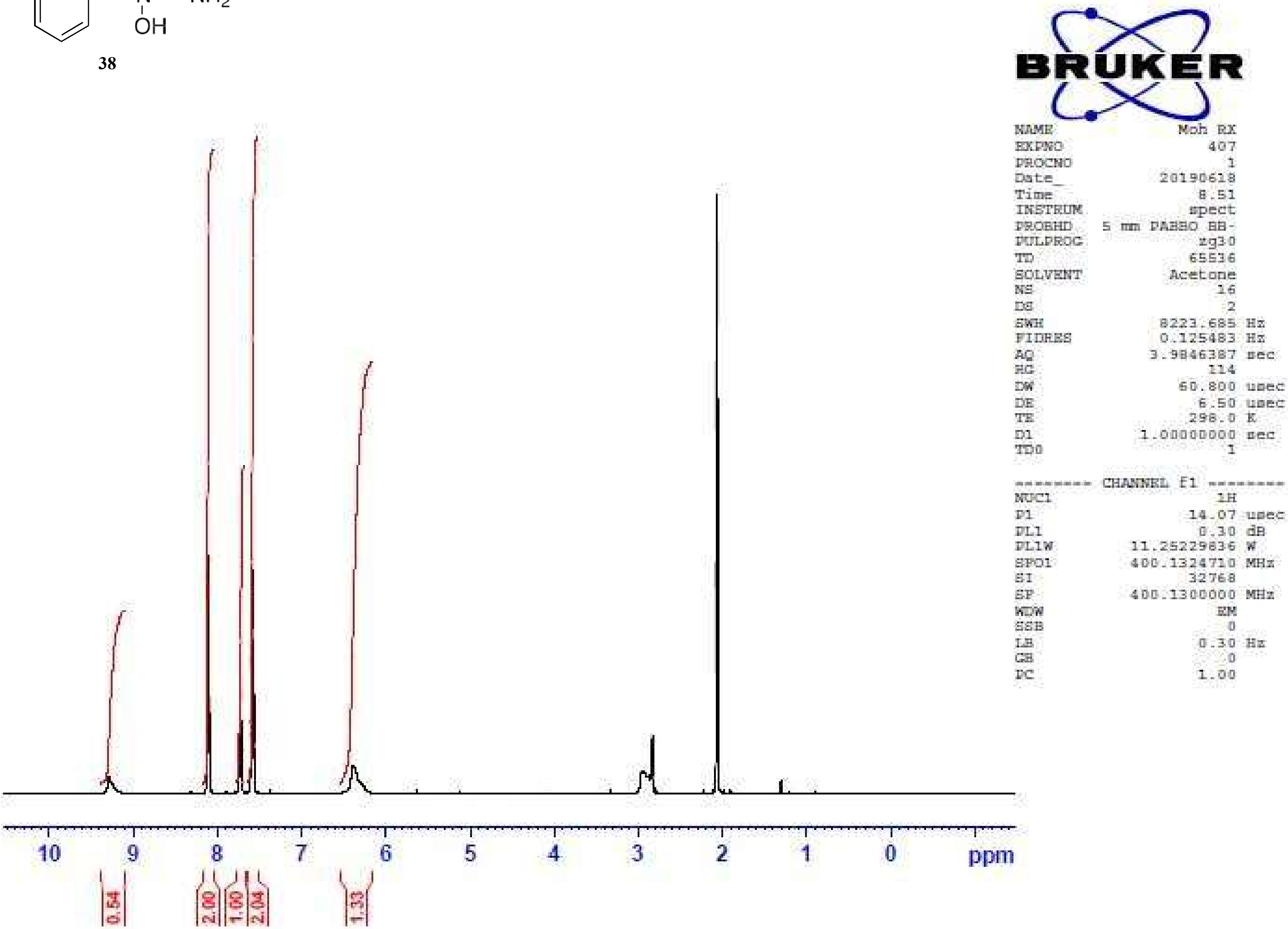

BRUKER
NAME Moh RX
EXPNO 407
PROCNO 1
Date_ 20190618
Time 8.51
INSTRUM spect
PROBHD 5 mm PABBO BB-
PULPROG zg30
TD 65536
SOLVENT Acetone
NS 16
DS 2
SWH 8223.685 Hz
FIDRES 0.125483 Hz
AQ 3.9846387 sec
RG 114
DW 60.800 usec
DE 6.50 usec
TE 298.0 K
D1 1.00000000 sec
TD0 1
======== CHANNEL f1 ========
NUC1 1H
P1 14.07 usec
PL1 0.30 dB
PL1W 11.25229636 W
SFO1 400.1324710 MHz
SI 32768
SF 400.1300000 MHz
WDW EM
SSB 0
LB 0.30 Hz
GB 0
PC 1.00
10 9 8 7 6 5 4 3 2 1 0 ppm
0.54
2.00
1.00
2.04
1.33

^{13}C NMR

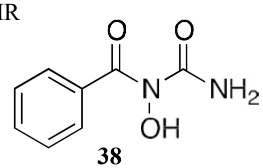
O
O
N
NH2
OH

38

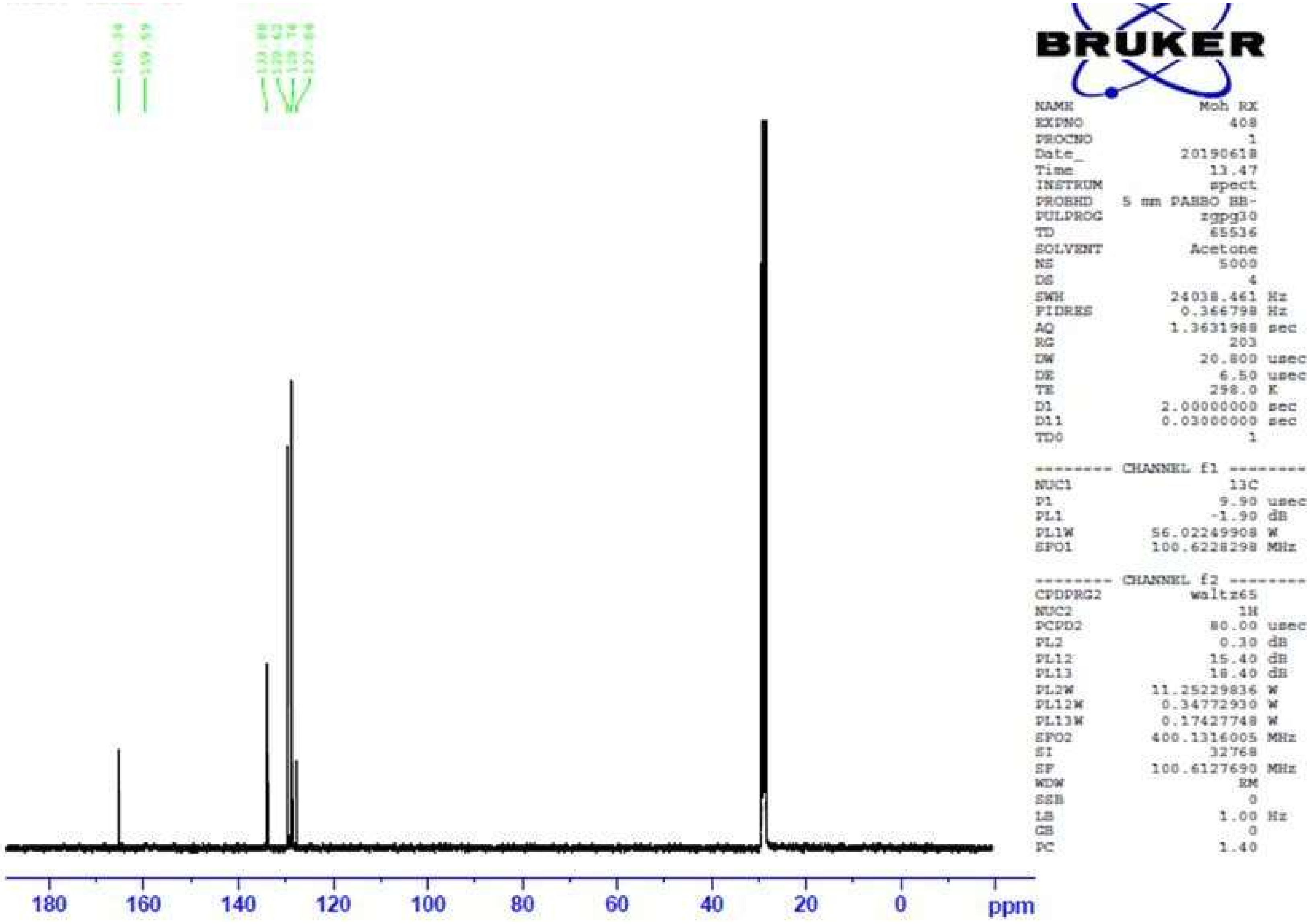
BRUKER
NAME Moh RX
EXPNO 408
PROCNO 1
Date_ 20190618
Time 13.47
INSTRUM spect
PROBHD 5 mm PABBO BB-
PULPROG zgpg30
TD 65536
SOLVENT Acetone
NS 5000
DS 4
SWH 24038.461 Hz
FIDRES 0.366798 Hz
AQ 1.3631988 sec
RG 203
DW 20.800 usec
DE 6.50 usec
TE 298.0 K
D1 2.00000000 sec
D11 0.03000000 sec
TD0 1
======== CHANNEL f1 ========
NUC1 13C
P1 9.90 usec
PL1 -1.90 dB
PL1W 56.02249908 W
SFO1 100.6228298 MHz
======== CHANNEL f2 ========
CPDPRG2 waltz65
NUC2 1H
PCPD2 80.00 usec
PL2 0.30 dB
PL12 15.40 dB
PL13 18.40 dB
PL2W 11.25229836 W
PL12W 0.34772930 W
PL13W 0.17427748 W
SFO2 400.1316005 MHz
SI 32768
SF 100.6127690 MHz
WDW EM
SSB 0
LB 1.00 Hz
GB 0
PC 1.40
180
160
140
120
100
80
60
40
20
0
ppm

HRMS

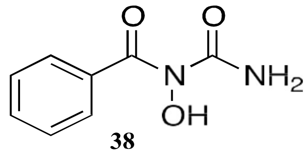
O
O
N
NH2
OH

38

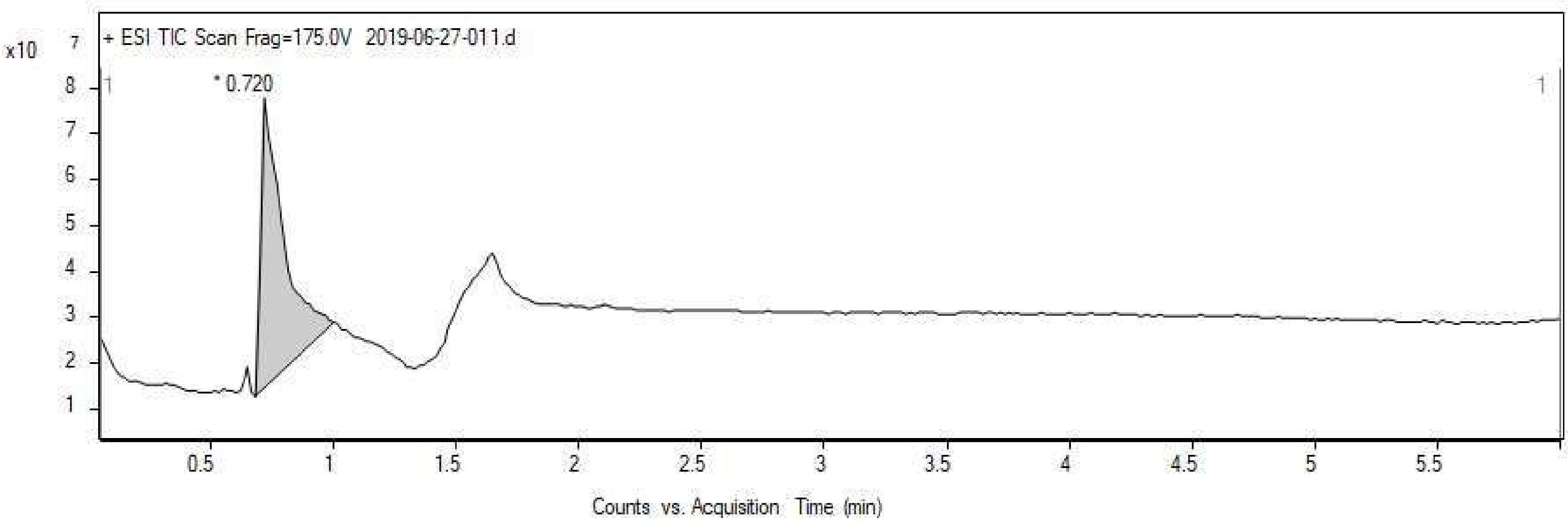
+ ESI TIC Scan Frag=175.0V 2019-06-27-011.d
x10 7
* 0.720
Counts vs. Acquisition Time (min)

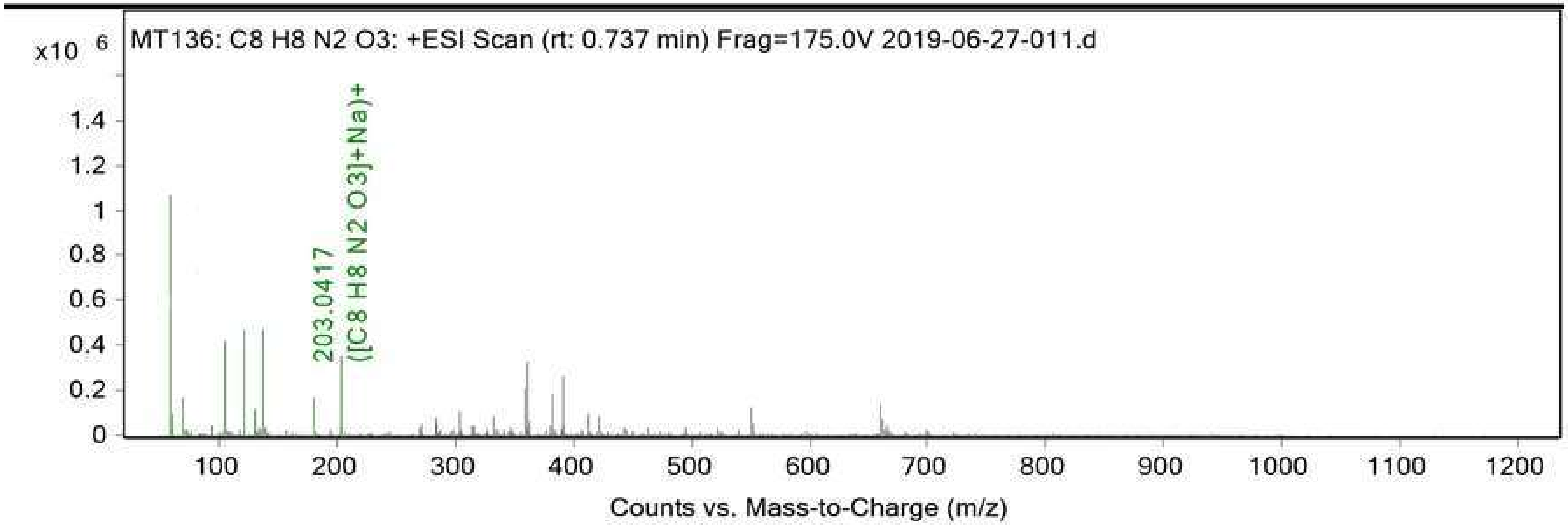
MT136: C8 H8 N2 O3: +ESI Scan (rt: 0.737 min) Frag=175.0V 2019-06-27-011.d
x10 6
203.0417
([C8 H8 N2 O3]+Na)+
Counts vs. Mass-to-Charge (m/z)

^{1}H NMR

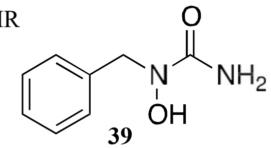

39

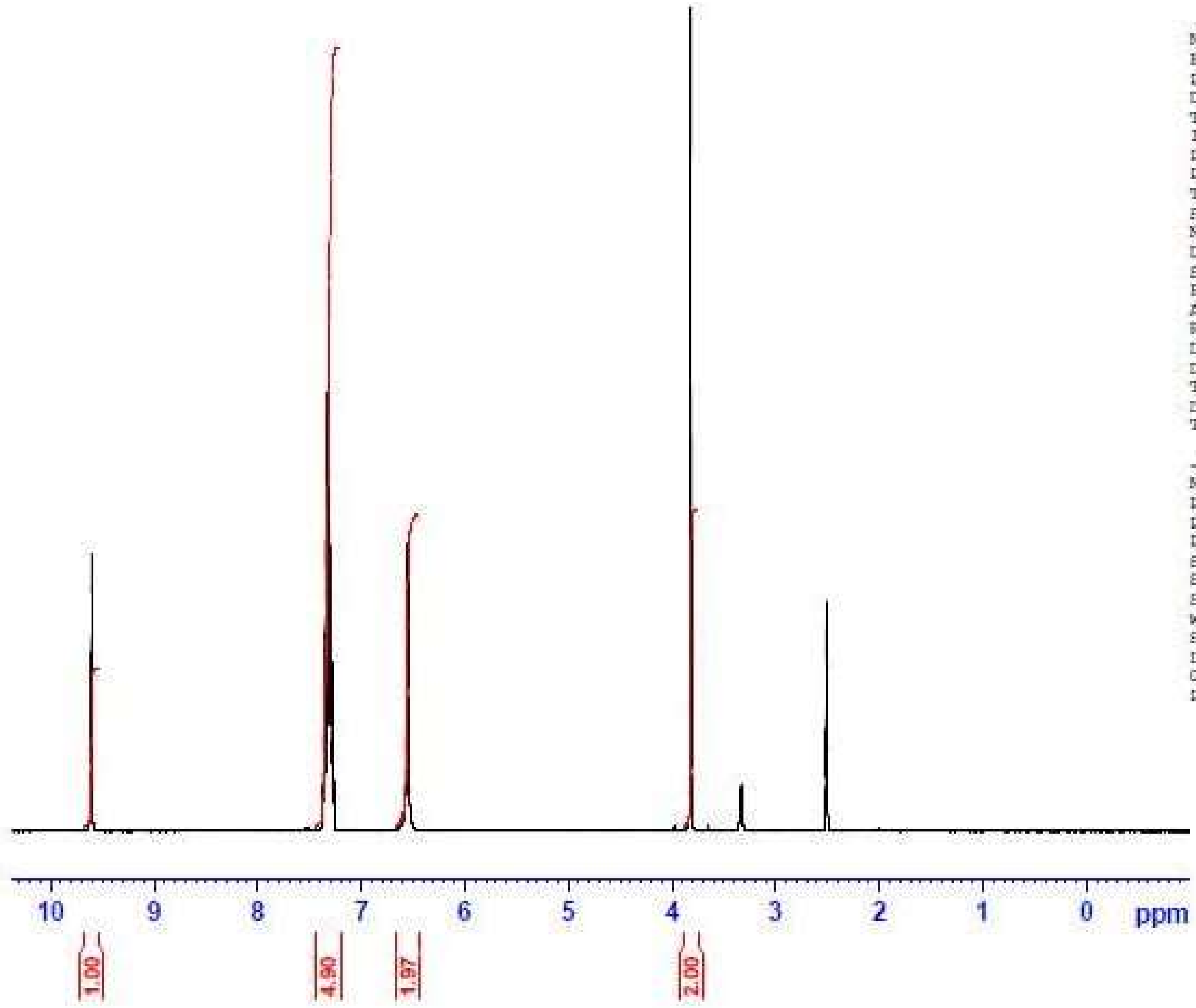

BRUKER

NAME	Moh RX
EXPNO	426
PROCNO	1
Date_	20190702
Time	10.20
INSTRUM	spect
PROBHD	5 mm PABBO BB-
PULPROG	zg30
TD	65536
SOLVENT	DMSO
NS	16
DS	2
SWH	8223.685 Hz
FIDRES	0.125483 Hz
AQ	3.9846387 sec
RG	114
DW	60.800 usec
DE	6.50 usec
TE	298.0 K
D1	1.00000000 sec
TD0	1

======== CHANNEL f1 ========

NUC1	1H
P1	14.07 usec
PL1	0.30 dB
PL1W	11.25229836 W
SFO1	400.1324710 MHz
SI	32768
SF	400.1300000 MHz
WDW	EM
SSB	0
LB	0.30 Hz
GB	0
PC	1.00

^{13}C NMR

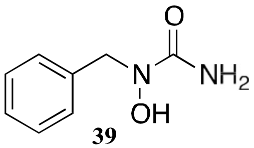
O
N
NH2
OH

39

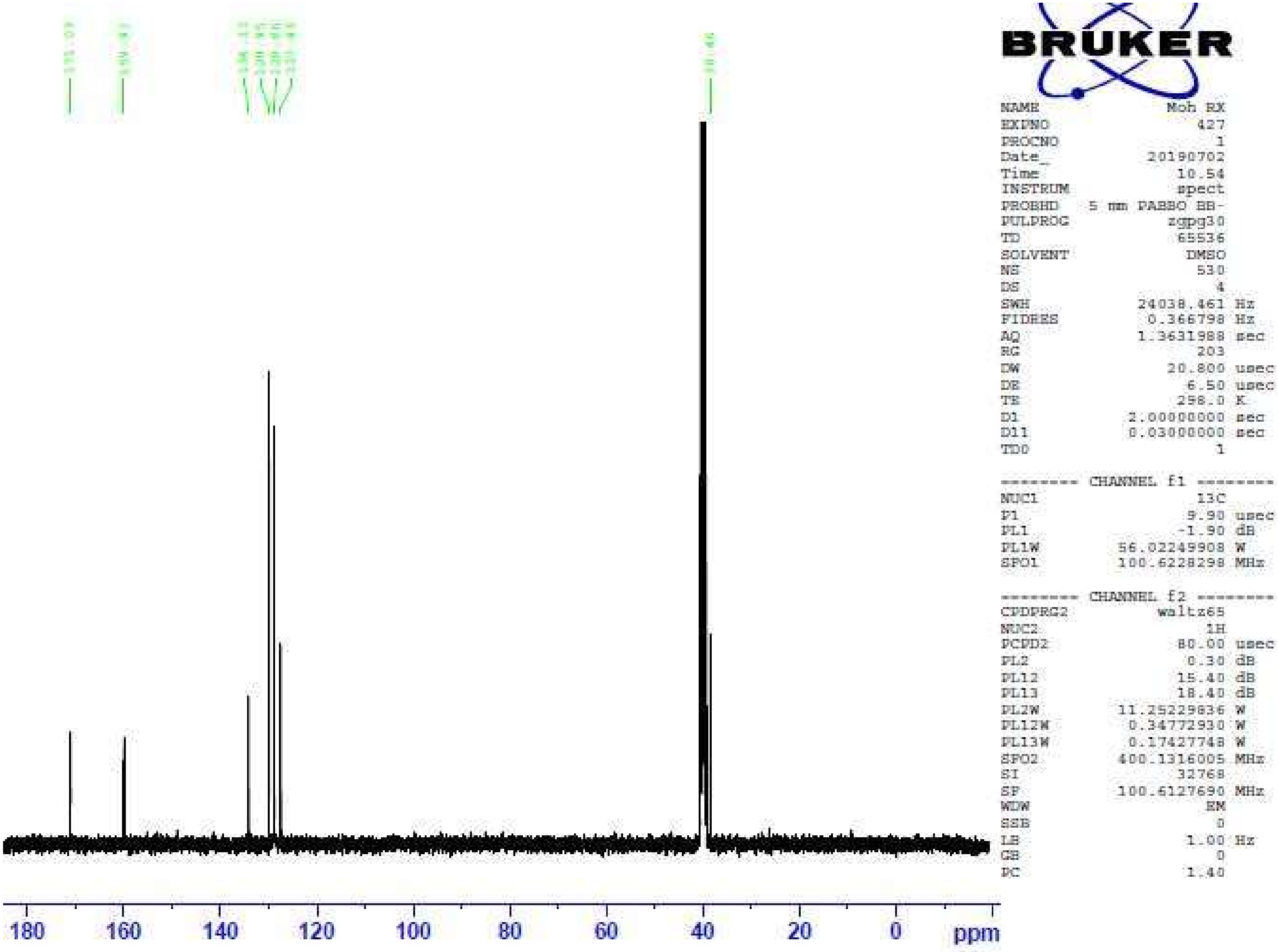
BRUKER
NAME Moh RX
EXPNO 427
PROCNO 1
Date_ 20190702
Time 10.54
INSTRUM spect
PROBHD 5 mm PABBO BB-
PULPROG zgpg30
TD 65536
SOLVENT DMSO
NS 530
DS 4
SWH 24038.461 Hz
FIDRES 0.366798 Hz
AQ 1.3631988 sec
RG 203
DW 20.800 usec
DE 6.50 usec
TE 298.0 K
D1 2.00000000 sec
D11 0.03000000 sec
TD0 1
======== CHANNEL f1 ========
NUC1 13C
P1 9.90 usec
PL1 -1.90 dB
PL1W 56.02249908 W
SFO1 100.6228298 MHz
======== CHANNEL f2 ========
CPDPRG2 waltz65
NUC2 1H
PCPD2 80.00 usec
PL2 0.30 dB
PL12 15.40 dB
PL13 18.40 dB
PL2W 11.25229836 W
PL12W 0.34772930 W
PL13W 0.17427748 W
SFO2 400.1316005 MHz
SI 32768
SF 100.6127690 MHz
WDW EM
SSB 0
LB 1.00 Hz
GB 0
PC 1.40
180
160
140
120
100
80
60
40
20
0
ppm

^{1}H NMR

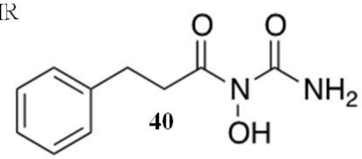

40

BRUKER

NAME	Moh RX	
EXPNO	410	
PROCNO	1	
Date_	20190620	
Time	8.51	
INSTRUM	spect	
PROBHD	5 mm PABBO BB-	
PULPROG	zg30	
TD	65536	
SOLVENT	DMSO	
NS	16	
DS	2	
SWH	8223.685	Hz
FIDRES	0.125483	Hz
AQ	3.9846387	sec
RG	101	
DW	60.800	usec
DE	6.50	usec
TE	298.0	K
D1	1.00000000	sec
TD0	1	

======== CHANNEL f1 ========		
NUC1	1H	
P1	14.07	usec
PL1	0.30	dB
PL1W	11.25229836	W
SFO1	400.1324710	MHz
SI	32768	
SF	400.1300000	MHz
WDW	EM	
SSB	0	
LB	0.30	Hz
GB	0	
PC	1.00	

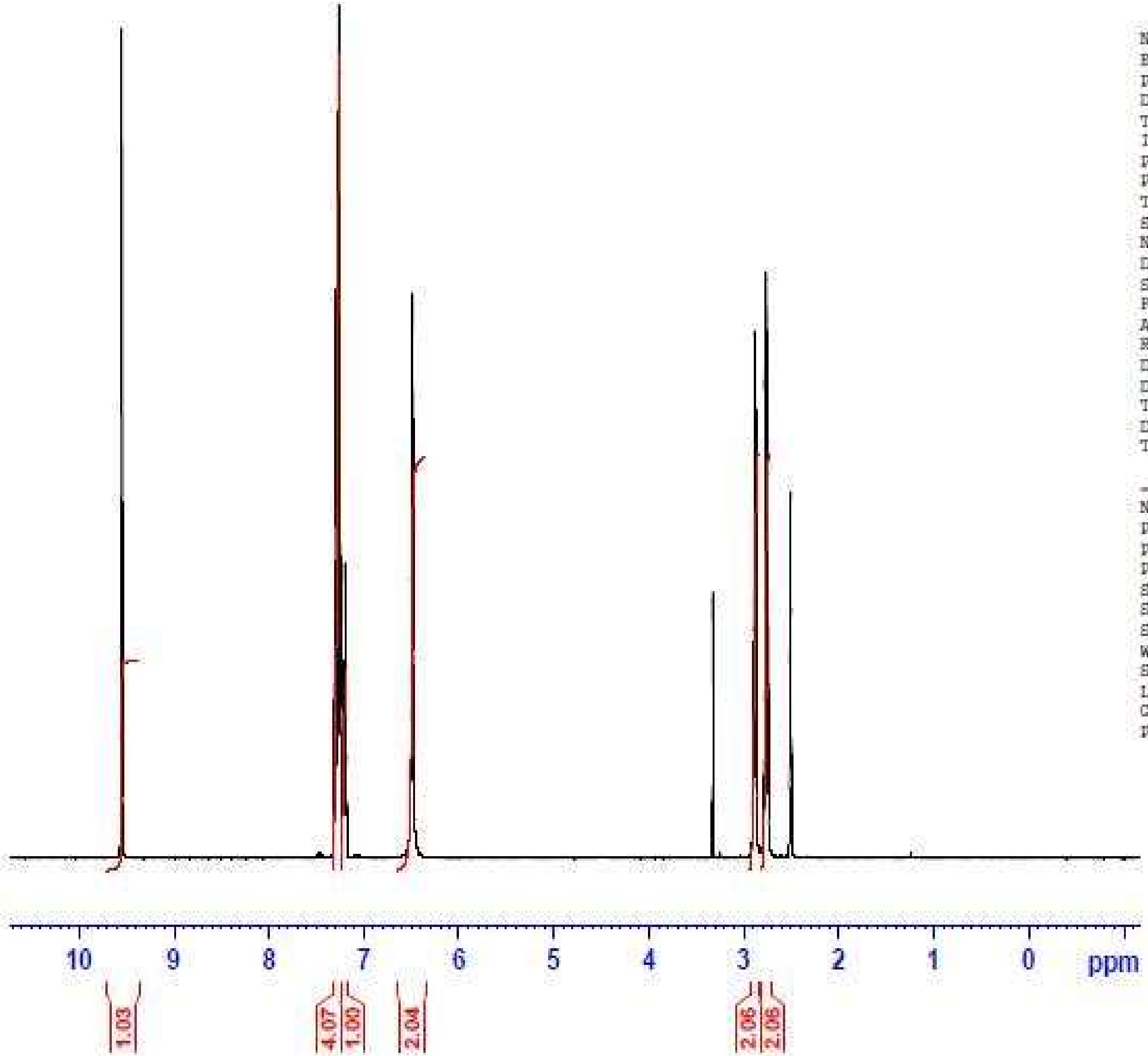

^{13}C NMR

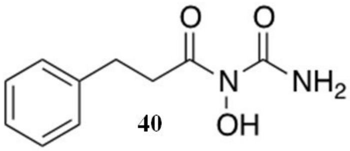
O
O
N
NH2
OH

40

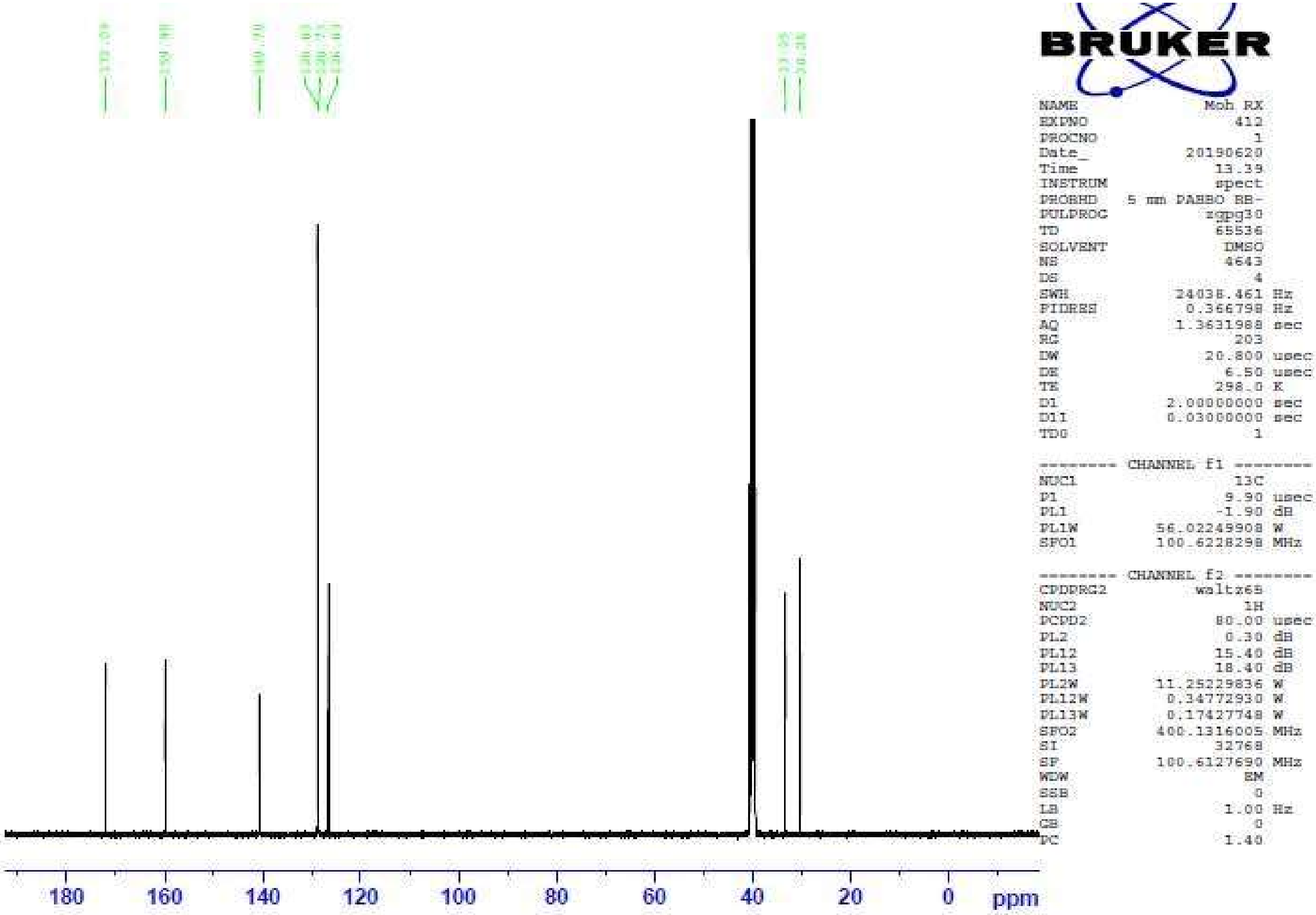
BRUKER
NAME Moh RX
EXPNO 412
PROCNO 1
Date_ 20190620
Time 13.39
INSTRUM spect
PROBHD 5 mm PABBO BB-
PULPROG zgpg30
TD 65536
SOLVENT DMSO
NS 4643
DS 4
SWH 24038.461 Hz
FIDRES 0.366798 Hz
AQ 1.3631988 sec
RG 203
DW 20.800 usec
DE 6.50 usec
TE 298.0 K
D1 2.00000000 sec
D11 0.03000000 sec
TD0 1
======== CHANNEL f1 ========
NUC1 13C
P1 9.90 usec
PL1 -1.90 dB
PL1W 56.02249908 W
SFO1 100.6228298 MHz
======== CHANNEL f2 ========
CPDPRG2 waltz65
NUC2 1H
PCPD2 80.00 usec
PL2 0.30 dB
PL12 15.40 dB
PL13 18.40 dB
PL2W 11.25229836 W
PL12W 0.34772930 W
PL13W 0.17427748 W
SFO2 400.1316005 MHz
SI 32768
SF 100.6127690 MHz
WDW EM
SSB 0
LB 1.00 Hz
GB 0
PC 1.40
180
160
140
120
100
80
60
40
20
0
ppm

HRMS

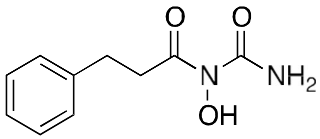

O
O
N
NH2
OH

40

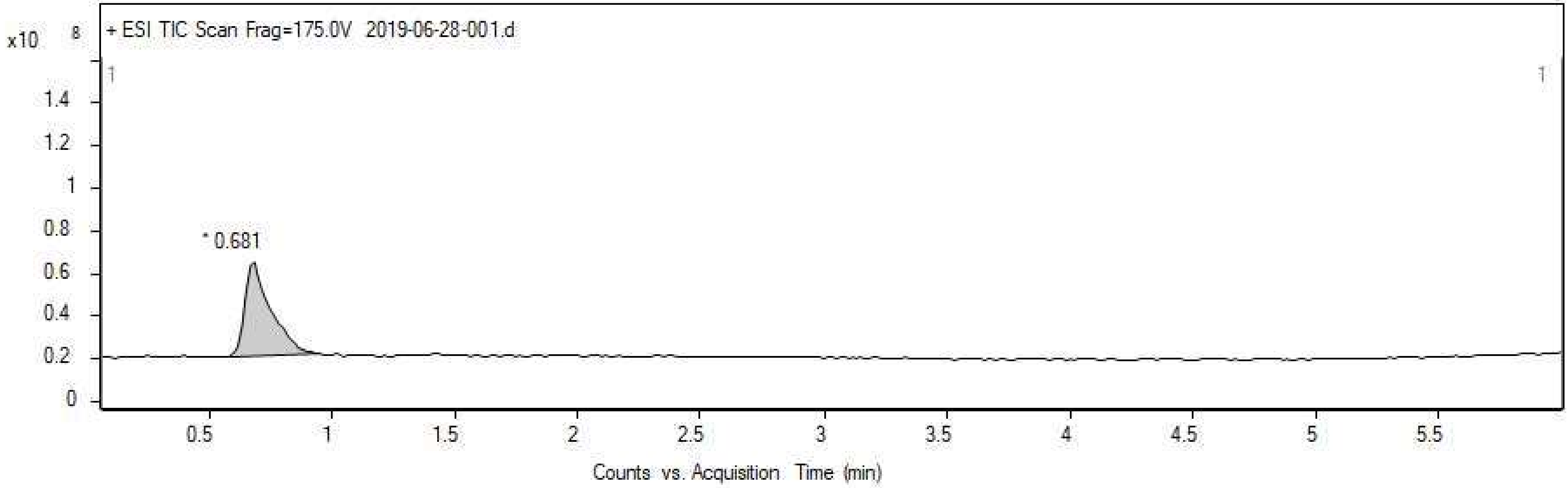

x10 8
+ ESI TIC Scan Frag=175.0V 2019-06-28-001.d
1.4
1.2
1
0.8
0.6
0.4
0.2
0
* 0.681
0.5
1
1.5
2
2.5
3
3.5
4
4.5
5
5.5
Counts vs. Acquisition Time (min)

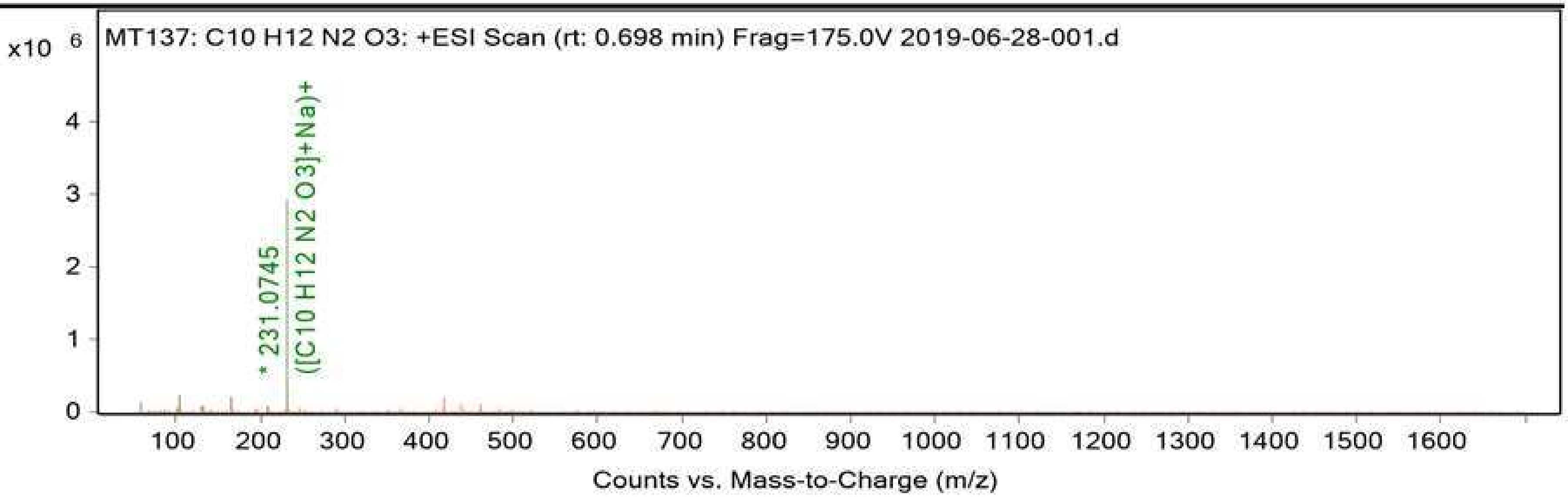

x10 6
MT137: C10 H12 N2 O3: +ESI Scan (rt: 0.698 min) Frag=175.0V 2019-06-28-001.d
* 231.0745
([C10 H12 N2 O3]+Na)+
4
3
2
1
0
100
200
300
400
500
600
700
800
900
1000
1100
1200
1300
1400
1500
1600
Counts vs. Mass-to-Charge (m/z)
